# Functional Group Transposition Enabled by Palladium and Photo Dual Catalysis

**DOI:** 10.1021/jacs.5c11429

**Published:** 2025-10-27

**Authors:** Menghua Xu, Chengjun Wu, Ming Chen

**Affiliations:** Department of Chemistry, 1757 Virginia Tech, Blacksburg, Virginia 24061, United States

## Abstract

The ability to precisely modify the structure of molecules is a captivating process that has fascinated the synthetic organic and medicinal chemistry communities. To this end, functional group transposition has recently emerged as a powerful strategy to edit molecules and allow for access to novel chemical entities without significantly altering the synthesis routes. Here we disclose an unusual functional group transposition reaction. By using palladium and photo dual catalysis, this radical-induced process enables the transposition between an iodo group and a boryl group to convert iodoarenes appended with an alkylboronate group to arylboronates appended with an alkyl iodide.

## Graphical abstract

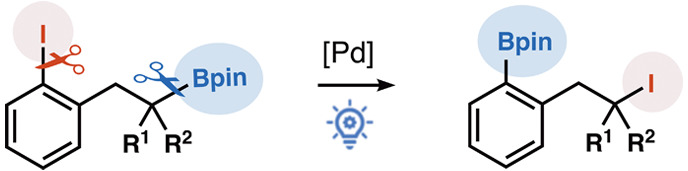

The ability to precisely modify the structure of molecules, particularly in the late stage, has become a captivating process that fascinates the synthetic organic chemistry and medicinal chemistry communities.^1^ By adding, removing, and exchanging atoms or functional groups of a molecule, the strategy allows for rapid generation of downstream products without or with minimum alteration of the synthesis routes. Meanwhile, the process also holds great potential in drug discovery and development, as it could facilitate the structure–activity relationship (SAR) studies to improve the potency, drug-like properties, and metabolic stabilities of the candidates.^2^

Functional group transposition has recently emerged as a powerful tool to edit molecules and allow for access to novel chemical entities.^3–6^ The traditional functional group transformations involve converting one functional group into another, which serves as one important foundation in modern organic synthesis.^7^ By contrast, functional group transposition centers on relocating a preexisting functional group to a new position or exchanging the locations of two functional groups. An appealing feature of this process is that it often simplifies the approaches to the targeted molecules or permits access to molecules that are otherwise difficult to synthesize. However, examples of catalytic functional group transposition are rare, and there is a critical need to enrich the toolbox of such transformations.

As depicted in Scheme 1, several strategies for functional group transposition have been achieved over the past few years. For example, the Dong group developed an innovative carbonyl group translocation.^3a^ By means of a Pd-catalyzed amination process, α-tetralone **A** was converted into β-tetralone **C** via the intermediacy of vinyl triflate **B** (Scheme 1a). Morandi and co-workers showed that diaryl thioethers **D** and **E** could produce mixed diaryl thioether **F** through Pd-catalyzed reversible oxidative addition–reductive elimination processes (Scheme 1b).^4a^ In parallel, the Arndtsen and Morandi groups simultaneously reported the σ-bond metathesis of aryl iodides and acid chlorides via similar Pd-catalyzed reversible oxidative addition–reductive elimination processes, allowing for the syntheses of acid chlorides **I** from benzoyl chloride **G**.^5^

**Scheme 1:**
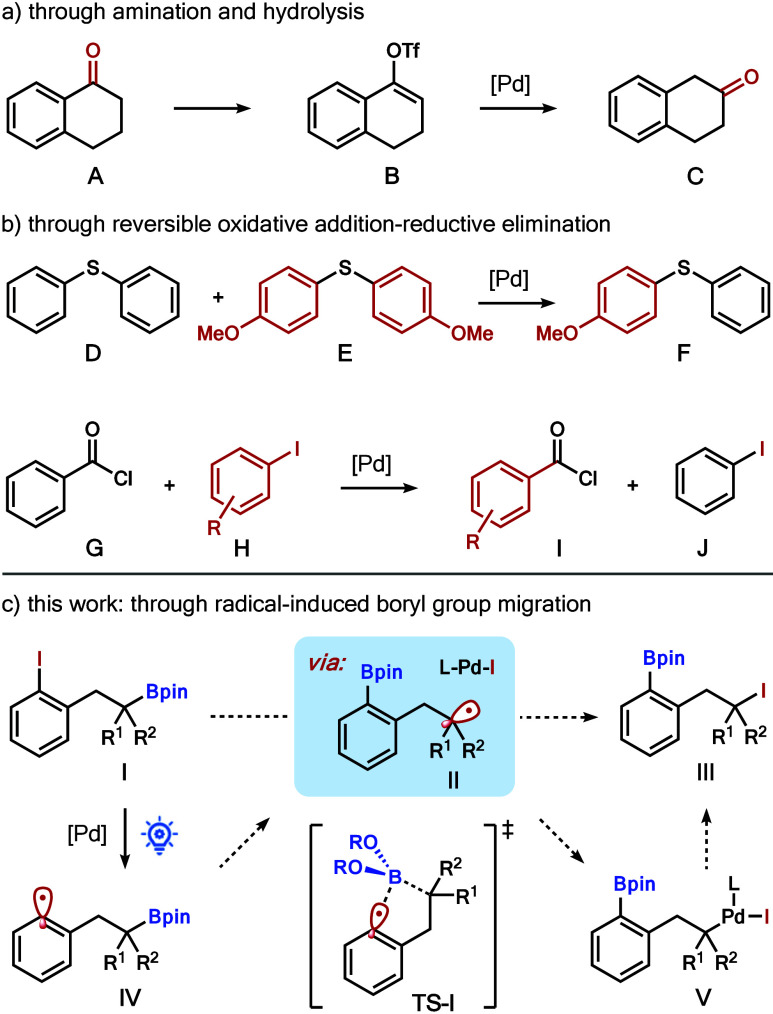
Functional Group Transposition Reactions

Along the line of our research interest in the development of novel catalytic methods with organoboron compounds,^8^ we questioned whether it would be possible to achieve intramolecular functional group transposition between a boryl group and an iodo group (e.g., from **I** to **III**). As shown in Scheme 1c, we anticipate that the reaction of iodoarene **I** appended with an alkylboronate group and a Pd(0) catalyst under appropriate conditions could produce aryl radical **IV** and the Pd­(I) complex L–Pd–I. The viability of this step is based on recent developments in excited-state Pd chemistry under photocatalytic conditions.^9^ We postulated that aryl radical **IV** could then undergo a 1,4-boryl migration via transition state **TS**-**1** to produce alkyl radical **II**, which should be thermodynamically more stable. However, the success of this step is predicated upon the feasibility of the key radical-induced 1,4-boryl migration. Inspired by the recent achievement of 1,2-boryl migration triggered by radicals,^10^ we felt that the five-membered transition state **TS**-**1**, resulting from the interaction of an ambiphilic aryl radical with the empty p orbital of the boron atom, seems reasonable. Moreover, the stability of alkyl radicals compared with aryl radicals serves as the thermodynamic driving force. Subsequent recombination of the Pd­(I) complex L–Pd–I with alkyl radical **II** should generate Pd­(II) complex **V**, which could undergo reductive elimination to generate arylboronate **III**.^11^ There are several attractive features of this transformation. First, it allows for the position switch of a boryl group and an iodo substituent, which constitutes a rare example of intramolecular functional group transposition. More importantly, this process inverts the polarity of two functional groups for subsequent transformations: from an electrophilic iodoarene in **I** to a nucleophilic arylboronate in **III** and from an alkylboronate in **I** that is often nucleophilic to the electrophilic alkyl iodide in **III**. Second, while there are ample examples to generate alkylboronates, methods for the synthesis of alkyl iodides are much less developed. This catalytic process would provide alkyl iodides, which are valuable intermediates for chemical synthesis. Lastly, this transformation is atom-economical, as the functional groups are completely preserved in the reaction product.^12^ However, there are also several potential challenges. First, the success of the 1,4-boryl migration is critically important. It will determine the fate of radical intermediate **IV**, which could undergo several competitive reaction pathways, such as hydrogen atom transfer (HAT). Moreover, in addition to generating alkyl iodide **III**, Pd­(II) complex **V** could also participate in other potential reaction pathways that lead to undesired side products. Herein we report successful implementation of this intramolecular functional group transposition. By using palladium and photo dual catalysis, this radical-induced process enables the transposition between a boryl group and an iodo group to convert iodoarenes **I** into arylboronates **III** (Scheme 1c).

To probe the feasibility of the proposed transformation, we prepared iodoarene **1a** appended with a tertiary boronic ester and evaluated the reaction parameters that could promote the Bpin and I group transposition. As summarized in Table 1, the reaction of **1a** was conducted with 10 mol % Pd­(OAc)_2_, 20 mol % DPEphos, and 2 equiv of K_3_PO_4_ in benzene at ambient temperature under blue LED irradiation. Under such conditions, tertiary alkyl iodide **2a** was isolated in 70% yield (entry 1). The reactions with other Pd catalysts, such as Pd­(dba)_2_ and Pd­(PPh_3_)_4_, gave inferior results (entries 2 and 3). A brief survey of ligands, including both monodentate and bidentate phosphine ligands, resulted in low yields of **2a** when DPEphos was replaced (the data with Xantphos or *rac*-BINAP as the ligand are shown in entries 4 and 5). The reaction with K_2_CO_3_ as the base gave **2a** in 41% yield (entry 6). When K_3_PO_4_ was substituted with the organic base Cy_2_NMe, **2a** was obtained in a comparable yield (entry 7). The reaction conducted in MeCN resulted in a poor yield of **2a** (entry 8). Control experiments revealed that the reaction requires the Pd catalyst, the ligand, and blue-light irradiation (entries 9, 10, and 12). The reaction can occur without the base, although the yield is poor (entry 11).

**Table 1:**
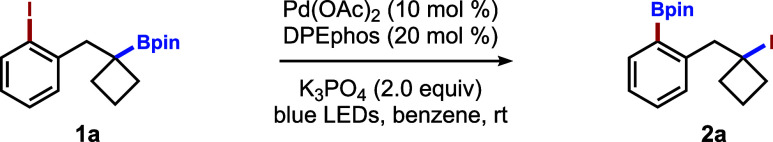
Evaluation of Reaction Parameters for the Bpin and I Group Transposition with Iodoarene **1a** ^a^

entry	deviation from the standard conditions	yield of **2a** (%)^b^
1	none	72 (70^c^)
2	Pd(dba)_2_ as the catalyst	16
3	Pd(PPh_3_)_4_ as the catalyst	17
4	Xantphos as the ligand	10
5	*rac*-BINAP as the ligand	24
6	K_2_CO_3_ as the base	41
7	Cy_2_NMe as the base	65
8	MeCN as the solvent	7
9	no Pd(OAc)_2_	NR^d^
10	no DPEphos	NR
11	no K_3_PO_4_	12
12	no blue LED irradiation	NR

a Reaction conditions: **1a** (0.1 mmol), Pd­(OAc)_2_ (10 mol %), DPEphos (20 mol %), K_3_PO_4_ (2.0 equiv), benzene (1.0 mL), blue LEDs (450 nm), rt, 3 h.

b The yields were determined by ^1^H NMR analysis of the crude reaction products using CH_2_Br_2_ as the internal standard.

c The yield of isolated product **2a** is listed in the parentheses.

d NR: no reaction.

After the optimal conditions were established, the reactions of a few iodoarene derivatives of **1a** were performed. As shown in Scheme 2, the reaction of substrate **1b** with a chlorine substituent on the arene gave tertiary alkyl iodide **2b** in 56% yield. The structure of **2b** was confirmed by X-ray crystallographic analysis. The reaction with iodoarene **1c** proceeded smoothly to afford alkyl iodide **2c** in 75% yield.

**Scheme 2:**
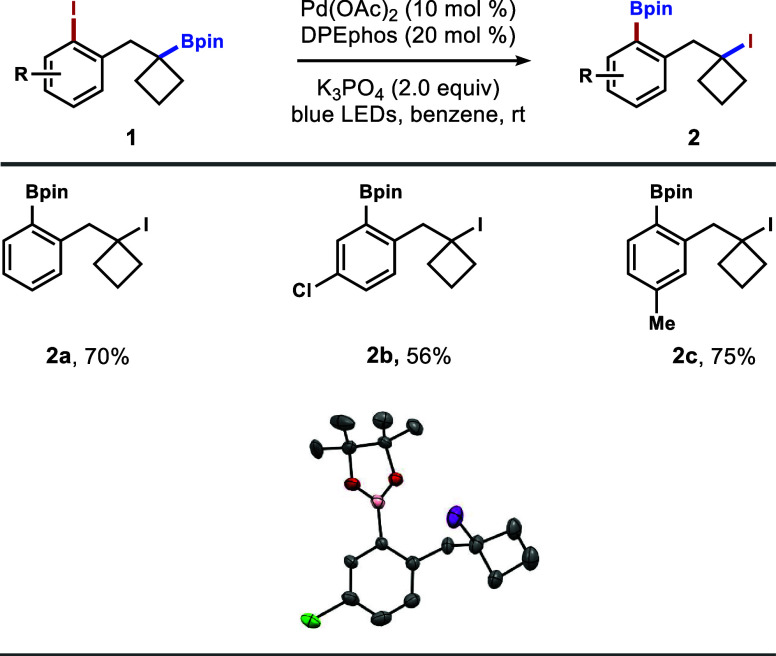
Scope of Iodoarenes with Tertiary Boronate^a^ ^,^ ^b^

a Reaction conditions: **1** (0.1 mmol), Pd­(OAc)_2_ (10 mol %), DPEphos (20 mol %), K_3_PO_4_ (2.0 equiv), benzene (1.0 mL), blue LEDs (450 nm), rt.
b The yields of isolated products **2** are listed.

Next, the reactions with iodoarenes **3** appended with a 1,1-bisborylalkane unit were investigated. By analogy to the proposed reaction pathway in Scheme 1, it was envisioned that the reaction with **3** should initially form an aryl radical (c.f., **IV** in Scheme 1), which triggers a 1,4-boryl group migration to generate a more stable secondary α-boryl radical intermediate. The empty p orbital of the boron atom could provide additional energetic benefit to further stabilize the secondary alkyl radical.^13^ As illustrated in Scheme 3, a variety of iodoarenes **3** participated in the reaction to give the desired α-iodoboronate products **4** (Cy_2_NMe or K_2_CO_3_ was used as the base). In general, the reaction tolerates the arene substitution at positions *meta* or *para* to the iodine group in **3**. A chlorine or bromine substituent is detrimental to the reaction, as products **4f** and **4g** were formed in low yields. In these cases, a mixture of side products derived from reduction (presumably via HAT from the reaction medium or the base) of the aryl radical intermediate (e.g., **IV** in Scheme 1) and aryl radical addition to the solvent was formed. The low yield of **4g** may also be attributed to competitive oxidative addition to the aryl bromide moiety, which could lead to other decomposition pathways of starting material **3g** or product **4g**. It is worth mentioning that α-iodoboronates are valuable intermediates in organic synthesis.^14,15^ This method constitutes a novel approach to generate α-iodoboronates.

**Scheme 3:**
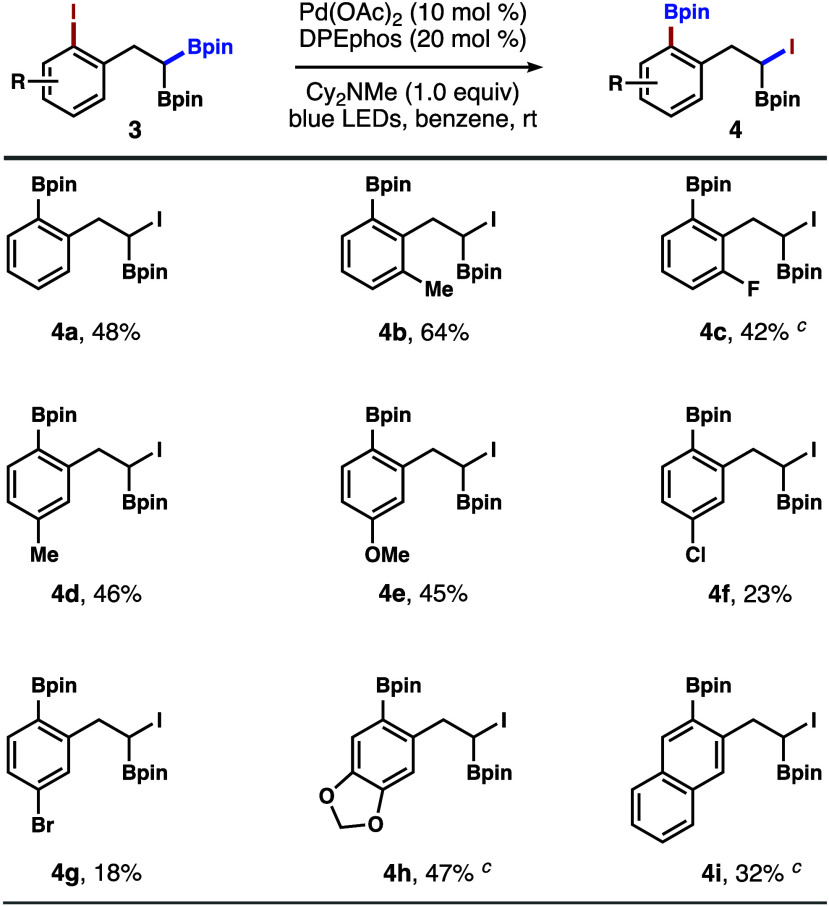
Scope of Iodoarenes with 1,1-Bisborylalkane^a^ ^,^ ^b^

a Reaction conditions: **3** (0.1 mmol), Pd­(OAc)_2_ (10 mol %), DPEphos (20 mol %), Cy_2_NMe (1.0 equiv), benzene (1.0 mL), blue LEDs (450 nm), rt.
b The yields of isolated products **4** are listed.
c The reactions were conducted with K_2_CO_3_ (1.0 equiv) as the base.

Scheme 4 summarizes the results from the reactions with iodoarenes **5** appended with a secondary alkylboronic ester. Overall, the reaction proceeded relatively well with a range of iodoarenes **5** to give secondary alkyl iodides **6**. Among the secondary alkylboronic esters **5** we explored, reactions with the methyl analogues are the most productive, affording **6a** and **6g**–**i** in synthetically useful yields. Lower efficiency was observed from the substrates with other alkyl groups, such as Et, ^*n*^Pr, ^*n*^Bu, homoallyl, and phenylethyl. In these cases, secondary alkyl iodides **6b**–**f** were obtained in lower yields.^16^

**Scheme 4:**
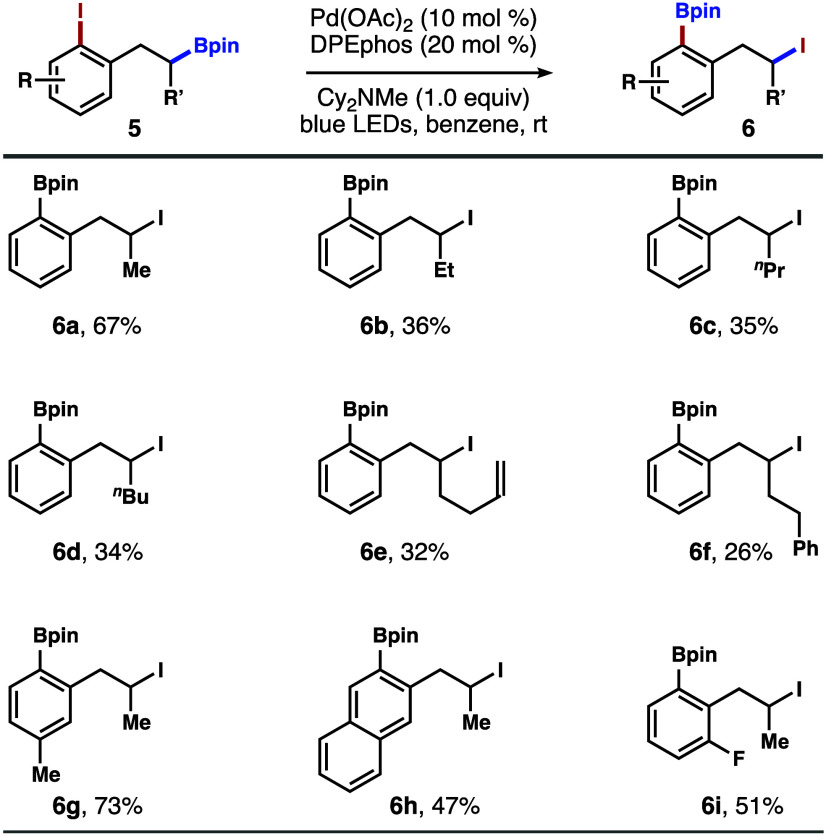
Scope of Secondary Boronic Esters^a^ ^,^ ^b^

a Reaction conditions: **5** (0.1 mmol), Pd­(OAc)_2_ (10 mol %), DPEphos (20 mol %), Cy_2_NMe (1.0 equiv), benzene (1.0 mL), blue LEDs (450 nm), rt.
b The yields of isolated products **6** are listed.

We conducted preliminary mechanistic investigations to gain insight into this novel dual catalytic functional group transposition reaction. As illustrated in Scheme 5, the reaction of **3a** was conducted under the standard conditions with the addition of 2 equiv of TEMPO. The lack of formation of product **4a** suggested the radical nature of the reaction process. Next, the reaction with secondary boronate **5j** with a cyclopropyl group was conducted. Under the standard conditions, ring-opening product **6j** was isolated as a 3:1 mixture of *E* and *Z* alkene isomers in a 40% yield. Additionally, the reaction with boronate **5k** generated the cyclization product **6k** as a 1:1 mixture of two diastereomers. The results indicate that the 1,4-boryl migration process in the reactions with **5j** and **5k** forms secondary alkyl radical intermediates, which undergo either cyclopropane ring-opening or 5-exo cyclization to give the corresponding products. Control experiments indicated that the Pd catalyst, ligand, and light are necessary for the reaction to proceed. The requirement of blue LED irradiation was further corroborated by the light on/off experiments in Scheme 5.

**Scheme 5:**
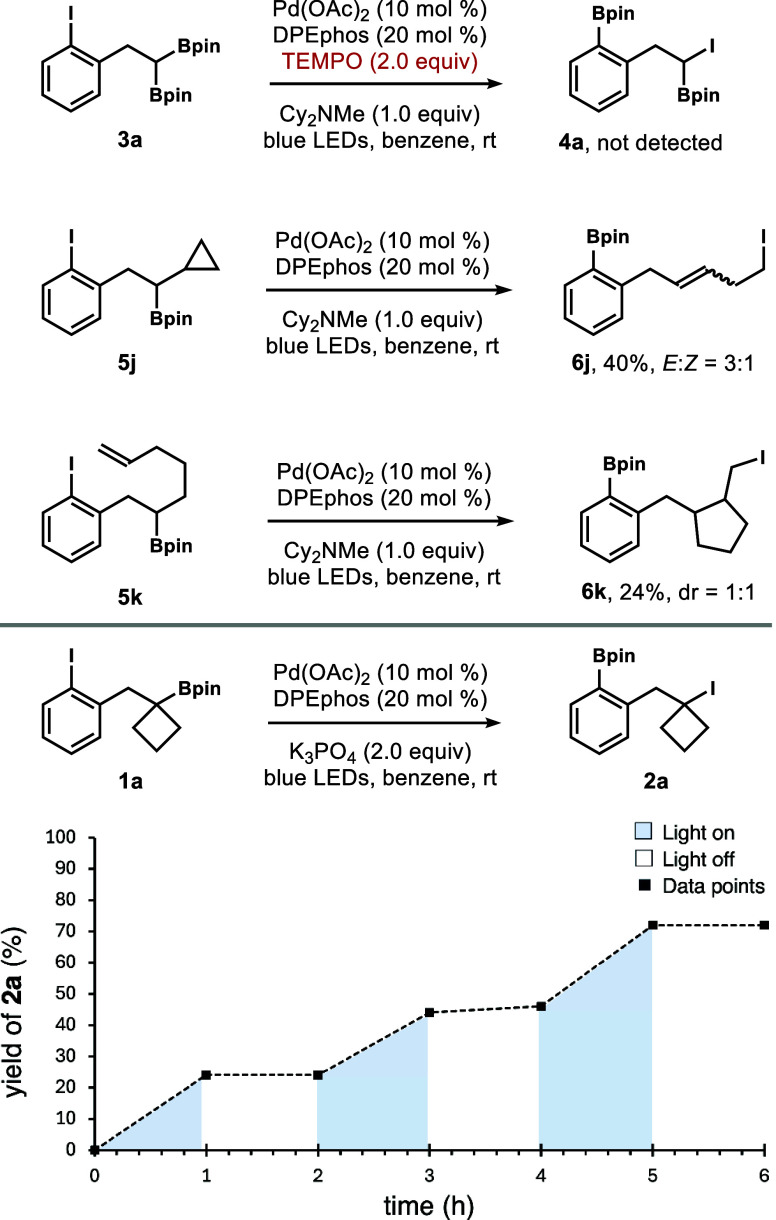
Preliminary Mechanistic Studies

The light on/off experiments in Scheme 5 suggested that the reaction is unlikely to be a chain reaction. To further support this conclusion, a crossover experiment between iodoarenes **5g** and **7** was performed. As shown in Scheme 6, the reaction of iodoarene **5g** under the standard conditions formed arylboronate **6g** in 73% yield. The reaction with iodoarene **7**, which has an ethylpinacol moiety, gave arylboronate **8** in 46% yield under identical conditions. If the transformation were a chain reaction, the formation of products **9** and **6a** would be expected. However, the crossover experiment between iodoarenes **7** and **5g** generated only arylboronates **8** and **6g** in 42% and 65% yield, respectively. Formation of the crossover products **9** and **6a** was not detected, supporting the notion that this functional group transposition occurred intramolecularly and is not a chain reaction.

**Scheme 6:**
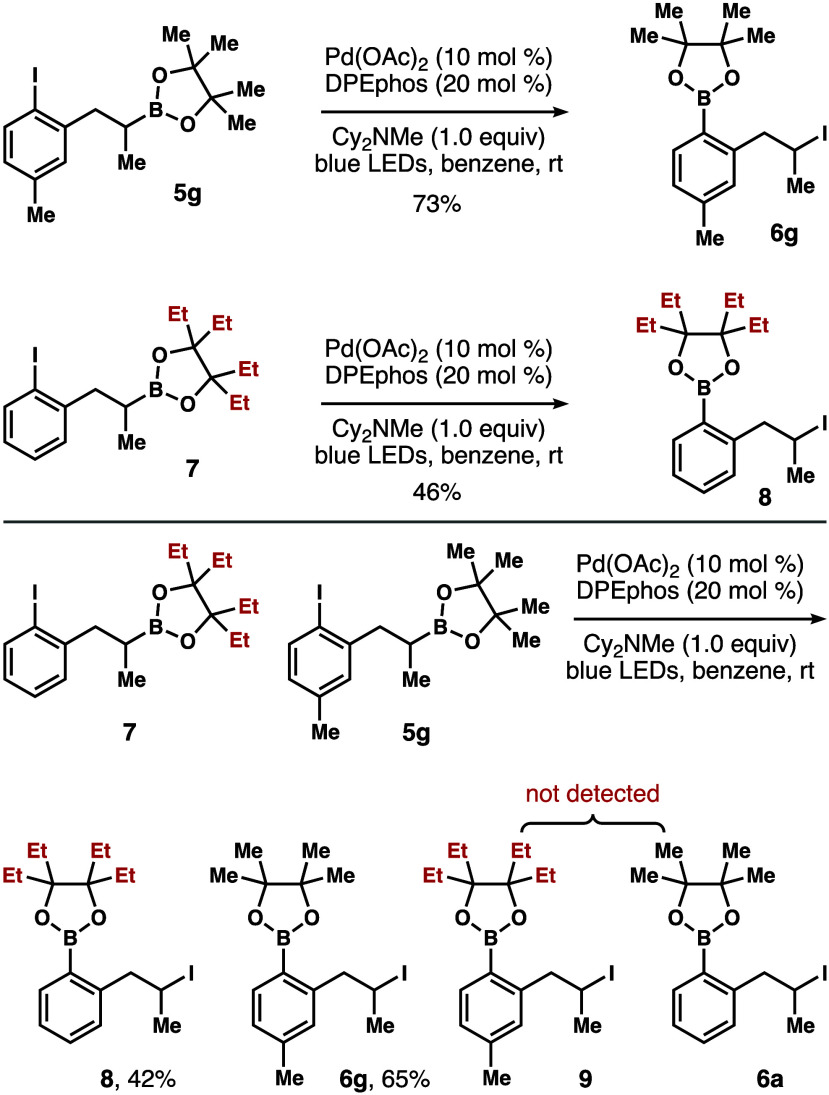
Crossover Experiment

As shown in Scheme 7, starting material **5a** contains an aryl iodide and a secondary boronate group, which could serve as an aryl electrophile and an alkyl nucleophile, respectively. For instance, the Pd-catalyzed coupling of **5a** with PhB­(OH)_2_ generated product **10** in 65% yield. On the other hand, the Bpin group of **5a** underwent amination to form product **11** in 53% yield. After the functional group transposition, however, product **6a** contains an arylboronate group and a secondary alkyl iodide, which reverses the polarity of the aryl and alkyl groups in **5a**. In the Suzuki coupling with PhI, the arylboronate of **6a** served as an aryl nucleophile to give product **12** in 53% yield. The secondary alkyl iodide in **6a** participated in a photocatalytic reaction to afford product **13** in 64% yield.

**Scheme 7:**
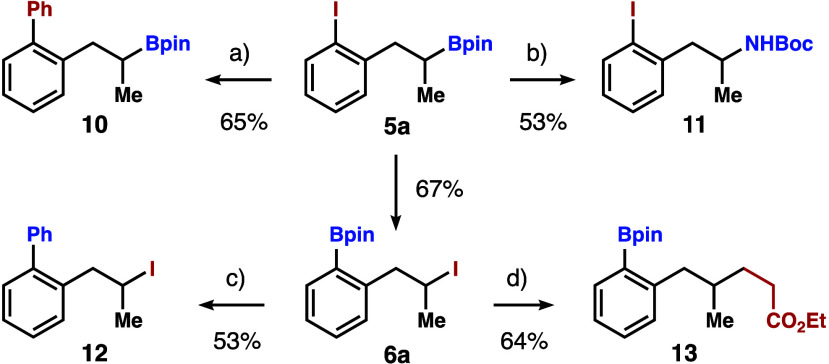
Derivatization Studies^a^

a Reagents and conditions: (a) Pd­(PPh_3_)_4_ (5 mol %), Cs_2_CO_3_ (1.5 equiv), PhB­(OH)_2_ (1.1 equiv), DMF, 100 °C. (b) MeONH_2_ **·**HCl (1.5 equiv), KO^*t*^Bu (3.0 equiv), toluene/THF (9:1), 80 °C, then Boc_2_O (1.5 equiv), THF, rt. (c) PdCl_2_(dppf)·DCM (5 mol %), PhI (1.5 equiv), K_2_CO_3_ (2.0 equiv), THF/H_2_O (4:1), 60 °C. (d) 4CzIPN (5 mol %), ethyl acrylate (3.0 equiv), NEt_3_ (2.0 equiv), MeCN/H_2_O (9:1), rt.

In summary, we developed a novel dual Pd and photocatalytic iodine and boryl group transposition reaction. Under the developed conditions, this radical-induced process enables the conversion of iodoarenes appended with an alkylboronate group into arylboronates bearing an alkyl iodide moiety, which inverts the polarities of the aryl and alkyl fragments of the starting iodoarenes. This method also offers a novel approach to generate alkyl iodides and α-iodoboronates. Preliminary mechanistic studies support that this transformation is (1) an intramolecular reaction and (2) radical-induced but unlikely to be a chain reaction. It should be noted that there are limitations to the reaction. Electron-deficient iodoarenes are poor substrates for the reaction. Besides the 1,4-boryl migration, the aryl radical intermediates generated from electron-deficient iodoarenes participate in other competitive reaction pathways that lead to a significant amount of side products, such as 1,5-HAT followed by Pd-catalyzed β-boryl elimination to give allylbenzene derivatives. When the 1,5-HAT pathway is not viable, the electron-poor aryl radical intermediate can abstract a hydrogen atom from the reaction medium or add to benzene (solvent). Additionally, the reactions with heteroaryl substrates have not been successful under the current conditions. Further investigations on reaction improvement and DFT computational studies on the reaction mechanism are currently underway.

## Supplementary Material



## Notes

Experimental procedures and spectra for all new compounds (PDF)
