# Supplementary material for: Functional Group Transposition Enabled by Palladium and Photo Dual Catalysis
Source: J Am Chem Soc. 2025 Oct 27;147(44):40058–63. doi: 10.1021/jacs.5c11429 (PMC12593406; doi:10.1021/jacs.5c11429)
Supplement: Supplementary file 1 [file ja5c11429_si_001.pdf]

Functional Group Transposition Enabled by Palladium and Photo Dual Catalysis

*Menghua Xu, Chengjun Wu, and Ming Chen**

Department of Chemistry, Virginia Tech

E-mail: mzc0102@vt.edu

Supporting Information: Experimental Procedures, Tabulated Spectroscopic Data, ^{1}H and ^{13}C Spectra of New Compounds

General Experimental Details. All reaction solvents were purified before use. Tetrahydrofuran, dichloromethane, diethyl ether and toluene were purified by passing through a solvent column composed of activated A-1 alumina. Unless indicated otherwise, all reactions were conducted under an atmosphere of argon using flame-dried or oven-dried (140 °C) glassware. 40 W Kessil 450 nm Blue LED lamps (Kessil A160WE Tuna Blue LED) were purchased from Kessil or Amazon. The term "concentrated under reduced pressure" refers to the removal of solvents and other volatile materials using a rotary evaporator with the water bath temperature below 30 °C, followed by removal of residual solvent at high vacuum (< 0.2 mbar).

Proton nuclear magnetic resonance (^1H NMR) spectra were acquired on commercial instruments (400, 500 and 600 MHz) at Virginia Tech NMR facility. Carbon-13 nuclear magnetic resonance (^{13}C NMR) spectra were acquired at 101, 126 and 151 MHz. The proton signal for residual non-deuterated solvent (δ 7.26 for $CHCl_3$ and δ 2.05 for acetone) was used as an internal reference for ^1H NMR spectra. For ^{13}C NMR spectra, chemical shifts are reported relative to the δ 77.36 resonance of $CHCl_3$ and the δ 29.84 resonance of acetone. Coupling constants are reported in Hz. High-resolution mass spectra were recorded on a commercial high-resolution mass spectrometer via the Micro Mass/Analytical Facility operated by the Department of Biochemistry, Virginia Tech.

Analytical thin layer chromatography (TLC) was performed on Kieselgel 60 F254 glass plates precoated with a 0.25 mm thickness of silica gel. The TLC plates were visualized with UV light and/or by staining with $KMnO_4$. Column chromatography was generally performed using Kieselgel 60 (230-400 mesh) silica gel, typically using a 50-100:1 weight ratio of silica gel to crude product.

Substrate Synthesis:

General Procedure A: Synthesis of Substrates ***3a-i***

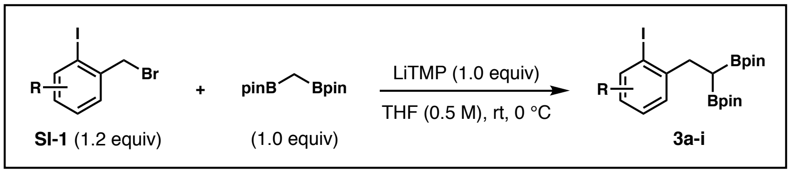

Under an argon atmosphere, to an oven-dried 25 mL round-bottom flask equipped with a magnetic stir bar was charged with TMP (2,2,6,6-tetramethylpiperidine, 593 mg, 4.2 mmol, 1.05 equiv) and THF (2.0 mL), followed by dropwise addition of ^{n}BuLi (1.6 M in hexanes, 2.5 mL, 4.0 mmol, 1.05 equiv) at 0 °C. After stirring at 0 °C for 15 minutes, a solution of 1,1-diborylethane (1.0 g, 4.0 mmol) in THF (2.0 mL) was added via syringe. After stirring at 0 °C for additional 15 minutes, a solution of compound **SI-1** (4.2 mmol) in THF (2.0 mL) was added. The reaction was kept stirring at 0 °C and the progress was monitored by TLC analyses. Upon completion of the reaction, a saturated aqueous solution of NH_4Cl (10 mL) was added to the reaction flask. The organic layer was separated, and the aqueous phase was extracted with ethyl acetate (3 × 15 mL). The combined organic extracts were washed with brine, dried over anhydrous Na_2SO_4, filtered, and concentrated under reduced pressure. The crude product was purified by flash column chromatography (gradient elution with hexanes and ethyl acetate) to give product **3a-i**.

General Procedure B: Synthesis of Substrates ***1a-c***

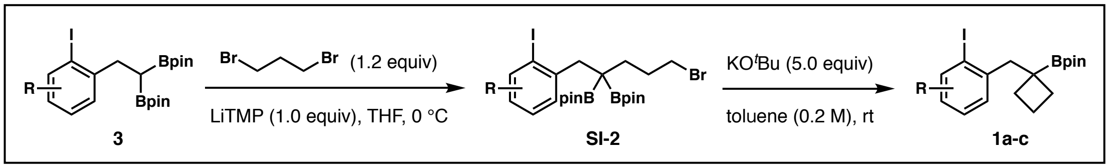

Step 1: Under an argon atmosphere, to an oven-dried 25 mL round-bottom flask equipped with a magnetic stir bar was charged with TMP (297 mg, 2.1 mmol, 1.05 equiv) and THF (1.0 mL), followed by the addition of ^{n}BuLi (2.5 M in hexanes, 0.80 mL, 2.0 mmol, 1.0 equiv) at 0 °C. After stirring for 15 min, a solution of diborylmethane **3** (2.0 mmol) in THF (2.0 mL) was added via a syringe, and the resulting mixture was allowed

to stir at 0 °C for 15 min. Then 1,3-bromoalkane (2.1 mmol) was added. The reaction was kept stirring at 0 °C and the progress was monitored by TLC analyses. Upon completion of the reaction, a saturated aqueous solution of NH_4Cl (5.0 mL) was added to the reaction flask. The organic layer was separated, and the aqueous phase was extracted with ethyl acetate (3 × 10 mL). The combined organic extracts were washed with brine, dried over anhydrous Na_2SO_4, filtered, and concentrated under reduced pressure. The crude product was purified by flash column chromatography (gradient elution with hexanes and ethyl acetate) to give product **SI-2**.

Step 2: In a glovebox, to an oven-dried 20 mL vial equipped with a magnetic stir bar was charged with **SI-2** (1.0 mmol, 1.0 equiv), toluene (5.0 mL, 0.2 M), and KO^{t}Bu (561 mg, 5.0 equiv). The vial was sealed with a polypropylene cap and removed from the glovebox. The resulting mixture was kept stirring at ambient temperature and the progress was monitored by TLC analyses. Upon completion of the reaction, diethyl ether (2.0 mL) was added to the reaction vial. The resulting mixture was filtered through a short pad of silica gel, and the silica pad was rinsed with additional diethyl ether (5.0 mL). The combined filtrate was concentrated under reduced pressure. The crude product was purified by flash column (gradient elution with hexanes and ethyl acetate) to give product **1a**–**c**.

*General Procedure C: Synthesis of Substrates **5a-i***

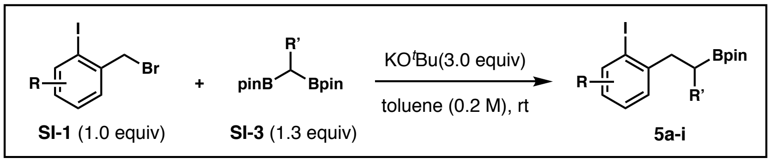

In a glovebox, to an oven-dried 20 mL reaction vial equipped with a magnetic stir bar was charged with **SI-1** (1.5 mmol, 1.0 equiv), **SI-3** (1.95 mmol, 1.3 equiv), toluene (7.5 mL), and KO^{t}Bu (505 mg, 4.5 mmol, 3.0 equiv). The reaction vial was sealed with a polypropylene cap and removed from the glovebox. The resulting mixture was kept stirring at ambient temperature and the progress was monitored by TLC analyses. Upon completion of the reaction, diethyl ether (2.0 mL) was added to the vial. The resulting mixture was filtered through a short pad of silica gel, and the silica pad was rinsed with additional diethyl ether (5.0 mL). The combined filtrate was concentrated under reduced pressure. The crude product was purified by flash column chromatography (gradient elution with hexanes and ethyl acetate) to give product **5a**–**i**.

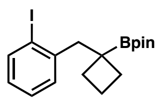

2-(1-(2-iodobenzyl)cyclobutyl)-4,4,5,5-tetramethyl-1,3,2-dioxaborolane (1a) Prepared according to the general procedure B. The crude mixture was purified by flash column chromatography to give compound **1a** as a white solid. ^{1}H NMR (500 MHz, $CDCl_3$) δ 7.81 (dd, *J* = 7.9, 1.3 Hz, 1H), 7.22 (ddd, *J* = 7.6, 7.4, 1.3 Hz, 1H), 7.06 (dd, *J* = 7.7, 1.7 Hz, 1H), 6.84 (ddd, *J* = 7.6, 7.5, 1.7 Hz, 1H), 2.98 (s, 2H), 2.23 – 2.28 (m, 2H), 1.86 – 2.02 (m, 4H), 1.19 (s, 12H). ^{13}C NMR (126 MHz, $CDCl_3$) δ 144.0, 139.6, 128.7, 128.2, 127.7, 103.0, 83.6, 49.6, 30.4, 24.9, 18.8. HRMS (ESI$^+$): m/z for $C_{17}H_{24}BIO_2Na$ $[M+Na]^+$ calcd. 421.0806, found: 421.0813.

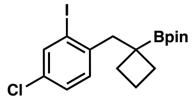

2-(1-(4-chloro-2-iodobenzyl)cyclobutyl)-4,4,5,5-tetramethyl-1,3,2-dioxaborolane (1b) Prepared according to the general procedure B. The crude mixture was purified by flash column chromatography to give compound **1b** as a white solid. ^{1}H NMR (500 MHz, $CDCl_3$) δ 7.81 (d, *J* = 2.2 Hz, 1H), 7.21 (dd, *J* = 8.3, 2.3 Hz, 1H), 6.98 (d, *J* = 8.3 Hz, 1H), 2.93 (s, 2H), 2.21 – 2.26 (m, 2H), 1.90 – 2.02 (m, 2H), 1.83 – 1.88 (m, 2H), 1.19 (s, 12H). ^{13}C NMR (126 MHz, $CDCl_3$) δ 142.7, 138.7, 132.0, 129.0, 128.2, 102.7, 83.7, 48.8, 30.3, 24.9, 18.8. HRMS (ESI$^+$): m/z for $C_{17}H_{24}BClIO_2$ $[M+H]^+$ calcd. 433.0597, found: 433.0596.

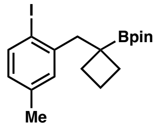

2-(1-(2-iodo-5-methylbenzyl)cyclobutyl)-4,4,5,5-tetramethyl-1,3,2-dioxaborolane (1c) Prepared according to the general procedure B. The crude mixture was purified by flash column chromatography to give compound **1c** as a white solid. ^{1}H NMR (500 MHz, $CDCl_3$) δ 7.67 (d, *J* = 8.0 Hz, 1H), 6.89 (d, *J* = 2.2 Hz, 1H), 6.67 (dd, *J* = 7.9, 2.2 Hz, 1H), 2.95 (s, 2H), 2.20 – 2.28 (m, 5H), 1.94 – 2.02 (m, 2H), 1.84 – 1.90 (m, 2H), 1.20 (s, 12H). ^{13}C NMR (126 MHz, $CDCl_3$) δ 143.7, 139.3, 138.0, 129.6, 128.6, 99.0, 83.6, 49.3, 30.3, 24.9, 21.4, 18.8. HRMS (ESI$^+$): m/z for $C_{18}H_{27}BIO_2$ $[M+H]^+$ calcd. 413.1143, found: 413.1128.

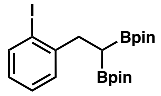

2,2'-(2-(2-iodophenyl)ethane-1,1-diyl)bis(4,4,5,5-tetramethyl-1,3,2-dioxaborolane) (3a) Prepared according to the general procedure A. The crude mixture was purified by flash column chromatography to give compound **3a** as a white solid. ^{1}H NMR (500 MHz, $CDCl_3$) δ 7.76 (dd, *J* = 7.9, 1.3 Hz, 1H), 7.34 (dd, *J* = 7.6, 1.7 Hz, 1H), 7.20 (ddd, *J* = 7.4, 7.4, 1.3 Hz, 1H), 6.81 (ddd, *J* = 7.6, 7.5, 1.7 Hz, 1H), 2.94 (d, *J* = 7.8 Hz, 2H), 1.26 (t, *J* = 7.9 Hz, 1H), 1.20 (s, 12H), 1.19 (s, 12H). ^{13}C NMR (126 MHz, $CDCl_3$) δ 147.1, 139.6, 129.8, 128.2, 127.7, 101.2, 83.5, 36.8, 25.2, 24.8. HRMS (ESI$^+$): m/z for $C_{20}H_{31}B_2IO_4Na$ $[M+Na]^+$ calcd. 507.1345, found: 507.1352.

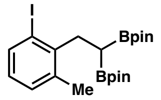

2,2'-(2-(2-iodo-6-methylphenyl)ethane-1,1-diyl)bis(4,4,5,5-tetramethyl-1,3,2-dioxaborolane) (3b) Prepared according to the general procedure A. The crude mixture was purified by flash column chromatography to give compound **3b** as a white solid. ^{1}H NMR (600 MHz, $CDCl_3$) δ 7.62 (dd, *J* = 8.0, 1.4 Hz, 1H), 7.03 (d, *J* = 7.5 Hz, 1H), 6.68 (dd, *J* = 7.7, 7.7 Hz, 1H), 3.06 (d, *J* = 7.1 Hz, 2H), 2.46 (s, 3H), 1.29 (t, *J* = 7.2 Hz, 1H), 1.20 (s, 12H), 1.19 (s, 12H). ^{13}C NMR (151 MHz, $CDCl_3$) δ 145.7, 138.0, 137.9, 130.6, 127.5, 102.4, 83.5, 32.7, 25.1, 25.0, 22.1. HRMS (ESI^+): m/z for $C_{21}H_{33}B_2IO_4Na$ $[M+Na]^+$ calcd. 521.1502, found: 521.1511.

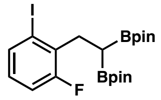

2,2'-(2-(2-fluoro-6-iodophenyl)ethane-1,1-diyl)bis(4,4,5,5-tetramethyl-1,3,2-dioxaborolane) (3c) Prepared according to the general procedure A. The crude mixture was purified by flash column chromatography to give compound **3c** as a white solid. ^{1}H NMR (500 MHz, $CDCl_3$) δ 7.54 (d, *J* = 7.8 Hz, 1H), 6.91 – 6.95 (m, 1H), 6.78 – 6.82 (m, 1H), 3.04 (dd, *J* = 7.4, 2.1 Hz, 2H), 1.19 – 1.23 (m, 25H). ^{13}C NMR (126 MHz, $CDCl_3$) δ 160.0 (d, *J* = 250.0 Hz), 135.3 (d, *J* = 3.6 Hz), 135.1 (d, *J* = 18.1 Hz), 128.7 (d, *J* = 8.7 Hz), 115.6 (d, *J* = 23.7 Hz), 101.6 (d, *J* = 3.8 Hz), 83.5, 29.4 (d, *J* = 2.2 Hz), 25.2, 24.7. ^{19}F NMR (565 MHz, $CDCl_3$) δ -108.6. HRMS (ESI^+): m/z for $C_{20}H_{30}B_2FIO_4Na$ $[M+Na]^+$ calcd. 525.1251, found: 525.1256.

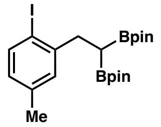

2,2'-(2-(2-iodo-5-methylphenyl)ethane-1,1-diyl)bis(4,4,5,5-tetramethyl-1,3,2-dioxaborolane) (3d) Prepared according to the general procedure A. The crude mixture was purified by flash column chromatography to give compound **3d** as a white solid. ^{1}H NMR (600 MHz, $CDCl_3$) δ 7.61 (d, *J* = 8.0 Hz, 1H), 7.17 (d, *J* = 2.2 Hz, 1H), 6.64 (dd, *J* = 8.0, 2.2 Hz, 1H), 2.90 (d, *J* = 7.8 Hz, 2H), 2.22 (s, 3H), 1.24 (t, *J* = 7.9 Hz, 1H), 1.20 (s, 24H). ^{13}C NMR (151 MHz, $CDCl_3$) δ 146.8, 139.3, 137.9, 130.8, 128.6, 96.9, 83.5, 36.6, 25.2, 24.8, 21.2. HRMS (ESI^+): m/z for $C_{21}H_{33}B_2IO_4Na$ $[M+Na]^+$ calcd. 521.1502, found: 521.1513.

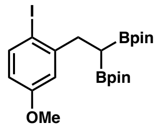

2,2'-(2-(2-iodo-5-methoxyphenyl)ethane-1,1-diyl)bis(4,4,5,5-tetramethyl-1,3,2-dioxaborolane) (3e) Prepared according to the general procedure A. The crude mixture was purified by flash column chromatography to give compound **3e** as a white solid. ^{1}H NMR (600 MHz, $CDCl_3$) δ 7.61 (d, *J* = 8.6 Hz, 1H), 6.94 (d, *J* = 3.0 Hz, 1H), 6.44 (dd, *J* = 8.7, 3.0 Hz, 1H), 3.75 (s, 3H), 2.90 (d, *J* = 7.7 Hz, 2H), 1.20 – 1.27 (m, 25H). ^{13}C NMR (126 MHz, $CDCl_3$) δ 160.0, 148.1, 139.9, 115.8, 113.8, 89.6, 83.5, 55.6, 36.8, 25.2, 24.8. HRMS (ESI^+): m/z for $C_{21}H_{33}B_2IO_5Na$ $[M+Na]^+$ calcd. 537.1451, found: 537.1458.

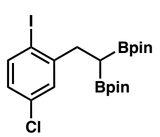

2,2'-(2-(5-chloro-2-iodophenyl)ethane-1,1-diyl)bis(4,4,5,5-tetramethyl-1,3,2-dioxaborolane) (3f) Prepared according to the general procedure A. The crude mixture was purified by flash column chromatography to give compound **3f** as a white solid. ^{1}H NMR (400 MHz, $CDCl_3$) δ 7.65 (d, *J* = 8.4 Hz, 1H), 7.36 (d, *J* = 2.6 Hz, 1H), 6.81 (dd, *J* = 8.4, 2.6 Hz, 1H), 2.90 (d, *J* = 7.9 Hz, 2H), 1.18 – 1.23 (m, 25H). ^{13}C NMR (101 MHz, $CDCl_3$) δ 149.0, 140.5, 134.3, 130.0, 127.7, 97.9, 83.6, 36.7, 25.2, 24.8. HRMS (ESI$^+$): m/z for $C_{20}H_{30}B_2ClIO_4Na$ $[M+Na]^+$ calcd. 541.0956, found: 541.0955.

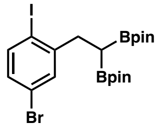

2,2'-(2-(5-bromo-2-iodophenyl)ethane-1,1-diyl)bis(4,4,5,5-tetramethyl-1,3,2-dioxaborolane) (3g) Prepared according to the general procedure A. The crude mixture was purified by flash column chromatography to give compound **3g** as a white solid. ^{1}H NMR (600 MHz, $CDCl_3$) δ 7.58 (d, *J* = 8.3 Hz, 1H), 7.51 (d, *J* = 2.4 Hz, 1H), 6.95 (dd, *J* = 8.4, 2.4 Hz, 1H), 2.89 (d, *J* = 7.9 Hz, 2H), 1.17 – 1.24 (m, 25H). ^{13}C NMR (151 MHz, $CDCl_3$) δ 149.3, 140.8, 132.8, 130.7, 122.4, 98.8, 83.6, 36.7, 25.2, 24.8, 11.6. HRMS (ESI$^+$): m/z for $C_{20}H_{30}B_2BrIO_4Na$ $[M+Na]^+$ calcd. 563.0631, found: 563.0638.

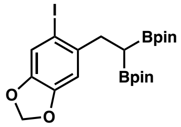

2,2'-(2-(6-iodobenzo[*d*][1,3]dioxol-5-yl)ethane-1,1-diyl)bis(4,4,5,5-tetramethyl-1,3,2-dioxaborolane) (3h) Prepared according to the general procedure A. The crude mixture was purified by column chromatography to give compound **3h** as a white solid. ^{1}H NMR (600 MHz, $CDCl_3$) δ 7.18 (d, *J* = 1.2 Hz, 1H), 6.91 (d, *J* = 1.3 Hz, 1H), 5.89 (s, 2H), 2.86 (d, *J* = 7.7 Hz, 2H), 1.20 (s, 24H), 1.16 (t, *J* = 7.8 Hz, 1H). ^{13}C NMR (151 MHz, $CDCl_3$) δ 148.2, 146.5, 140.6, 118.7, 109.9, 101.5, 88.0, 83.5, 36.6, 25.2, 24.7, 12.1. HRMS (ESI$^+$): m/z for $C_{21}H_{31}B_2IO_6Na$ $[M+Na]^+$ calcd. 551.1244, found: 551.1260.

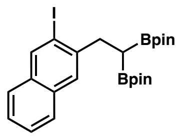

2,2'-(2-(3-iodonaphthalen-2-yl)ethane-1,1-diyl)bis(4,4,5,5-tetramethyl-1,3,2-dioxaborolane) (3i) Prepared according to the general procedure A. The crude mixture was purified by flash column chromatography to give compound **3h** as a white solid. ^{1}H NMR (600 MHz, $CDCl_3$) δ 8.34 (s, 1H), 7.78 (s, 1H), 7.65 – 7.69 (m, 2H), 7.38 – 7.44 (m, 2H), 3.10 (d, *J* = 7.8 Hz, 2H), 1.37 (t, *J* = 7.8 Hz, 1H), 1.20 (s, 24H). ^{13}C NMR (151 MHz, $CDCl_3$) δ 143.4, 138.8, 133.7, 133.3, 127.6, 127.3, 126.7, 126.6, 126.0, 99.7, 83.6, 36.7, 25.3, 24.8. HRMS (ESI$^+$): m/z for $C_{24}H_{33}B_2IO_4Na$ $[M+Na]^+$ calcd. 557.1502, found: 557.1503.

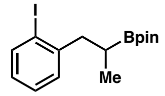

2-(1-(2-iodophenyl)propan-2-yl)-4,4,5,5-tetramethyl-1,3,2-dioxaborolane (5a) Prepared according to the general procedure C. The crude mixture was purified by flash column to give compound **5a** as colorless oil. ^{1}H NMR (500 MHz, $CDCl_3$) δ 7.79 (dd, *J* = 7.8, 1.2 Hz, 1H), 7.20 – 7.24 (m, 2H), 6.84 (ddd, *J* = 7.8, 6.9, 2.1 Hz, 1H), 2.92 (dd, *J* = 13.8, 8.0 Hz, 1H), 2.61 (dd, *J* = 13.8, 7.8 Hz, 1H), 1.44 – 1.51 (m, 1H), 1.21 (s, 6H), 1.20 (s, 6H), 1.01 (d, *J* = 7.4 Hz, 3H). ^{13}C NMR (126 MHz, $CDCl_3$) δ 145.2, 139.8, 130.3, 128.1, 127.8, 101.5, 83.4, 43.9, 25.1, 25.0, 15.7. HRMS (ESI$^+$): m/z for $C_{15}H_{22}BIO_2Na$ $[M+Na]^+$ calcd. 395.0650, found: 395.0633.

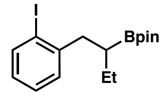

2-(1-(2-iodophenyl)butan-2-yl)-4,4,5,5-tetramethyl-1,3,2-dioxaborolane (5b) Prepared according to the general procedure C. The crude mixture was purified by flash column chromatography to give compound **5b** as colorless oil. ^{1}H NMR (500 MHz, $CDCl_3$) δ 7.78 (dd, *J* = 7.8, 1.2 Hz, 1H), 7.26 – 7.28 (m, 1H), 7.20 – 7.23 (m, 1H), 6.82 – 6.85 (m, 1H), 2.84 (dd, *J* = 13.8, 9.0 Hz, 1H), 2.72 (dd, *J* = 13.8, 6.8 Hz, 1H), 1.47 – 1.53 (m, 2H), 1.38 – 1.44 (m, 1H), 1.20 (s, 6H), 1.17 (s, 6H), 0.97 (t, *J* = 7.4 Hz, 3H). ^{13}C NMR (126 MHz, $CDCl_3$) δ 145.3, 139.8, 130.3, 128.1, 127.8, 101.5, 83.4, 42.1, 25.2, 25.1, 24.6, 13.9. HRMS (ESI$^+$): m/z for $C_{16}H_{25}BIO_2$ $[M+H]^+$ calcd. 387.0987, found: 387.0975.

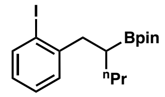

2-(1-(2-iodophenyl)pentan-2-yl)-4,4,5,5-tetramethyl-1,3,2-dioxaborolane (5c) Prepared according to the general procedure C. The crude mixture was purified by flash column chromatography to give compound **5c** as colorless oil. ^{1}H NMR (500 MHz, $CDCl_3$) δ 7.78 (dd, *J* = 7.9, 1.3 Hz, 1H), 7.27 (dd, *J* = 7.7, 1.9 Hz, 1H), 7.20 – 7.23 (m, 1H), 6.82 – 6.85 (m, 1H), 2.83 (dd, *J* = 13.8, 8.6 Hz, 1H), 2.72 (dd, *J* = 13.8, 5.9 Hz, 1H), 1.32 – 1.51 (m, 5H), 1.19 (s, 6H), 1.16 (s, 6H), 0.91 (t, *J* = 7.1 Hz, 3H). ^{13}C NMR (126 MHz, $CDCl_3$) δ 145.3, 139.8, 130.3, 128.1, 127.8, 101.5, 83.4, 42.5, 34.1, 25.2, 25.1, 22.6, 14.8. HRMS (ESI$^+$): m/z for $C_{17}H_{27}BIO_2$ $[M+H]^+$ calcd. 401.1143, found: 401.1162.

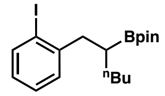

2-(1-(2-iodophenyl)hexan-2-yl)-4,4,5,5-tetramethyl-1,3,2-dioxaborolane (5d) Prepared according to the general procedure C. The crude mixture was purified by flash column chromatography to give compound **5d** as colorless oil. ^{1}H NMR (500 MHz, $CDCl_3$) δ 7.81 (dd, *J* = 7.8, 1.3 Hz, 1H), 7.30 (dd, *J* = 7.7, 1.9 Hz, 1H), 7.22 – 7.25 (m, 1H), 6.84 – 6.88 (m, 1H), 2.86 (dd, *J* = 13.7, 8.7 Hz, 1H), 2.75 (dd, *J* = 13.7, 5.7 Hz, 1H), 1.44 – 1.54 (m, 3H), 1.32 – 1.41 (m, 4H), 1.22 (s, 6H), 1.18 (s, 6H), 0.91 (t, *J* = 6.9 Hz, 3H). ^{13}C NMR (126 MHz, $CDCl_3$) δ 145.3, 139.7, 130.2, 128.1,

127.8, 101.5, 83.4, 42.5, 31.6, 31.5, 25.15, 25.09, 23.3, 14.4. HRMS (ESI$^+$): m/z for $C_{18}H_{29}BIO_2$ [M+H]$^+$ calcd. 415.1300, found: 415.1299.

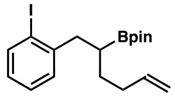

2-(1-(2-iodophenyl)hex-5-en-2-yl)-4,4,5,5-tetramethyl-1,3,2-dioxaborolane (5e) Prepared according to the general procedure C. The crude mixture was purified to give compound **5e** as colorless oil. ^{1}H NMR (400 MHz, $CDCl_3$) δ 7.78 (d, *J* = 7.9 Hz, 1H), 7.27 (d, *J* = 7.5 Hz, 1H), 7.20 – 7.23 (m, 1H), 6.82 – 6.86 (m, 1H), 5.77 – 5.87 (m, 1H), 5.00 (dd, *J* = 17.1, 2.1 Hz, 1H), 4.93 (d, *J* = 10.2 Hz, 1H), 2.86 (dd, *J* = 13.8, 8.7 Hz, 1H), 2.73 (dd, *J* = 13.8, 6.3 Hz, 1H), 2.04 – 2.19 (m, 2H), 1.44 – 1.64 (m, 3H), 1.20 (s, 6H), 1.16 (s, 6H). ^{13}C NMR (101 MHz, $CDCl_3$) δ 145.1, 139.8, 139.3, 130.3, 128.2, 127.9, 114.8, 101.5, 83.5, 42.4, 33.6, 31.0, 25.2, 25.1. HRMS (ESI$^+$): m/z for $C_{18}H_{26}BIO_2Na$ [M+Na]$^+$ calcd. 435.0963, found: 435.0957.

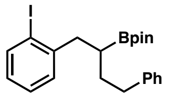

2-(1-(2-iodophenyl)-4-phenylbutan-2-yl)-4,4,5,5-tetramethyl-1,3,2-dioxaborolane (5f) Prepared according to the general procedure C. The crude mixture was purified by flash column chromatography to give compound **5f** as colorless oil. ^{1}H NMR (400 MHz, $CDCl_3$) δ 7.79 (dd, *J* = 7.9, 1.2 Hz, 1H), 7.26 – 7.29 (m, 2H), 7.15 – 7.25 (m, 5H), 6.83 – 6.87 (m, 1H), 2.91 (dd, *J* = 13.8, 9.2 Hz, 1H), 2.79 (dd, *J* = 13.8, 6.8 Hz, 1H), 2.60 – 2.74 (m, 2H), 1.70 – 1.88 (m, 2H), 1.54 – 1.61 (m, 1H), 1.23 (s, 6H), 1.19 (s, 6H). ^{13}C NMR (101 MHz, $CDCl_3$) δ 145.0, 143.1, 139.8, 130.3, 128.7, 128.6, 128.2, 127.9, 126.0, 101.5, 83.5, 42.3, 35.8, 33.7, 25.2, 25.1. HRMS (ESI$^+$): m/z for $C_{22}H_{29}BIO_2$ [M+H]$^+$ calcd. 463.1300, found: 463.1303.

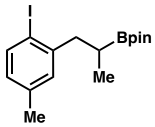

2-(1-(2-iodo-5-methylphenyl)propan-2-yl)-4,4,5,5-tetramethyl-1,3,2-dioxaborolane (5g) Prepared according to the general procedure C. The crude mixture was purified by flash column chromatography to give compound **5g** as colorless oil. ^{1}H NMR (500 MHz, $CDCl_3$) δ 7.64 (d, *J* = 8.0 Hz, 1H), 7.08 (d, *J* = 2.2 Hz, 1H), 6.67 (dd, *J* = 8.0, 2.2 Hz, 1H), 2.86 (dd, *J* = 13.7, 8.4 Hz, 1H), 2.58 (dd, *J* = 13.7, 7.4 Hz, 1H), 2.25 (s, 3H), 1.42 – 1.50 (m, 1H), 1.22 (s, 6H), 1.20 (s, 6H), 1.02 (d, *J* = 7.4 Hz, 3H). ^{13}C NMR (126 MHz, $CDCl_3$) δ 144.9, 139.4, 137.9, 131.2, 128.8, 97.4, 83.4, 43.8, 25.1, 25.0, 21.2, 15.8. HRMS (ESI$^+$): m/z for $C_{16}H_{25}BIO_2$ [M+H]$^+$ calcd. 387.0987, found: 387.0976.

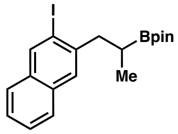

2-(1-(3-iodonaphthalen-2-yl)propan-2-yl)-4,4,5,5-tetramethyl-1,3,2-dioxaborolane (5h) Prepared according to the general procedure C. The crude mixture was purified by flash column chromatography to give

compound **5h** as colorless oil. ^{1}H NMR (500 MHz, $CDCl_3$) δ 8.36 (s, 1H), 7.67 – 7.71 (m, 3H), 7.39 – 7.46 (m, 2H), 3.07 (dd, *J* = 14.0, 8.6 Hz, 1H), 2.75 (dd, *J* = 14.1, 7.1 Hz, 1H), 1.55 – 1.61 (m, 1H), 1.20 (s, 6H), 1.18 (s, 6H), 1.07 (d, *J* = 7.4 Hz, 3H). ^{13}C NMR (126 MHz, $CDCl_3$) δ 141.5, 138.9, 133.8, 133.2, 127.9, 127.6, 126.7 (two overlapping carbon signals), 126.1, 99.9, 83.4, 44.0, 25.2, 25.0, 15.8. HRMS (ESI^+): m/z for $C_{19}H_{25}BIO_2$ $[M+H]^+$ calcd. 423.0987, found: 423.0978.

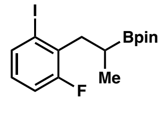

2-(1-(2-fluoro-6-iodophenyl)propan-2-yl)-4,4,5,5-tetramethyl-1,3,2-dioxaborolane (5i) Prepared according to the general procedure C. The crude mixture was purified by flash column chromatography to give compound **5i** as colorless oil. ^{1}H NMR (500 MHz, $CDCl_3$) δ 7.58 – 7.61 (m, 1H), 6.96 – 7.00 (m, 1H), 6.82 – 6.87 (m, 1H), 2.95 (ddd, *J* = 13.6, 6.3, 2.6 Hz, 1H), 2.74 (ddd, *J* = 13.6, 9.7, 2.3 Hz, 1H), 1.44 – 1.52 (m, 1H), 1.24 (s, 6H), 1.23 (s, 6H), 0.97 (dd, *J* = 7.5, 1.2 Hz, 3H). ^{13}C NMR (126 MHz, $CDCl_3$) δ 160.5 (d, *J* = 249.1 Hz), 135.5 (d, *J* = 3.3 Hz), 133.1 (d, *J* = 17.9 Hz), 128.9 (d, *J* = 8.8 Hz), 115.6 (d, *J* = 23.9 Hz), 101.9 (d, *J* = 4.1 Hz), 83.4, 36.2, 25.10, 25.08, 15.1. ^{19}F NMR (376 MHz, $CDCl_3$) δ -109.4. HRMS (ESI^+): m/z for $C_{19}H_{25}BIO_2$ $[M+H]^+$ calcd. 423.0987, found: 423.0978.

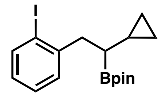

2-(1-cyclopropyl-2-(2-iodophenyl)ethyl)-4,4,5,5-tetramethyl-1,3,2-dioxaborolane (5j) Prepared according to the general procedure C. The crude mixture was purified by flash column chromatography to give compound **5j** as colorless oil. ^{1}H NMR (600 MHz, $CDCl_3$) δ 7.76 (dd, *J* = 7.9, 1.3 Hz, 1H), 7.29 (dd, *J* = 7.6, 1.7 Hz, 1H), 7.18 – 7.21 (m, 1H), 6.81 – 6.85 (m, 1H), 2.87 – 2.96 (m, 2H), 1.20 (s, 6H), 1.17 (s, 6H), 0.83 – 0.87 (m, 1H), 0.74 – 0.80 (m, 1H), 0.39 – 0.44 (m, 2H), 0.16 – 0.18 (m, 1H), 0.10 – 0.13 (m, 1H). ^{13}C NMR (126 MHz, $CDCl_3$) δ 145.1, 139.7, 130.3, 128.1, 127.8, 101.4, 83.5, 42.9, 25.1, 25.0, 13.0, 5.7, 3.9.

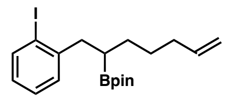

2-(1-(2-iodophenyl)hept-6-en-2-yl)-4,4,5,5-tetramethyl-1,3,2-dioxaborolane (5k) Prepared according to the general procedure C. The crude mixture was purified by column chromatography to give compound **5k** as colorless oil. ^{1}H NMR (600 MHz, $CDCl_3$) δ 7.78 (dd, *J* = 7.9, 1.3 Hz, 1H), 7.26 – 7.28 (m, 1H), 7.20 – 7.23 (m, 1H), 6.82 – 6.85 (m, 1H), 5.78 – 5.83 (m, 1H), 4.97 – 5.01 (m, 1H), 4.91 – 4.92 (m, 1H), 2.84 (dd, *J* = 13.7, 8.6 Hz, 1H), 2.72 (dd, *J* = 13.8, 5.7 Hz, 1H), 2.01 – 2.10 (m, 2H), 1.41 – 1.52 (m, 5H), 1.19 (s, 6H), 1.16 (s, 6H). ^{13}C NMR (151 MHz, $CDCl_3$) δ 145.2, 139.8, 139.3, 130.3, 128.1, 127.9, 114.6, 101.5, 83.4, 42.5, 34.3, 31.3, 28.7, 25.2, 25.1.

Procedure for Palladium and Photo Dual Catalytic Functional Group Transposition

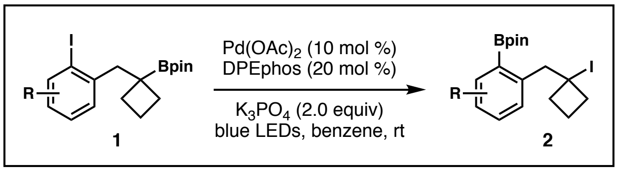

General Procedure D: In a glove box, to an oven-dried 10 mL reaction tube equipped with a magnetic stir bar was charged with iodoarene **1** (0.1 mmol, 1.0 equiv), $Pd(OAc)_2$ (2 mg, 0.01 mmol, 10 mol %), DPEphos (11 mg, 0.02 mmol, 20 mol %), and K_3PO_4 (43 mg, 0.2 mmol, 2.0 equiv) under a nitrogen atmosphere. Degassed anhydrous benzene (1.0 mL) was added, and the reaction tube was sealed with a rubber septum. The reaction tube was irradiated with 40 W Kessil 450 nm blue LED lamps (Kessil A160WE Tuna Blue LED) with cooling provided by a fan (temperature reached 37 °C). The distance from the lamp to the reaction tube is about 3 cm. The reaction progress was monitored by ^1H NMR analysis. Upon completion of the reaction, the reaction mixture was diluted with ethyl acetate (2 mL) and filtered through Celite. The filtrate was concentrated under reduced pressure. The crude residue was purified by flash column chromatography (gradient elution with hexanes and ethyl acetate) to afford product **2**.

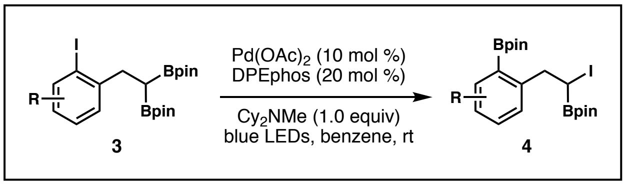

General Procedure E: In a glove box, to an oven-dried 10 mL reaction tube equipped with a magnetic stir bar was charged with iodoarene **3** (0.1 mmol, 1.0 equiv), $Pd(OAc)_2$ (2 mg, 0.01 mmol, 10 mol %), DPEphos (11 mg, 0.02 mmol, 20 mol %), and Cy_2NMe (20 mg, 0.1 mmol, 1.0 equiv) under a nitrogen atmosphere. Degassed anhydrous benzene (1.0 mL) was added, and the reaction tube was sealed with a rubber septum. The reaction tube was irradiated with 40 W Kessil 450 nm blue LED lamps (Kessil A160WE Tuna Blue LED) with cooling provided by a fan (temperature reached 37 °C). The distance from the lamp to the reaction tube is about 3 cm. The reaction progress was monitored by ^1H NMR analysis. Upon completion of the reaction, the reaction mixture was diluted with ethyl acetate (2.0 mL) and filtered through Celite. The filtrate was concentrated under reduced pressure. The crude residue was purified by flash column chromatography (gradient elution with hexanes and ethyl acetate) to afford product **4**.

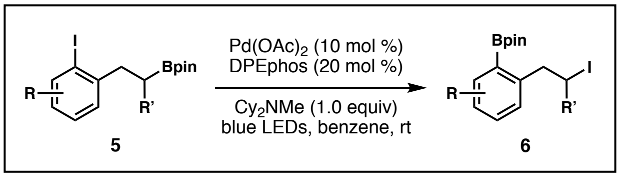

General Procedure F: In a glove box, to an oven-dried 10 mL reaction tube equipped with a magnetic stir bar was charged with iodoarene **5** (0.1 mmol, 1.0 equiv), $Pd(OAc)_2$ (2 mg, 0.01 mmol, 10 mol %), DPEphos (11 mg, 0.02 mmol, 20 mol %), and Cy_2NMe (20 mg, 0.1 mmol, 1.0 equiv) under a nitrogen atmosphere. Degassed anhydrous benzene (1.0 mL) was added, and the reaction tube was sealed with a rubber septum. The reaction tube was irradiated with 40 W Kessil 450 nm blue LED lamps (Kessil A160WE Tuna Blue LED) with cooling provided by a fan (temperature reached 37 °C). The distance from the lamp to the reaction tube is about 3 cm. The reaction progress was monitored by ^{1}H NMR analysis. Upon completion of the reaction, the reaction mixture was diluted with ethyl acetate (2.0 mL) and filtered through Celite. The filtrate was concentrated under reduced pressure. The crude residue was purified by flash column chromatography (gradient elution with hexanes and ethyl acetate) to afford product **6**.

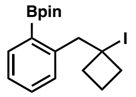

2-(2-((1-iodocyclobutyl)methyl)phenyl)-4,4,5,5-tetramethyl-1,3,2-dioxaborolane (2a) Prepared according to the general procedure D. The crude mixture was purified by flash column chromatography to give compound **2a** as colorless oil in 70% yield (28 mg). ^{1}H NMR (600 MHz, Acetone-d_6) δ 7.81 (dd, J = 7.5, 1.6 Hz, 1H), 7.43 (ddd, J = 7.5, 7.4, 1.5 Hz, 1H), 7.39 (dd, J = 7.6, 1.1 Hz, 1H), 7.29 (ddd, J = 7.4, 7.3, 1.3 Hz, 1H), 3.73 (s, 2H), 2.44 – 2.53 (m, 4H), 2.23 – 2.30 (m, 1H), 1.64 – 1.70 (m, 1H), 1.36 (s, 12H). ^{13}C NMR (151 MHz, Acetone-d_6) δ 145.4, 136.8, 131.6, 131.4, 126.8, 84.4, 53.4, 51.5, 41.9, 25.3, 19.1. HRMS (ESI$^+$): m/z for $C_{17}H_{25}BIO_2$ $[M+H]^+$ calcd. 399.0987, found: 399.0977.

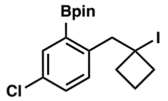

2-(5-chloro-2-((1-iodocyclobutyl)methyl)phenyl)-4,4,5,5-tetramethyl-1,3,2-dioxaborolane (2b) Prepared according to the general procedure D. The crude mixture was purified to give compound **2b** as a yellow solid in 56% yield (24 mg). ^{1}H NMR (600 MHz, $CDCl_3$) δ 7.80 (d, J = 2.4 Hz, 1H), 7.36 (dd, J = 8.3, 2.4 Hz, 1H), 7.26 (d, J = 0.8 Hz, 1H), 3.65 (s, 2H), 2.52 – 2.56 (m, 2H), 2.36 – 2.41 (m, 2H), 2.26 – 2.34 (m, 1H), 1.67 – 1.73 (m, 1H), 1.35 (s, 12H). ^{13}C NMR (126 MHz, Acetone-d_6) δ 144.3, 136.0, 133.4, 132.6, 131.2, 85.0, 52.8, 50.4, 42.1, 25.2, 19.1. HRMS (ESI$^+$): m/z for $C_{17}H_{23}BClIO_2Na$ $[M+Na]^+$ calcd. 455.0417, found: 455.0427.

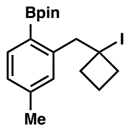

2-(2-((1-iodocyclobutyl)methyl)-4-methylphenyl)-4,4,5,5-tetramethyl-1,3,2-dioxaborolane (2c) Prepared according to the general procedure D. The crude mixture was purified by flash column chromatography to give compound **2c** as colorless oil in 75% yield (31 mg). ^{1}H NMR (600 MHz, $CDCl_3$) δ 7.74 (d, J = 7.6 Hz, 1H), 7.13 (s, 1H), 7.10 (dd, J = 7.6, 1.6 Hz, 1H), 3.70 (s, 2H), 2.49 – 2.53 (m, 2H), 2.38 – 2.42 (m, 2H), 2.36 (s, 3H), 2.24 – 2.34 (m, 1H), 1.60 – 1.66 (m, 1H), 1.33 (s, 12H). ^{13}C NMR (126 MHz, $CDCl_3$) δ 144.9, 141.0, 136.5, 132.1, 127.2, 83.8, 53.3, 51.4, 41.3, 25.3, 22.0, 19.0. HRMS (ESI$^+$): m/z for $C_{18}H_{27}BIO_2$ $[M+H]^+$ calcd. 413.1143, found: 413.1154.

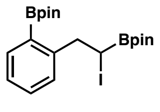

2-(1-iodo-2-(2-(4,4,5,5-tetramethyl-1,3,2-dioxaborolan-2-yl)phenyl)ethyl)-4,4,5,5-tetramethyl-1,3,2-dioxaborolane (4a) Prepared according to the general procedure E. The crude mixture was purified by flash column chromatography to give compound **4a** as colorless oil in 48% yield (23 mg). ^{1}H NMR (600 MHz, $CDCl_3$) δ 7.78 (dd, J = 7.4, 1.5 Hz, 1H), 7.32 (ddd, J = 7.6, 7.3, 1.5 Hz, 1H), 7.29 (dd, J = 7.7, 1.4 Hz, 1H), 7.22 (ddd, J = 7.3, 7.2, 1.4 Hz, 1H), 3.57 (dd, J = 12.3, 6.2 Hz, 1H), 3.49 (dd, J = 10.5, 6.2 Hz, 1H), 3.34 (dd, J = 12.4, 10.4 Hz, 1H), 1.36 (s, 12H), 1.22 (s, 6H), 1.20 (s, 6H). ^{13}C NMR (151 MHz, $CDCl_3$) δ 147.9, 136.7, 131.1, 130.4, 126.4, 84.2, 84.0, 41.6, 25.23, 25.22, 24.7, 24.5. HRMS (ESI$^+$): m/z for $C_{20}H_{32}B_2IO_4$ $[M+H]^+$ calcd. 485.1526, found: 485.1530.

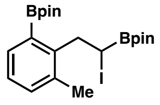

2-(1-iodo-2-(2-methyl-6-(4,4,5,5-tetramethyl-1,3,2-dioxaborolan-2-yl)phenyl)ethyl)-4,4,5,5-tetramethyl-1,3,2-dioxaborolane (4b) Prepared according to the general procedure E. The crude mixture was purified by flash column chromatography to give compound **4b** as colorless oil in 64% yield (31 mg). ^{1}H NMR (600 MHz, $CDCl_3$) δ 7.64 (dd, J = 7.4, 1.5 Hz, 1H), 7.20 (d, J = 7.5 Hz, 1H), 7.12 (dd, J = 7.4, 7.4 Hz, 1H), 3.57 – 3.49 (m, 3H), 2.44 (s, 3H), 1.364 (s, 6H), 1.360 (s, 6H), 1.18 (s, 6H), 1.16 (s, 6H). ^{13}C NMR (151 MHz, $CDCl_3$) δ 146.2, 136.4, 134.6, 133.7, 126.3, 84.1, 83.9, 36.2, 25.3, 25.1, 24.5, 24.4, 20.9 ppm. HRMS (ESI$^+$): m/z for $C_{21}H_{34}B_2IO_4$ $[M+H]^+$ calcd. 499.1682, found: 499.1683.

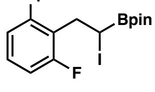

2-(3-fluoro-2-(2-iodo-2-(4,4,5,5-tetramethyl-1,3,2-dioxaborolan-2-yl)ethyl)phenyl)-4,4,5,5-tetramethyl-1,3,2-dioxaborolane (4c) Prepared according to the general procedure E with K_2CO_3 (1 equiv) as the base. The crude mixture was purified by column chromatography to give compound **4c** as colorless

oil in 42% yield (21 mg). ^{1}H NMR (600 MHz, $CDCl_3$) δ 7.55 (dd, *J* = 7.2, 1.1 Hz, 1H), 7.19 – 7.22 (m, 1H), 7.06 – 7.09 (m, 1H), 3.57 – 3.61 (m, 1H), 3.48 – 3.53 (m, 2H), 1.36 (s, 12H), 1.20 (s, 12H). ^{13}C NMR (126 MHz, $CDCl_3$) δ 161.0 (d, *J* = 246.2 Hz), 134.2 (d, *J* = 14.1 Hz), 132.0 (d, *J* = 3.5 Hz), 128.0 (d, *J* = 7.9 Hz), 118.4 (d, *J* = 23.3 Hz), 84.3, 84.2, 32.5 (d, *J* = 2.6 Hz), 25.3, 25.1, 24.54, 24.46. ^{19}F NMR (376 MHz, $CDCl_3$) δ -116.1. HRMS (ESI^+): m/z for $C_{20}H_{30}B_2FIO_4Na$ $[M+Na]^+$ calcd. 525.1251, found: 525.1244.

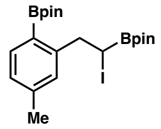

2-(2-(2-iodo-2-(4,4,5,5-tetramethyl-1,3,2-dioxaborolan-2-yl)ethyl)-4-methylphenyl)-4,4,5,5-tetramethyl-1,3,2-dioxaborolane (4d) Prepared according to the general procedure E. The crude mixture was purified by flash column to give compound **4d** as colorless oil in 46% yield (23 mg). ^{1}H NMR (600 MHz, $CDCl_3$) δ 7.67 (dd, *J* = 7.5, 2.3 Hz, 1H), 7.12 (s, 1H), 7.04 (d, *J* = 7.5 Hz, 1H), 3.60 (ddd, *J* = 12.3, 6.0, 2.3 Hz, 1H), 3.47 (ddd, *J* = 11.1, 5.9, 2.3 Hz, 1H), 3.25 – 3.29 (m, 1H), 2.30 (s, 3H), 1.35 (s, 12H), 1.22 (s, 6H), 1.20 (s, 6H). ^{13}C NMR (126 MHz, $CDCl_3$) δ 148.0, 141.2, 136.9, 131.3, 127.1, 84.1, 83.8, 41.6, 25.22, 25.19, 24.8, 24.4, 21.8. HRMS (ESI^+): m/z for $C_{21}H_{34}B_2IO_4$ $[M+H]^+$ calcd. 499.1682, found: 499.1694.

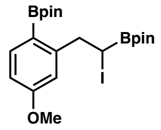

2-(2-(2-iodo-2-(4,4,5,5-tetramethyl-1,3,2-dioxaborolan-2-yl)ethyl)-4-methoxyphenyl)-4,4,5,5-tetramethyl-1,3,2-dioxaborolane (4e) Prepared according to the general procedure E. The crude mixture was purified by flash column chromatography to give compound **4e** as colorless oil in 45% yield (23 mg). ^{1}H NMR (500 MHz, $CDCl_3$) δ 7.73 (d, *J* = 8.2 Hz, 1H), 6.84 (d, *J* = 2.5 Hz, 1H), 6.75 (dd, *J* = 8.3, 2.5 Hz, 1H), 3.80 (s, 3H), 3.56 (dd, *J* = 12.1, 6.3 Hz, 1H), 3.49 (dd, *J* = 10.1, 6.4 Hz, 1H), 3.30 (dd, *J* = 12.1, 10.1 Hz, 1H), 1.34 (s, 12H), 1.23 (s, 6H), 1.20 (s, 6H). ^{13}C NMR (126 MHz, $CDCl_3$) δ 161.9, 150.1, 138.6, 116.5, 111.5, 84.2, 83.7, 55.4, 41.7, 25.23, 25.19, 24.7, 24.6. HRMS (ESI^+): m/z for $C_{21}H_{33}B_2IO_5Na$ $[M+Na]^+$ calcd. 537.1448, found: 537.1448.

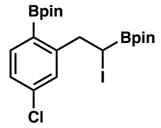

2-(4-chloro-2-(2-iodo-2-(4,4,5,5-tetramethyl-1,3,2-dioxaborolan-2-yl)ethyl)phenyl)-4,4,5,5-tetramethyl-1,3,2-dioxaborolane (4f) Prepared according to the general procedure E. The crude mixture was purified by flash column chromatography to give compound **4f** as colorless oil in 23% yield (12 mg). ^{1}H NMR (600 MHz, $CDCl_3$) δ 7.70 (d, *J* = 8.0 Hz, 1H), 7.32 (d, *J* = 2.1 Hz, 1H), 7.20 (dd, *J* = 8.0, 2.1 Hz, 1H), 3.60 (dd, *J* = 12.3, 5.7 Hz, 1H), 3.43 (dd, *J* = 11.3, 5.7 Hz, 1H), 3.25 (*appears* t, *J* = 11.8 Hz, 1H), 1.351 (s, 6H), 1.349 (s, 6H), 1.23 (s, 6H),

1.21 (s, 6H). ^{13}C NMR (126 MHz, $CDCl_3$) δ 149.9, 138.1, 137.2, 130.6, 126.5, 84.4, 84.2, 41.4, 25.23, 25.19, 24.8, 24.4. HRMS (ESI$^+$): m/z for $C_{20}H_{30}B_2ClIO_4Na$ $[M+Na]^+$ calcd. 541.0956, found: 541.0958.

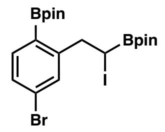

2-(4-bromo-2-(2-iodo-2-(4,4,5,5-tetramethyl-1,3,2-dioxaborolan-2-yl)ethyl)phenyl)-4,4,5,5-tetramethyl-1,3,2-dioxaborolane (4g) Prepared according to the general procedure E. The crude mixture was purified by flash column chromatography to give compound **4g** as colorless oil in 18% yield (10 mg). ^{1}H NMR (600 MHz, $CDCl_3$) δ 7.63 (d, *J* = 8.0 Hz, 1H), 7.49 (d, *J* = 1.9 Hz, 1H), 7.36 (dd, *J* = 7.8, 2.0 Hz, 1H), 3.60 (dd, *J* = 12.3, 5.6 Hz, 1H), 3.42 (dd, *J* = 11.4, 5.6 Hz, 1H), 3.23 (*app.* t, *J* = 11.9 Hz, 1H), 1.35 (s, 12H), 1.24 (s, 6H), 1.21 (s, 6H). ^{13}C NMR (126 MHz, $CDCl_3$) δ 150.0, 138.2, 133.4, 129.5, 125.9, 84.4, 84.3, 41.4, 25.23, 25.18, 24.8, 24.4. HRMS (ESI$^+$): m/z for $C_{20}H_{31}B_2BrIO_4$ $[M+H]^+$ calcd. 563.0631, found: 563.0633.

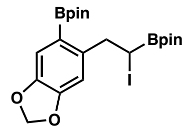

2-(6-(2-iodo-2-(4,4,5,5-tetramethyl-1,3,2-dioxaborolan-2-yl)ethyl)benzo[*d*][1,3]dioxol-5-yl)-4,4,5,5-tetramethyl-1,3,2-dioxaborolane (4h) Prepared according to the general procedure E with K_2CO_3 (1 equiv) as the base. The crude mixture was purified by flash column chromatography to give compound **4h** as colorless oil in 47% yield (25 mg). ^{1}H NMR (600 MHz, $CDCl_3$) δ 7.22 (s, 1H), 6.82 (s, 1H), 5.91 (d, *J* = 1.6 Hz, 2H), 3.55 (dd, *J* = 12.6, 6.2 Hz, 1H), 3.41 (dd, *J* = 10.6, 6.2 Hz, 1H), 3.23 (dd, *J* = 12.6, 10.7 Hz, 1H), 1.33 (s, 12H), 1.23 (s, 6H), 1.22 (s, 6H). ^{13}C NMR (151 MHz, $CDCl_3$) δ 149.9, 146.1, 143.8, 115.6, 111.4, 101.2, 84.2, 83.9, 41.2, 25.20, 25.18, 24.8, 24.5. HRMS (ESI$^+$): m/z for $C_{21}H_{31}B_2IO_6Na$ $[M+Na]^+$ calcd. 551.1244, found: 551.1241.

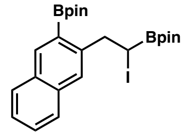

2-(1-iodo-2-(3-(4,4,5,5-tetramethyl-1,3,2-dioxaborolan-2-yl)naphthalen-2-yl)ethyl)-4,4,5,5-tetramethyl-1,3,2-dioxaborolane (4i) Prepared according to the general procedure E with K_2CO_3 (1 equiv) as the base. The crude mixture was purified by flash column chromatography to give compound **4i** as colorless oil in 32% yield (17 mg). ^{1}H NMR (600 MHz, $CDCl_3$) δ 8.35 (s, 1H), 7.83 (d, *J* = 8.0 Hz, 1H), 7.73 (s, 1H), 7.72 (d, *J* = 6.4 Hz, 1H), 7.47 (ddd, *J* = 8.2, 6.8, 1.4 Hz, 1H), 7.43 (ddd, *J* = 8.0, 6.8, 1.3 Hz, 1H), 3.73 (dd, *J* = 12.3, 5.6 Hz, 1H), 3.58 (dd, *J* = 11.0, 5.6 Hz, 1H), 3.49 (t, *J* = 11.7 Hz 1H), 1.41 (s, 12H), 1.21 (s, 6H), 1.16 (s, 6H). ^{13}C NMR (151 MHz, $CDCl_3$) δ 143.4, 138.5, 135.0, 131.9, 128.7, 128.6, 127.6, 127.5, 125.8, 84.18, 84.17, 41.8, 25.28, 25.25, 24.9, 24.4. HRMS (ESI$^+$): m/z for $C_{24}H_{34}B_2IO_4$ $[M+H]^+$ calcd. 535.1682, found: 535.1680.

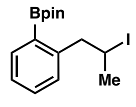

2-(2-(2-iodopropyl)phenyl)-4,4,5,5-tetramethyl-1,3,2-dioxaborolane (6a) Prepared according to the general procedure F. The crude mixture was purified by flash column chromatography to give compound **6a** as colorless oil in 67% yield (25 mg). ^{1}H NMR (500 MHz, $CDCl_3$) δ 7.82 (dd, J = 7.4, 1.6 Hz, 1H), 7.36 – 7.39 (m, 1H), 7.24 – 7.28 (m, 1H), 7.19 (dd, J = 7.5, 1.1 Hz, 1H), 4.31 – 4.38 (m, 1H), 3.59 (dd, J = 13.3, 6.9 Hz, 1H), 3.31 (dd, J = 13.4, 7.6 Hz, 1H), 1.88 (d, J = 6.8 Hz, 3H), 1.36 (s, 6H), 1.36 (s, 6H). ^{13}C NMR (126 MHz, $CDCl_3$) δ 146.8, 136.9, 131.2, 130.7, 126.5, 84.1, 49.6, 31.8, 28.4, 25.3, 25.2. HRMS (ESI$^+$): m/z for $C_{15}H_{22}BIO_2Na$ $[M+Na]^+$ calcd. 395.0650, found: 395.0667.

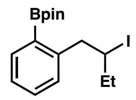

2-(2-(2-iodobutyl)phenyl)-4,4,5,5-tetramethyl-1,3,2-dioxaborolane (6b) Prepared according to the general procedure F. The crude mixture was purified by flash column chromatography to give compound **6b** as colorless oil in 36% yield (14 mg). ^{1}H NMR (500 MHz, $CDCl_3$) δ 7.82 (dd, J = 7.4, 1.5 Hz, 1H), 7.36 – 7.39 (m, 1H), 7.24 – 7.28 (m, 1H), 7.20 (dd, J = 7.7, 1.2 Hz, 1H), 4.25 – 4.30 (m, 1H), 3.54 (dd, J = 13.6, 7.7 Hz, 1H), 3.41 (dd, J = 13.6, 6.9 Hz, 1H), 1.74 – 1.80 (m, 2H), 1.36 (s, 6H), 1.35 (s, 6H), 1.05 (t, J = 7.2 Hz, 3H). ^{13}C NMR (126 MHz, $CDCl_3$) δ 147.0, 136.9, 131.2, 130.8, 126.4, 84.0, 47.4, 44.8, 33.0, 25.3, 25.2, 14.8. HRMS (ESI$^+$): m/z for $C_{16}H_{24}BIO_2Na$ $[M+Na]^+$ calcd. 409.0806, found: 409.0811.

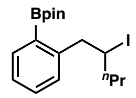

2-(2-(2-iodopentyl)phenyl)-4,4,5,5-tetramethyl-1,3,2-dioxaborolane (6c) Prepared according to the general procedure F. The crude mixture was purified to give compound **6c** as colorless oil in 35% yield (14 mg). ^{1}H NMR (500 MHz, $CDCl_3$) δ 7.83 (dd, J = 7.4, 1.5 Hz, 1H), 7.36 – 7.39 (m, 1H), 7.24 – 7.27 (m, 1H), 7.21 (d, J = 7.6 Hz, 1H), 4.28 – 4.33 (m, 1H), 3.55 (dd, J = 13.5, 7.7 Hz, 1H), 3.42 (dd, J = 13.6, 6.8 Hz, 1H), 1.76 – 1.85 (m, 1H), 1.59 – 1.71 (m, 2H), 1.37 – 1.44 (m, 1H), 1.36 (s, 6H), 1.35 (s, 6H), 0.90 (t, J = 7.1 Hz, 3H). ^{13}C NMR (126 MHz, $CDCl_3$) δ 147.0, 136.9, 131.1, 130.9, 126.4, 84.0, 47.8, 42.6, 41.9, 25.3, 25.2, 23.5, 13.6. HRMS (ESI$^+$): m/z for $C_{17}H_{27}BIO_2$ $[M+H]^+$ calcd. 401.1143, found: 401.1154.

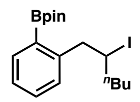

2-(2-(2-iodohexyl)phenyl)-4,4,5,5-tetramethyl-1,3,2-dioxaborolane (6d) Prepared according to the general procedure F. The crude mixture was purified by flash column chromatography to give compound **6d** as colorless oil in 34% yield (14 mg). ^{1}H NMR (500 MHz, $CDCl_3$) δ 7.82 (dd, J = 7.5, 1.5 Hz, 1H), 7.36 – 7.39 (m, 1H), 7.24 – 7.27 (m, 1H), 7.20 (d, J = 7.5 Hz, 1H), 4.26 – 4.32 (m, 1H), 3.54 (dd, J = 13.6, 7.7 Hz, 1H), 3.41 (dd, J = 13.6, 6.8 Hz, 1H), 1.77 – 1.84 (m, 1H), 1.57

– 1.71 (m, 2H), 1.36 (s, 6H), 1.35 (s, 6H), 1.21 – 1.39 (m, 3H), 0.89 (t, *J* = 7.2 Hz, 3H). ^{13}C NMR (126 MHz, $CDCl_3$) δ 147.0, 136.9, 131.1, 130.8, 126.4, 84.0, 47.8, 42.8, 39.6, 32.4, 25.3, 25.2, 22.3, 14.4. HRMS (ESI$^+$): m/z for $C_{18}H_{28}BIO_2Na$ $[M+Na]^+$ calcd. 437.1119, found: 437.1110.

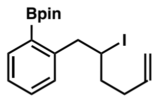

2-(2-(2-iodohex-5-en-1-yl)phenyl)-4,4,5,5-tetramethyl-1,3,2-dioxaborolane (6e) Prepared according to the general procedure F. The crude mixture was purified by flash column chromatography to give compound **6e** as colorless oil in 32% yield (13 mg). ^{1}H NMR (500 MHz, $CDCl_3$) δ 7.83 (dd, *J* = 7.5, 1.6 Hz, 1H), 7.36 – 7.39 (m, 1H), 7.24 – 7.27 (m, 1H), 7.20 (dd, *J* = 7.5, 1.2 Hz, 1H), 5.68 – 5.76 (m, 1H), 5.02 – 5.06 (m, 1H), 4.94 – 4.97 (m, 1H), 4.25 – 4.30 (m, 1H), 3.58 (dd, *J* = 13.6, 7.6 Hz, 1H), 3.42 (dd, *J* = 13.6, 7.0 Hz, 1H), 2.34 – 2.41 (m, 1H), 2.11 – 2.19 (m, 1H), 1.85 – 1.93 (m, 1H), 1.72 – 1.79 (m, 1H), 1.35 (s, 6H), 1.35 (s, 6H). ^{13}C NMR (126 MHz, $CDCl_3$) δ 146.8, 137.5, 136.9, 131.2, 130.8, 126.4, 115.7, 84.1, 47.8, 41.6, 38.8, 34.4, 25.4, 25.2. HRMS (ESI$^+$): m/z for $C_{18}H_{26}BIO_2Na$ $[M+Na]^+$ calcd. 435.0963, found: 435.0961.

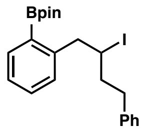

2-(2-(2-iodo-4-phenylbutyl)phenyl)-4,4,5,5-tetramethyl-1,3,2-dioxaborolane (6f) Prepared according to the general procedure F. The crude mixture was purified by flash column chromatography to give compound **6f** as colorless oil in 26% yield (12 mg). ^{1}H NMR (500 MHz, $CDCl_3$) δ 7.82 (dd, *J* = 7.4, 1.5 Hz, 1H), 7.35 – 7.39 (m, 1H), 7.23 – 7.27 (m, 3H), 7.12 – 7.20 (m, 4H), 4.24 – 4.30 (m, 1H), 3.62 (dd, *J* = 13.5, 7.5 Hz, 1H), 3.44 (dd, *J* = 13.5, 7.2 Hz, 1H), 2.93 – 2.99 (m, 1H), 2.65 – 2.71 (m, 1H), 2.06 – 2.14 (m, 1H), 1.95 – 2.02 (m, 1H), 1.30 (s, 12H). ^{13}C NMR (126 MHz, $CDCl_3$) δ 146.7, 141.4, 137.0, 131.2, 130.8, 128.9, 128.8, 126.5, 126.3, 84.0, 47.8, 41.4, 41.3, 36.3, 25.3, 25.1. HRMS (ESI$^+$): m/z for $C_{22}H_{28}BIO_2Na$ $[M+Na]^+$ calcd. 485.1119, found: 485.1120.

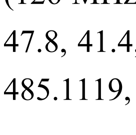

2-(2-(2-iodopropyl)-4-methylphenyl)-4,4,5,5-tetramethyl-1,3,2-dioxaborolane (6g) Prepared according to the general procedure F. The crude mixture was purified to give compound **6g** as colorless oil in 73% yield (28 mg). ^{1}H NMR (400 MHz, $CDCl_3$) δ 7.71 (d, *J* = 7.5 Hz, 1H), 7.06 – 7.09 (m, 1H), 7.00 (d, *J* = 1.6 Hz, 1H), 4.30 – 4.39 (m, 1H), 3.57 (dd, *J* = 13.3, 6.7 Hz, 1H), 3.27 (dd, *J* = 13.3, 7.8 Hz, 1H), 2.35 (s, 3H), 1.87 (d, *J* = 6.8 Hz, 3H), 1.35 (s, 6H), 1.34 (s, 6H). ^{13}C NMR (126 MHz, $CDCl_3$) δ 146.8, 141.4, 137.0, 131.5, 127.2, 83.9, 49.6, 31.8, 28.3, 25.3, 25.2, 21.9. HRMS (ESI$^+$): m/z for $C_{16}H_{25}BIO_2$ $[M+H]^+$ calcd. 387.0987, found: 387.0976.

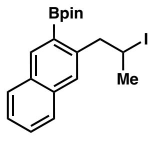

2-(3-(2-iodopropyl)naphthalen-2-yl)-4,4,5,5-tetramethyl-1,3,2-dioxaborolane (6h) Prepared according to the general procedure F. The crude mixture was purified by flash column chromatography to give compound **6h** as colorless oil in 47% yield (20 mg). ^{1}H NMR (500 MHz, $CDCl_3$) δ 8.39 (s, 1H), 7.85 (d, *J* = 8.0 Hz, 1H), 7.80 (d, *J* = 8.1 Hz, 1H), 7.62 (s, 1H), 7.49 – 7.52 (m, 1H), 7.43 – 7.46 (m, 1H), 4.41 – 4.48 (m, 1H), 3.74 (dd, *J* = 13.4, 6.8 Hz, 1H), 3.43 (dd, *J* = 13.4, 7.6 Hz, 1H), 1.92 (d, *J* = 6.8 Hz, 3H), 1.41 (s, 6H), 1.40 (s, 6H). ^{13}C NMR (126 MHz, $CDCl_3$) δ 142.1, 138.7, 135.0, 132.0, 128.9, 128.7, 127.71, 127.68, 126.0, 84.2, 49.9, 31.7, 28.4, 25.31, 25.25. HRMS (ESI$^+$): m/z for $C_{19}H_{24}BIO_2Na$ $[M+Na]^+$ calcd. 445.0806, found: 445.0810.

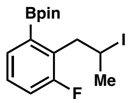

2-(3-(2-iodopropyl)naphthalen-2-yl)-4,4,5,5-tetramethyl-1,3,2-dioxaborolane (6i) Prepared according to the general procedure F. The crude mixture was purified by flash column chromatography to give compound **6i** as colorless oil in 51% yield (20 mg). ^{1}H NMR (500 MHz, $CDCl_3$) δ 7.59 (dd, *J* = 7.4, 1.3 Hz, 1H), 7.22 – 7.26 (m, 1H), 7.09 – 7.13 (m, 1H), 4.34 – 4.41 (m, 1H), 3.69 (ddd, *J* = 13.4, 6.5, 2.1 Hz, 1H), 3.44 (ddd, *J* = 13.3, 8.7, 1.5 Hz, 1H), 1.86 (dd, *J* = 6.8, 1.2 Hz, 3H), 1.375 (s, 6H), 1.367 (s, 6H). ^{13}C NMR (126 MHz, $CDCl_3$) δ 161.3 (d, *J* = 245.2 Hz), 133.3 (d, *J* = 13.9 Hz), 132.3 (d, *J* = 3.4 Hz), 128.2 (d, *J* = 8.1 Hz), 118.4 (d, *J* = 23.5 Hz), 84.4, 40.9 (d, *J* = 2.4 Hz), 29.0 (d, *J* = 1.9 Hz), 28.1, 25.3, 25.2. ^{19}F NMR (376 MHz, $CDCl_3$) δ -117.0. HRMS (ESI$^+$): m/z for $C_{15}H_{22}BFIO_2$ $[M+H]^+$ calcd. 391.0736, found: 391.0731.

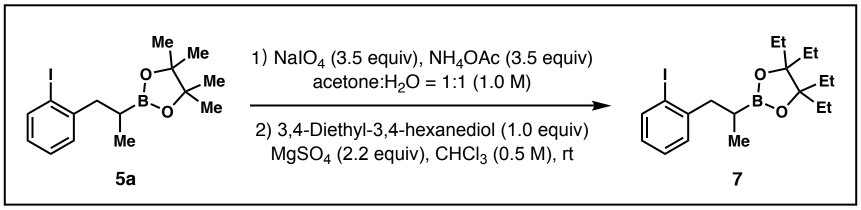

4,4,5,5-tetraethyl-2-(1-(2-iodophenyl)propan-2-yl)-1,3,2-dioxaborolane (7) To an oven-dried 20 mL vial with a magnetic stir bar was charged with $NaIO_4$ (749 mg, 3.5 mmol, 3.5 equiv), NH_4OAc (270 mg, 3.5 mmol, 3.5 equiv), and iodoarene **5a** [372 mg, 1.0 mmol, 1.0 equiv, dissolved in acetone-H_2O (1.0 mL, v:v = 1:1)]. The resulting slurry was allowed to stir at ambient temperature, and the reaction progress was monitored by TLC analyses. Upon completion of the reaction, water (5.0 mL) was added, and the mixture was extracted with ethyl acetate (3 × 5.0 mL). The combined organic extracts were washed with brine, dried over anhydrous Na_2SO_4, filtered, and concentrated under

reduced pressure. The resulting boronic acid crude product was dissolved in $CHCl_3$ (2.0 mL). Then 3,4-diethyl-3,4-hexanediol (174 mg, 1.0 mmol, 1.0 equiv) and $MgSO_4$ (265 mg, 2.2 mmol, 2.2 equiv) were added. The resulting mixture was allowed to stir at ambient temperature, and the reaction progress was monitored by TLC analyses. Upon completion of the reaction, water (2.0 mL) was added, and the mixture was extracted with ethyl acetate (3 × 5.0 mL). The combined organic extracts were washed with brine, dried over anhydrous Na_2SO_4, filtered, and concentrated under reduced pressure. The crude product was performed by flash column chromatography (gradient elution with hexane and ethyl acetate) to give **7** in 51% yield over two steps (218 mg) as a colorless oil. ^{1}H NMR (500 MHz, $CDCl_3$) δ 7.79 (dd, *J* = 7.9, 1.1 Hz, 1H), 7.20 – 7.26 (m, 2H), 6.82 – 6.86 (m, 1H), 2.95 (dd, *J* = 13.9, 7.5 Hz, 1H), 2.59 (dd, *J* = 13.9, 8.2 Hz, 1H), 1.57 – 1.68 (m, 8H), 1.45 – 1.52 (m, 1H), 1.01 (d, *J* = 7.4 Hz, 3H), 0.58 – 0.90 (m, 12H). ^{13}C NMR (126 MHz, $CDCl_3$) δ 145.3, 139.7, 130.3, 128.1, 127.7, 101.5, 88.4, 44.0, 26.61, 26.58, 15.9, 9.13, 9.09. HRMS (ESI$^+$): m/z for $C_{19}H_{31}BIO_2$ $[M+H]^+$ calcd. 429.1456, found: 429.1462.

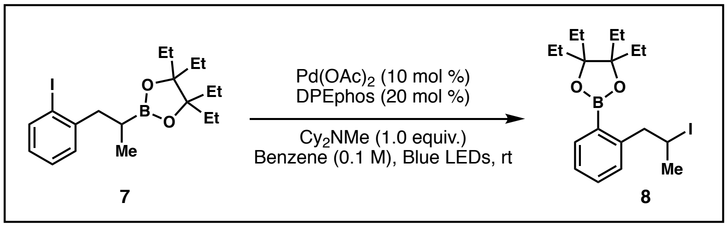

4,4,5,5-tetraethyl-2-(2-(2-iodopropyl)phenyl)-1,3,2-dioxaborolane (8) Prepared according to the general procedure F. The crude mixture was purified by flash column chromatography to give compound **8** as colorless oil in 47% yield (20 mg). ^{1}H NMR (500 MHz, $CDCl_3$) δ 7.87 (dd, *J* = 7.3, 1.6 Hz, 1H), 7.36 – 7.39 (m, 1H), 7.25 – 7.28 (m, 1H), 7.19 (d, *J* = 7.6 Hz, 1H), 4.38 – 4.45 (m, 1H), 3.59 (dd, *J* = 13.3, 7.1 Hz, 1H), 3.33 (dd, *J* = 13.3, 7.7 Hz, 1H), 1.88 (d, *J* = 6.7 Hz, 3H), 1.71 – 1.85 (m, 8H), 0.97 – 1.00 (m, 12H). ^{13}C NMR (126 MHz, $CDCl_3$) δ 146.7, 137.2, 131.1, 130.7, 126.4, 89.2, 49.6, 31.5, 28.5, 26.73, 26.72, 9.35, 9.33. HRMS (ESI$^+$): m/z for $C_{19}H_{31}BIO_2$ $[M+H]^+$ calcd. 429.1456, found: 429.1452.

Control Experiment with TEMPO as the Additive:

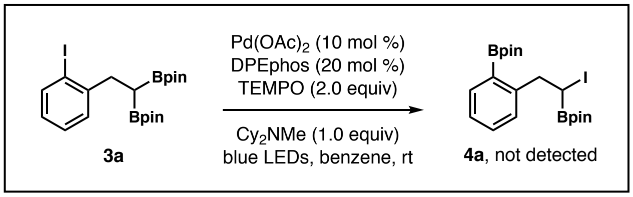

In a glove box, to an oven-dried 10 mL reaction tube equipped with a magnetic stir bar was charged with iodoarene **3a** (48 mg, 0.1 mmol, 1.0 equiv), $Pd(OAc)_2$ (2 mg, 0.01 mmol, 10 mol %), DPEphos (11 mg, 0.02 mmol, 20 mol %), TEMPO (31 mg, 0.2 mmol, 2.0 equiv) and Cy_2NMe (20 mg, 0.1 mmol, 1.0 equiv) under a nitrogen atmosphere. Degassed anhydrous benzene (1.0 mL) was added, and the reaction tube was sealed with a rubber septum. The reaction tube was taken out of the glove box and irradiated with 40 W Kessil 450 nm blue LED lamps (Kessil A160WE Tuna Blue LED) with cooling provided by a fan (temperature reached 37 °C). The distance from the lamp to the reaction tube is about 3 cm. The reaction progress was monitored by ^{1}H NMR analysis. Upon completion of the reaction, the reaction mixture was diluted with ethyl acetate (2.0 mL) and filtered through Celite. The filtrate was concentrated under reduced pressure. Analyses of the crude reaction product by ^{1}H NMR indicated that **3a** remained unreacted and the formation of product **4a** was not detected.

Radical Clock Experiments:

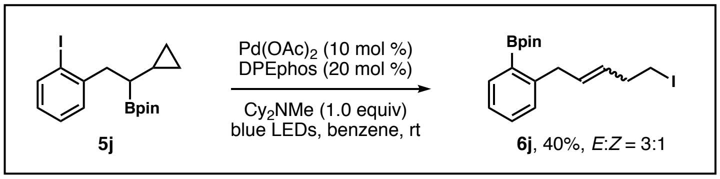

2-(2-(5-iodopent-2-en-1-yl)phenyl)-4,4,5,5-tetramethyl-1,3,2-dioxaborolane (6j)
Prepared according to *general procedure F*. The crude mixture was purified by flash column chromatography to give compound **6j** as a 3:1 mixture of *E*- and *Z*-isomers as colorless oil in 40% yield (16 mg).

E-isomer: ^{1}H NMR (600 MHz, $CDCl_3$) δ 7.78 – 7.80 (m, 1H), 7.35 – 7.38 (m, 1H), 7.18 – 7.21 (m, 2H), 5.68 – 5.75 (m, 1H), 5.37 – 5.42 (m, 1H), 3.65 (dd, *J* = 6.8, 1.5 Hz, 2H), 3.13 (t, *J* = 7.3 Hz, 2H), 2.54 – 2.57 (m, 2H), 1.34 (s, 12H). ^{13}C NMR (151 MHz, $CDCl_3$)

δ 147.3, 136.5, 133.9, 131.4, 129.5, 129.2, 125.7, 83.8, 38.8, 37.2, 25.3, 6.1. HRMS (ESI^+): m/z for $C_{17}H_{24}BIO_2Na$ $[M+Na]^+$ calcd. 421.0806, found: 421.0804.

Z-isomer: ^{1}H NMR (600 MHz, $CDCl_3$) δ 7.78 – 7.80 (m, 1H), 7.35 – 7.38 (m, 1H), 7.18 – 7.21(m, 2H), 5.68 – 5.75 (m, 1H), 5.37 – 5.42 (m, 1H), 3.71 (dd, *J* = 7.4, 1.7 Hz, 2H), 3.18 (t, *J* = 7.4 Hz, 2H), 2.76 – 2.81 (m, 2H), 1.35 (s, 12H). ^{13}C NMR (151 MHz, $CDCl_3$) δ 147.6, 136.6, 132.7, 131.5, 129.1, 128.2, 125.6, 83.9, 33.7, 32.1, 25.3, 5.7. HRMS (ESI^+): m/z for $C_{17}H_{24}BIO_2Na$ $[M+Na]^+$ calcd. 421.0806, found: 421.0804.

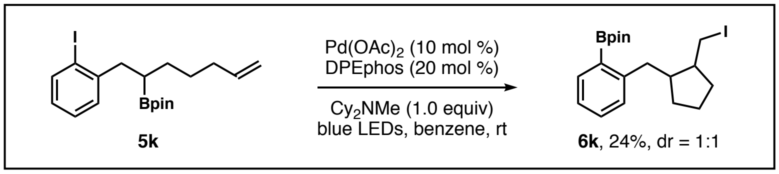

2-(2-((2-(iodomethyl)cyclopentyl)methyl)phenyl)-4,4,5,5-tetramethyl-1,3,2-dioxaborolane (6k) Prepared according to *general procedure F*. The crude mixture was purified by column chromatography to give compound **6k** as a 1:1 mixture of two diastereomers as colorless oil in 24% yield (10 mg).

Diastereomer 1: ^{1}H NMR (500 MHz, $CDCl_3$) δ 7.78 – 7.82 (m, 1H), 7.32 – 7.36 (m, 1H), 7.16 – 7.20 (m, 2H), 3.51 (dd, *J* = 9.4, 5.4 Hz, 1H), 3.16 – 3.20 (m, 1H), 3.10 (dd, *J* = 12.7, 4.4 Hz, 1H), 2.58 – 2.62 (m, 1H), 2.38 – 2.44 (m, 1H), 2.17 – 2.26 (m, 1H), 1.90 – 2.00 (m, 1H), 1.69 – 1.81 (m, 2H), 1.39 – 1.64 (m, 3H), 1.34 (s, 12H).

Diastereomer 2: ^{1}H NMR (500 MHz, $CDCl_3$) δ 7.78 – 7.82 (m, 1H), 7.32 – 7.36 (m, 1H), 7.16 – 7.20 (m, 2H), 3.34 (dd, *J* = 9.4, 3.8 Hz, 1H), 3.13 – 3.17 (m, 1H), 2.92 – 2.95 (m, 1H), 2.73 (dd, *J* = 12.7, 8.3 Hz, 1H), 1.90 – 2.00 (m, 1H), 1.83 – 1.89 (m, 1H), 1.69 – 1.81 (m, 1H), 1.39 – 1.64 (m, 5H), 1.34 (s, 12H).

The results from these reactions showed that: (1) the reaction is inhibited by a radical scavenger, 2,2,6,6-tetramethylpiperidine 1-oxyl radical (TEMPO); (2) the 1,4-boryl migration process in the reactions with **5j** forms a secondary alkyl radical intermediate, which underwent cyclopropane ring-opening to give alkene products; (3) the 1,4-boryl migration process in the reactions with **5k** forms a secondary alkyl radical intermediate, which underwent 5-exo-cyclization to give the corresponding product. These data support the radical nature of the reaction process.

Light ON/OFF Experiments:

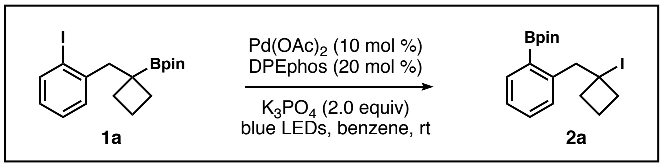

In a glove box, to an oven-dried 10 mL reaction tube equipped with a magnetic stir bar was charged with iodoarene **1a** (40 mg, 0.1 mmol, 1.0 equiv), $Pd(OAc)_2$ (2 mg, 0.01 mmol, 10 mol %), DPEphos (11 mg, 0.02 mmol, 20 mol %), and K_3PO_4 (43 mg, 0.2 mmol, 2.0 equiv) under a nitrogen atmosphere. Degassed anhydrous benzene (1.0 mL) was added, and the reaction tube was sealed with a rubber septum. Six such reaction tubes were setup in parallel. The reaction tubes were taken out of the glove box and irradiated with 40 W Kessil 450 nm blue LED lamps (Kessil A160WE Tuna Blue LED) with cooling provided by a fan (temperature reached 37 °C). The distance from the lamp to the reaction tube is about 3 cm. After irradiation for 1 hour, one tube was removed and analyzed by ^1H NMR spectroscopy of the crude reaction mixture. The yield of **2a** was calculated by using CH_2Br_2 as an internal standard. The remaining tubes were kept stirring without irradiation for 1 hour. Then one tube was removed and analyzed by ^1H NMR spectroscopy of the crude reaction mixture. The yield of **2a** was calculated by using CH_2Br_2 as an internal standard. The processes were repeated for the rest reaction tubes to obtain the yields of **2a**. The results indicate that the reaction requires blue LED irradiation and it is not a chain reaction.

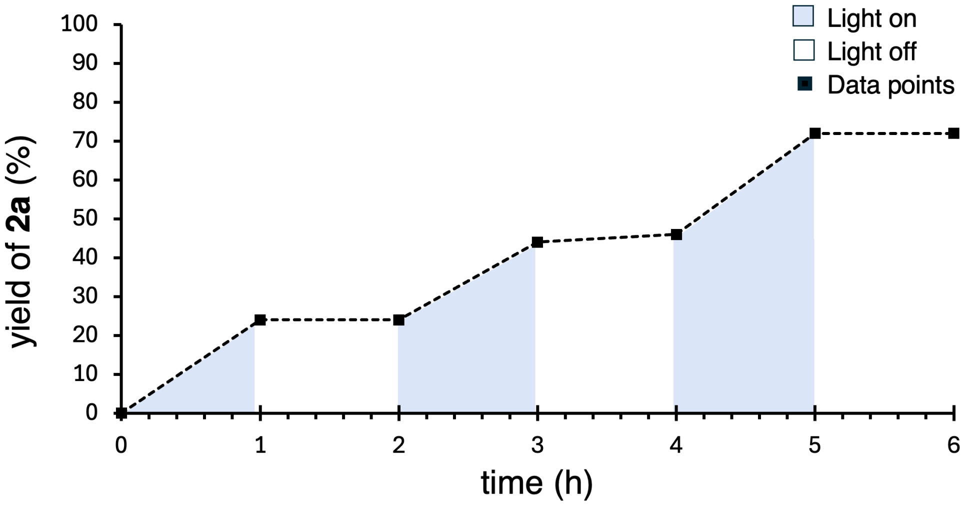

Crossover Experiment:

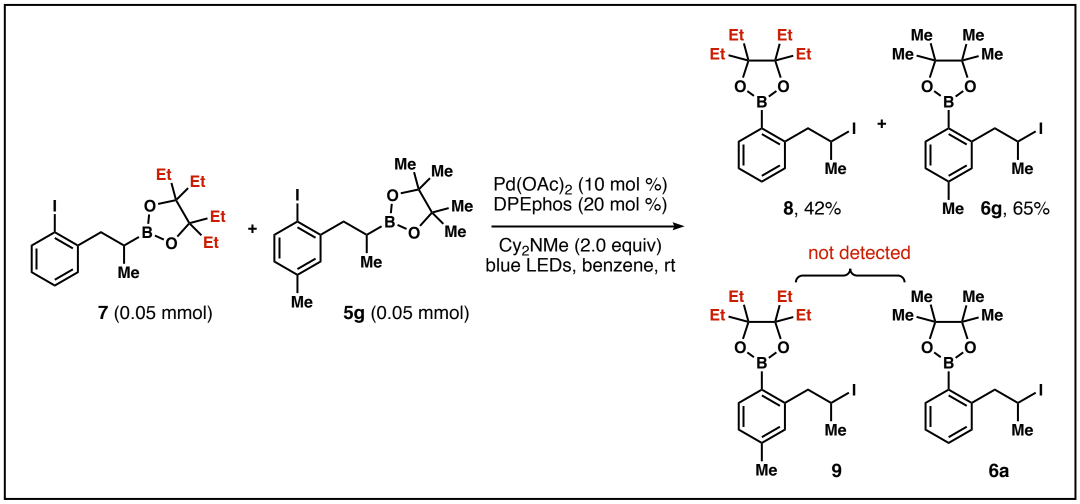

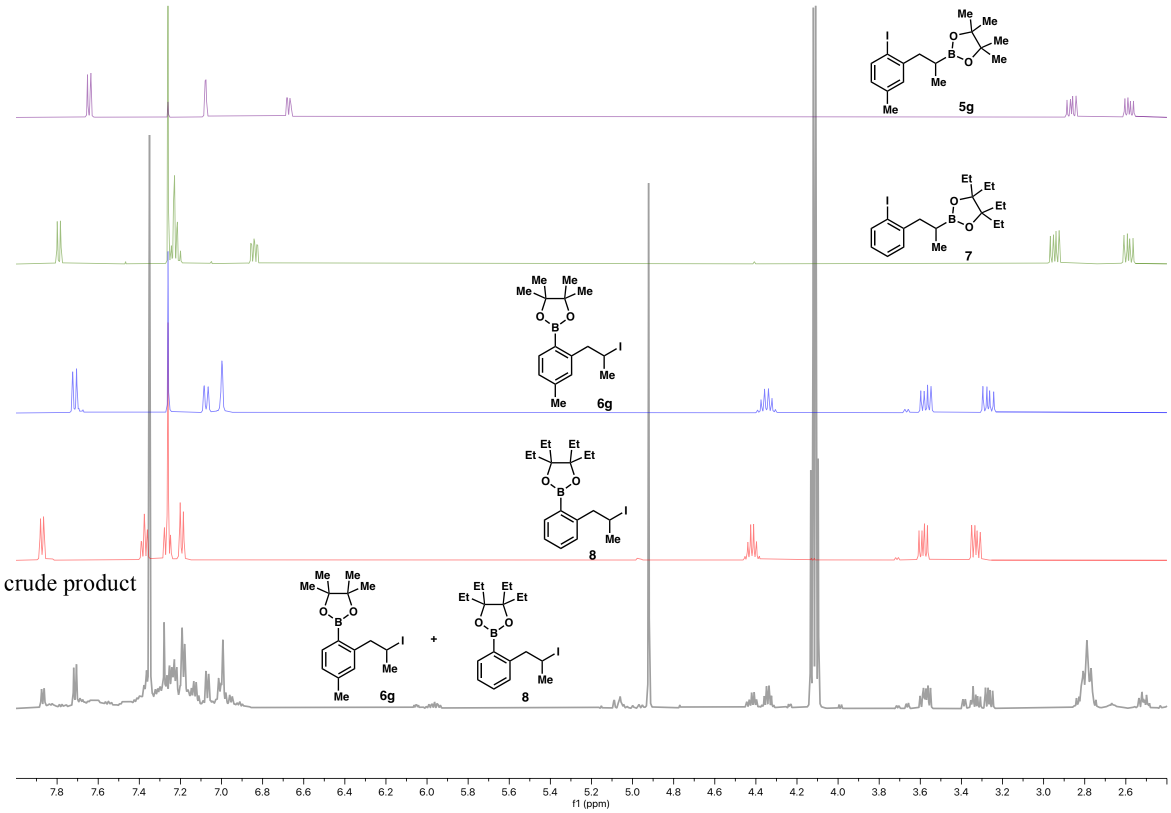

In a glove box, to an oven-dried 10 mL reaction tube equipped with a magnetic stir bar was charged with iodoarene **7** (0.05 mmol, 1.0 equiv), **5g** (0.05 mmol, 1.0 equiv), $Pd(OAc)_2$ (2 mg, 0.01 mmol), DPEphos (11 mg, 0.02 mmol), and Cy_2NMe (20 mg, 0.1

mmol) under a nitrogen atmosphere. Degassed anhydrous benzene (1.0 mL) was added, and the reaction tube was sealed with a rubber septum. The reaction tube was irradiated with 40 W Kessil 450 nm blue LED lamps (Kessil A160WE Tuna Blue LED) with cooling provided by a fan (temperature reached 37 °C). The distance from the lamp to the reaction tube is about 3 cm. The reaction progress was monitored by ^{1}H NMR analysis. Upon completion of the reaction, the reaction mixture was diluted with ethyl acetate (2.0 mL) and filtered through Celite. The filtrate was concentrated under reduced pressure. ^{1}H NMR analyses of the crude reaction mixtures indicate there is no crossover products (**9** and **6a**). The crude product was purified by flash column chromatography (gradient elution with hexanes and ethyl acetate) to afford product **6g** (65% yield) and **8** (42% yield).

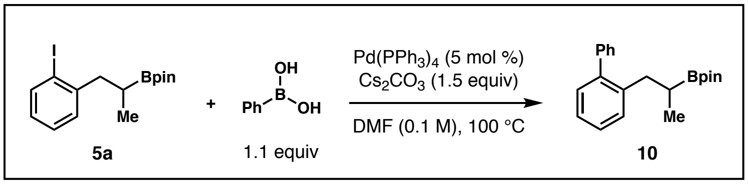

2-(1-([1,1'-biphenyl]-2-yl)propan-2-yl)-4,4,5,5-tetramethyl-1,3,2-dioxaborolane (10) In a glove box, to an oven-dried 10 mL reaction tube equipped with a magnetic stir bar was charged with iodoarene **5a** (37 mg, 0.1 mmol, 1.0 equiv), phenylboronic acid (14 mg, 0.11 mmol, 1.1 equiv), $Pd(PPh_3)_4$ (6 mg, 5 mol %), Cs_2CO_3 (49 mg, 0.15 mmol, 1.5 equiv), and anhydrous DMF (1.0 mL) under a nitrogen atmosphere. The reaction tube was sealed with a rubber septum and removed from the glove box. The tube was place in a heating block and was kept stirring at 100 °C. The reaction progress was monitored by TLC analyses. Upon completion of the reaction (~7 h), ethyl acetate (2 mL) was added. The resulting mixture was filtered through a pad of Celite. The filtrate was concentrated under reduced pressure. The crude reaction mixture was purified by flash column chromatography (eluent: hexanes and ethyl acetate) to afford product **10** as colorless oil in 65% yield (21 mg). ^{1}H NMR (500 MHz, $CDCl_3$) δ 7.30 – 7.41 (m, 6H), 7.24 – 7.27 (m, 1H), 7.19 – 7.22 (m, 1H), 7.17 (dd, J = 7.5, 1.8 Hz, 1H), 2.80 (dd, J = 14.0, 8.1 Hz, 1H), 2.54 (dd, J = 14.1, 7.8 Hz, 1H), 1.19 – 1.24 (m, 1H), 1.15 (s, 6H), 1.14 (s, 6H), 0.81 (d, J = 7.4 Hz, 3H). ^{13}C NMR (126 MHz, $CDCl_3$) δ 142.6, 142.5, 140.3, 130.3, 129.74, 129.72, 128.2, 127.4, 126.9, 125.8, 83.2, 36.1, 25.00, 24.96, 15.8. HRMS (ESI^{+}): m/z for $C_{21}H_{27}BO_2Na$ $[M+Na]^{+}$ calcd. 345.1996, found: 345.2003.

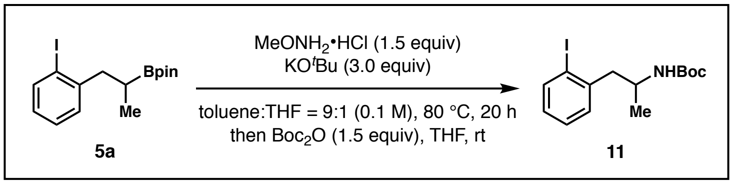

***tert*-butyl (1-(2-iodophenyl)propan-2-yl)carbamate (11)** In a glove box, to an oven-dried 10 mL tube equipped with a magnetic stir bar was charged with KO^{t}Bu (34 mg, 0.30 mmol, 3.0 equiv), $MeONH_2$•HCl (13 mg, 0.15 mmol, 1.5 equiv) and anhydrous THF (0.1 mL) under a nitrogen atmosphere. Then a solution of boronate **5a** (37 mg, 0.10 mmol, 1.0 equiv) in anhydrous toluene (0.9 mL) was added. The resulting solution was kept stirring at 80 °C for 20 h. Then the reaction mixture was cooled to ambient temperature, and Boc_2O (0.30 mL, 0.5 M in THF, 0.15 mmol, 1.5 equiv) was added. The resulting mixture was kept stirring for 1 h at ambient temperature. Upon completion of the reaction, Et_2O (5.0 mL) and water (2.0 mL) were added. The organic phase was separated, and the aqueous phase was extracted with Et_2O (2 × 5 mL). The combined organic extracts were dried over anhydrous Na_2SO_4 and filtered. The filtrate was concentrated under reduced pressure. The crude product was purified by flash column chromatography to give **11** as a white solid in 53% yield (19 mg). ^{1}H NMR (500 MHz, $CDCl_3$) δ 7.81 (dd, J = 8.0, 1.2 Hz, 1H), 7.21 – 7.28 (m, 2H), 6.88 – 6.91 (m, 1H), 4.46 (s, 1H), 3.94 – 4.07 (m, 1H), 2.81 – 2.99 (m, 2H), 1.37 (s, 9H), 1.19 (d, J = 6.7 Hz, 3H). ^{13}C NMR (126 MHz, $CDCl_3$) δ 155.4, 141.9, 139.8, 130.76, 128.54, 128.47, 101.8, 79.4, 47.7, 30.0, 28.7, 21.2. HRMS (ESI$^+$): m/z for $C_{14}H_{20}INO_2Na$ [M+Na]$^+$ calcd. 384.0431, found: 384.0431.

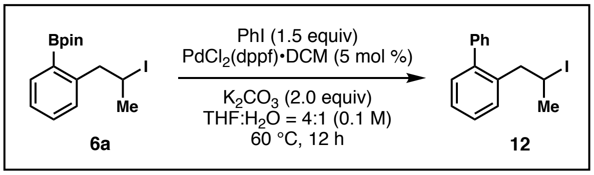

2-(2-iodopropyl)-1,1'-biphenyl (12) In a glove box, to an oven-dried 10 mL reaction tube equipped with a magnetic stir bar was charged with **6a** (37 mg, 0.1 mmol, 1.0 equiv), PhI (31 mg, 0.15 mmol, 1.5 equiv), $PdCl_2$(dppf)•DCM (4 mg, 5 mol %), K_2CO_3 (28 mg, 0.2 mmol, 2.0 equiv) and THF-H_2O (1.0 mL, v:v = 4:1) under a nitrogen atmosphere. The reaction tube was sealed with a rubber septum and removed from the glove box. The tube was place in a heating block and was kept stirring at 60 °C. The reaction progress was monitored by TLC analyses. Upon completion of the reaction (~12 h), Et_2O (5.0 mL) and

water (2.0 mL) were added. The organic phase was separated, and the aqueous phase was extracted with Et_2O (2 × 5 mL). Then the combined organic extracts were dried over anhydrous Na_2SO_4 and filtered. The filtrate was concentrated under reduced pressure. The crude product was purified by flash column chromatography to give **12** as colorless oil in 53% yield (17 mg). ^{1}H NMR (600 MHz, $CDCl_3$) δ 7.41 – 7.44 (m, 2H), 7.35 – 7.38 (m, 1H), 7.27 – 7.34 (m, 5H), 7.20 – 7.23 (m, 1H), 4.02 – 4.08 (m, 1H), 3.32 (dd, *J* = 14.4, 7.5 Hz, 1H), 3.13 (dd, *J* = 14.4, 7.6 Hz, 1H), 1.68 (d, *J* = 6.8 Hz, 3H). ^{13}C NMR (151 MHz, $CDCl_3$) δ 142.4, 141.7, 137.7, 130.7, 130.2, 129.6, 128.7, 127.8, 127.5, 127.2, 46.9, 28.4, 28.2.

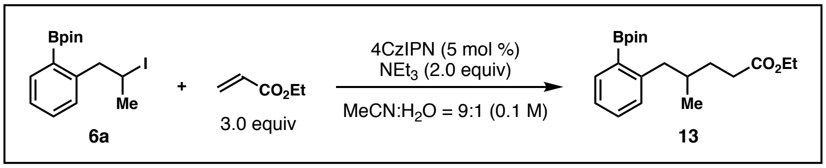

ethyl 4-methyl-5-(2-(4,4,5,5-tetramethyl-1,3,2-dioxaborolan-2-yl)phenyl)pentanoate (13) In a glove box, to an oven-dried 10 mL tube equipped with a magnetic stir bar was charged with **6a** (37 mg, 0.10 mmol, 1.0 equiv), ethyl acrylate (30 mg, 0.30 mmol, 3.0 equiv), and 4CzIPN (4 mg, 5 mol%). The reaction tube was sealed with a rubber septum and removed from the glove box. Then NEt_3 (20 mg, 0.20 mmol, 2.0 equiv) and MeCN-H_2O (9:1, 1.0 mL) were added to the reaction tube (using a syringe) under a nitrogen atmosphere. The reaction tube was irradiated with 40 W Kessil 450 nm blue LED lamps (Kessil A160WE Tuna Blue LED) with cooling provided by a fan (vial temperature reached 37 °C). The distance from the lamp to the reaction tube is about 3 cm. The reaction progress was monitored by ^{1}H NMR analyses. Upon completion of the reaction, ethyl acetate (2 mL) was added. The resulting mixture was filtered through a pad of Celite. The filtrate was concentrated under reduced pressure. The crude product was purified by flash column chromatography to give **13** as a colorless oil in 64% yield (22 mg). ^{1}H NMR (500 MHz, $CDCl_3$) δ 7.79 (dd, *J* = 7.5, 1.6 Hz, 1H), 7.31 – 7.34 (m, 1H), 7.16 – 7.19 (m, 1H), 7.12 (dd, *J* = 7.6, 1.2 Hz, 1H), 4.10 (q, *J* = 7.1 Hz, 2H), 2.95 (dd, *J* = 12.7, 5.8 Hz, 1H), 2.64 (dd, *J* = 12.7, 8.2 Hz, 1H), 2.38 (ddd, *J* = 15.8, 10.3, 5.6 Hz, 1H), 2.29 (ddd, *J* = 15.6, 10.2, 6.0 Hz, 1H), 1.72 – 1.79 (m, 1H), 1.64 – 1.70 (m, 1H), 1.45 – 1.55 (m, 1H), 1.34 (s, 12H), 1.23 (t, *J* = 7.1 Hz, 3H), 0.84 (d, *J* = 6.6 Hz, 3H). ^{13}C NMR (126 MHz, $CDCl_3$) δ 174.5, 148.5, 136.6, 130.9, 130.6, 125.4, 83.8, 60.5, 43.1, 36.5, 32.7, 32.3, 25.3, 25.2, 19.1, 14.6. HRMS (ESI$^+$): m/z for $C_{20}H_{31}BO_4Na$ $[M+Na]^+$ calcd. 369.2208, found: 369.2210.

Light Source:

Blue LED light: Kessil A160WE TUNA BLUE (KSA160WE-TB); 40 W Max.

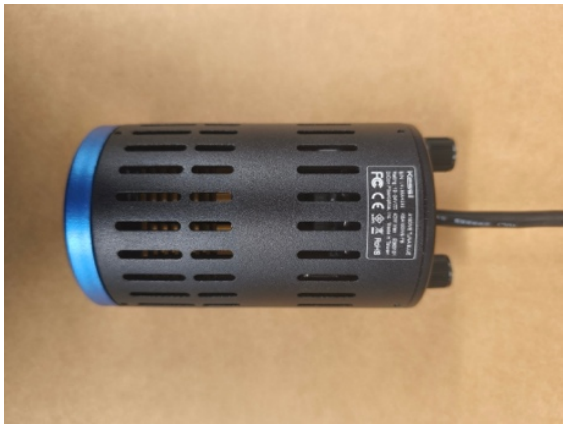

Reaction Setup:

An oven-dried 10 mL reaction tube equipped with a magnetic stir bar was sealed with a rubber septum and placed upright in a tube rack. Two 40 W Kessil A160WE blue LEDs (450 nm) were positioned perpendicular to the reaction tube at a distance of 3–4 cm, with forced-air cooling provided by a fan. During the irradiation, the temperature of the reaction typically stabilizes at 37 °C.

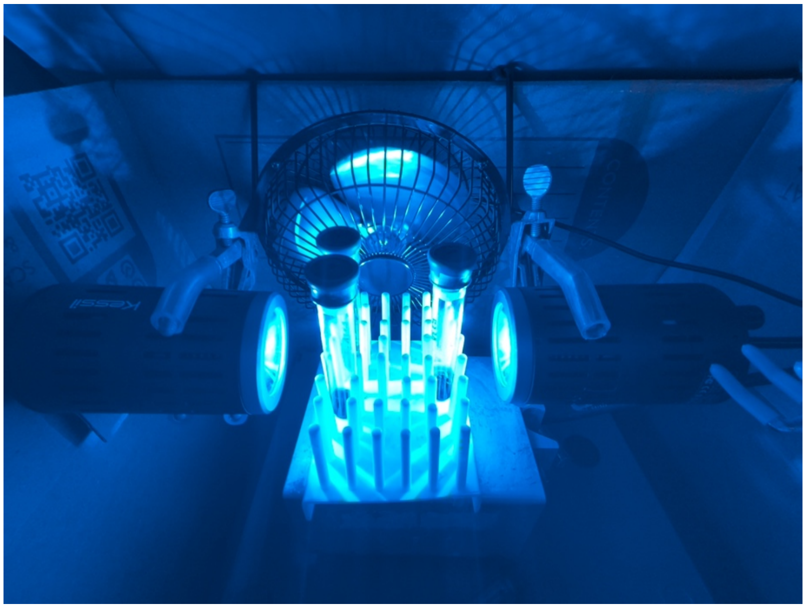

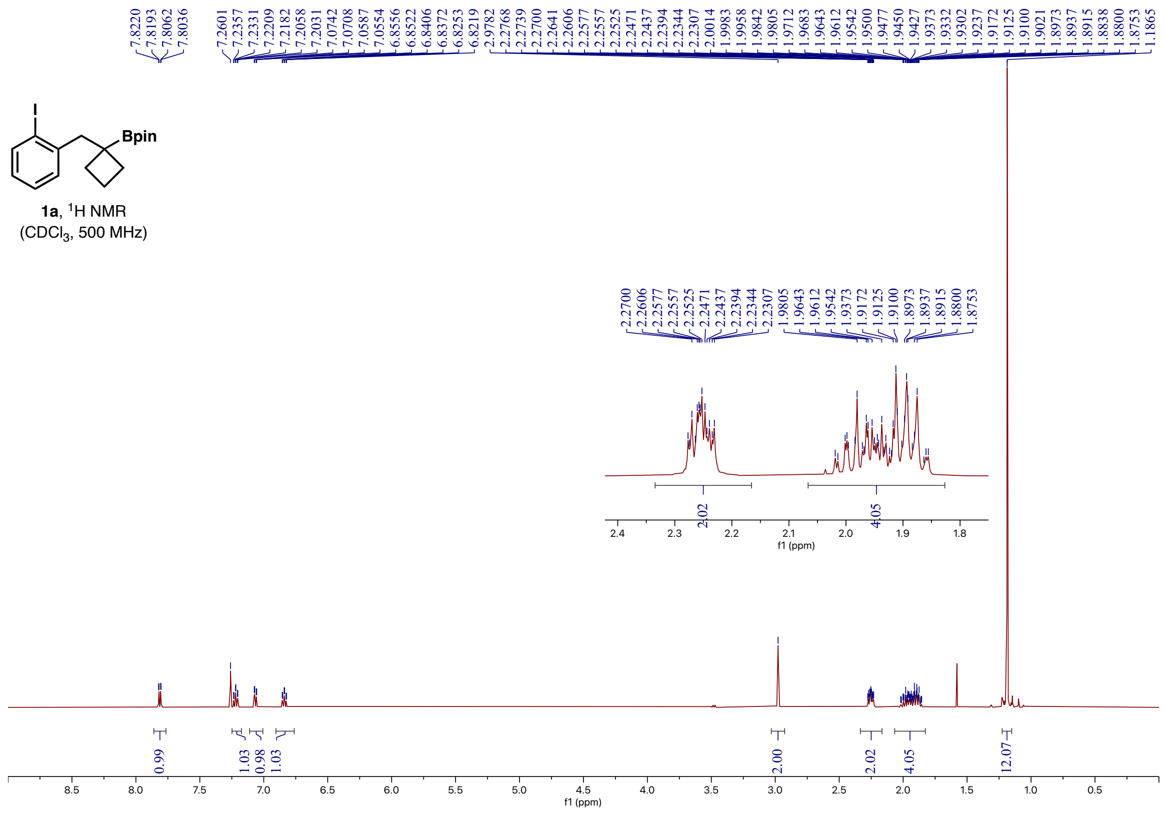

1a, ^{1}H NMR
($CDCl_3$, 500 MHz)

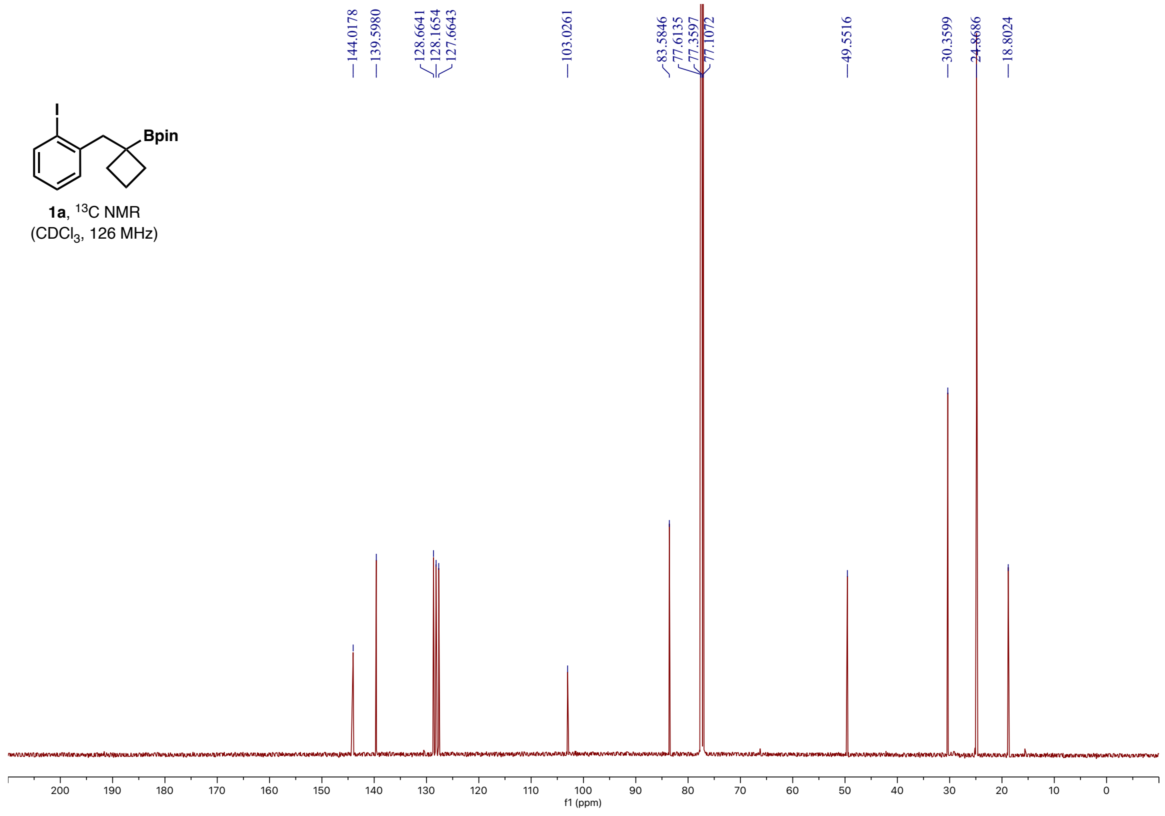

1a, ^{13}C NMR
($CDCl_3$, 126 MHz)

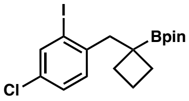

1b, ^{1}H NMR
($CDCl_3$, 500 MHz)

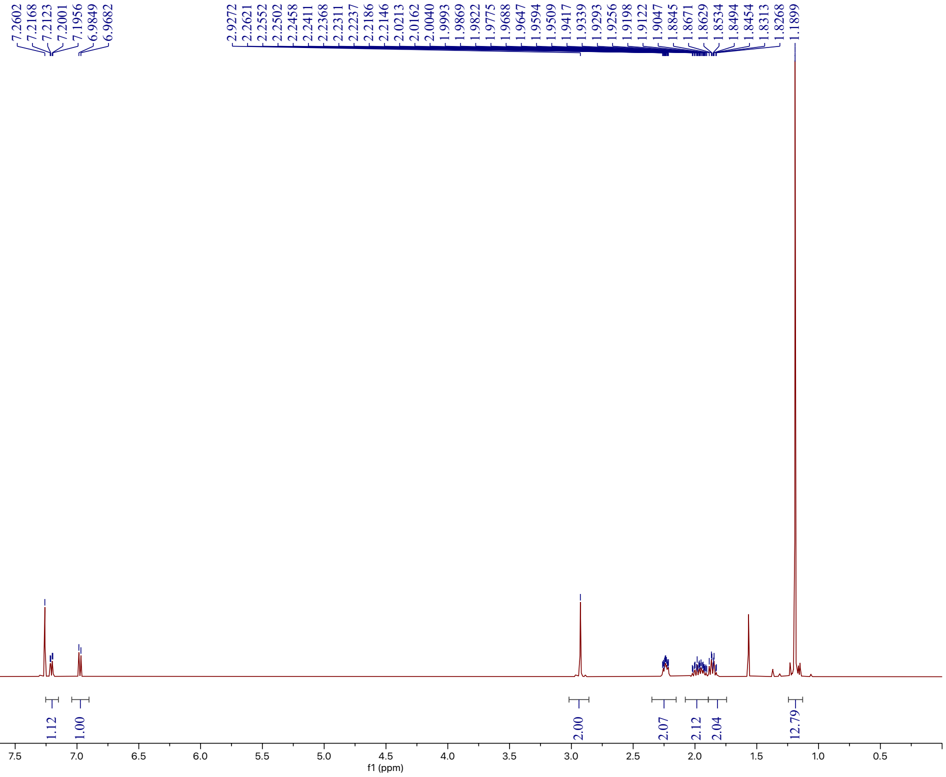

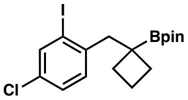

1b, ^{13}C NMR
($CDCl_3$, 126 MHz)

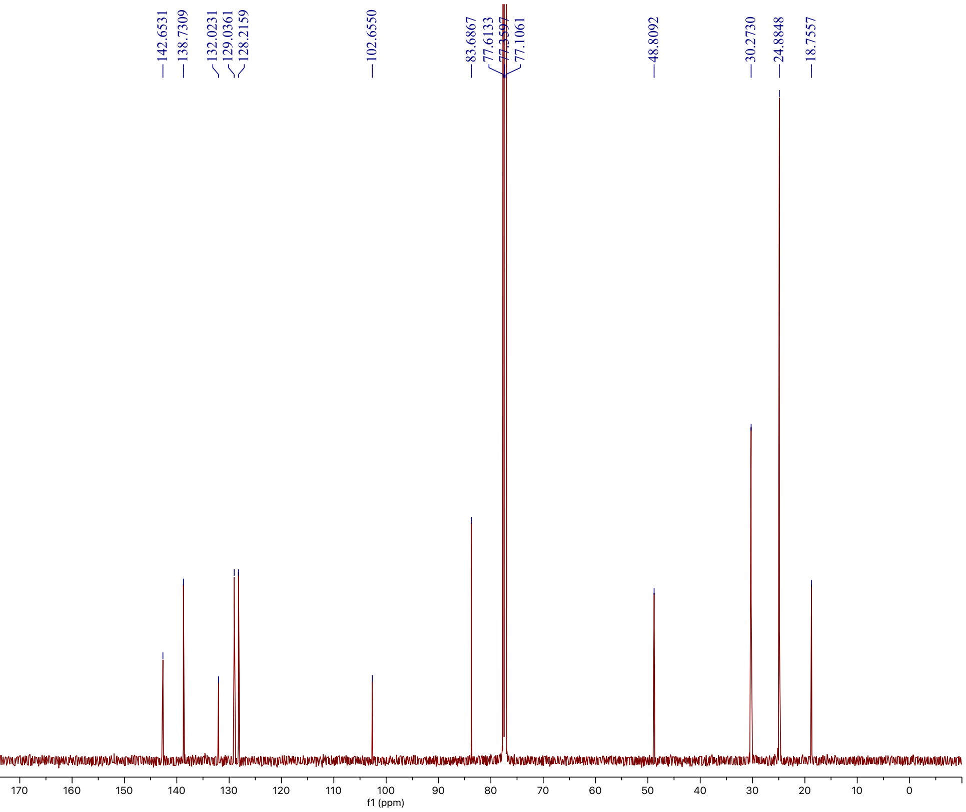

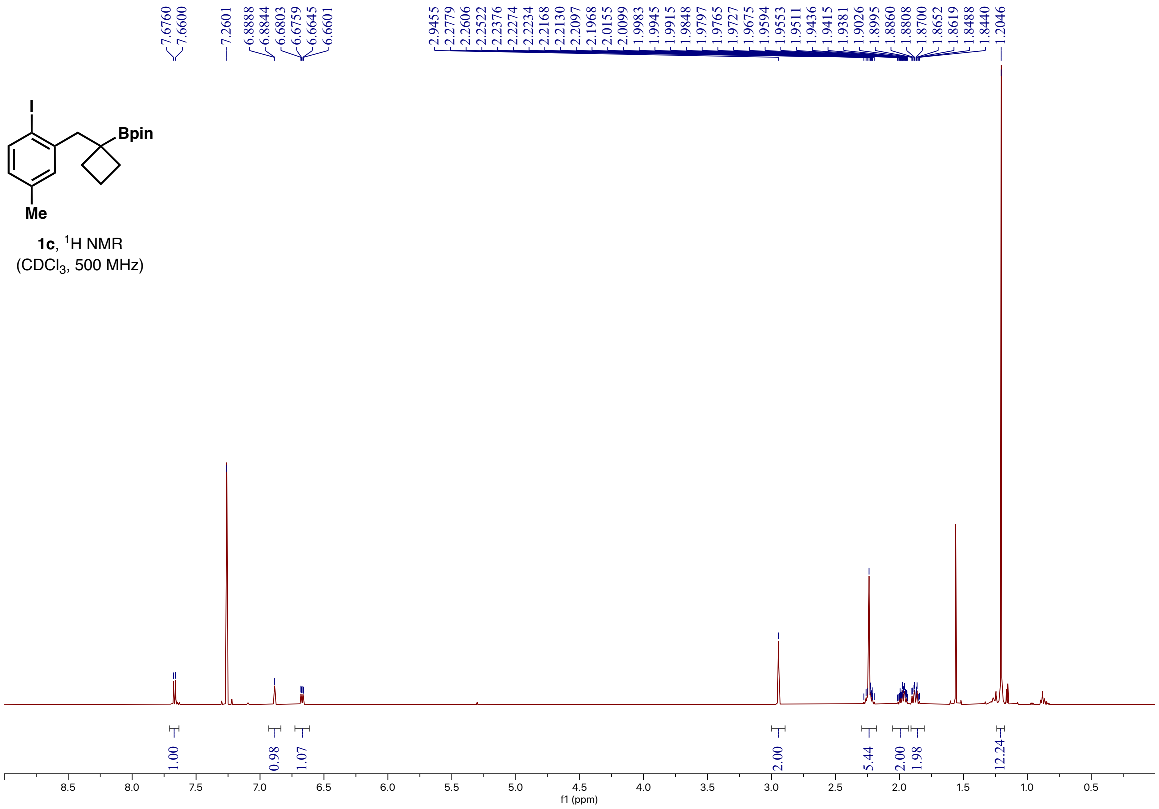

1c, ^{1}H NMR
($CDCl_3$, 500 MHz)

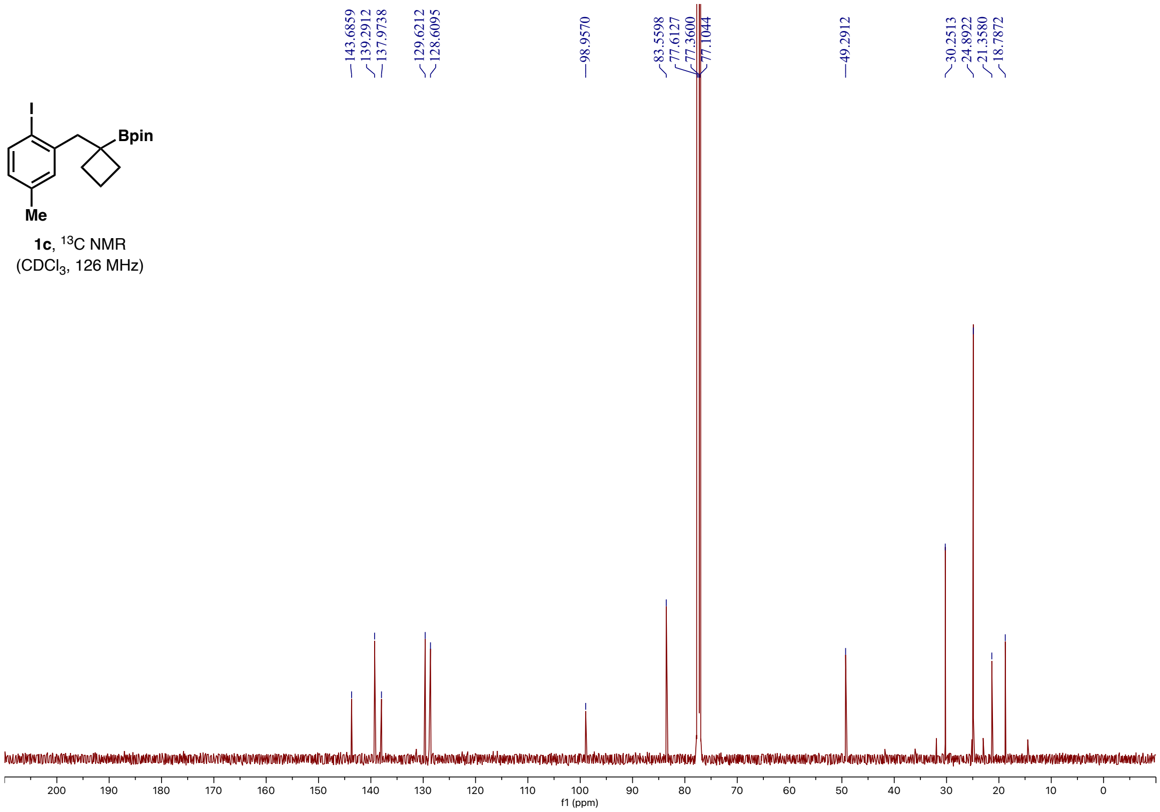

1c, ^{13}C NMR
($CDCl_3$, 126 MHz)

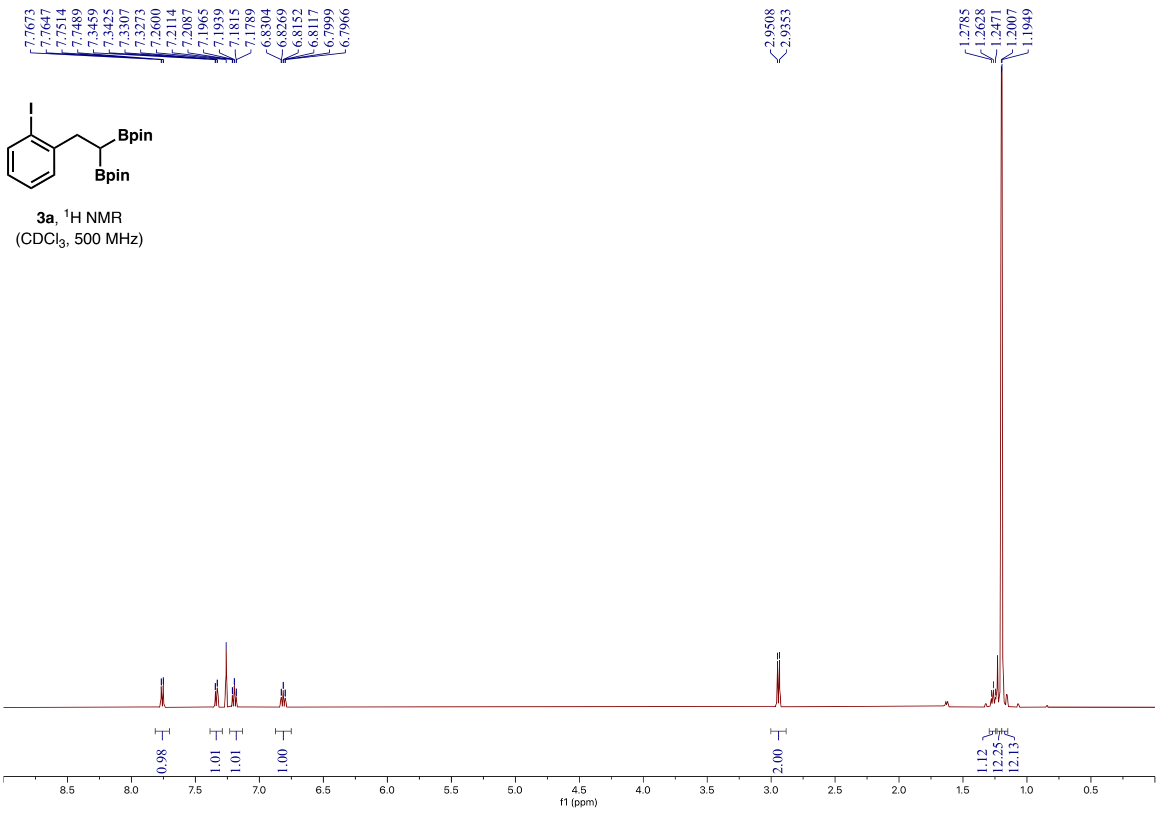

3a, ^{1}H NMR
($CDCl_3$, 500 MHz)

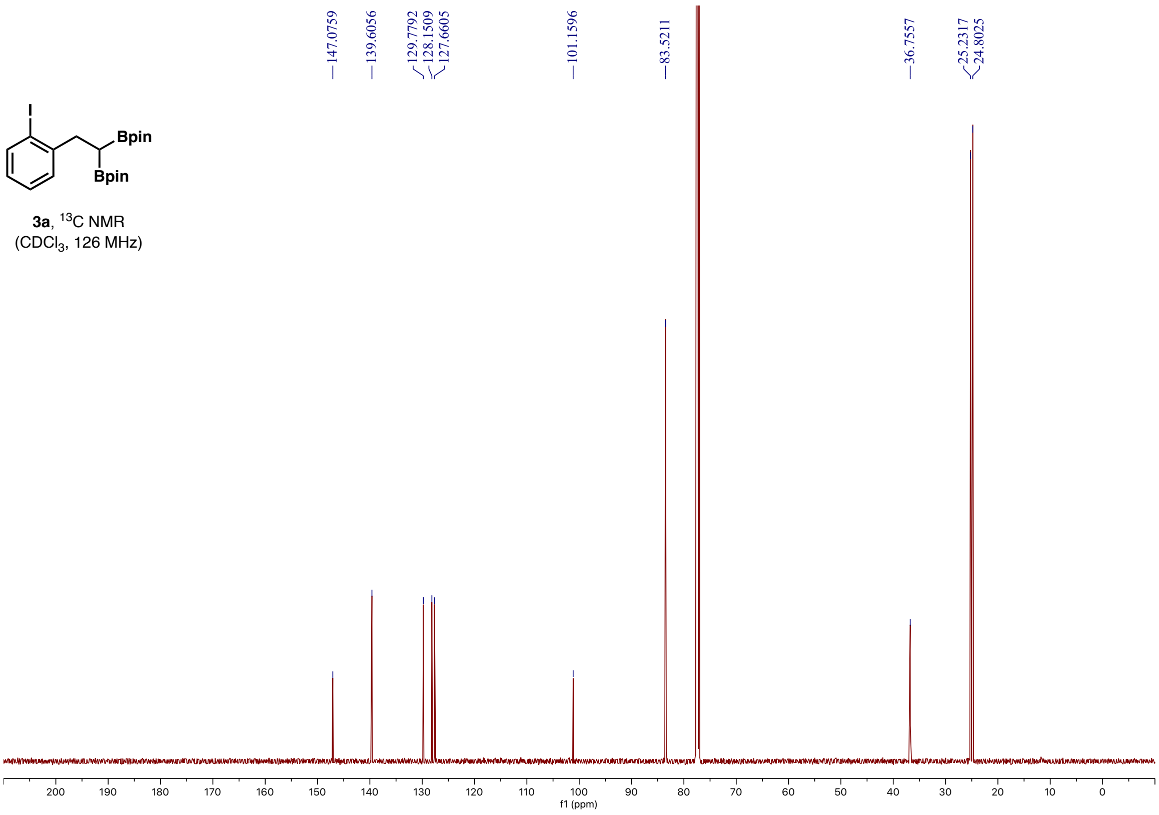

3a, ^{13}C NMR
($CDCl_3$, 126 MHz)

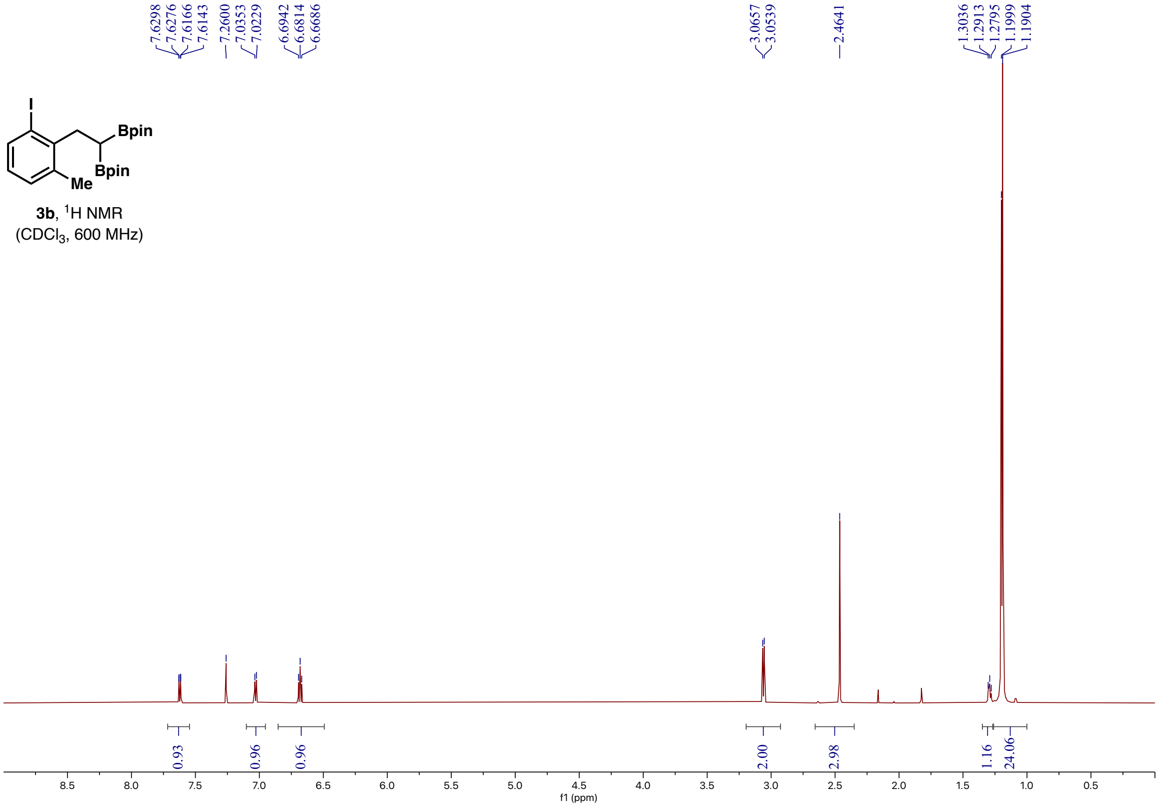

3b, ^{1}H NMR
($CDCl_3$, 600 MHz)

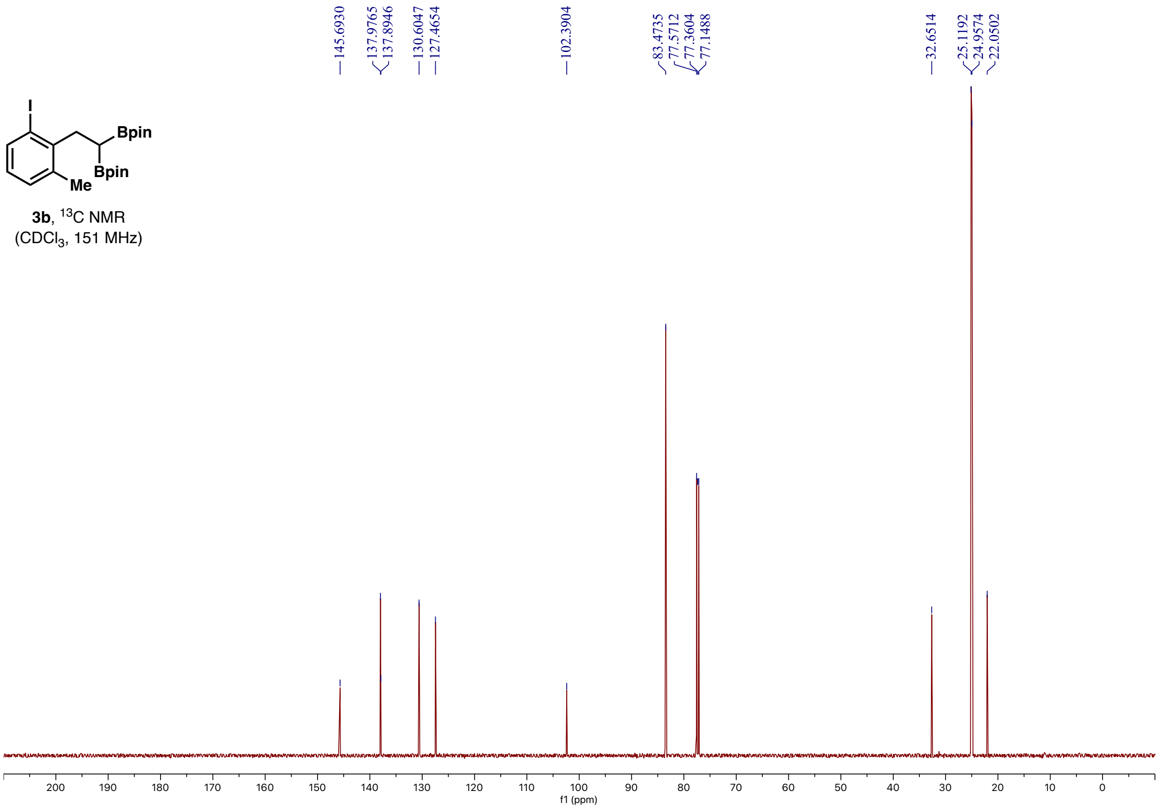

3b, ^{13}C NMR
($CDCl_3$, 151 MHz)

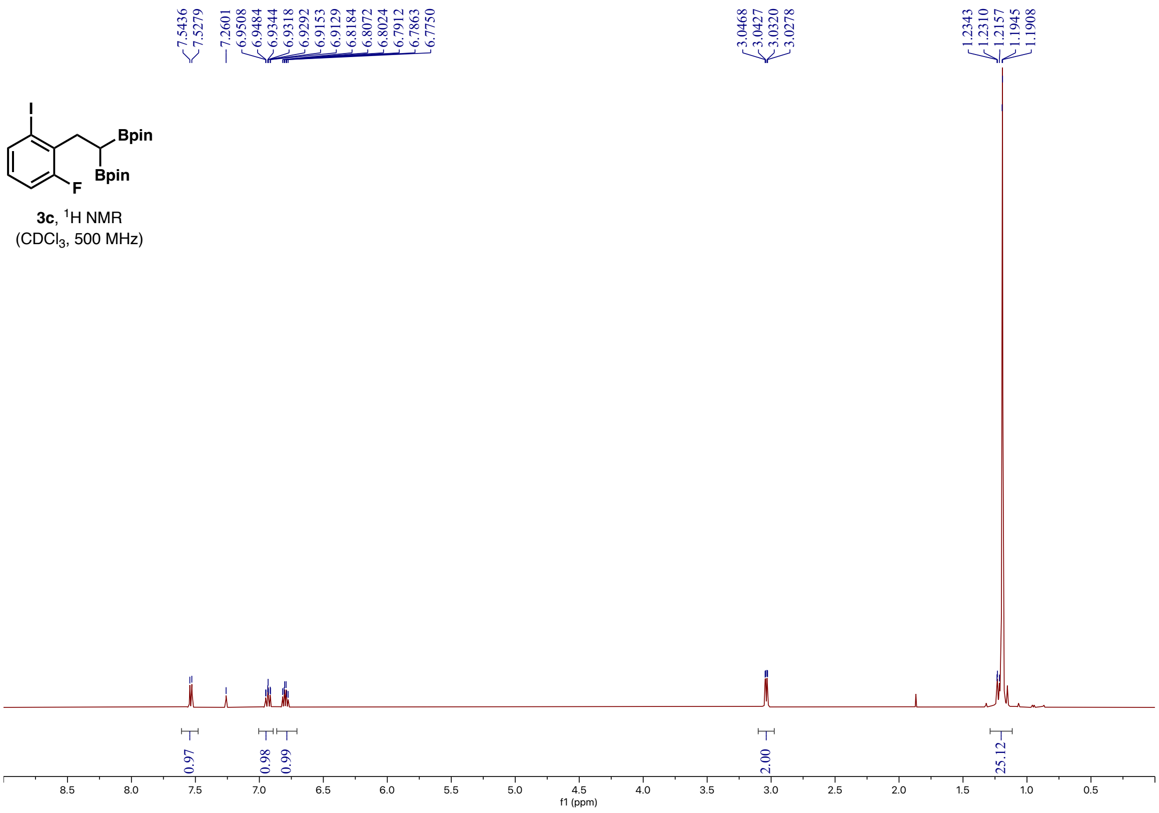

3c, ^{1}H NMR (CDCl$_3$, 500 MHz)

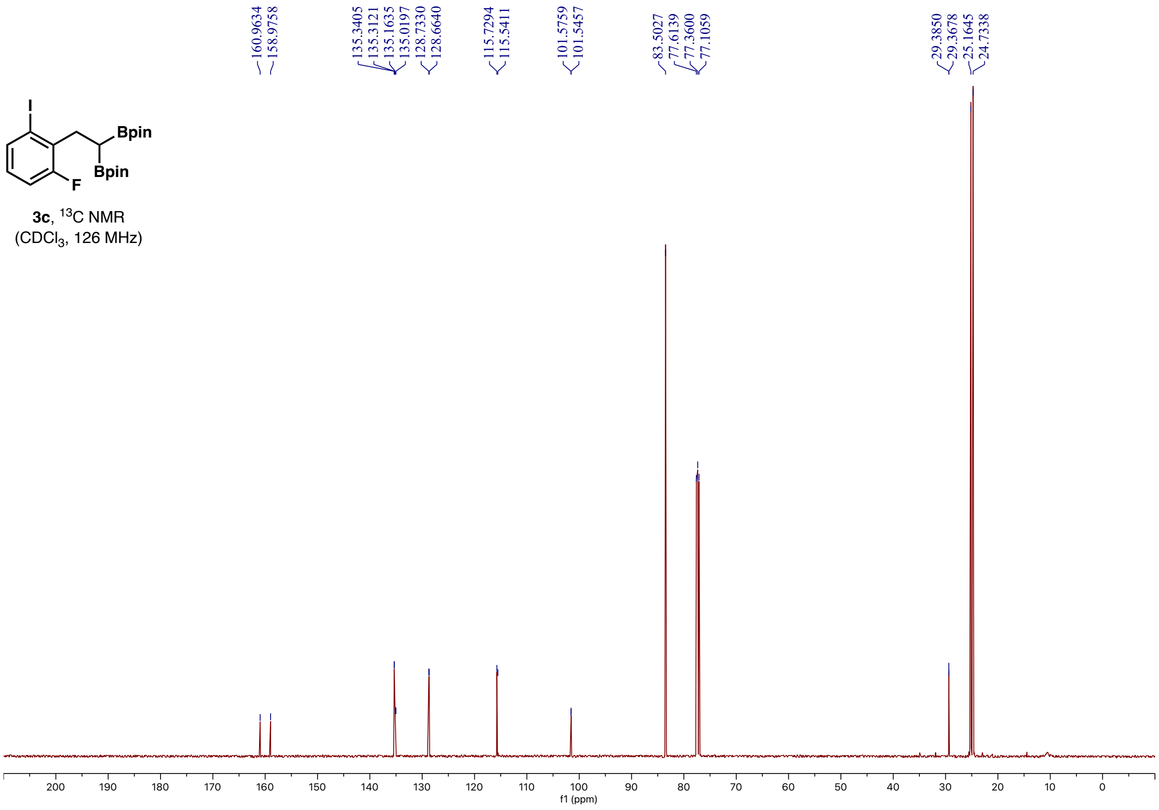

3c, ^{13}C NMR (CDCl$_3$, 126 MHz)

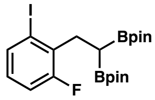

3c, ^{19}F NMR
($CDCl_3$, 565 MHz)

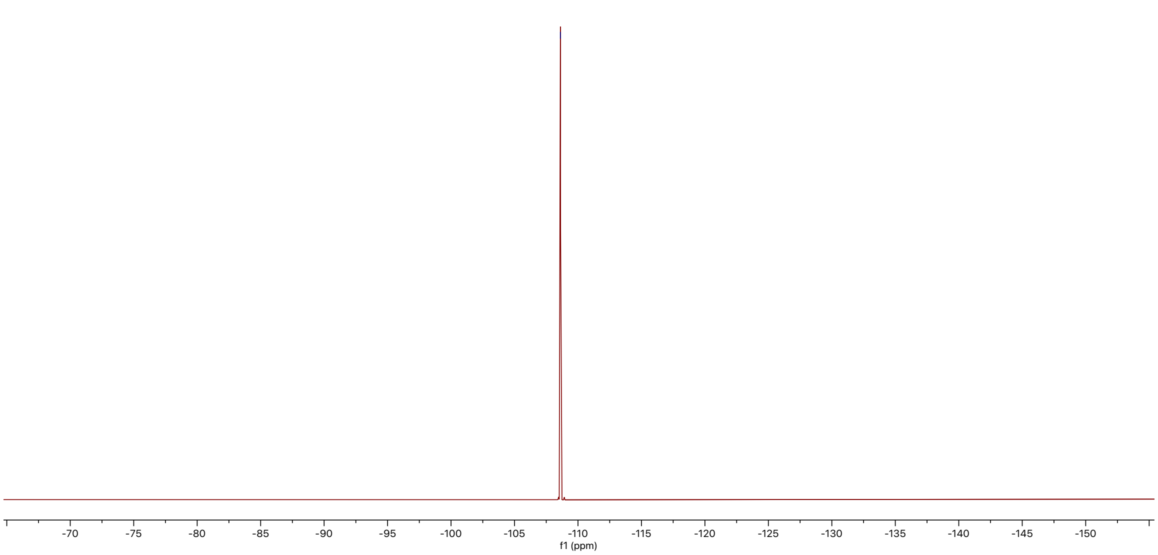

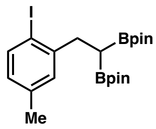

3d, ^{1}H NMR
($CDCl_3$, 600 MHz)

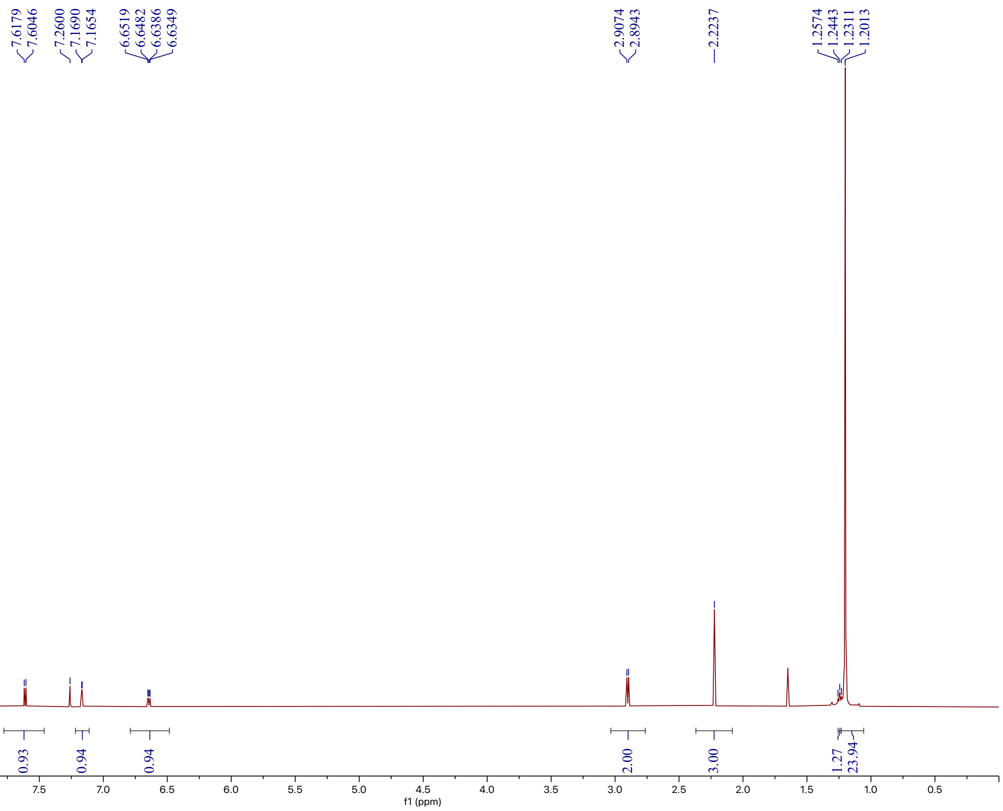

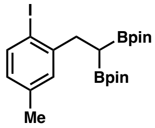

3d, ^{13}C NMR
($CDCl_3$, 151 MHz)

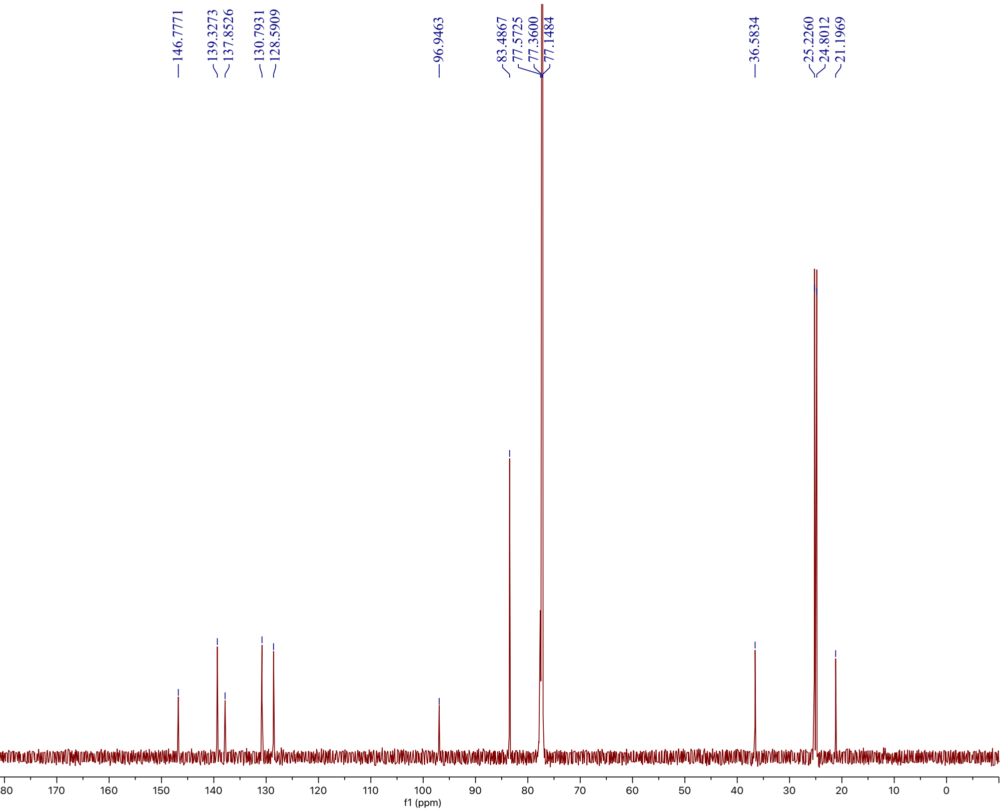

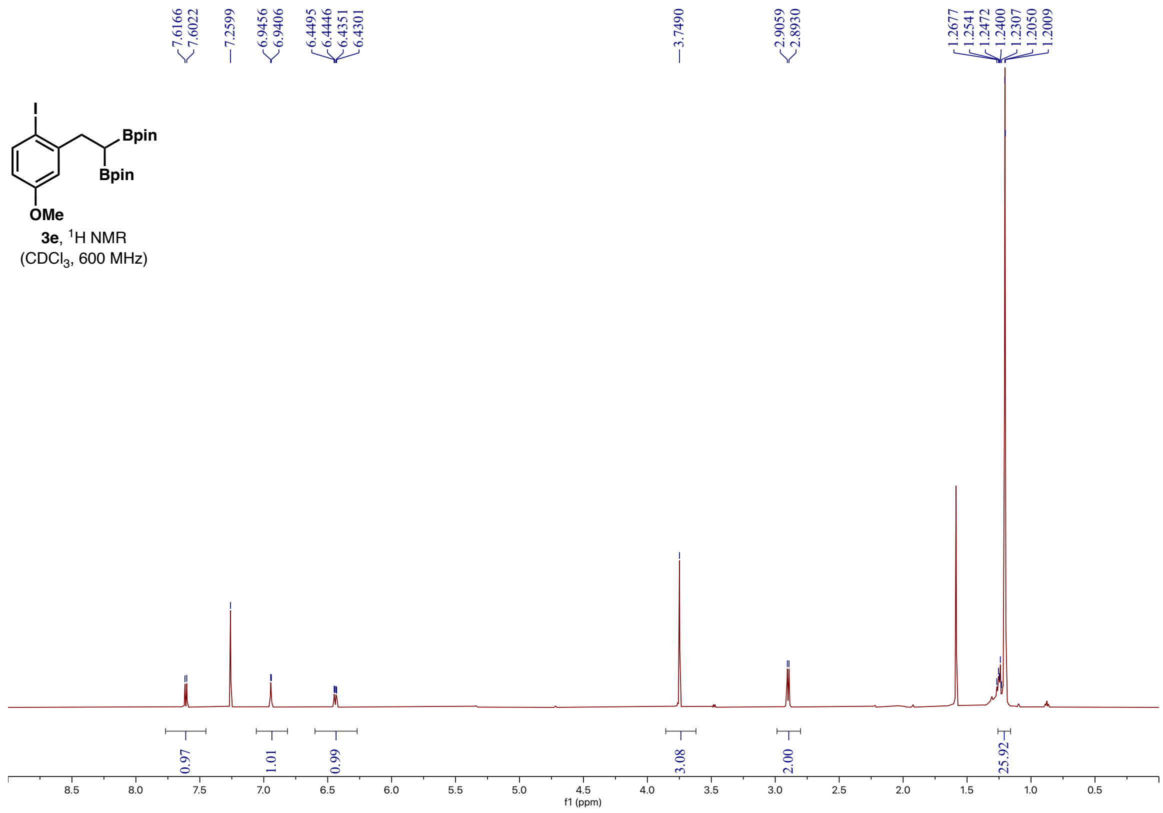
3e, ¹H NMR
(CDCl3, 600 MHz)
Bpin
Bpin
OMe
I
7.6166
7.6022
7.2599
6.9456
6.9406
6.4495
6.4446
6.4351
6.4301
3.7490
2.9059
2.8930
1.2677
1.2541
1.2472
1.2400
1.2307
1.2050
1.2009
0.97
1.01
0.99
3.08
2.00
25.92
f1 (ppm)

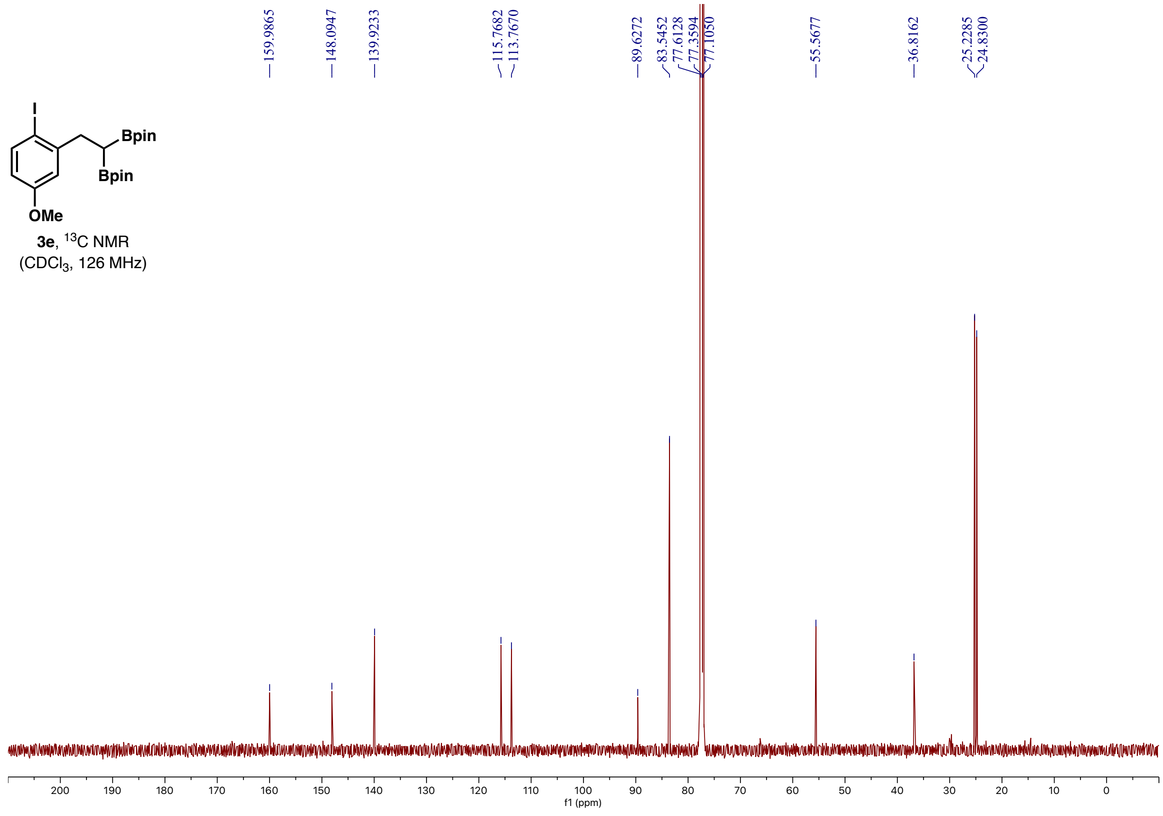
3e, ¹³C NMR
(CDCl3, 126 MHz)
Bpin
Bpin
OMe
I
159.9865
148.0947
139.9233
115.7682
113.7670
89.6272
83.5452
77.6128
77.3594
77.1050
55.5677
36.8162
25.2285
24.8300
f1 (ppm)

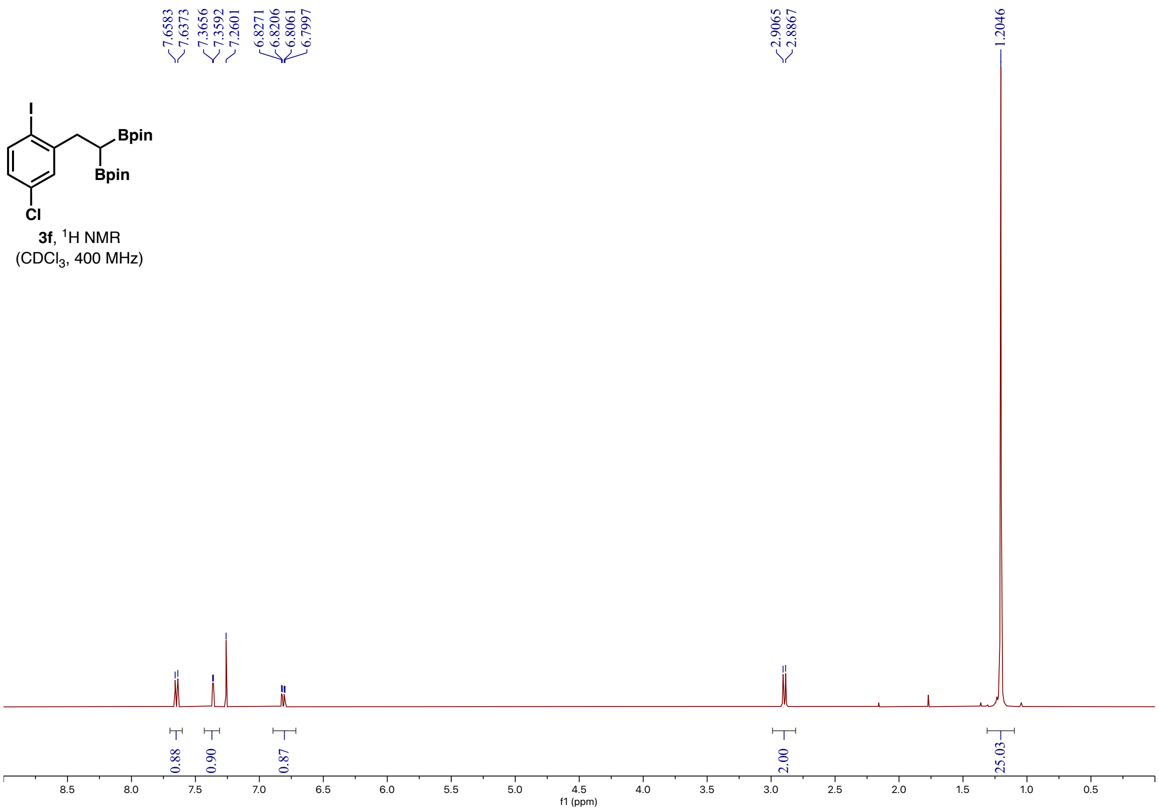
7.6583
7.6373
7.3656
7.3592
7.2601
6.8271
6.8206
6.8061
6.7997
2.9065
2.8867
1.2046
Bpin
Bpin
I
Cl
3f, 1H NMR
(CDCl3, 400 MHz)
0.88
0.90
0.87
2.00
25.03
f1 (ppm)

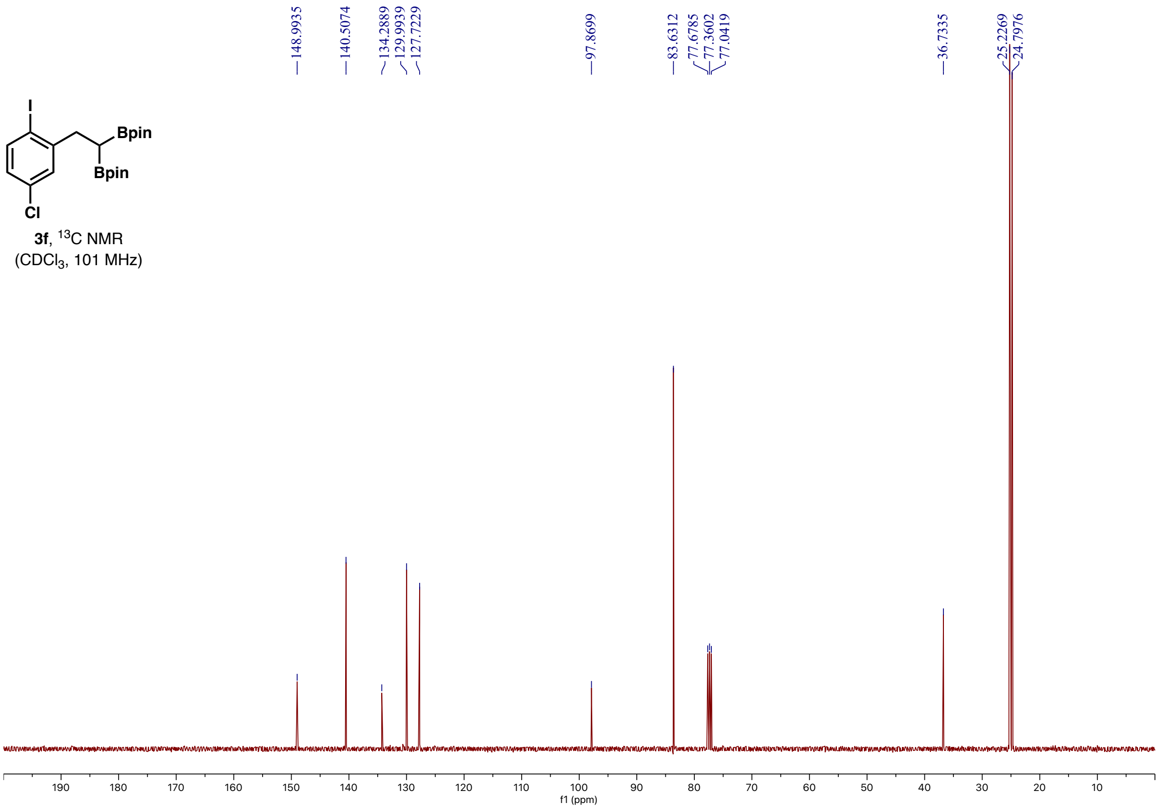
148.9935
140.5074
134.2889
129.9939
127.7229
97.8699
83.6312
77.6785
77.3602
77.0419
36.7335
25.2269
24.7976
Bpin
Bpin
I
Cl
3f, 13C NMR
(CDCl3, 101 MHz)
f1 (ppm)

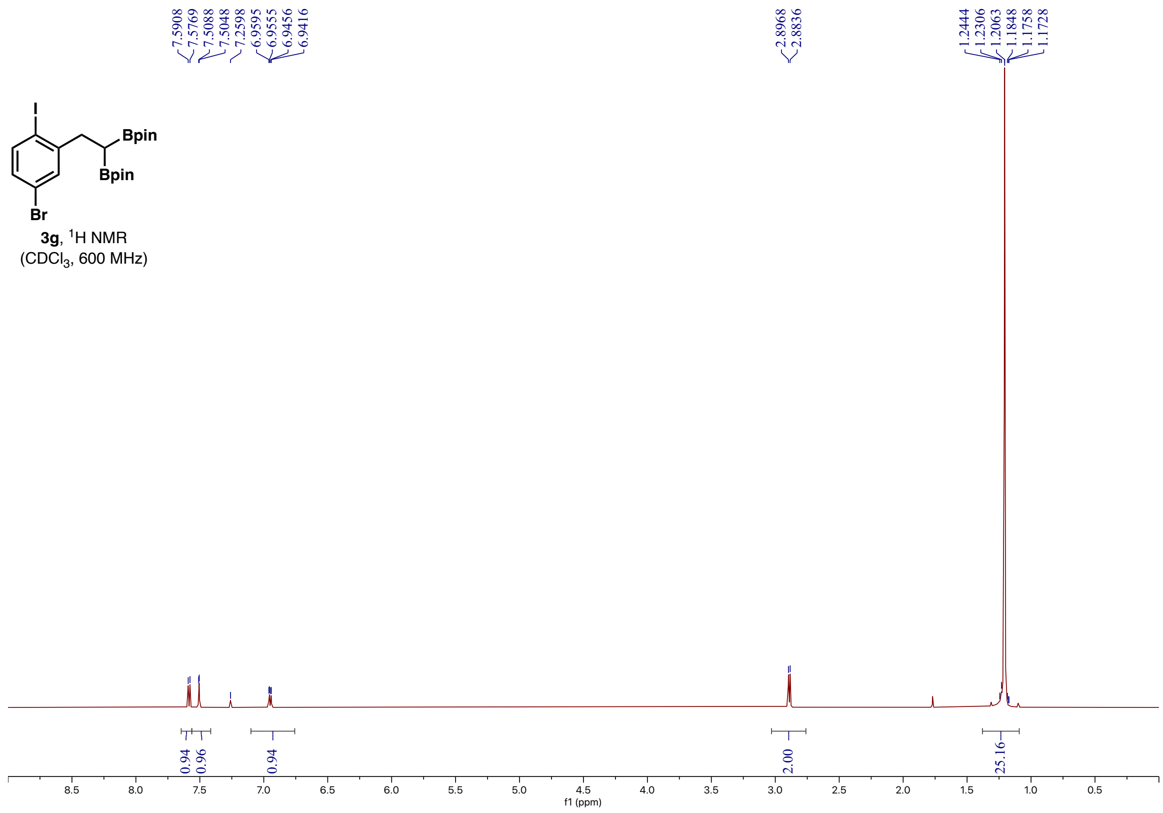

3g, 1H NMR
(CDCl3, 600 MHz)
7.5908
7.5769
7.5088
7.5048
7.2598
6.9595
6.9555
6.9456
6.9416
2.8968
2.8836
1.2444
1.2306
1.2063
1.1848
1.1758
1.1728
0.94
0.96
0.94
2.00
25.16
f1 (ppm)

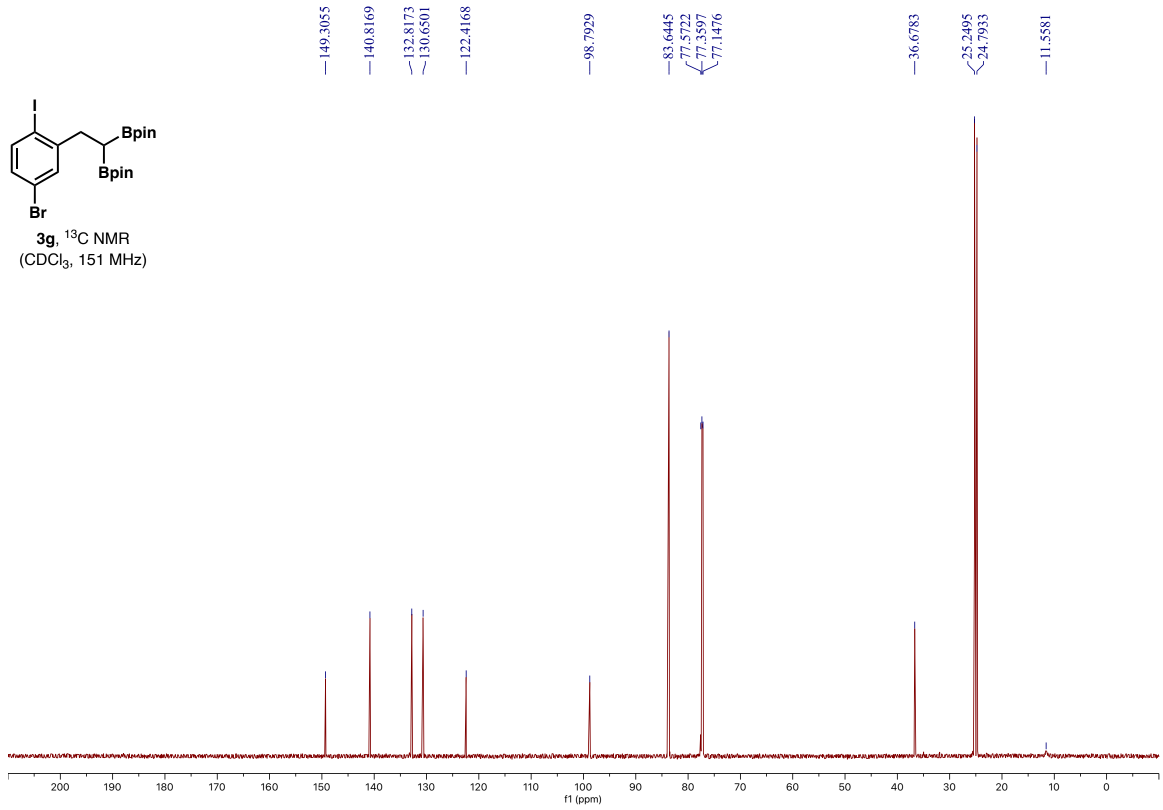

3g, 13C NMR
(CDCl3, 151 MHz)
149.3055
140.8169
132.8173
130.6501
122.4168
98.7929
83.6445
77.5722
77.3597
77.1476
36.6783
25.2495
24.7933
11.5581
f1 (ppm)

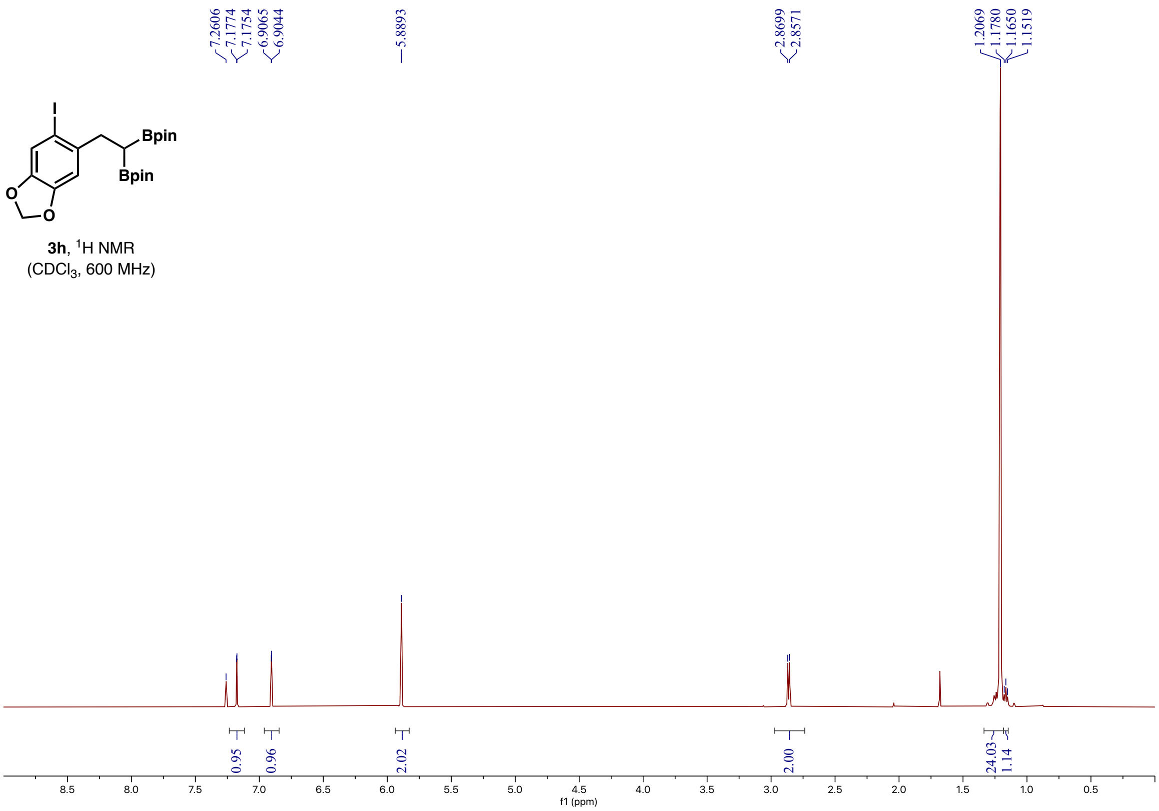
I
Bpin
Bpin
O
O
3h, 1H NMR
(CDCl3, 600 MHz)
7.2606
7.1774
7.1754
6.9065
6.9044
5.8893
2.8699
2.8571
1.2069
1.1780
1.1650
1.1519
0.95
0.96
2.02
2.00
24.03
1.14
f1 (ppm)

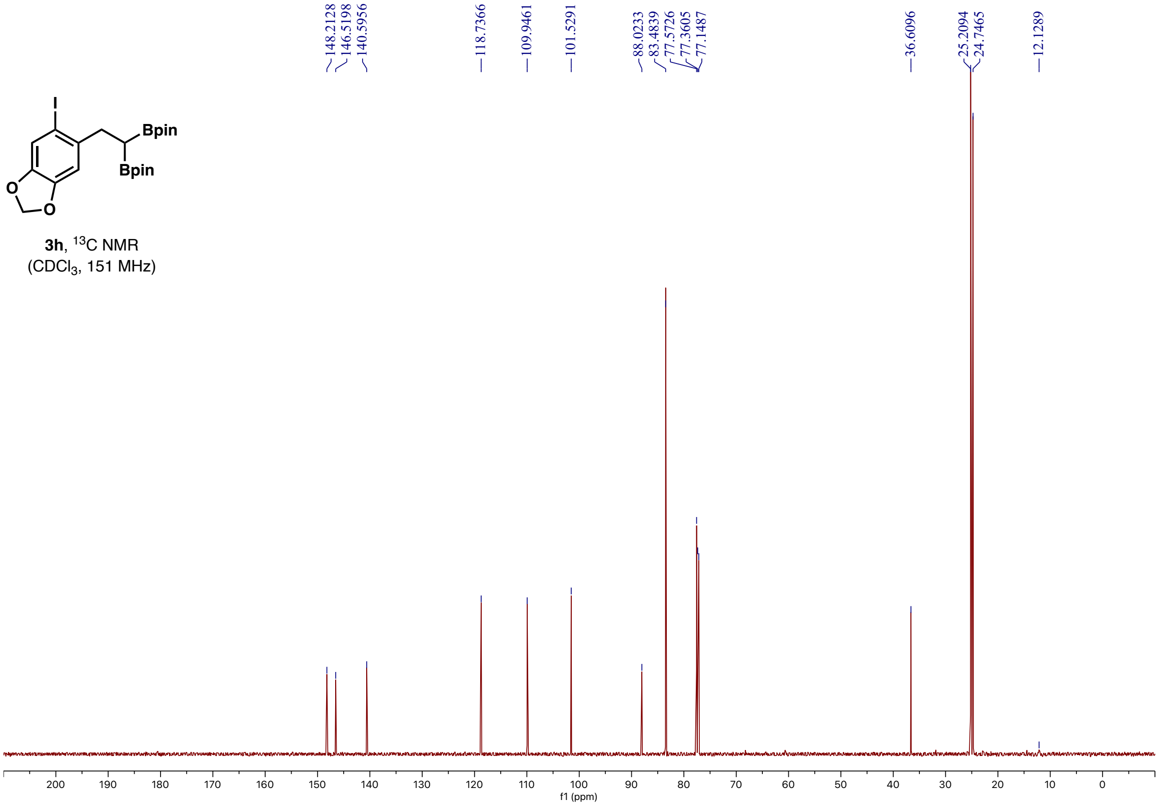
I
Bpin
Bpin
O
O
3h, 13C NMR
(CDCl3, 151 MHz)
148.2128
146.5198
140.5956
118.7366
109.9461
101.5291
88.0233
83.4839
77.5726
77.3605
77.1487
36.6096
25.2094
24.7465
12.1289
f1 (ppm)

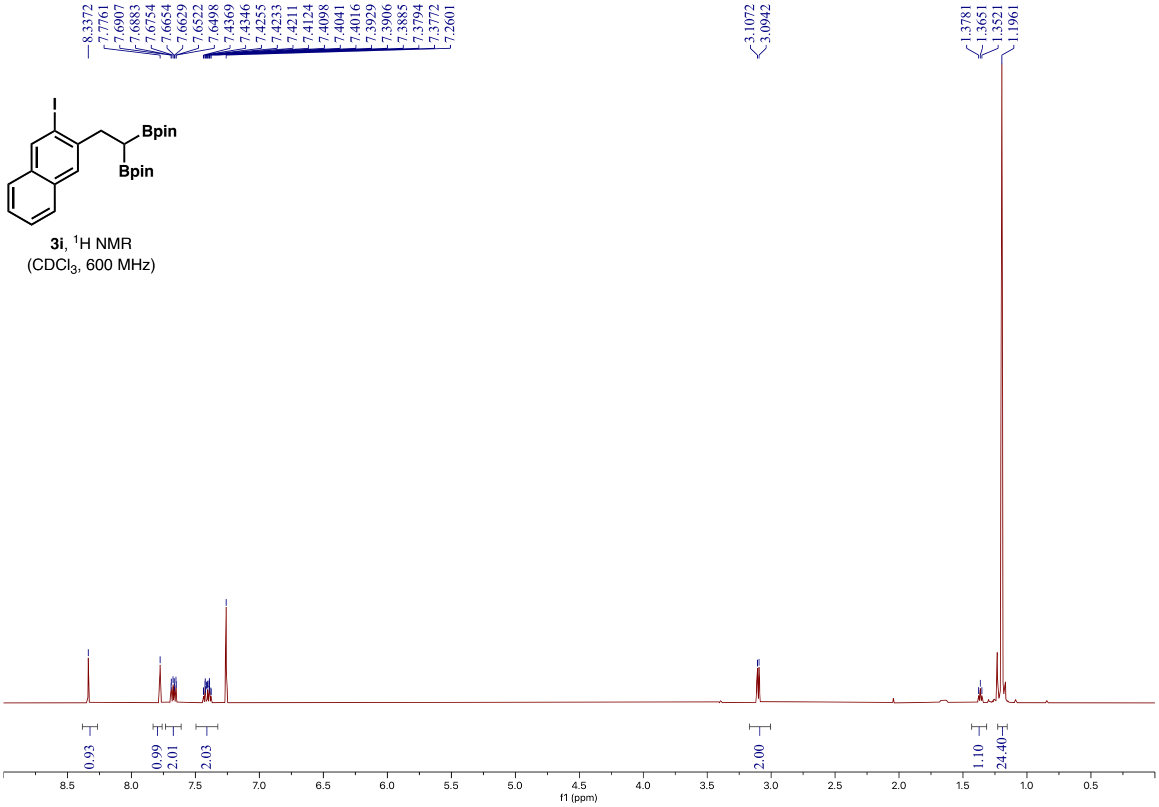

3i, ^{1}H NMR
($CDCl_3$, 600 MHz)

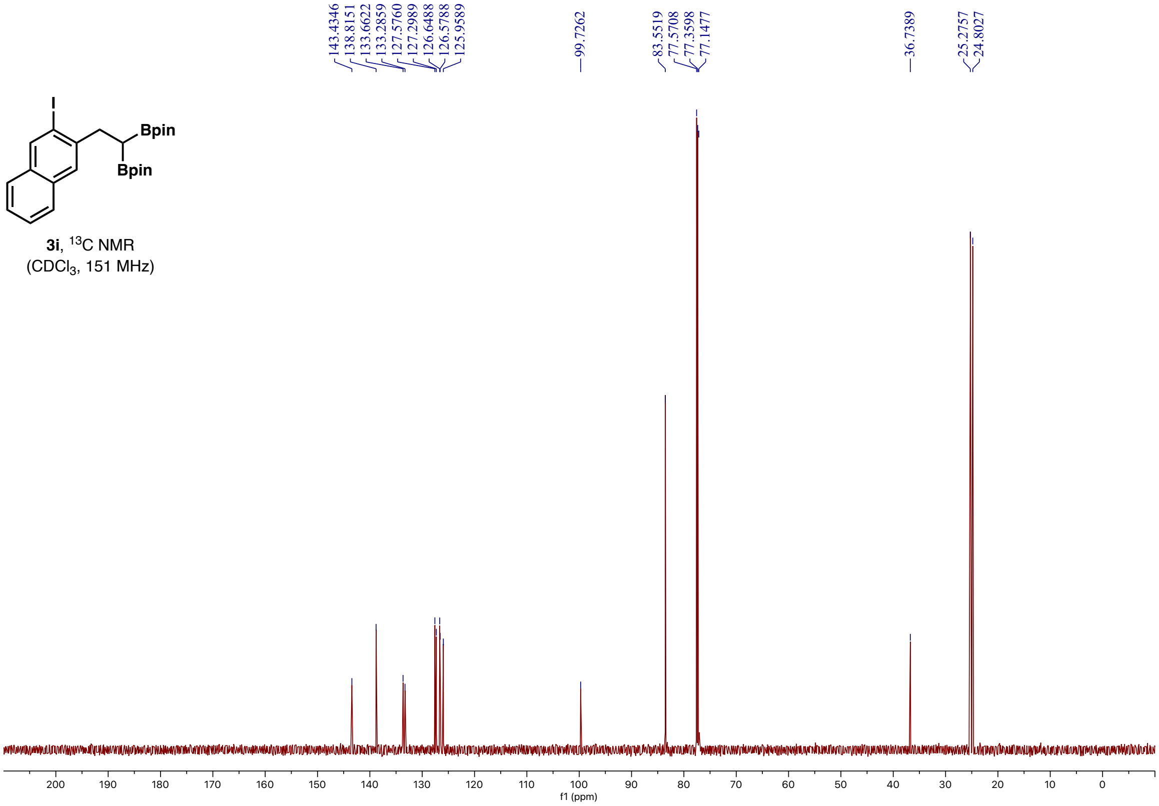

3i, ^{13}C NMR
($CDCl_3$, 151 MHz)

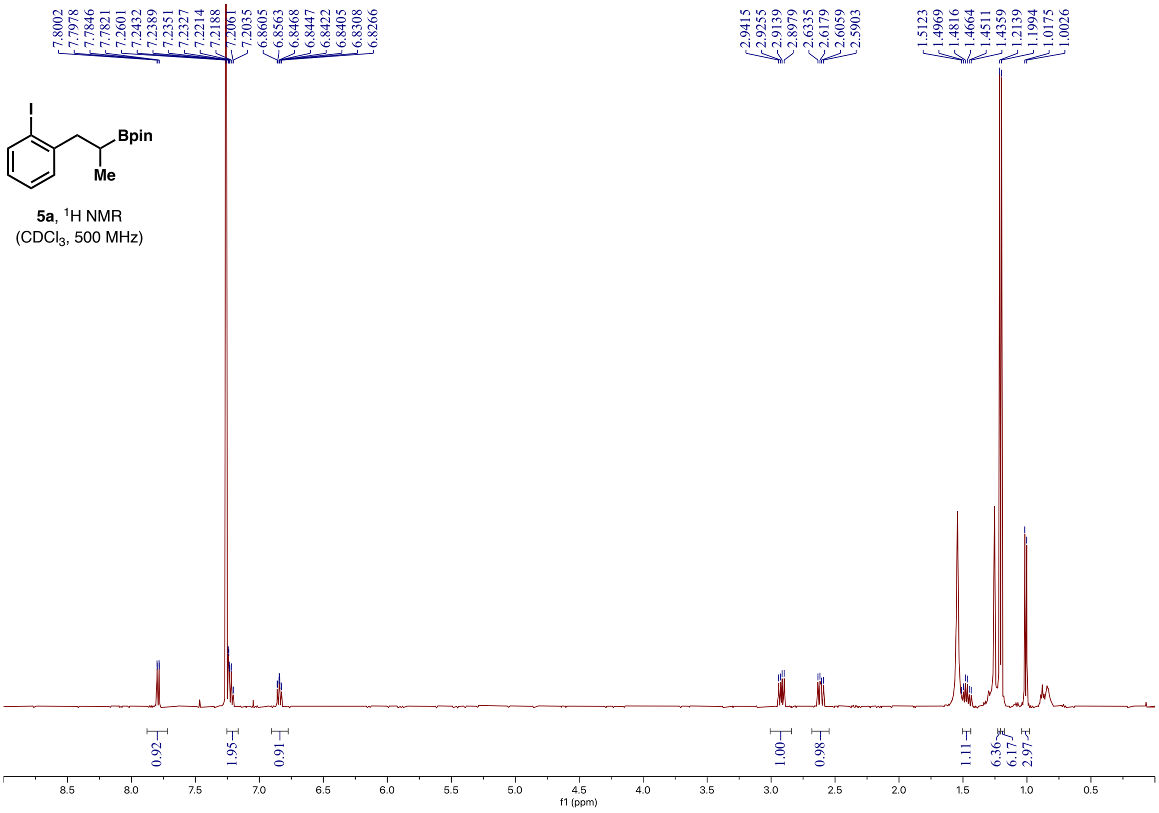
7.8002
7.7978
7.7846
7.7821
7.2601
7.2432
7.2389
7.2351
7.2327
7.2214
7.2188
7.2061
7.2035
6.8605
6.8563
6.8468
6.8447
6.8422
6.8405
6.8308
6.8266
2.9415
2.9255
2.9139
2.8979
2.6335
2.6179
2.6059
2.5903
1.5123
1.4969
1.4816
1.4664
1.4511
1.4359
1.2139
1.1994
1.0175
1.0026
I
Bpin
Me
5a, 1H NMR
(CDCl3, 500 MHz)
0.92
1.95
0.91
1.00
0.98
1.11
6.36
6.17
2.97
f1 (ppm)

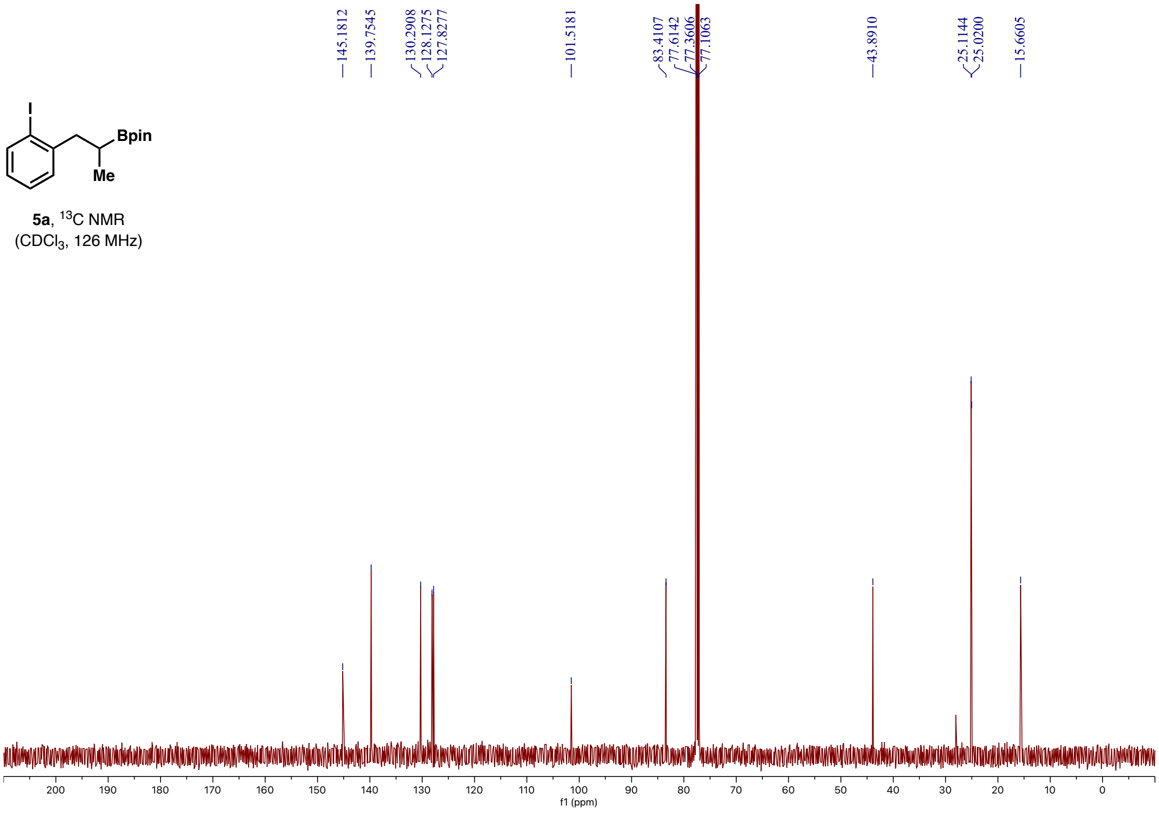
145.1812
139.7545
130.2908
128.1275
127.8277
101.5181
83.4107
77.6142
77.3606
77.1063
43.8910
25.1144
25.0200
15.6605
I
Bpin
Me
5a, 13C NMR
(CDCl3, 126 MHz)
f1 (ppm)

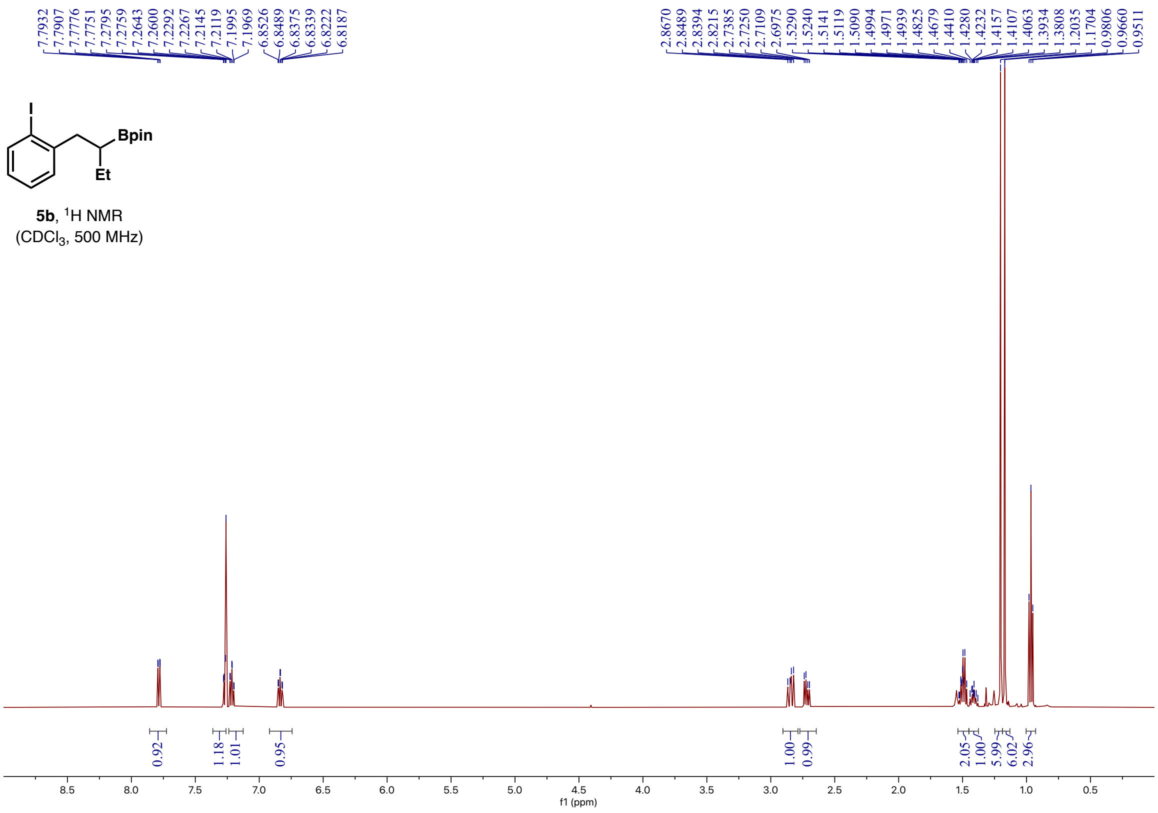
7.7932
7.7907
7.7776
7.7751
7.2795
7.2759
7.2643
7.2600
7.2292
7.2267
7.2145
7.2119
7.1995
7.1969
6.8526
6.8489
6.8375
6.8339
6.8222
6.8187
2.8670
2.8489
2.8394
2.8215
2.7385
2.7250
2.7109
2.6975
1.5290
1.5240
1.5141
1.5119
1.5090
1.4994
1.4971
1.4939
1.4825
1.4679
1.4410
1.4280
1.4232
1.4157
1.4107
1.4063
1.3934
1.3808
1.2035
1.1704
0.9806
0.9660
0.9511
Bpin
Et
5b, 1H NMR
(CDCl3, 500 MHz)
0.92
1.18
1.01
0.95
1.00
0.99
2.05
1.00
5.99
6.02
2.96
f1 (ppm)

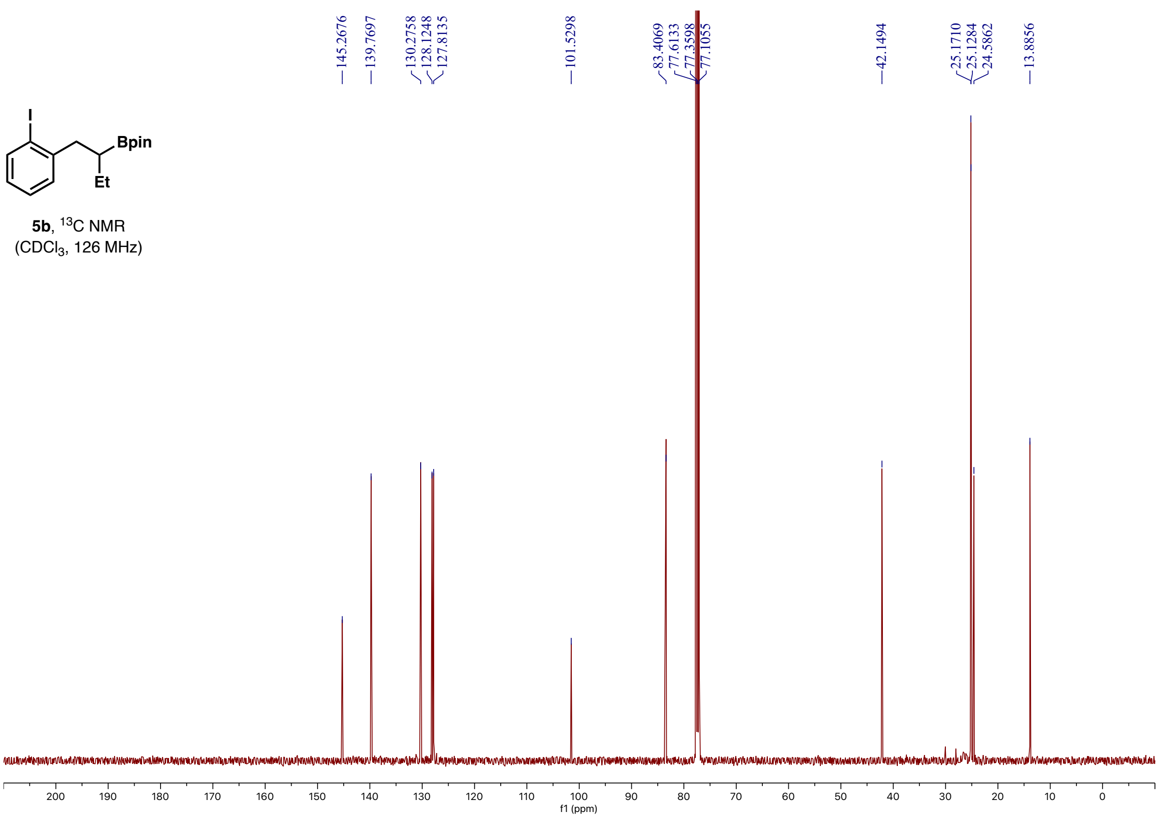
145.2676
139.7697
130.2758
128.1248
127.8135
101.5298
83.4069
77.6133
77.3598
77.1055
42.1494
25.1710
25.1284
24.5862
13.8856
Bpin
Et
5b, 13C NMR
(CDCl3, 126 MHz)
f1 (ppm)

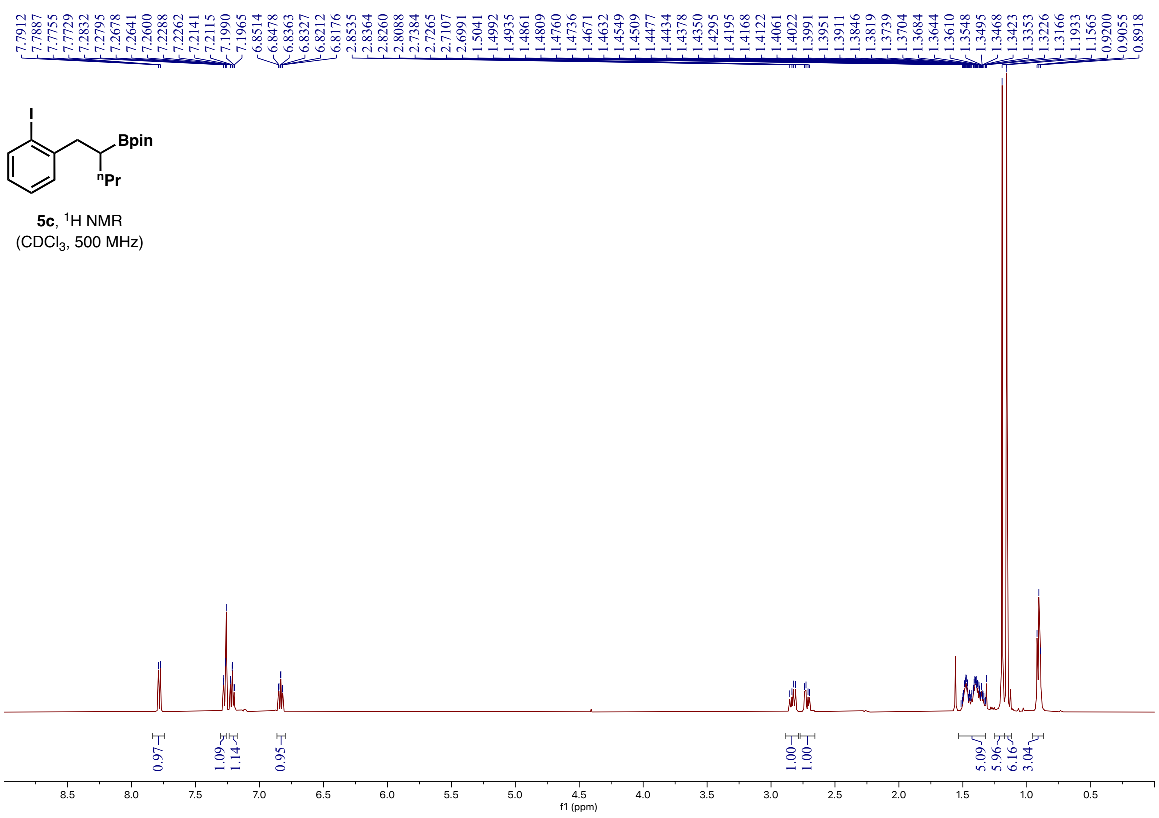

5c, ^{1}H NMR
($CDCl_3$, 500 MHz)

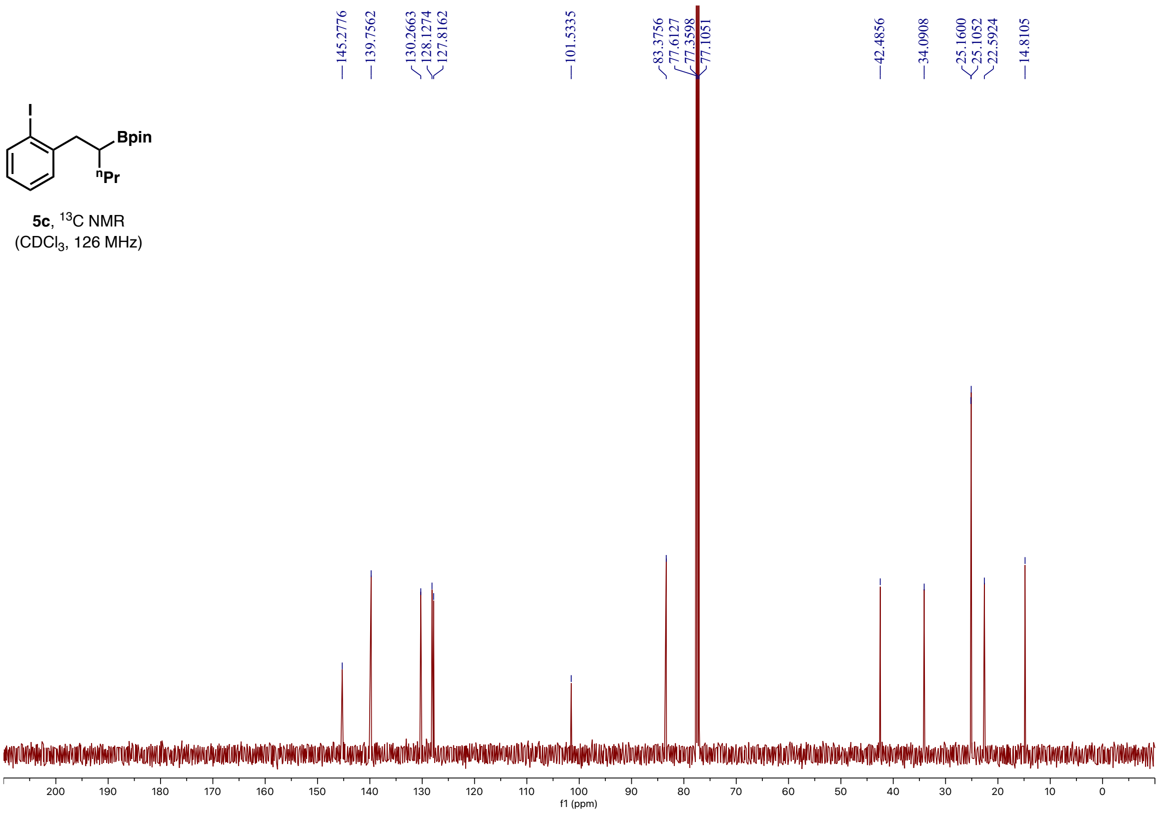

5c, ^{13}C NMR
($CDCl_3$, 126 MHz)

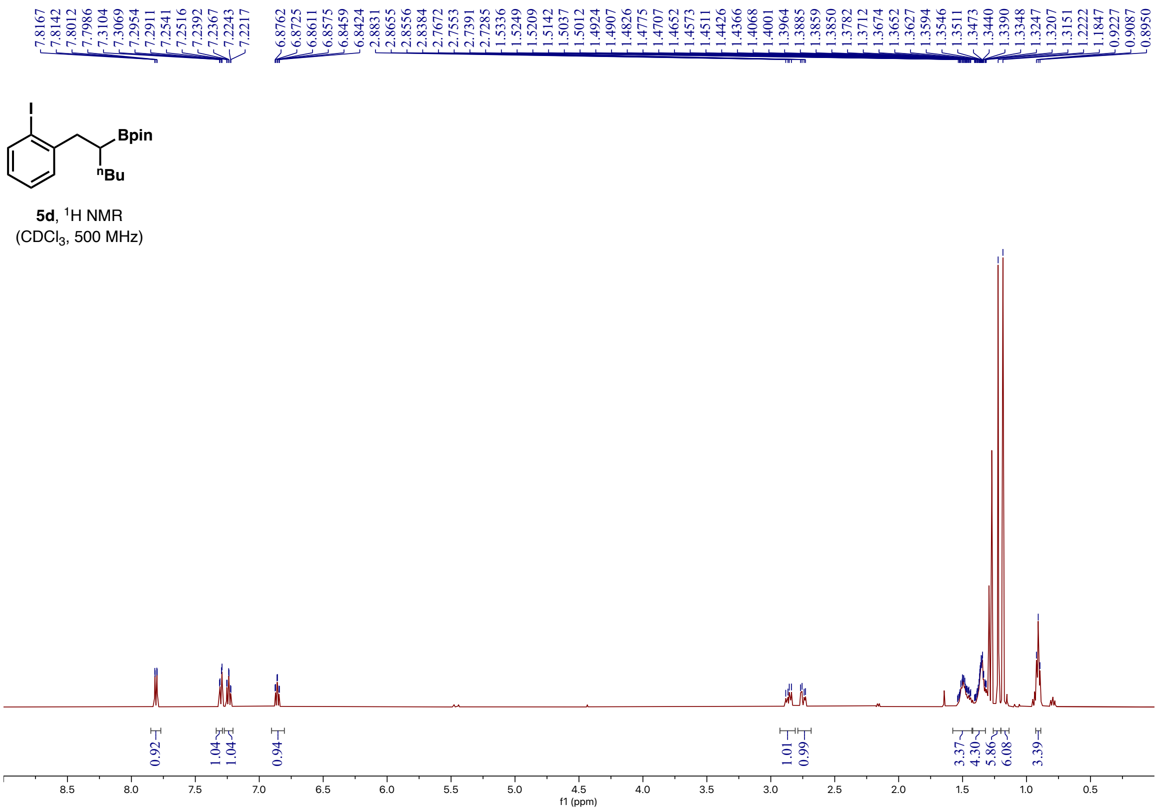

5d, ^{1}H NMR
($CDCl_3$, 500 MHz)

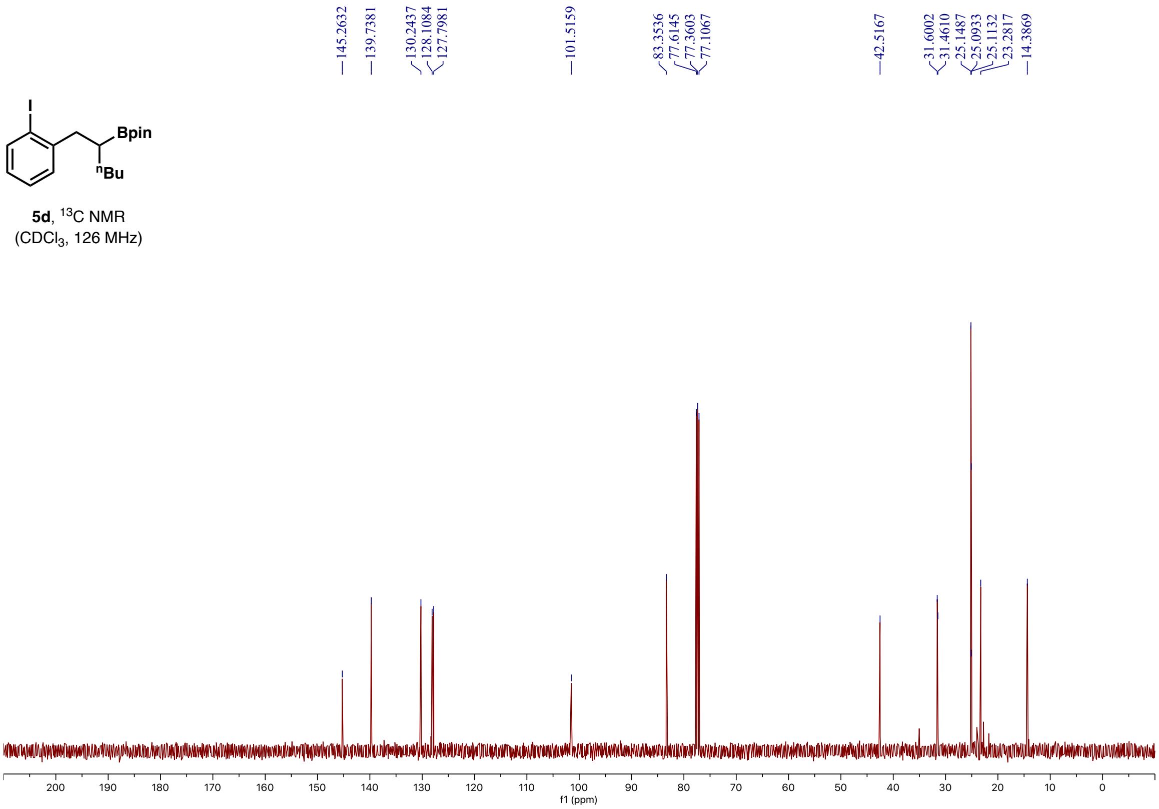

5d, ^{13}C NMR
($CDCl_3$, 126 MHz)

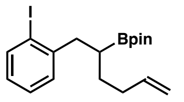

5e, ^{1}H NMR
($CDCl_3$, 400 MHz)

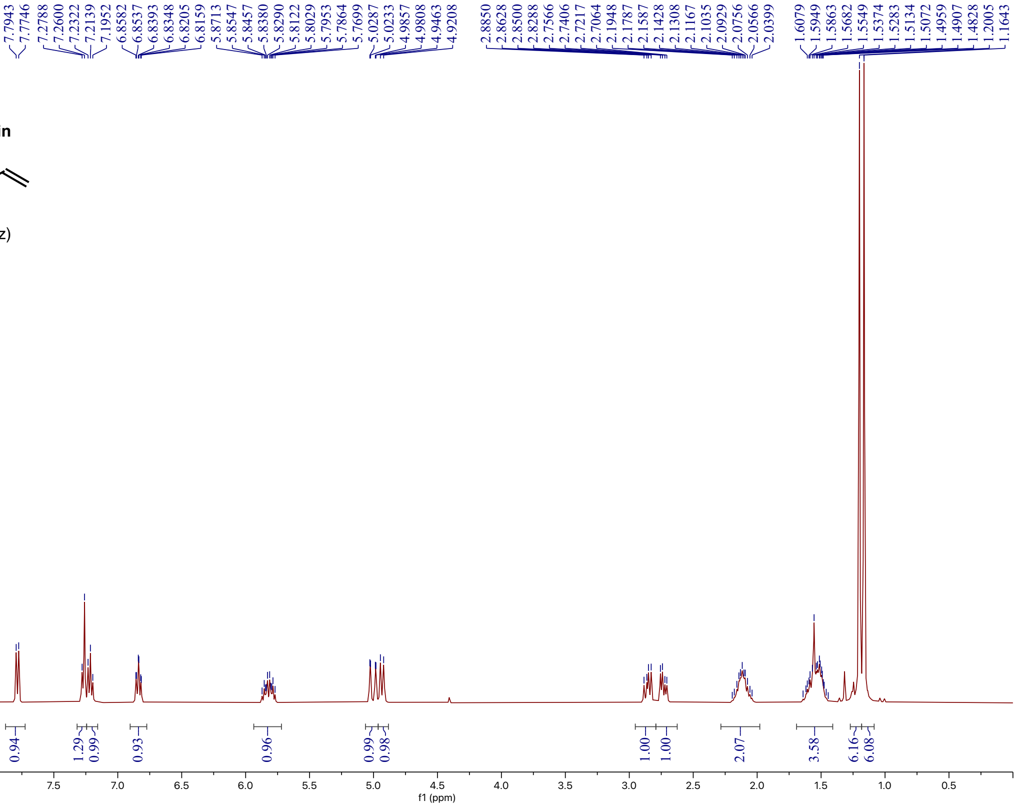

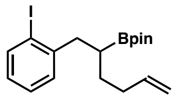

5e, ^{13}C NMR
($CDCl_3$, 101 MHz)

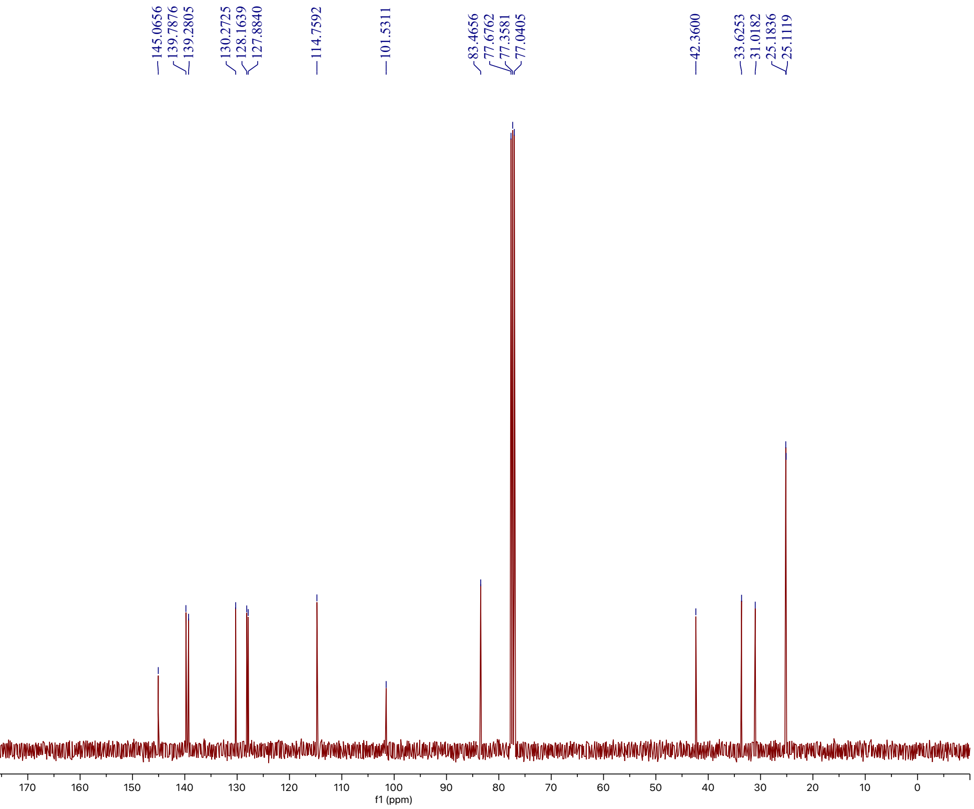

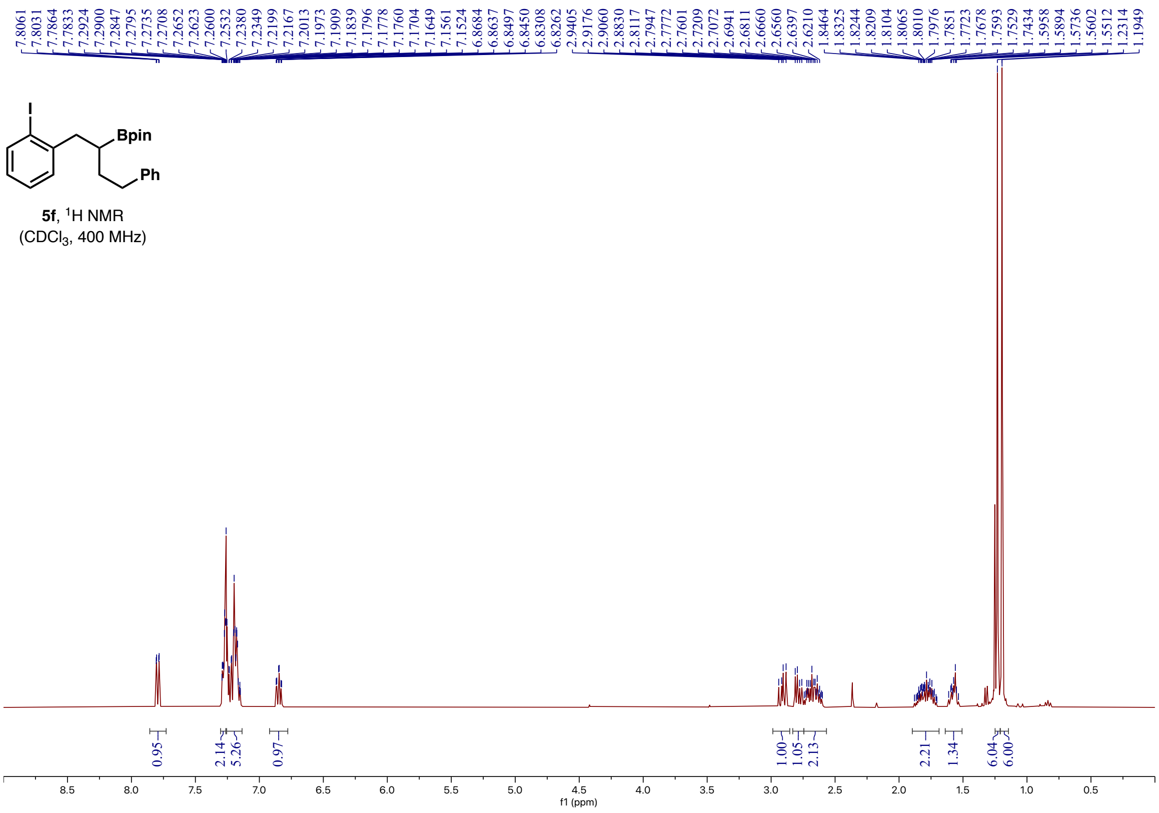

5f, ^{1}H NMR
($CDCl_3$, 400 MHz)

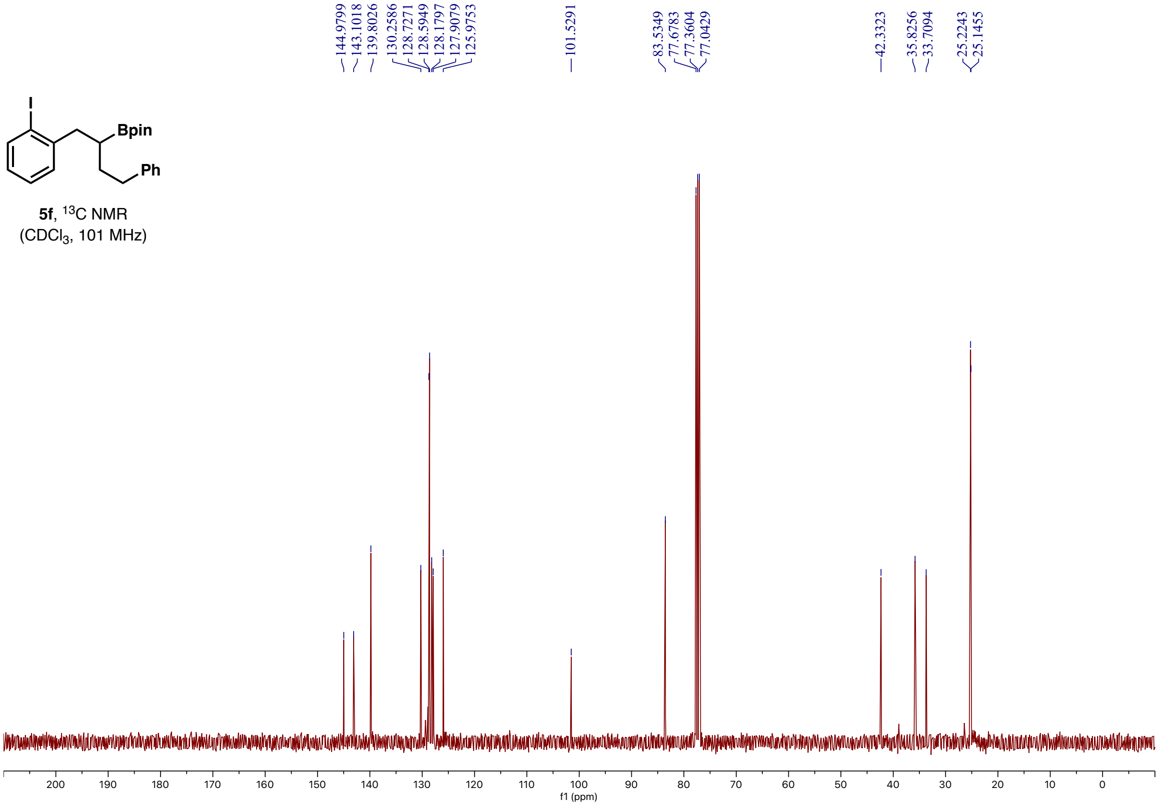

5f, ^{13}C NMR
($CDCl_3$, 101 MHz)

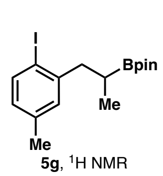

5g, ^{1}H NMR
($CDCl_3$, 500 MHz)

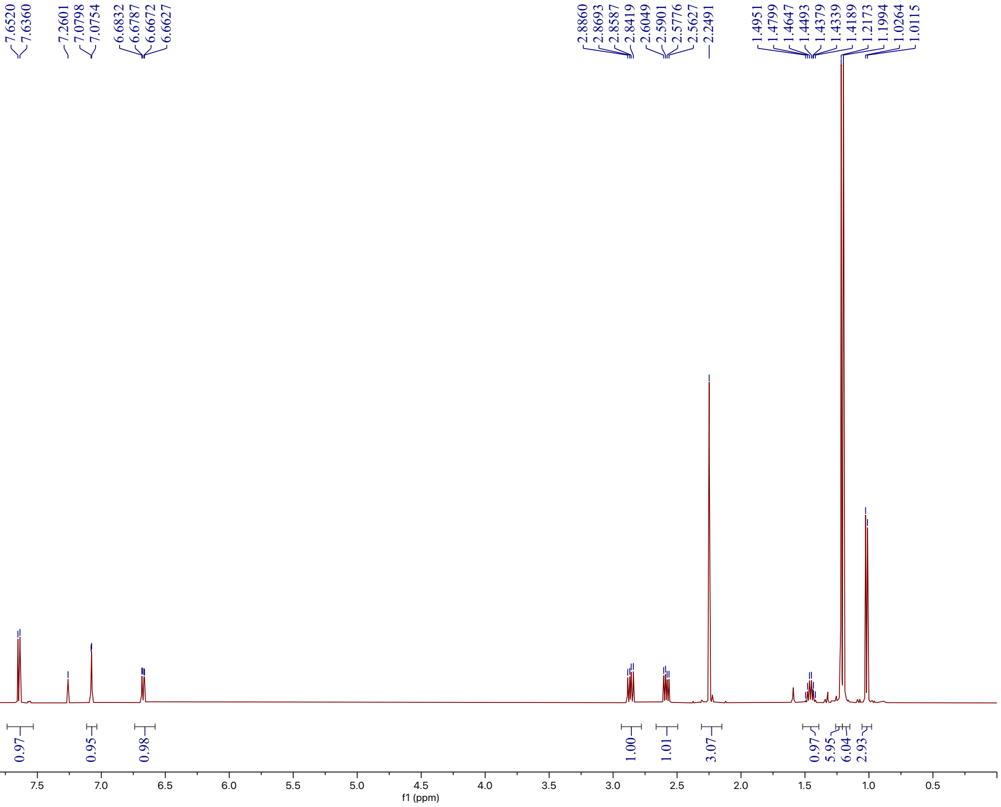

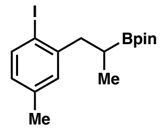

5g, ^{13}C NMR
($CDCl_3$, 126 MHz)

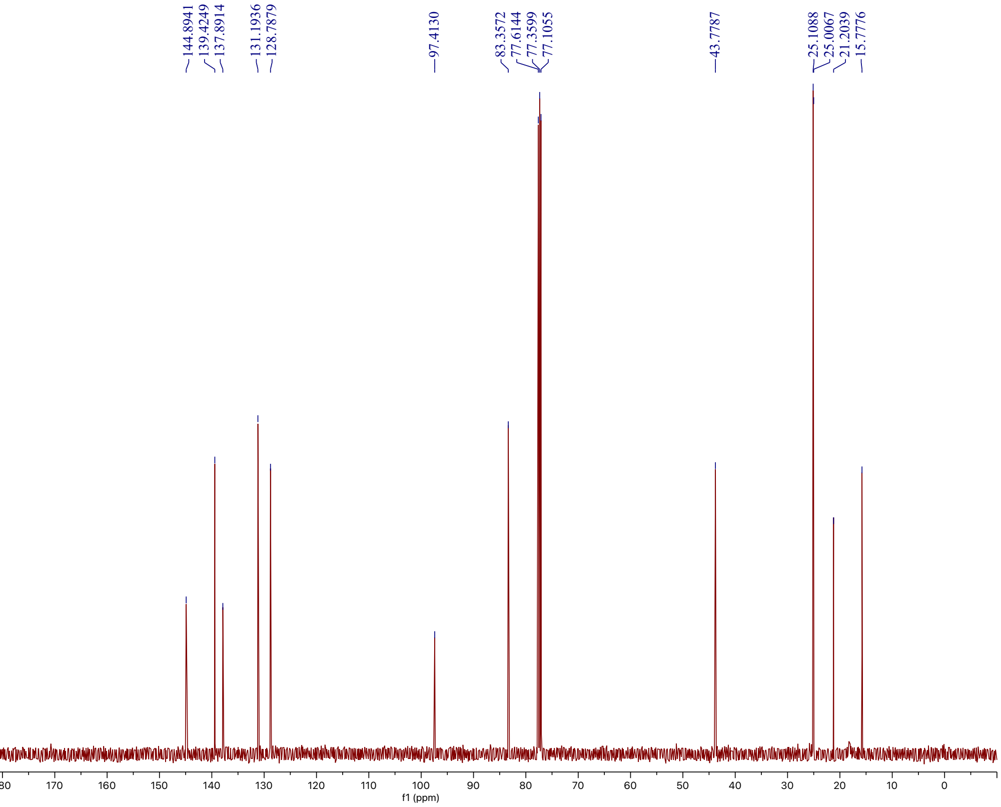

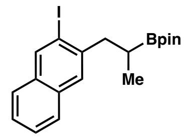

5h, ^{1}H NMR
($CDCl_3$, 500 MHz)

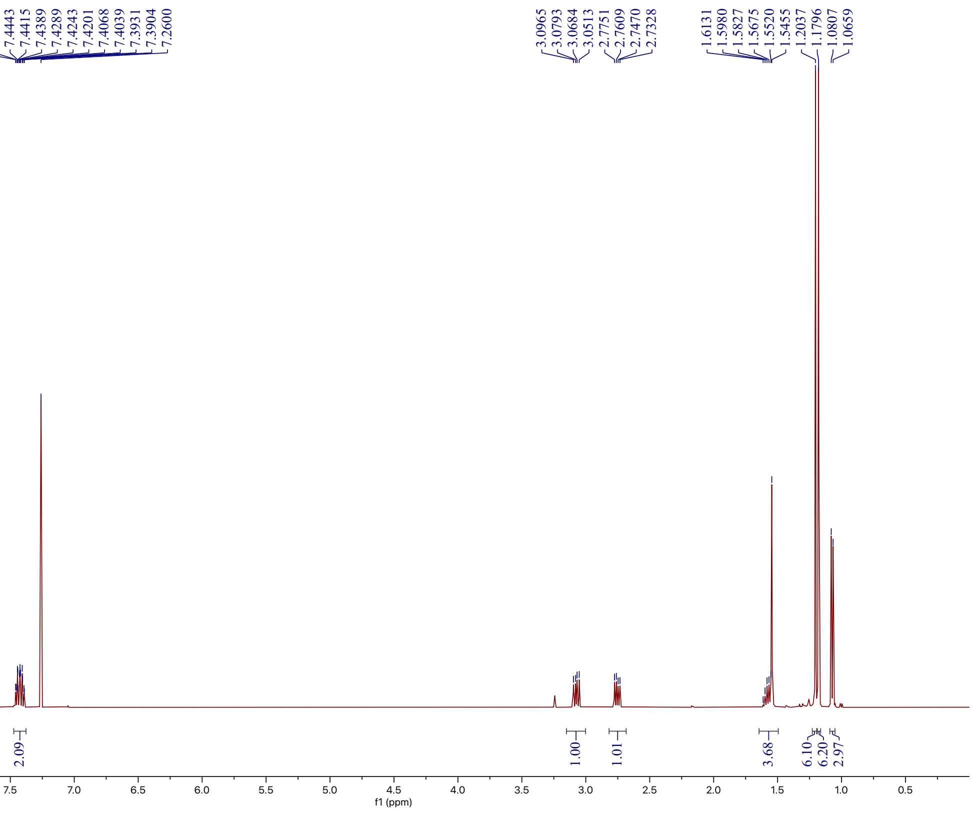

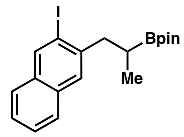

5h, ^{13}C NMR
($CDCl_3$, 126 MHz)

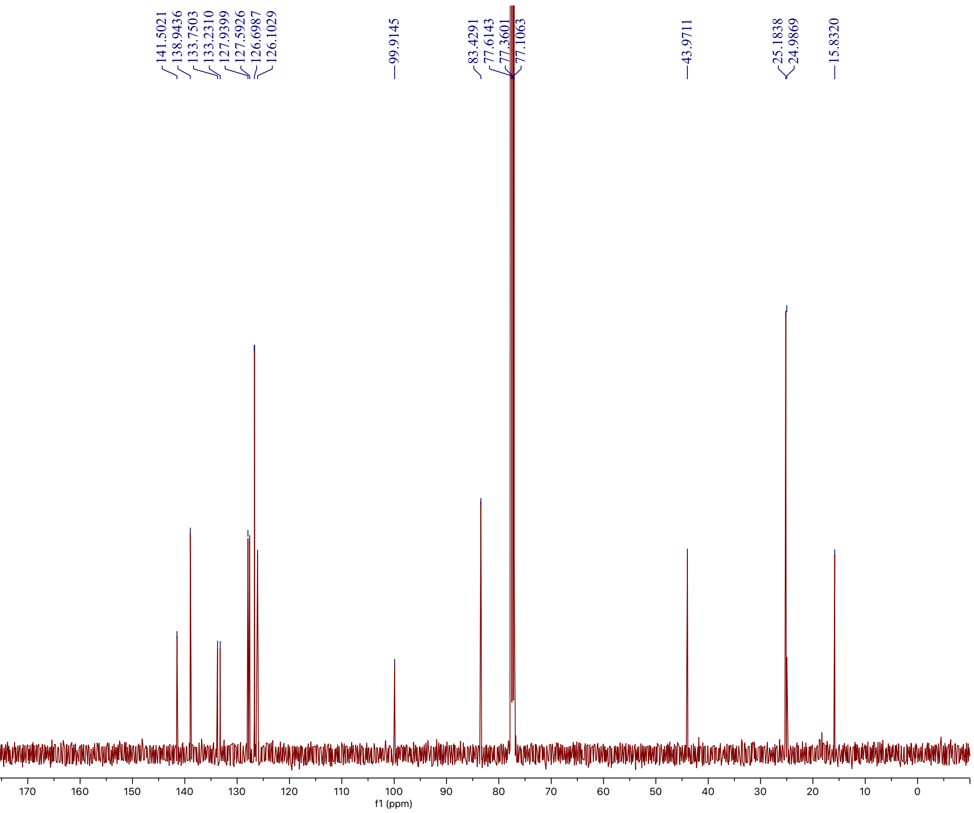

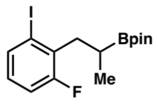

5i, ¹H NMR
($CDCl_3$, 500 MHz)

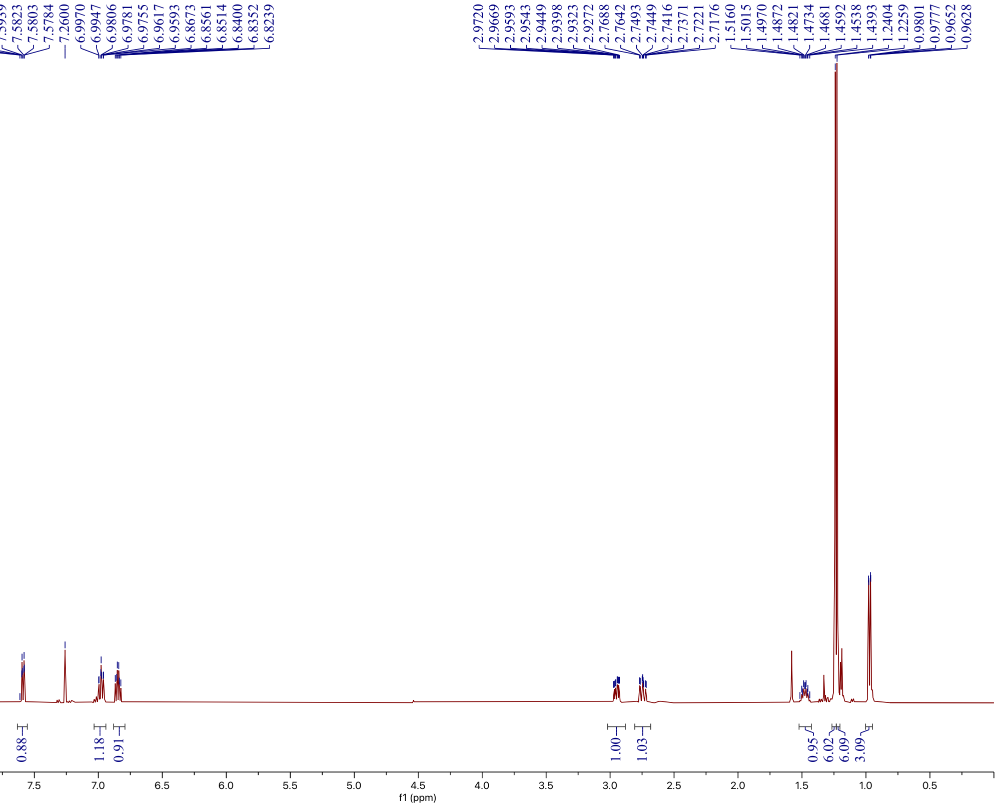

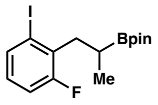

5i, ¹³C NMR
($CDCl_3$, 126 MHz)

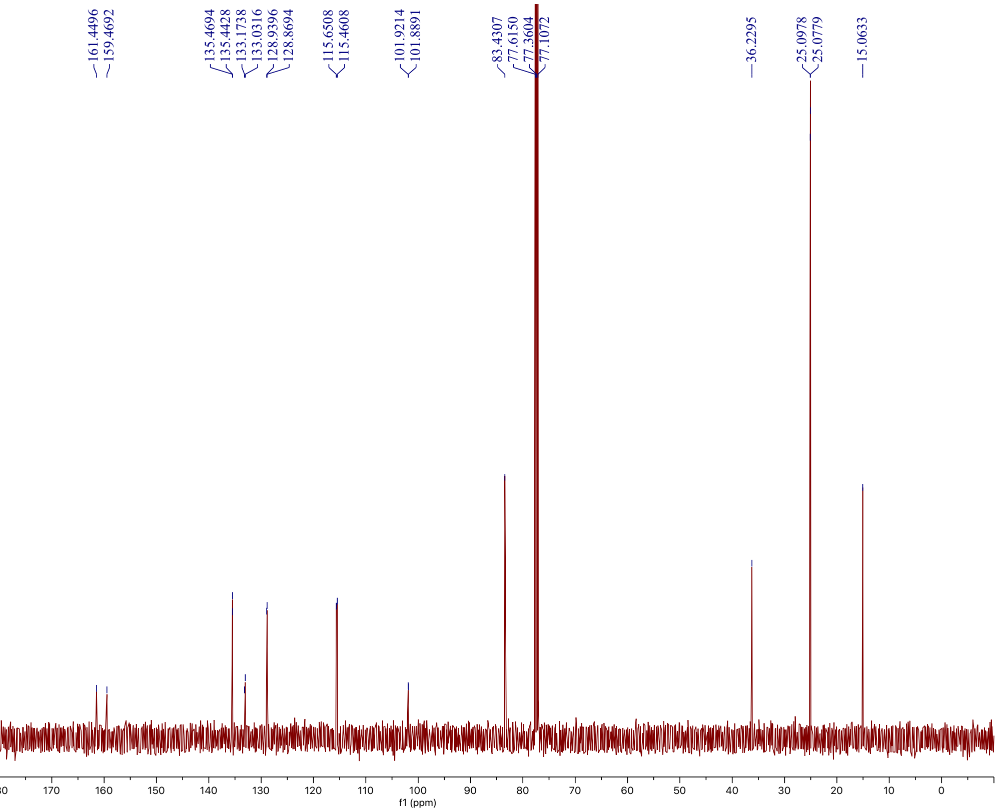

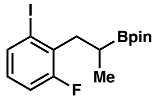

5i, ^{19}F NMR
($CDCl_3$, 376 MHz)

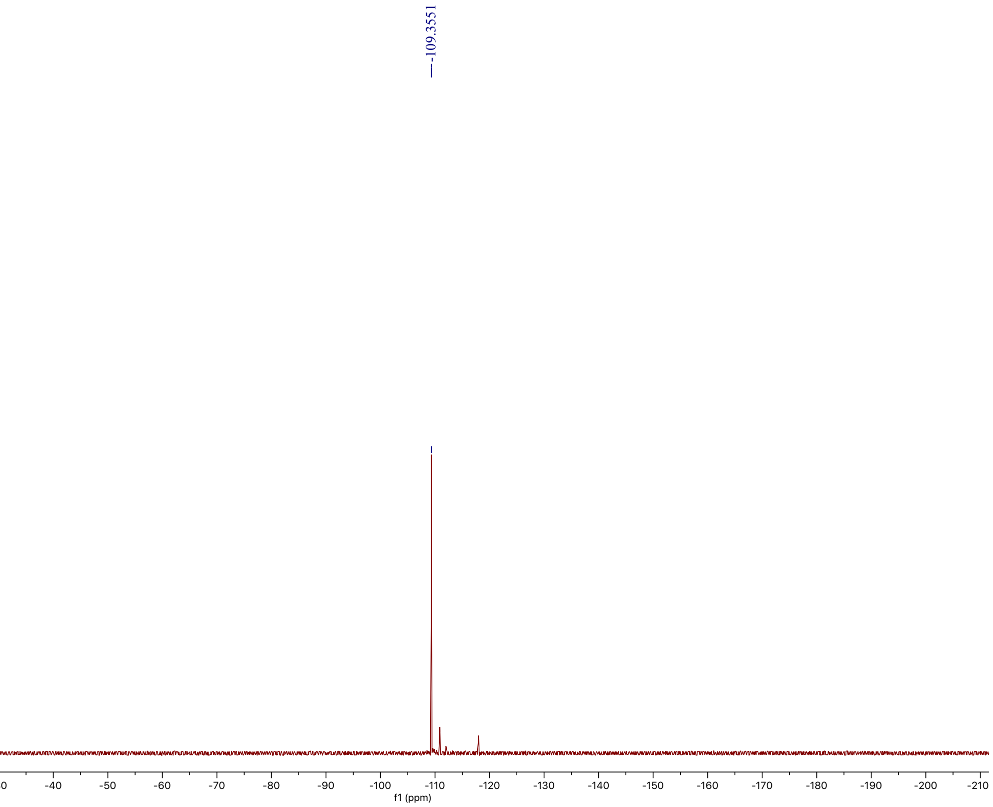

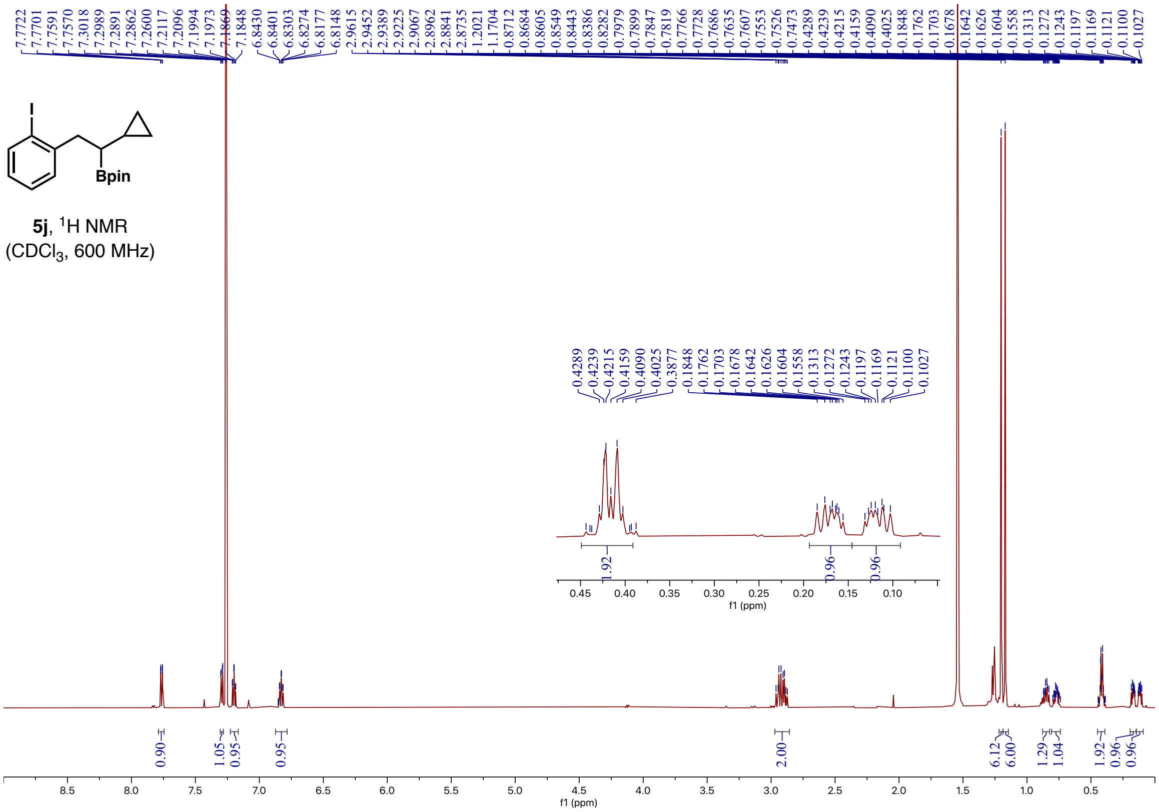

5j, ^{1}H NMR
($CDCl_3$, 600 MHz)

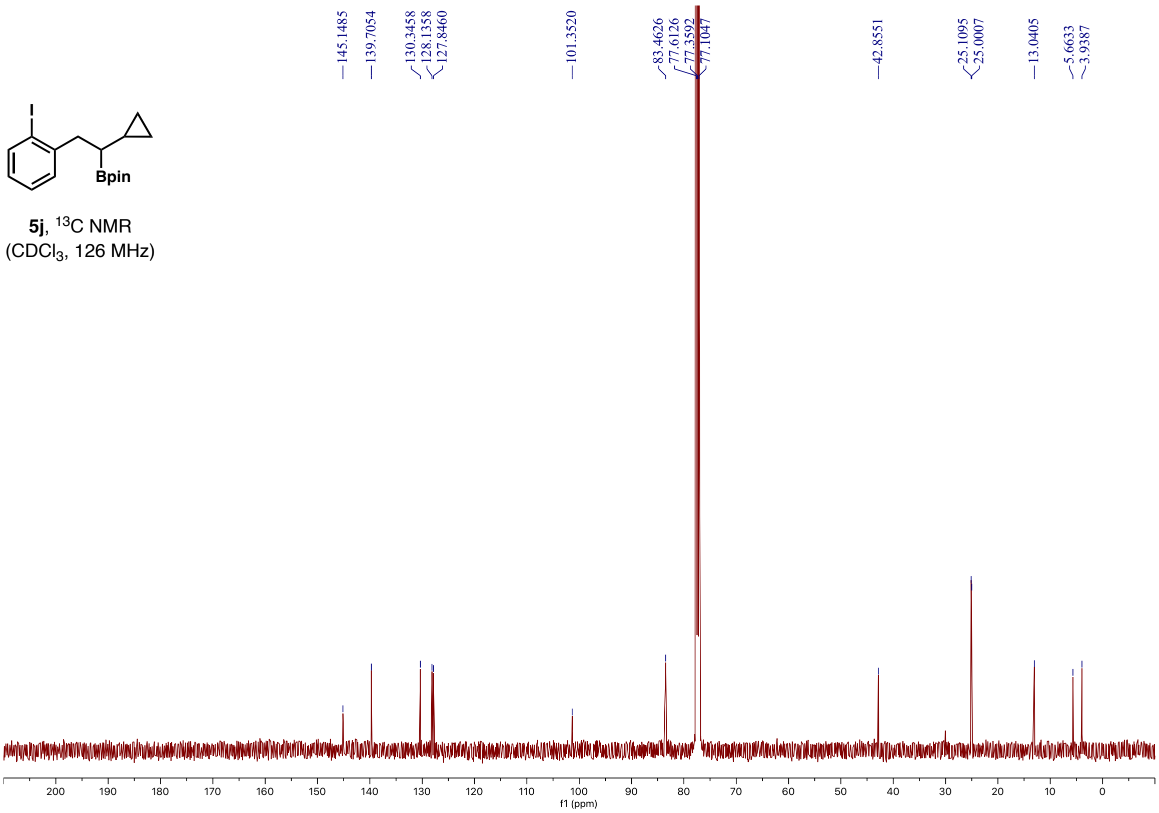

5j, ^{13}C NMR
($CDCl_3$, 126 MHz)

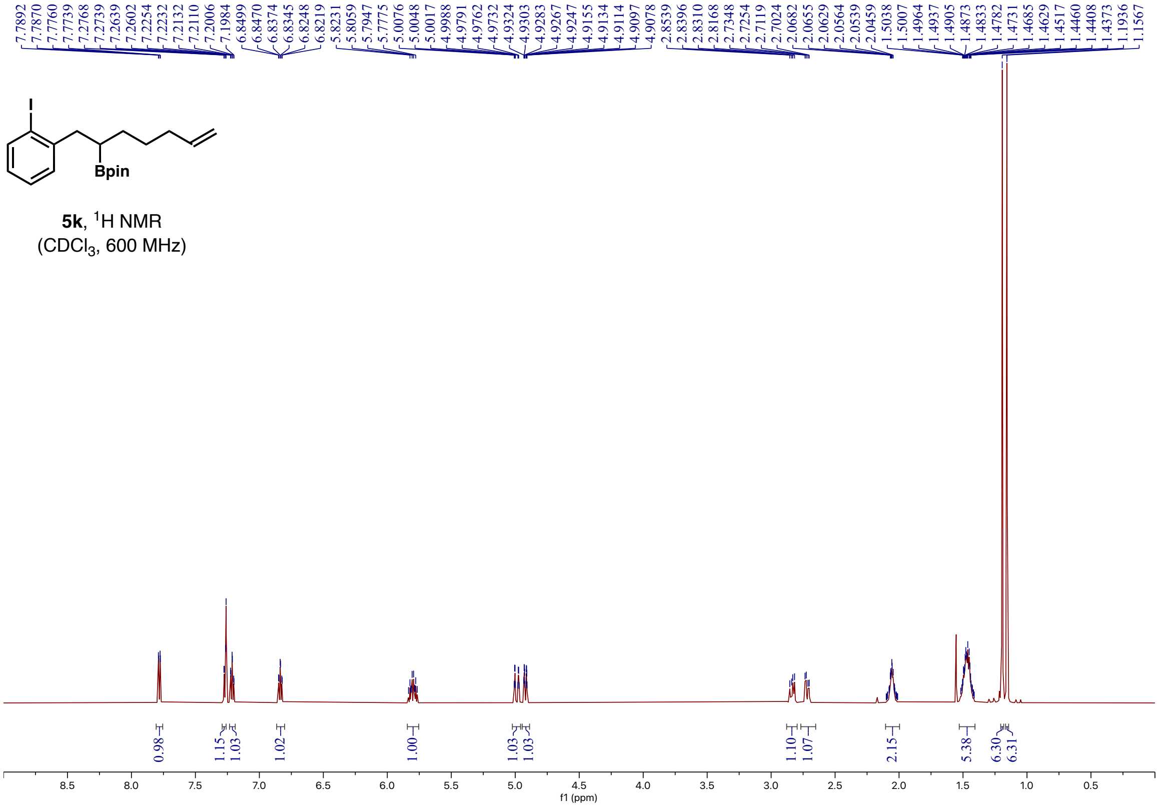

5k, ^{1}H NMR
($CDCl_3$, 600 MHz)

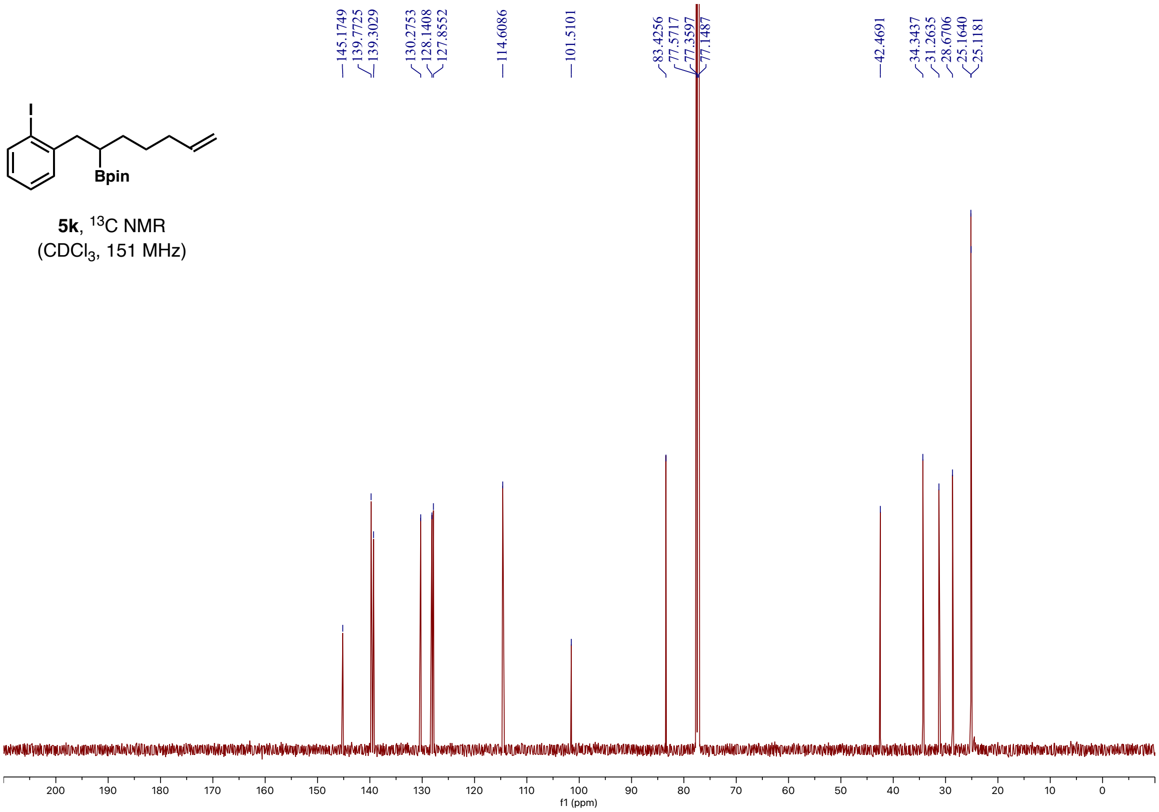

5k, ^{13}C NMR
($CDCl_3$, 151 MHz)

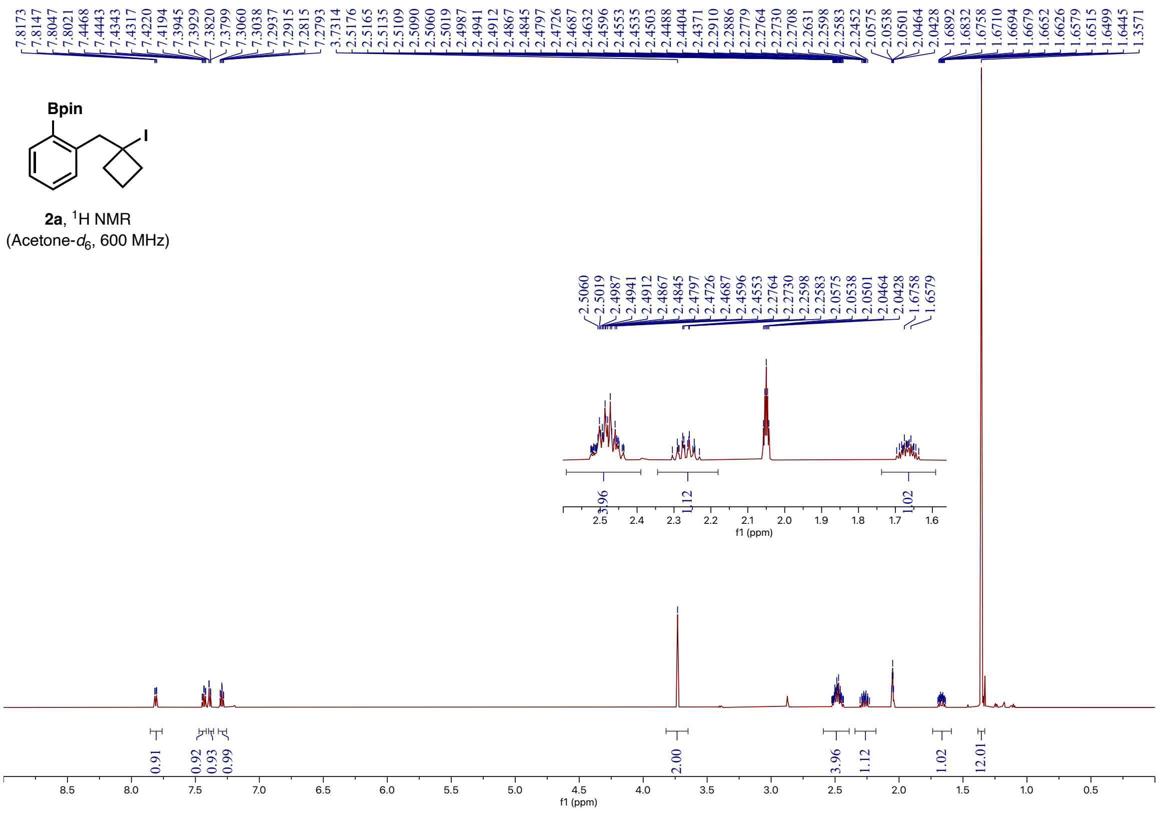
7.8173
7.8147
7.8047
7.8021
7.4468
7.4443
7.4343
7.4317
7.4220
7.4194
7.3945
7.3929
7.3820
7.3799
7.3060
7.3038
7.2937
7.2915
7.2815
7.2793
3.7314
2.5176
2.5165
2.5135
2.5109
2.5090
2.5060
2.5019
2.4987
2.4941
2.4912
2.4867
2.4845
2.4797
2.4726
2.4687
2.4632
2.4596
2.4553
2.4535
2.4503
2.4488
2.4404
2.4371
2.2910
2.2886
2.2779
2.2764
2.2730
2.2708
2.2631
2.2598
2.2583
2.2452
2.0575
2.0538
2.0501
2.0464
2.0428
1.6892
1.6832
1.6758
1.6710
1.6694
1.6679
1.6652
1.6626
1.6579
1.6515
1.6499
1.6445
1.3571
Bpin
I
2a, ^{1}H NMR
(Acetone-d_6, 600 MHz)
0.91
0.92
0.93
0.99
2.00
3.96
1.12
1.02
12.01
f1 (ppm)

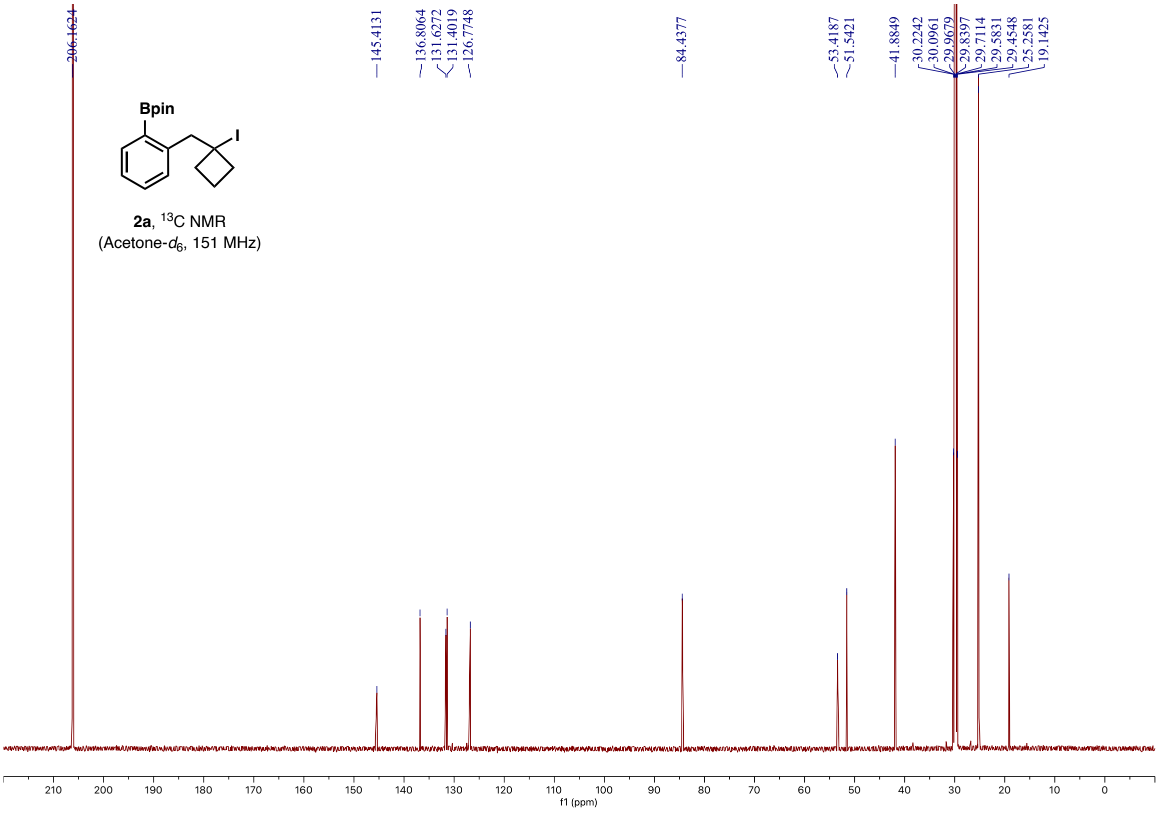
206.1624
145.4131
136.8064
131.6272
131.4019
126.7748
84.4377
53.4187
51.5421
41.8849
30.2242
30.0961
29.9679
29.8397
29.7114
29.5831
29.4548
25.2581
19.1425
Bpin
I
2a, ^{13}C NMR
(Acetone-d_6, 151 MHz)
f1 (ppm)

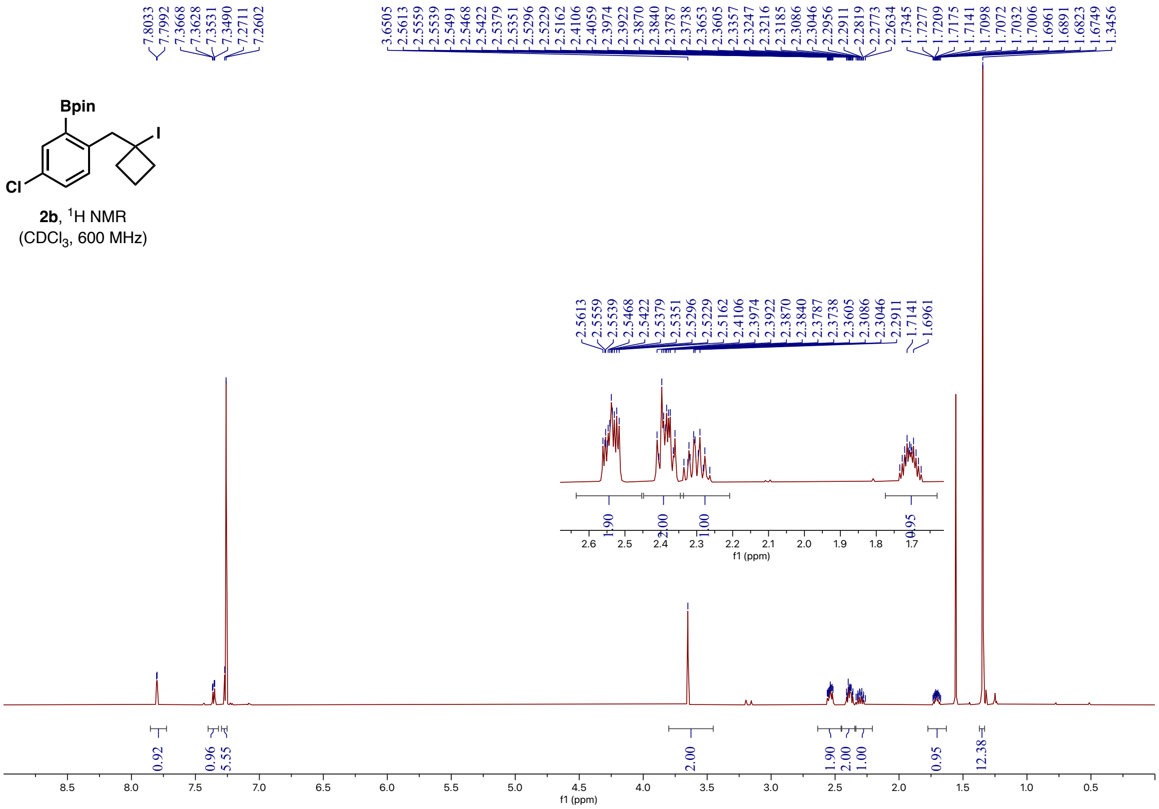

2b, ^{1}H NMR
($CDCl_3$, 600 MHz)

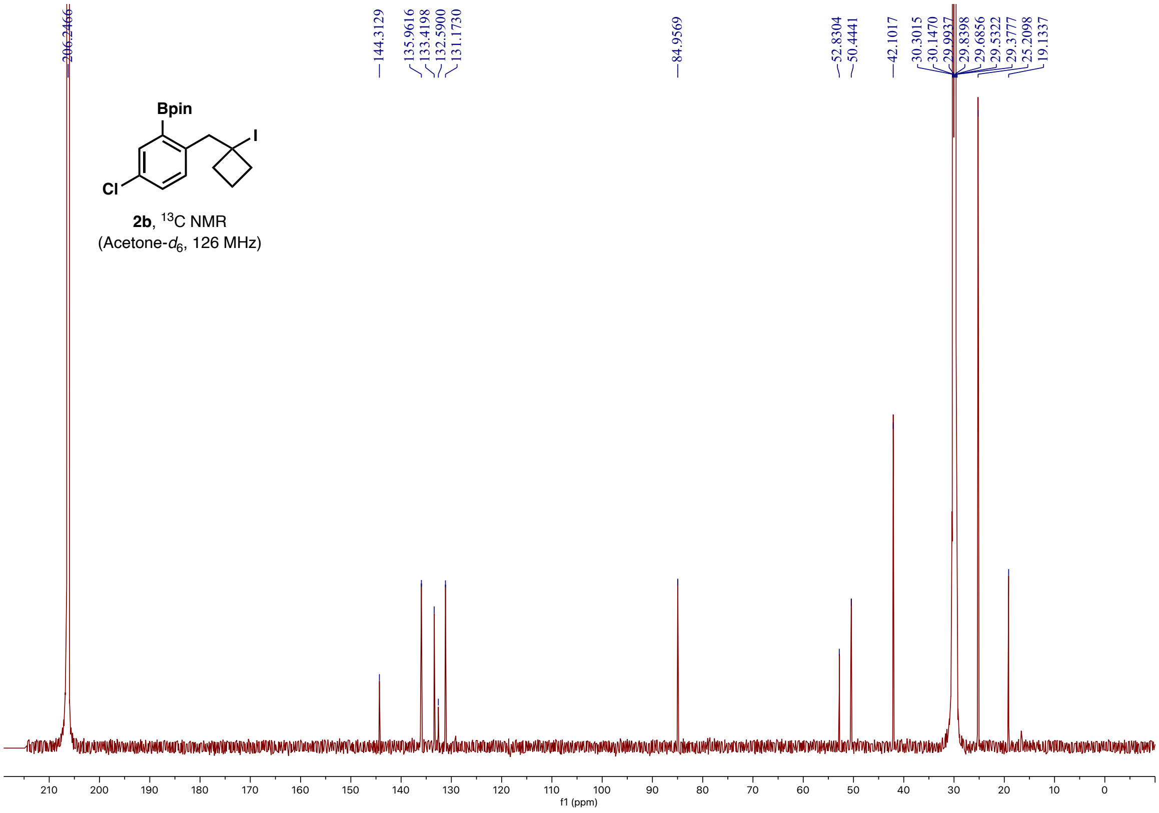

2b, ^{13}C NMR
(Acetone-d_6, 126 MHz)

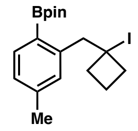

2c, ^{1}H NMR
($CDCl_3$, 600 MHz)

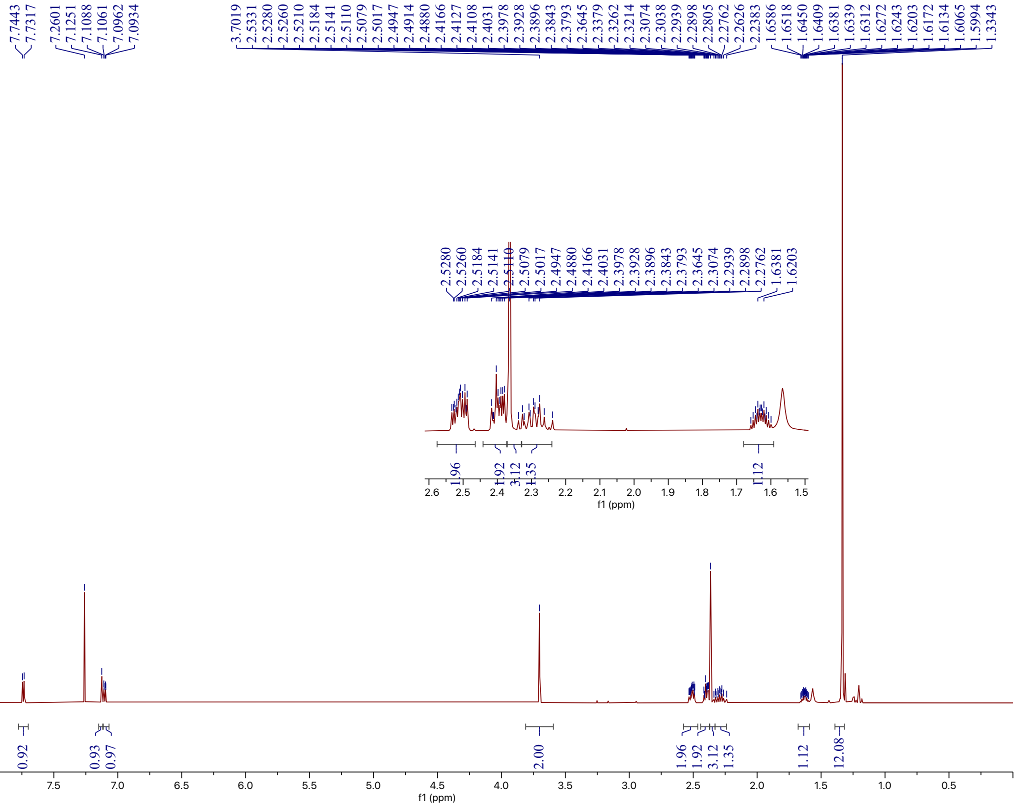

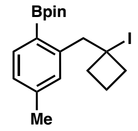

2c, ^{13}C NMR
($CDCl_3$, 126 MHz)

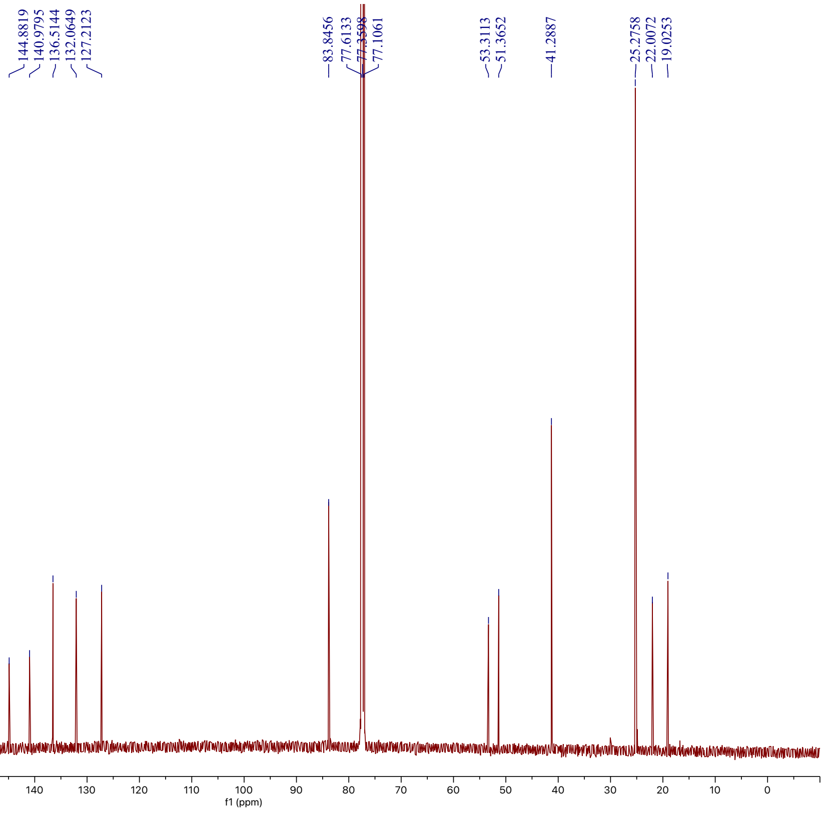

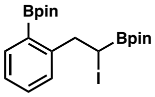

4a, ^{1}H NMR
($CDCl_3$, 600 MHz)

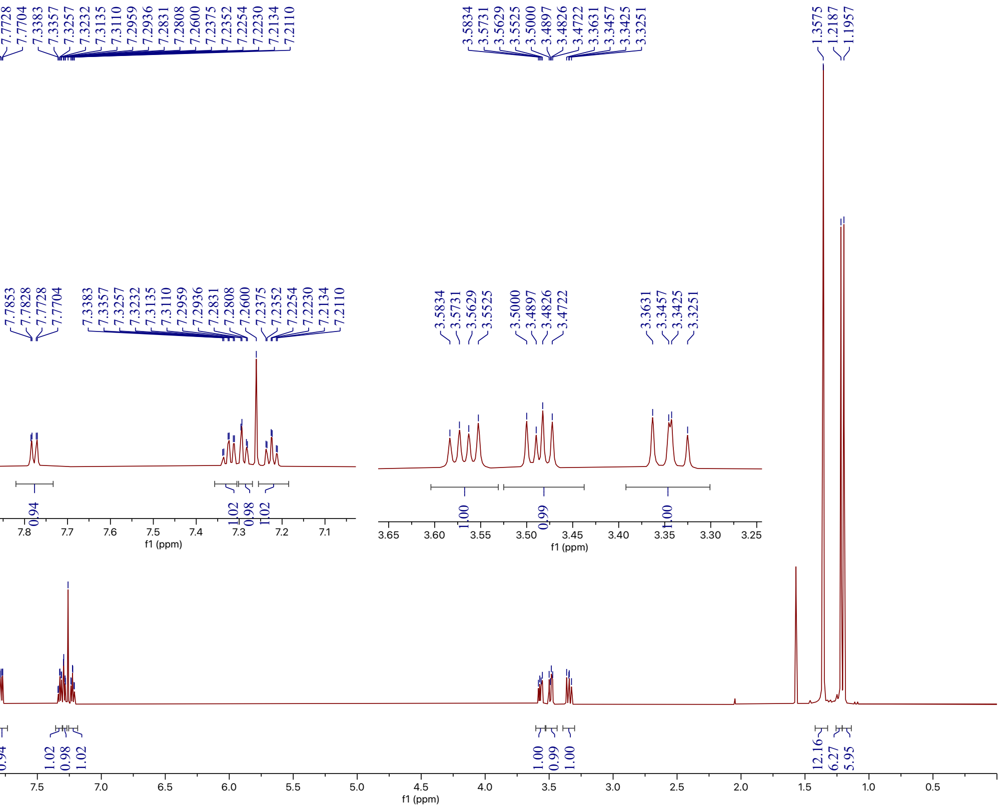

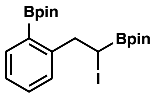

4a, ^{13}C NMR
($CDCl_3$, 151 MHz)

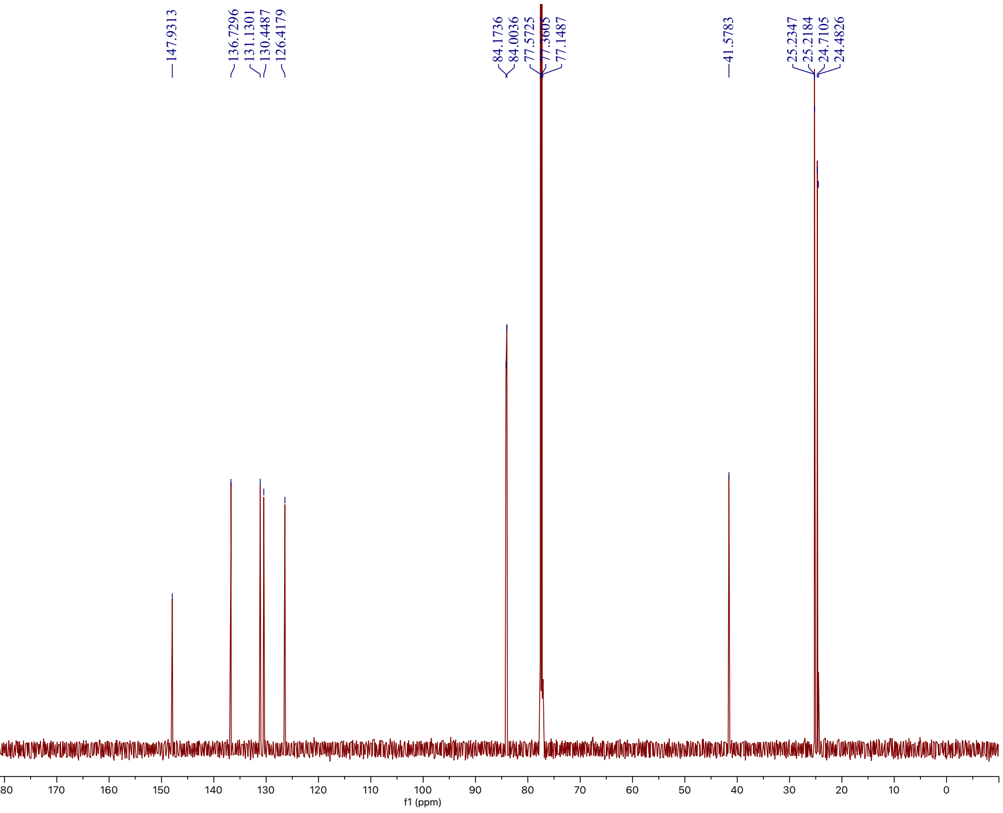

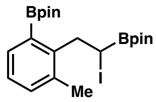

4b, ^{1}H NMR
($CDCl_3$, 600 MHz)

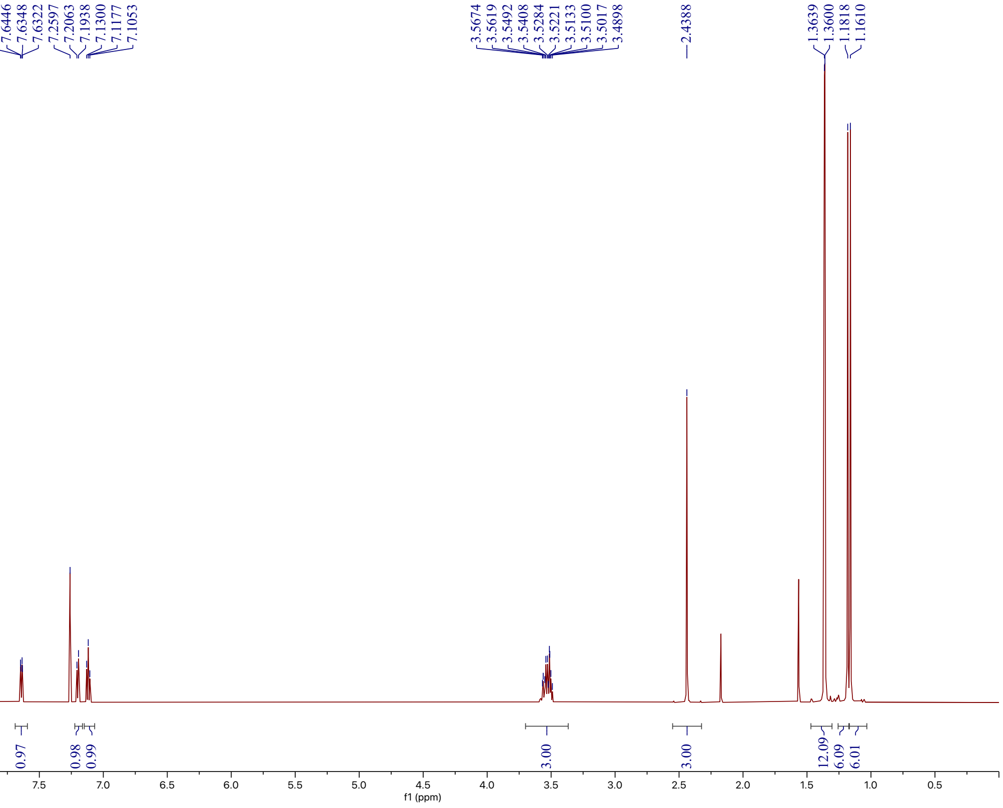

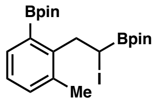

4b, ^{13}C NMR
($CDCl_3$, 151 MHz)

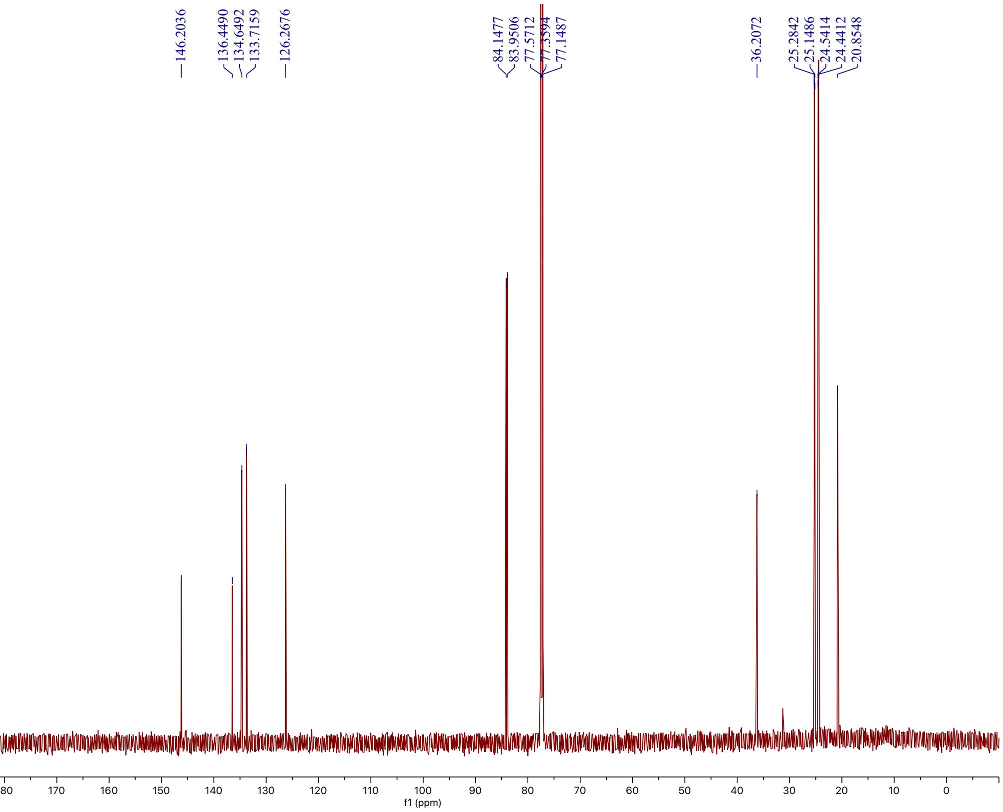

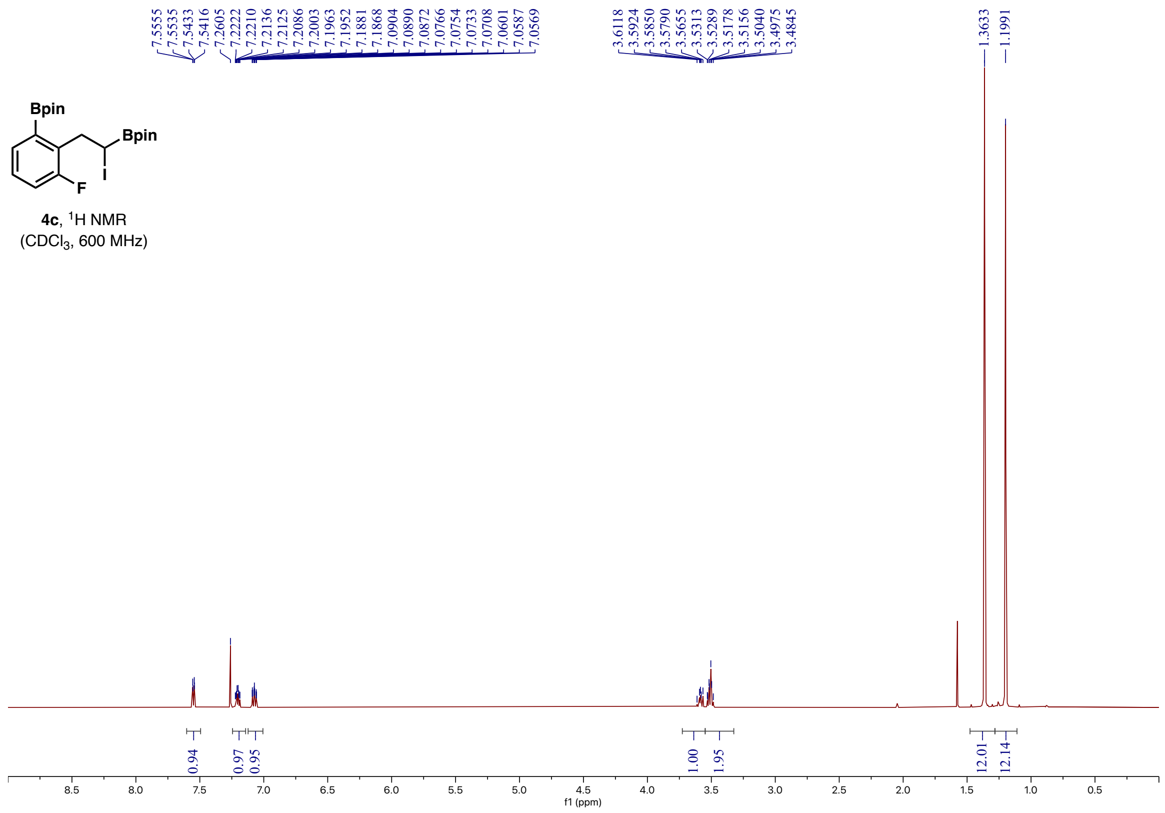

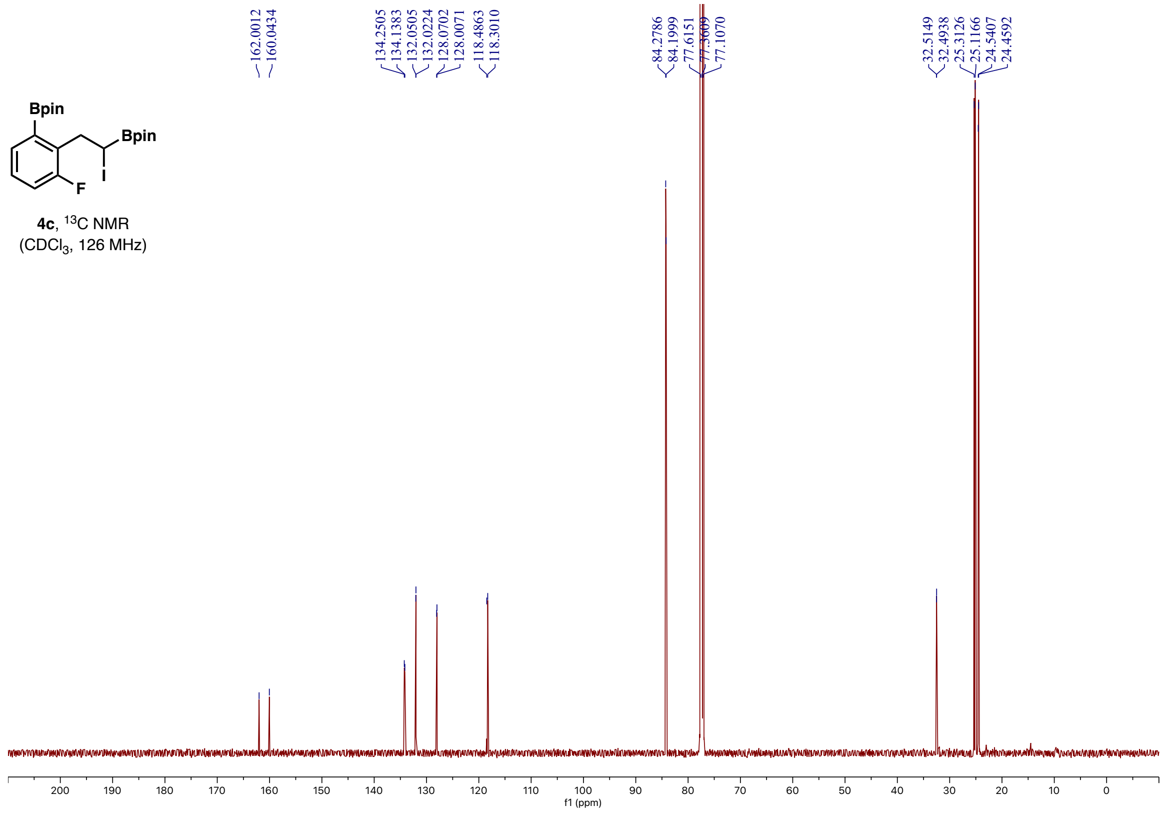

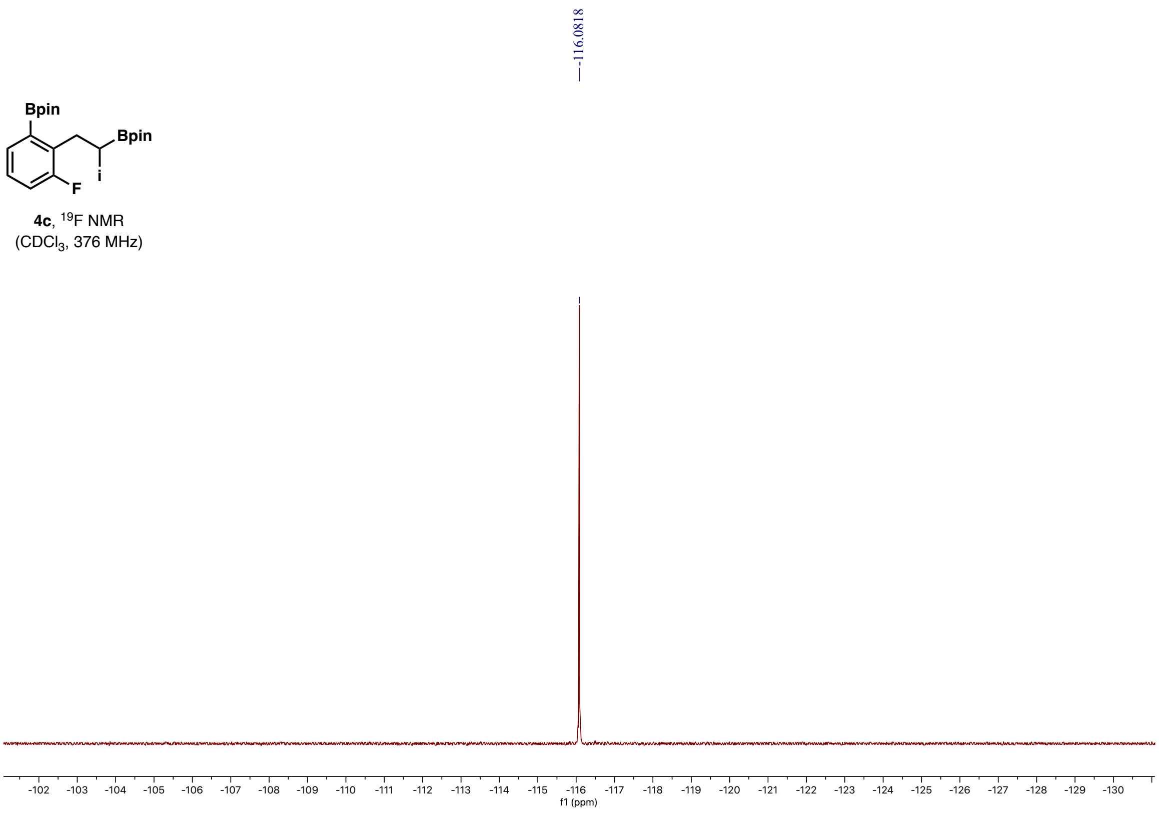

-116.0818
Bpin
Bpin
F
4c, ^{19}F NMR
($CDCl_3$, 376 MHz)
-102 -103 -104 -105 -106 -107 -108 -109 -110 -111 -112 -113 -114 -115 -116 -117 -118 -119 -120 -121 -122 -123 -124 -125 -126 -127 -128 -129 -130
f1 (ppm)

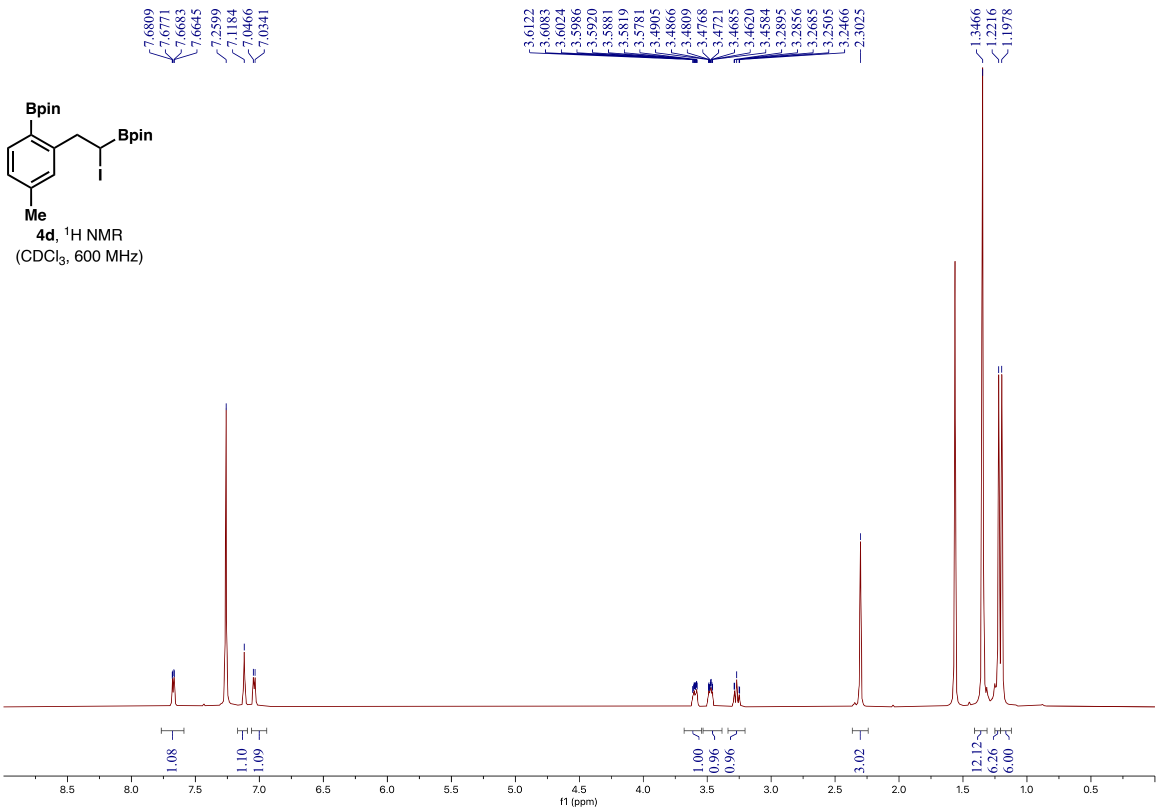
Bpin
Bpin
I
Me
4d, 1H NMR
(CDCl3, 600 MHz)
7.6809
7.6771
7.6683
7.6645
7.2599
7.1184
7.0466
7.0341
3.6122
3.6083
3.6024
3.5986
3.5920
3.5881
3.5819
3.5781
3.4905
3.4866
3.4809
3.4768
3.4721
3.4685
3.4620
3.4584
3.2895
3.2856
3.2685
3.2505
3.2466
2.3025
1.3466
1.2216
1.1978
1.08
1.10
1.09
1.00
0.96
0.96
3.02
12.12
6.26
6.00
f1 (ppm)

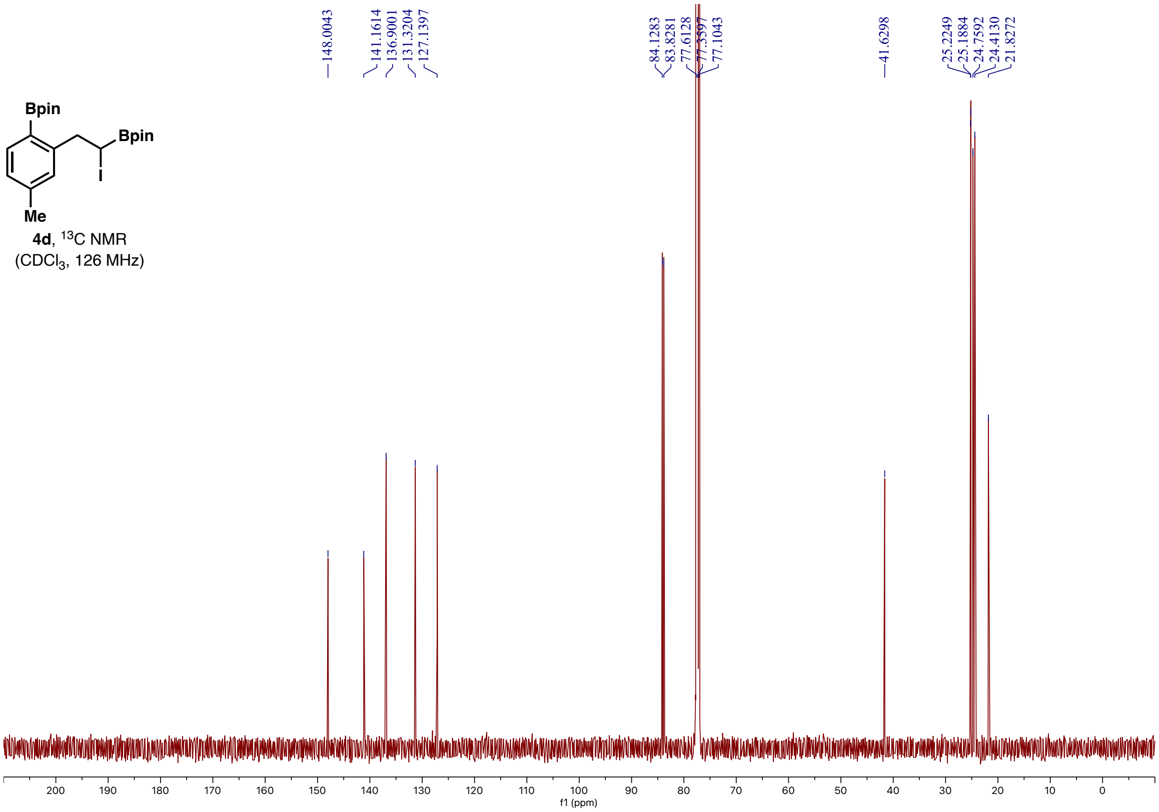
Bpin
Bpin
I
Me
4d, 13C NMR
(CDCl3, 126 MHz)
148.0043
141.1614
136.9001
131.3204
127.1397
84.1283
83.8281
77.6128
77.3597
77.1043
41.6298
25.2249
25.1884
24.7592
24.4130
21.8272
f1 (ppm)

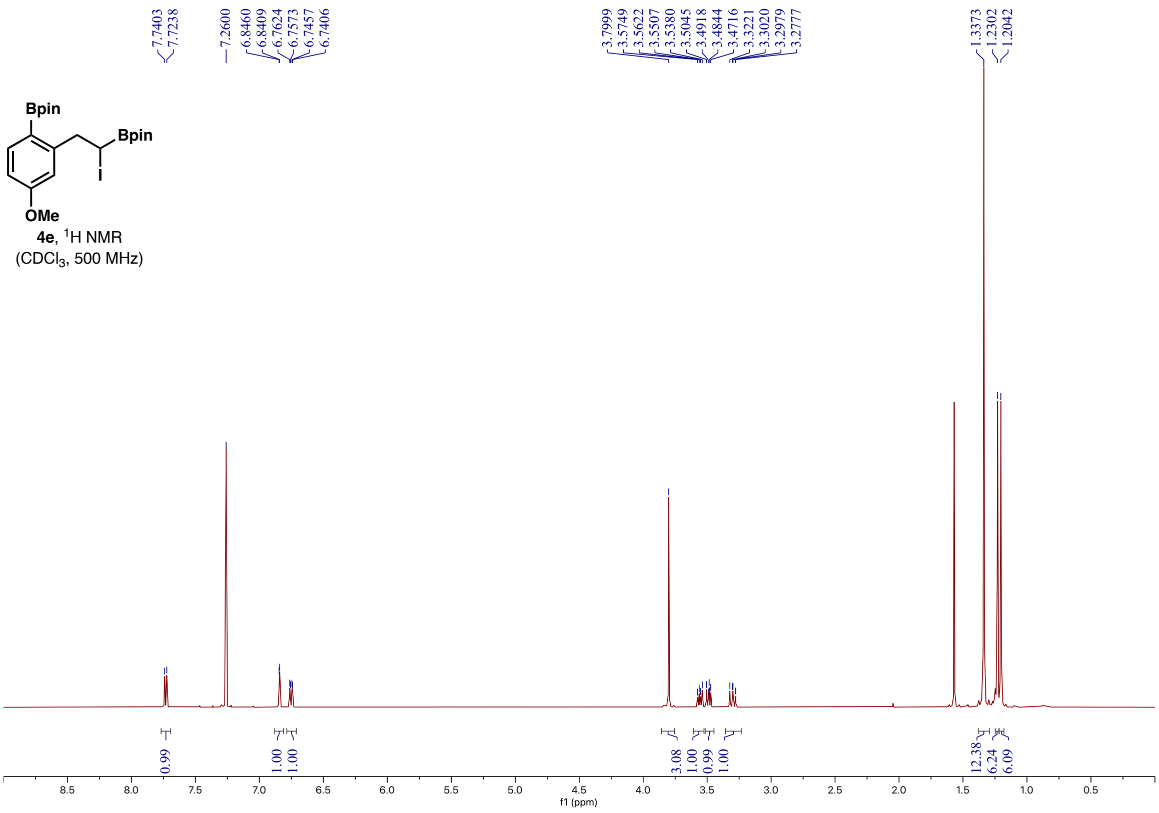

4e, ^{1}H NMR
($CDCl_3$, 500 MHz)

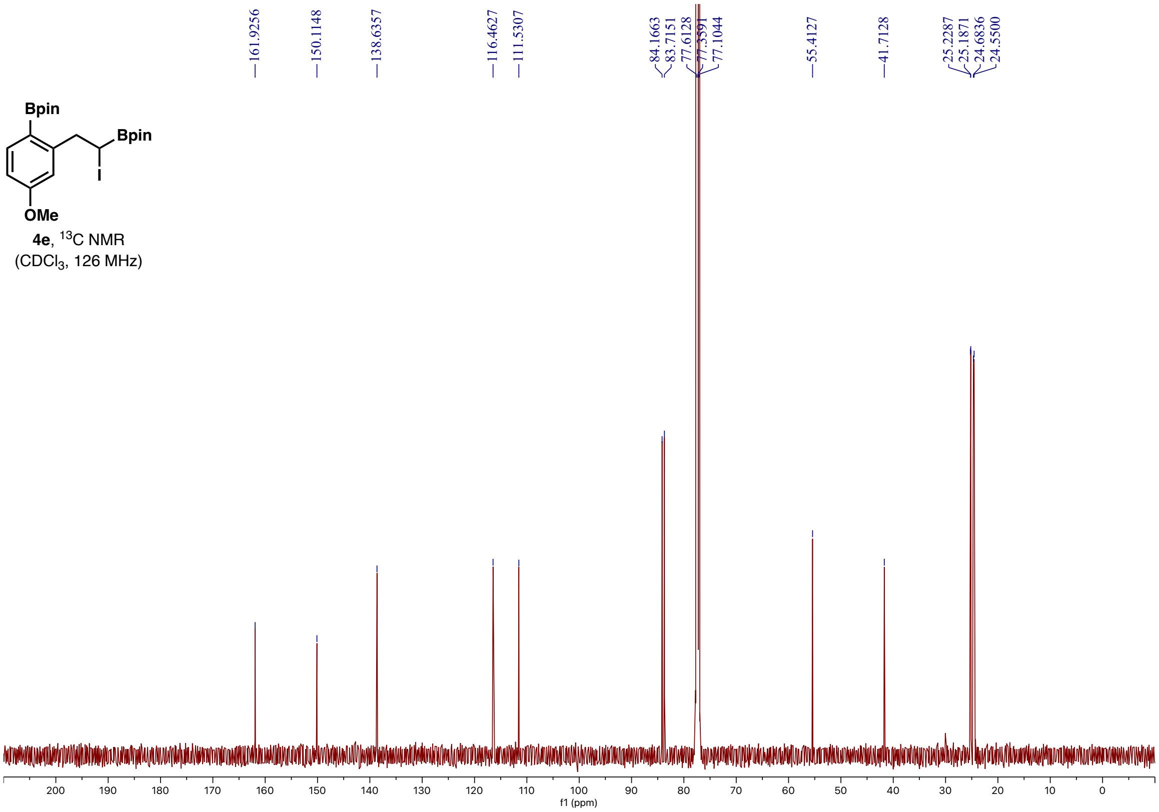

4e, ^{13}C NMR
($CDCl_3$, 126 MHz)

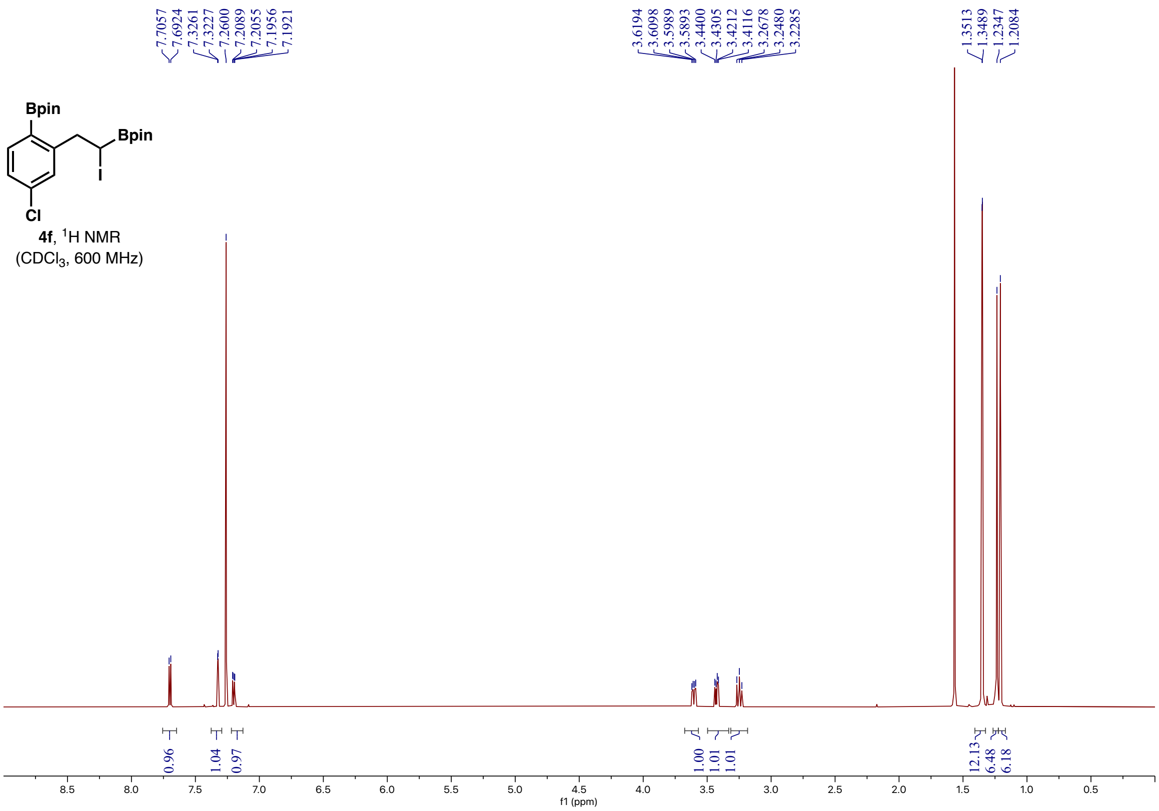

4f, ^{1}H NMR
($CDCl_3$, 600 MHz)

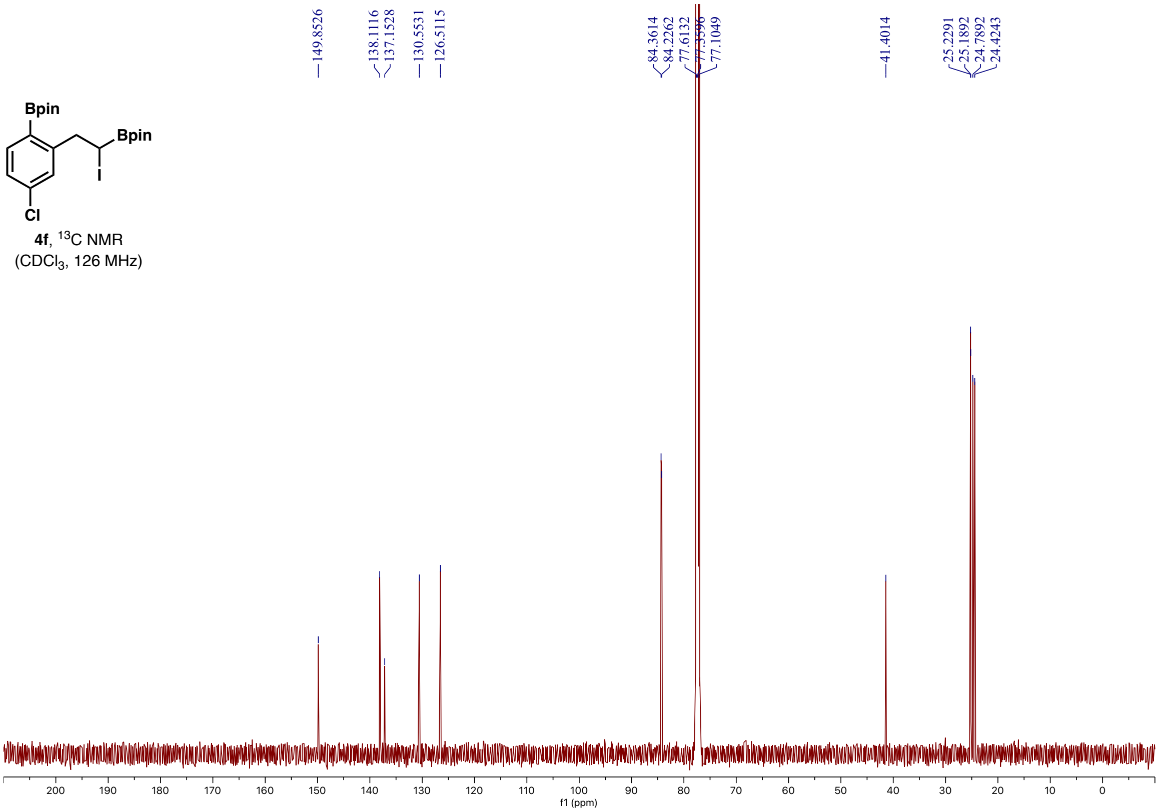

4f, ^{13}C NMR
($CDCl_3$, 126 MHz)

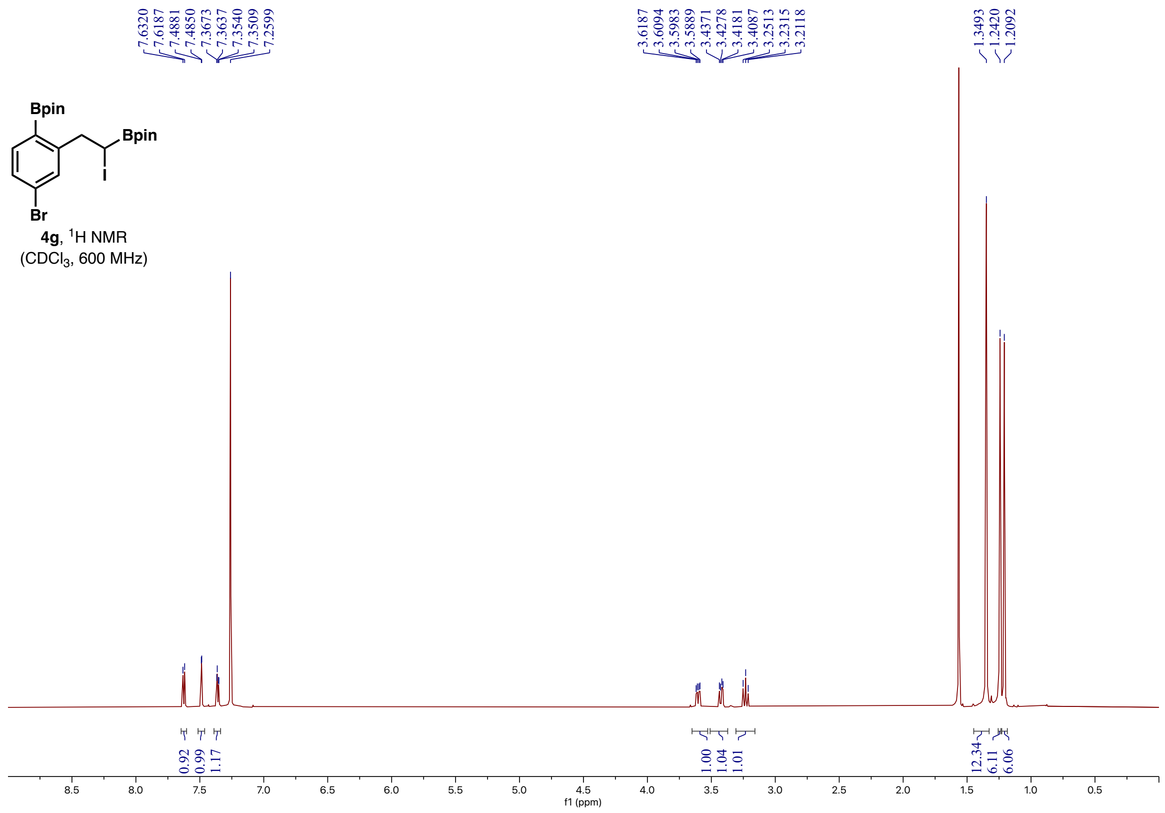

7.6320
7.6187
7.4881
7.4850
7.3673
7.3637
7.3540
7.3509
7.2599
3.6187
3.6094
3.5983
3.5889
3.4371
3.4278
3.4181
3.4087
3.2513
3.2315
3.2118
1.3493
1.2420
1.2092
Bpin
Bpin
I
Br
4g, 1H NMR
(CDCl3, 600 MHz)
0.92
0.99
1.17
1.00
1.04
1.01
12.34
6.11
6.06
f1 (ppm)

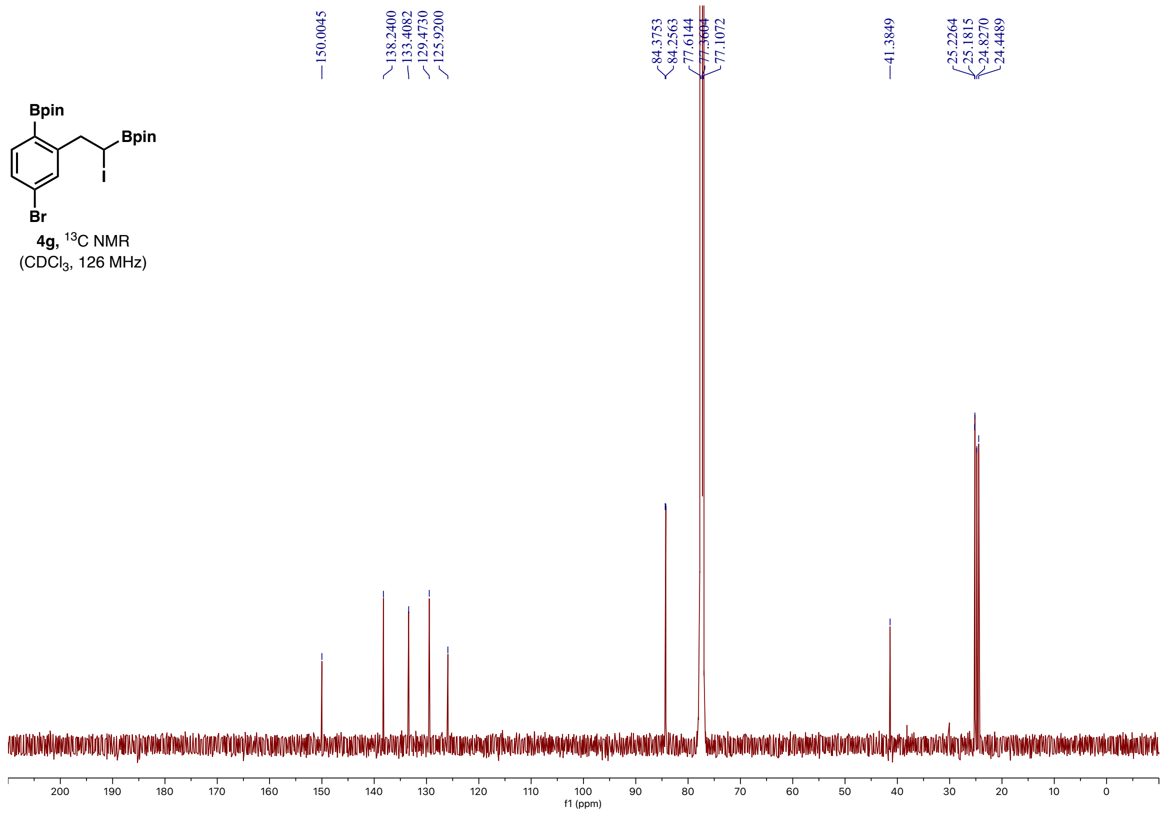

150.0045
138.2400
133.4082
129.4730
125.9200
84.3753
84.2563
77.6144
77.3604
77.1072
41.3849
25.2264
25.1815
24.8270
24.4489
Bpin
Bpin
I
Br
4g, 13C NMR
(CDCl3, 126 MHz)
f1 (ppm)

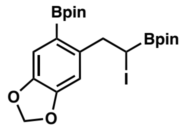

4h, ^{1}H NMR
($CDCl_3$, 600 MHz)

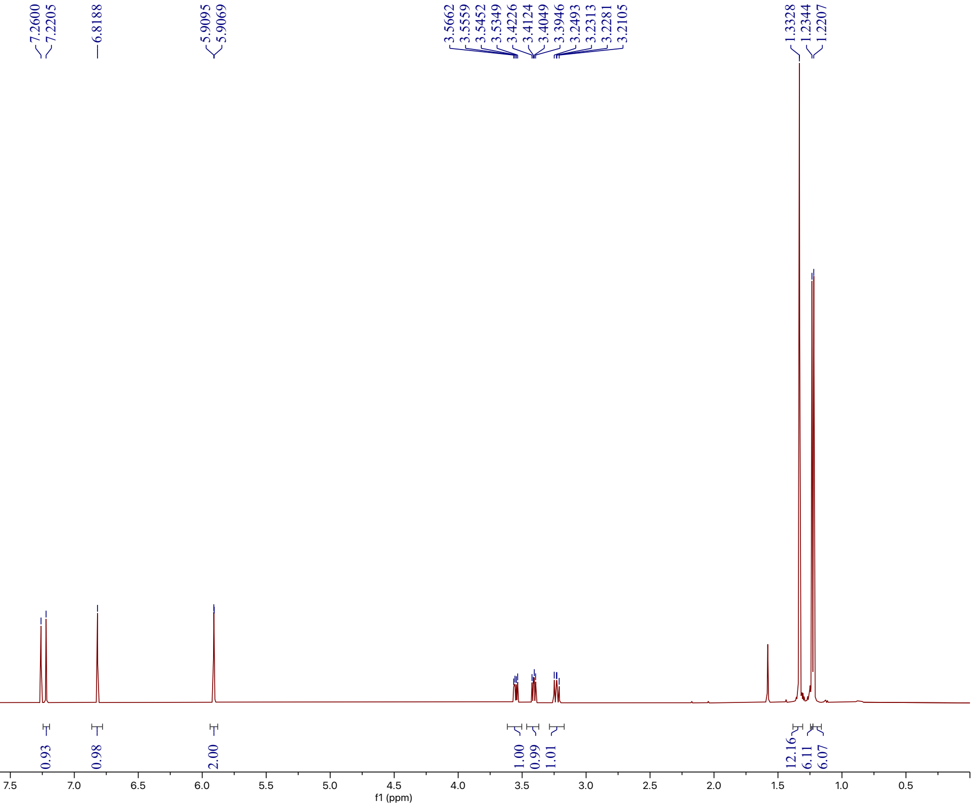

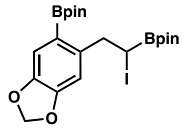

4h, ^{13}C NMR
($CDCl_3$, 151 MHz)

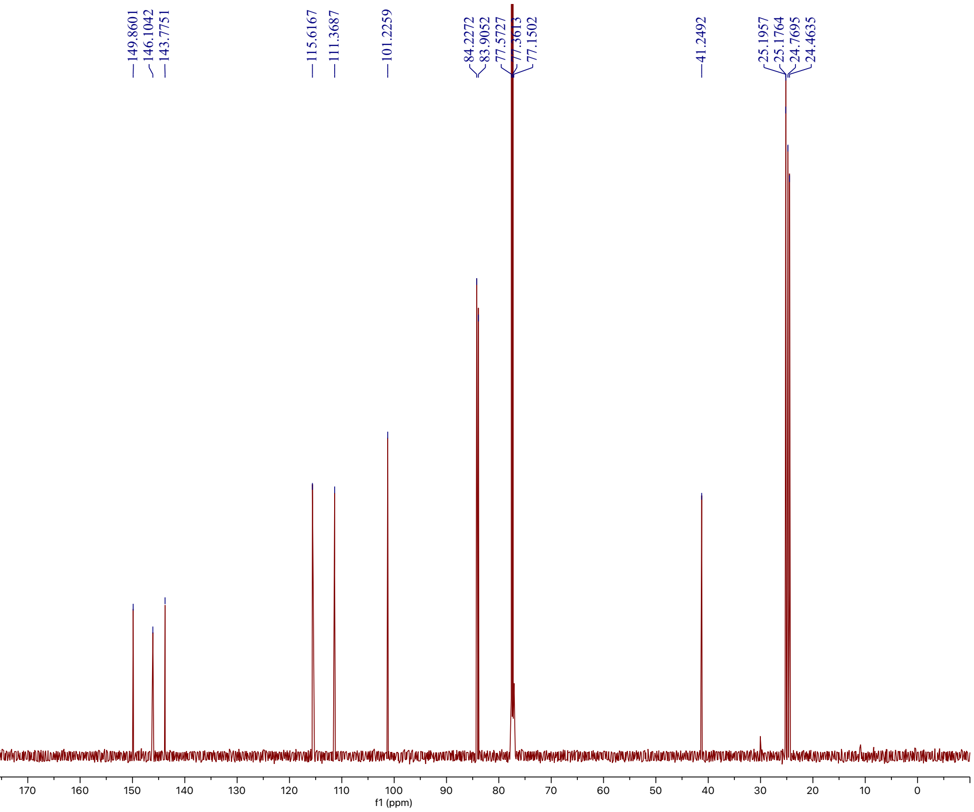

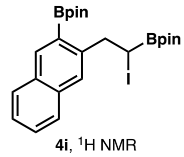

4i, ^{1}H NMR
($CDCl_3$, 600 MHz)

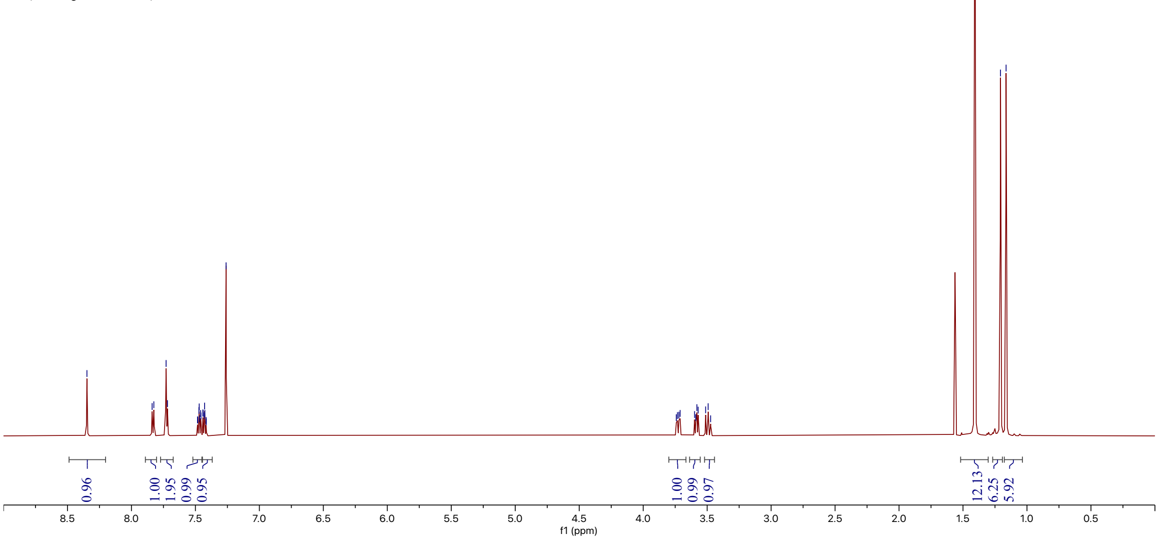

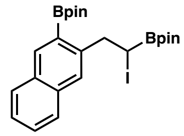

4i, ^{13}C NMR
($CDCl_3$, 151 MHz)

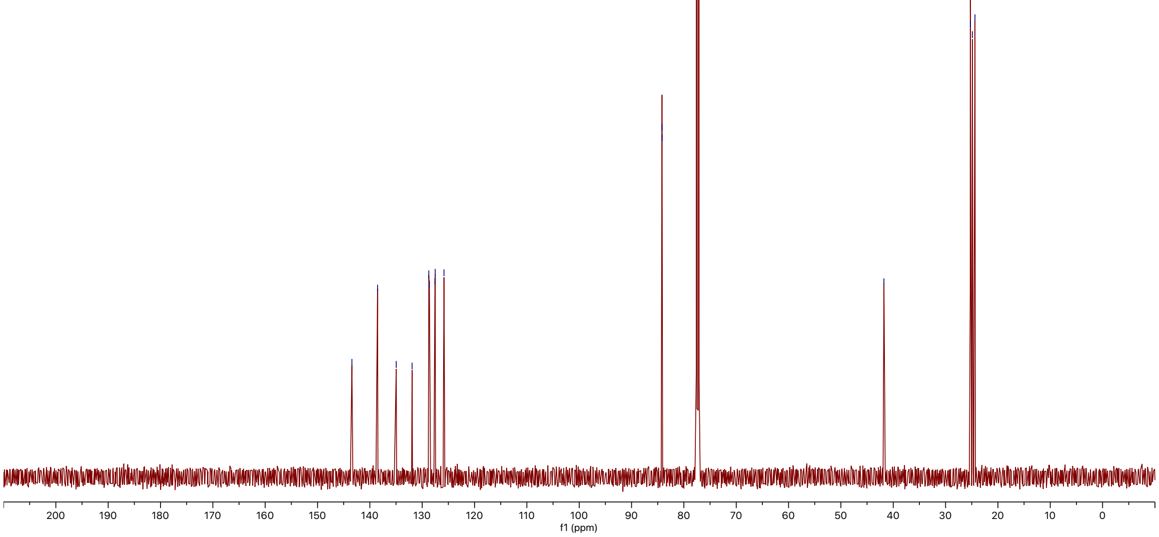

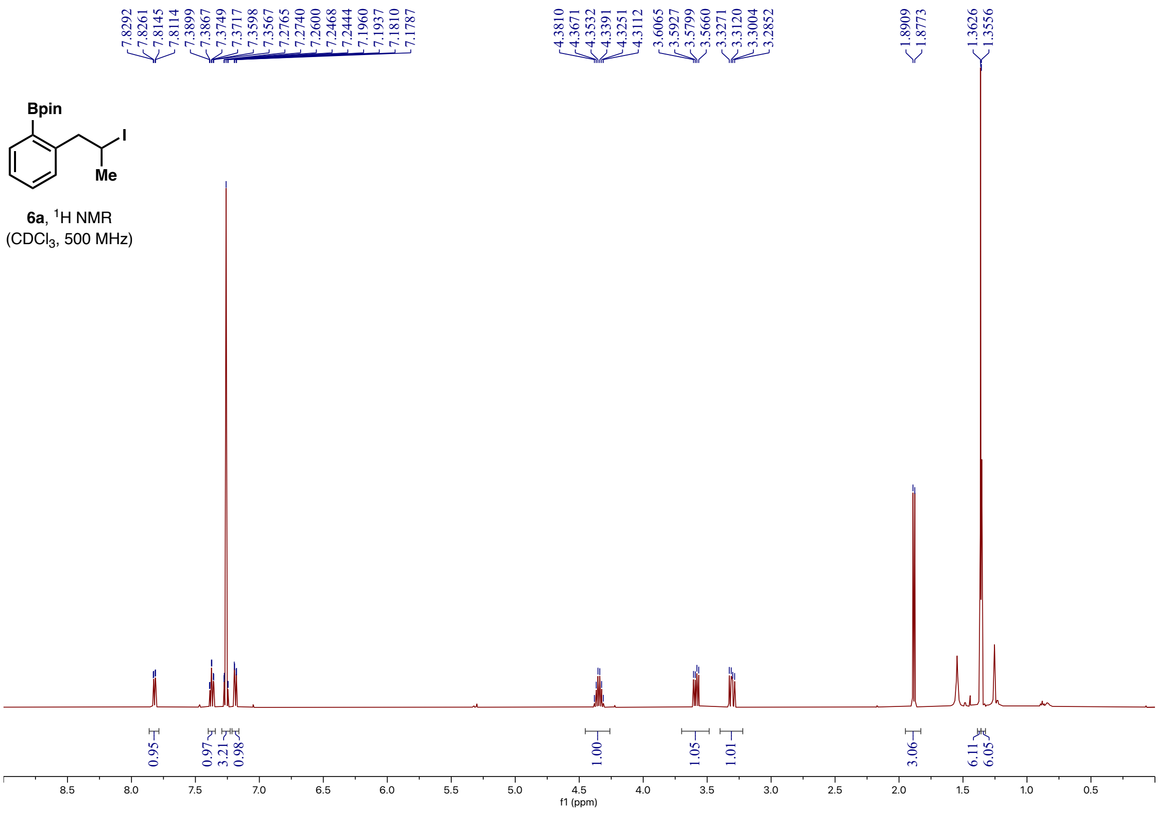
7.8292
7.8261
7.8145
7.8114
7.3899
7.3867
7.3749
7.3717
7.3598
7.3567
7.2765
7.2740
7.2600
7.2468
7.2444
7.1960
7.1937
7.1810
7.1787
4.3810
4.3671
4.3532
4.3391
4.3251
4.3112
3.6065
3.5927
3.5799
3.5660
3.3271
3.3120
3.3004
3.2852
1.8909
1.8773
1.3626
1.3556
Bpin
I
Me
6a, ^{1}H NMR
(CDCl$_3$, 500 MHz)
0.95
0.97
3.21
0.98
1.00
1.05
1.01
3.06
6.11
6.05
f1 (ppm)

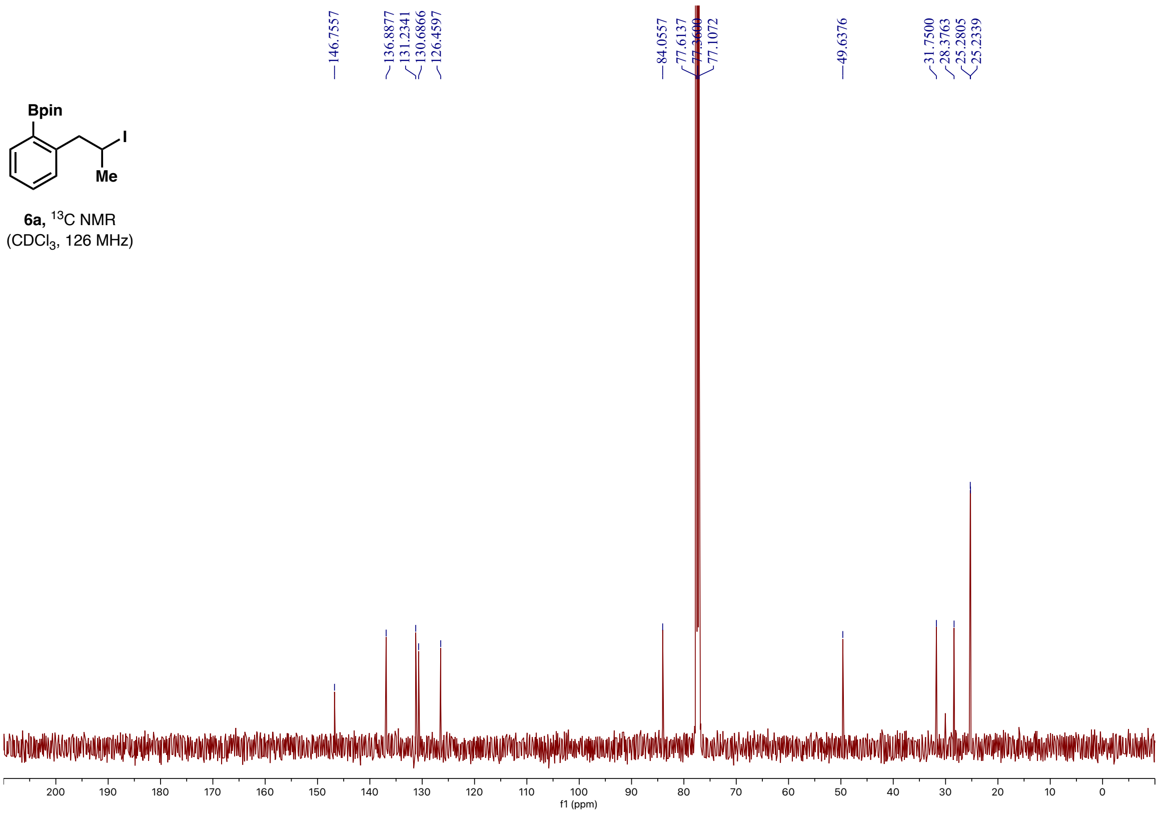
146.7557
136.8877
131.2341
130.6866
126.4597
84.0557
77.6137
77.3600
77.1072
49.6376
31.7500
28.3763
25.2805
25.2339
Bpin
I
Me
6a, ^{13}C NMR
(CDCl$_3$, 126 MHz)
f1 (ppm)

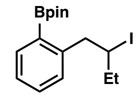

6b, ^{1}H NMR
($CDCl_3$, 500 MHz)

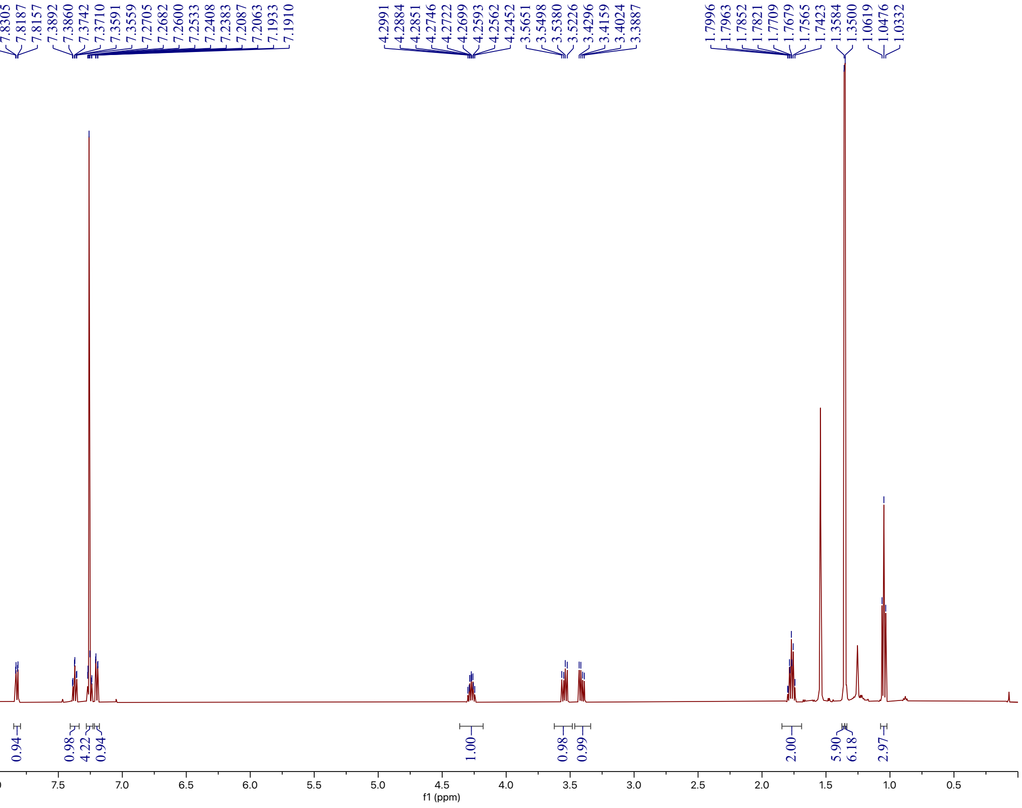

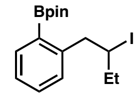

6b, ^{13}C NMR
($CDCl_3$, 126 MHz)

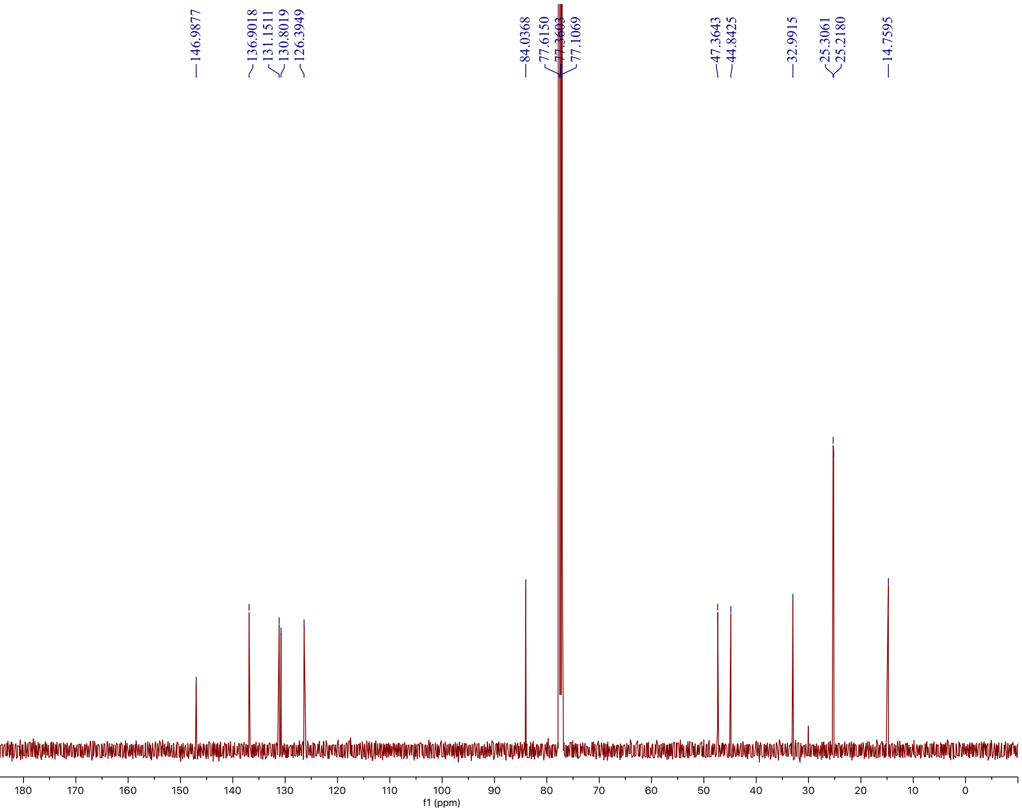

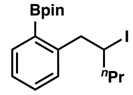

6c, ^{1}H NMR
($CDCl_3$, 500 MHz)

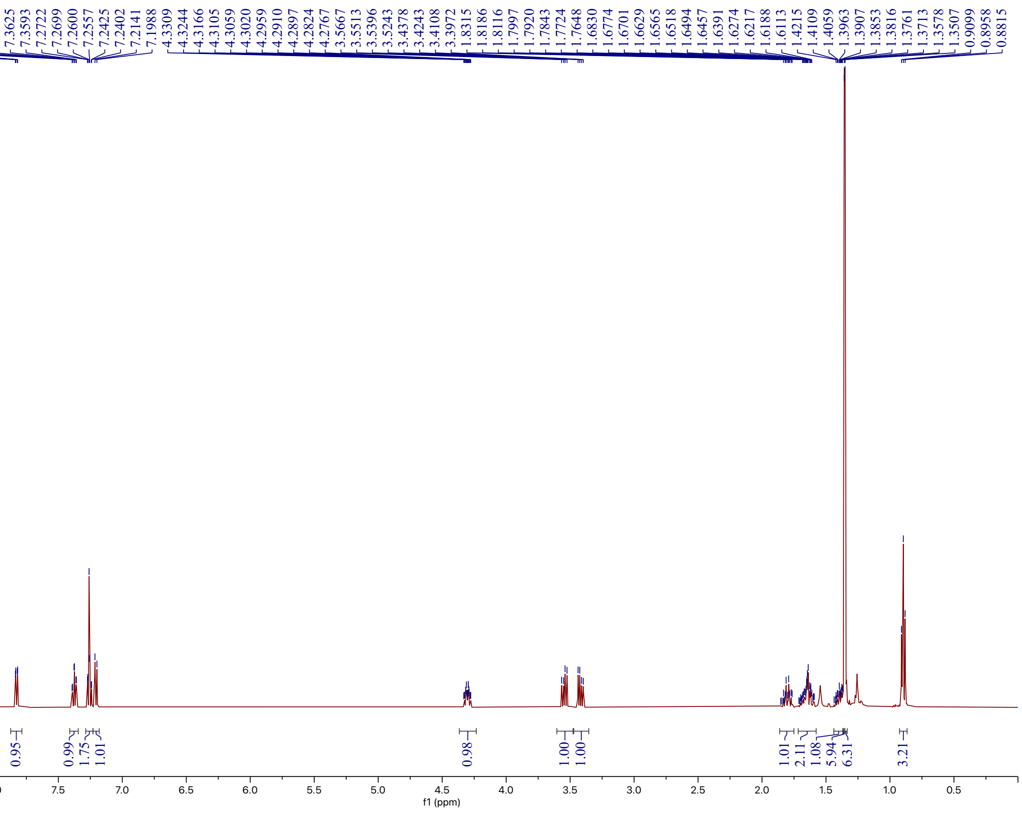

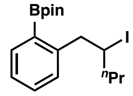

6c, ^{13}C NMR
($CDCl_3$, 126 MHz)

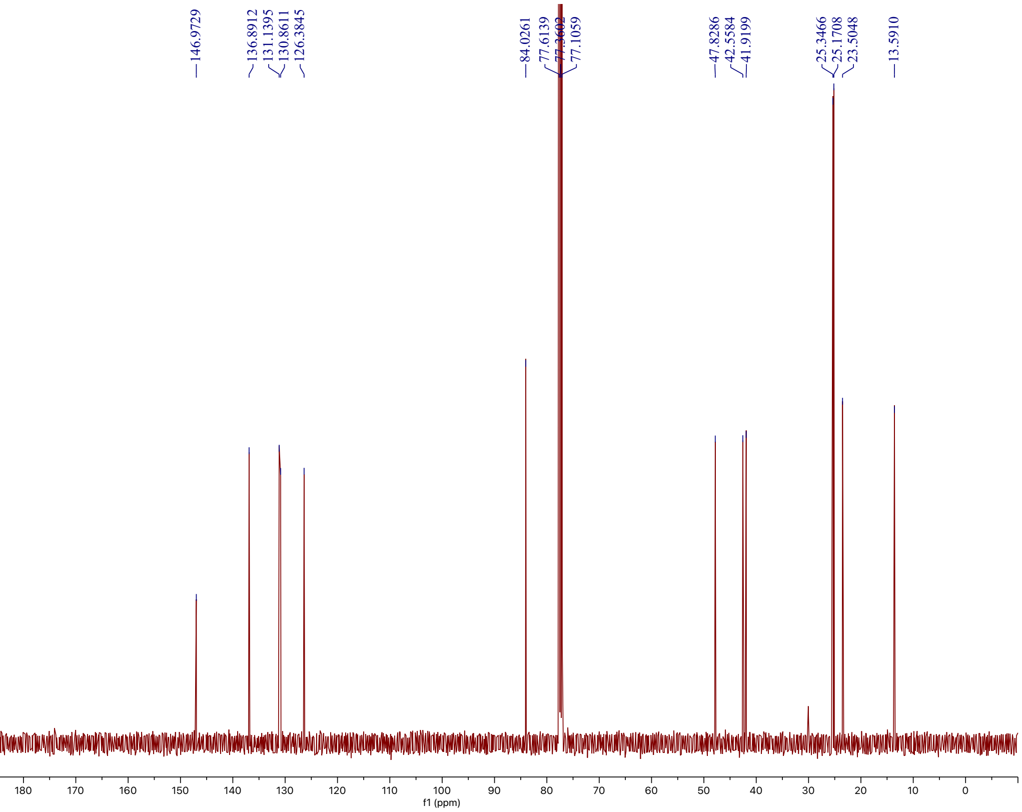

6d, ^{1}H NMR
($CDCl_3$, 500 MHz)

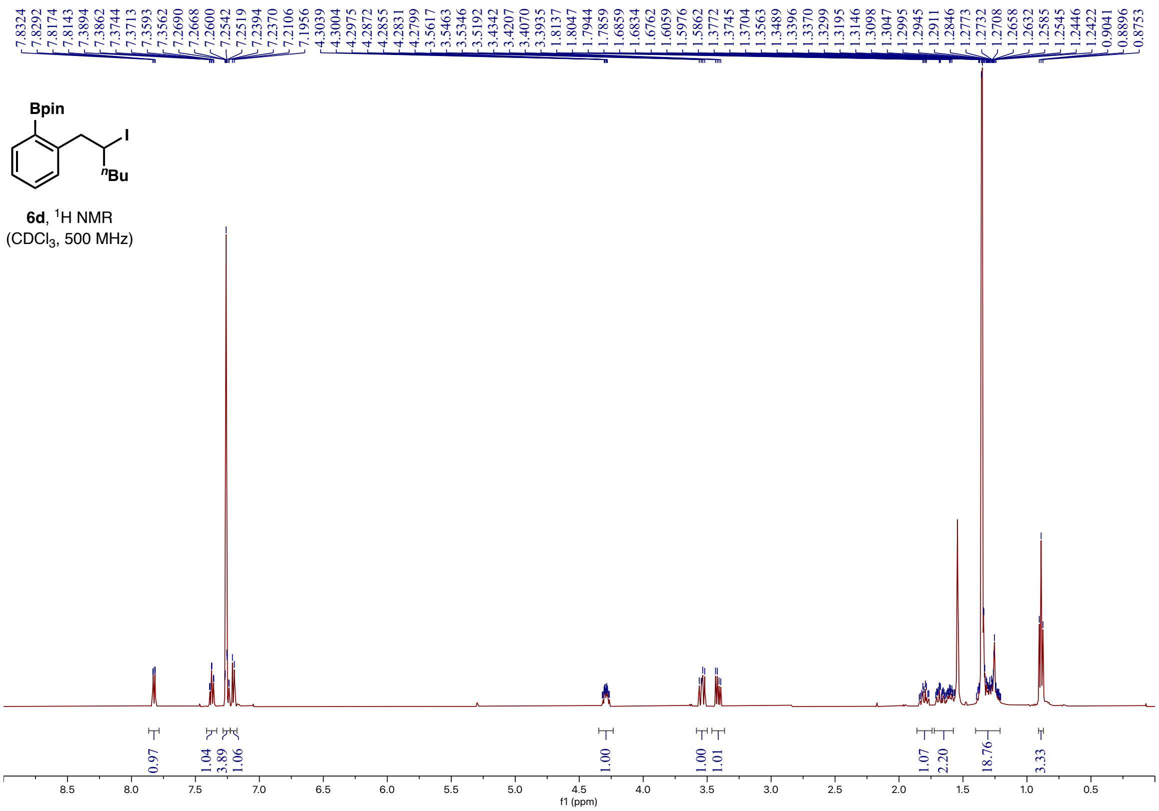

6d, ^{13}C NMR
($CDCl_3$, 126 MHz)

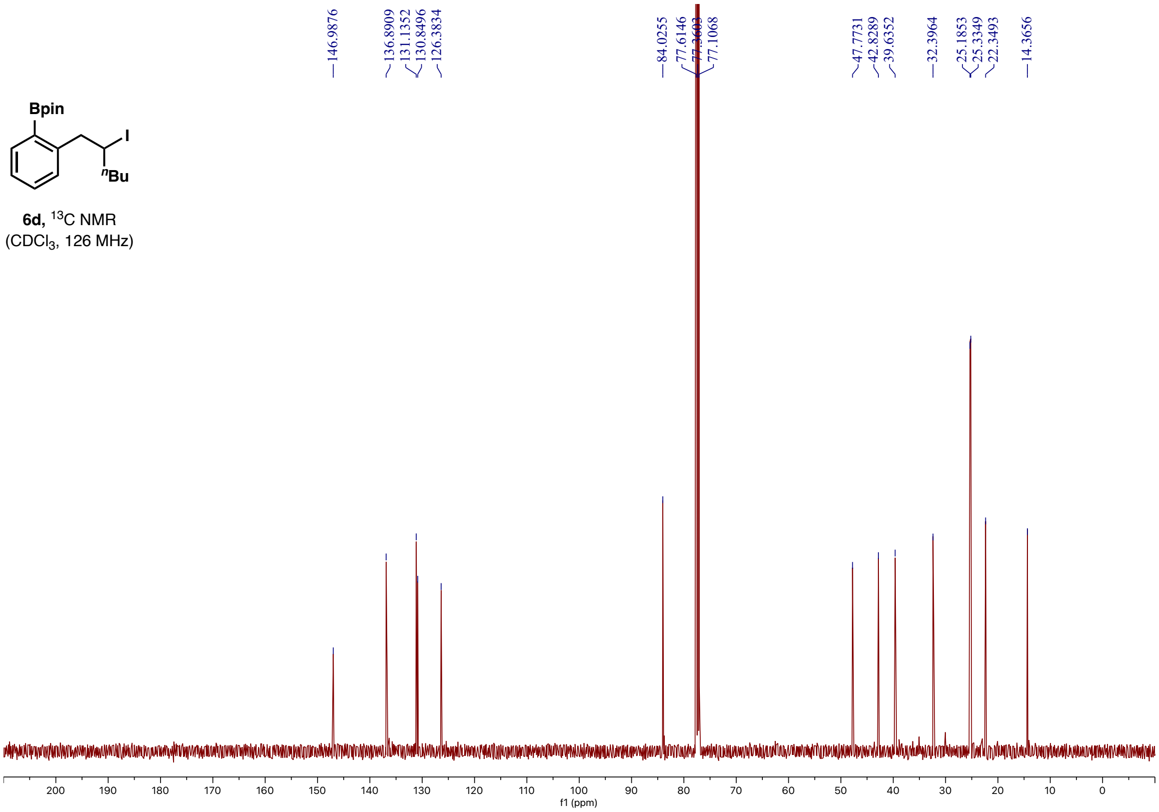

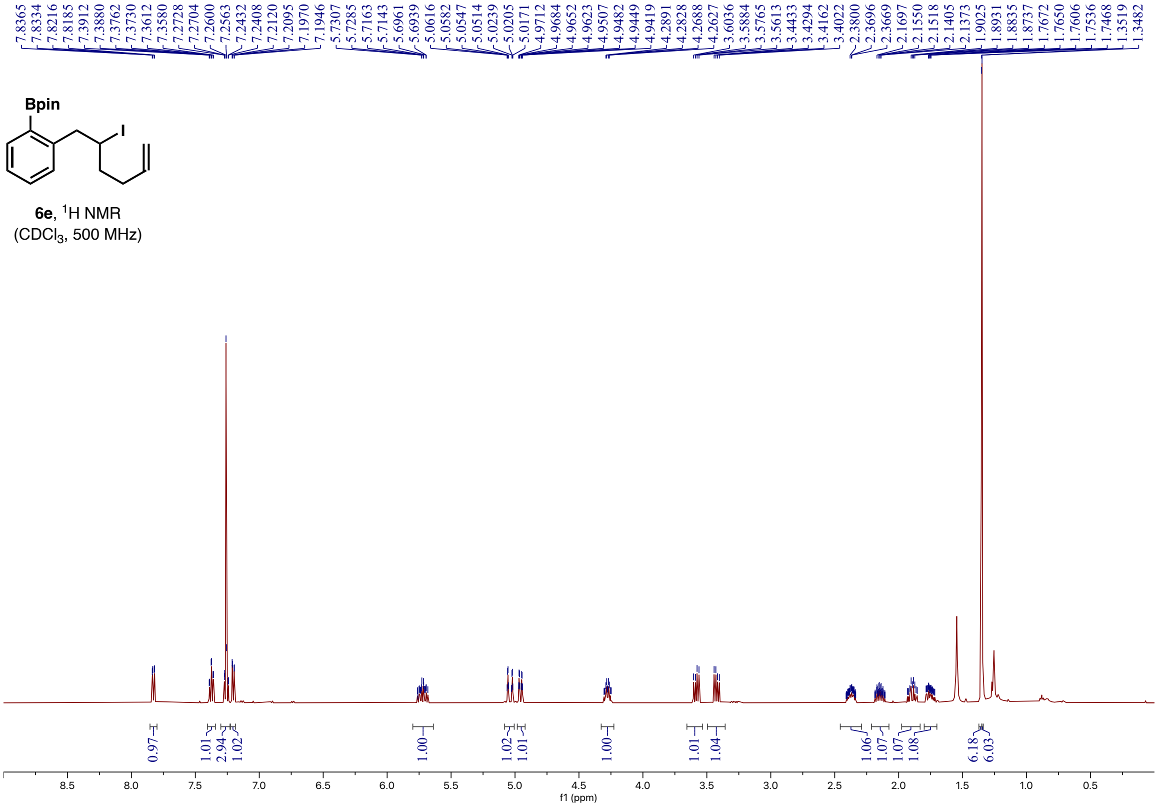

6e, ^{1}H NMR
($CDCl_3$, 500 MHz)

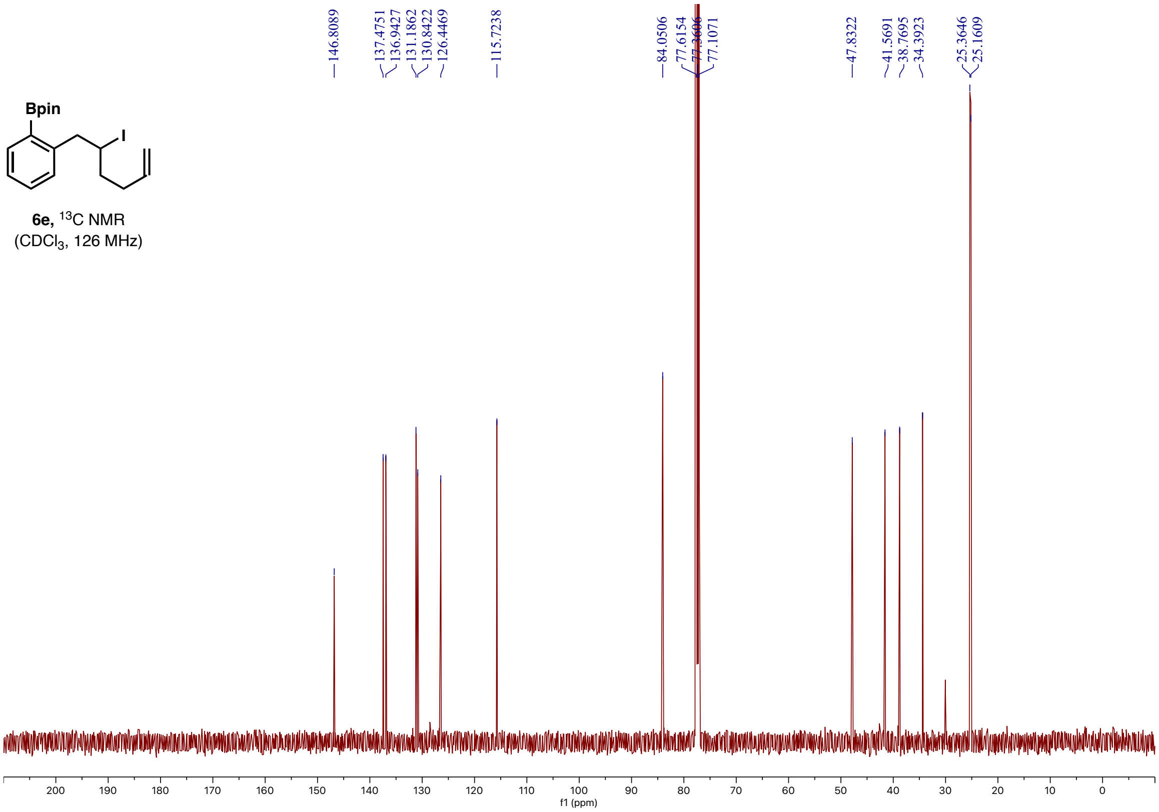

6e, ^{13}C NMR
($CDCl_3$, 126 MHz)

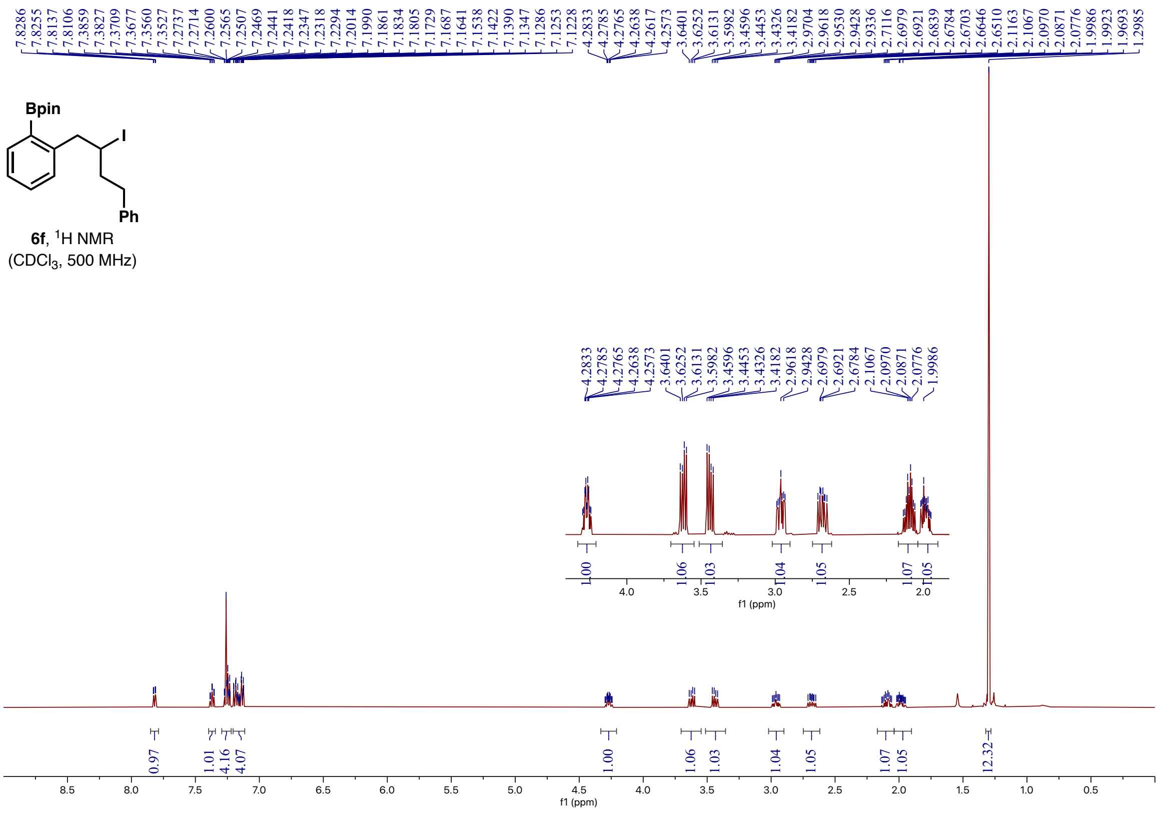
7.8286 7.8255 7.8137 7.8106 7.3859 7.3827 7.3709 7.3677 7.3560 7.3527 7.2737 7.2714 7.2600 7.2565 7.2507 7.2469 7.2441 7.2418 7.2347 7.2318 7.2294 7.2014 7.1990 7.1861 7.1834 7.1805 7.1729 7.1687 7.1641 7.1538 7.1422 7.1390 7.1347 7.1286 7.1253 7.1228 4.2833 4.2785 4.2765 4.2638 4.2617 4.2573 3.6401 3.6252 3.6131 3.5982 3.4596 3.4453 3.4326 3.4182 2.9704 2.9618 2.9530 2.9428 2.9336 2.7116 2.6979 2.6921 2.6839 2.6784 2.6703 2.6646 2.6510 2.1163 2.1067 2.0970 2.0871 2.0776 1.9986 1.9923 1.9693 1.2985
Bpin
I
Ph
6f, 1H NMR
(CDCl3, 500 MHz)
0.97
1.01
4.16
4.07
1.00
1.06
1.03
1.04
1.05
1.07
1.05
12.32
f1 (ppm)

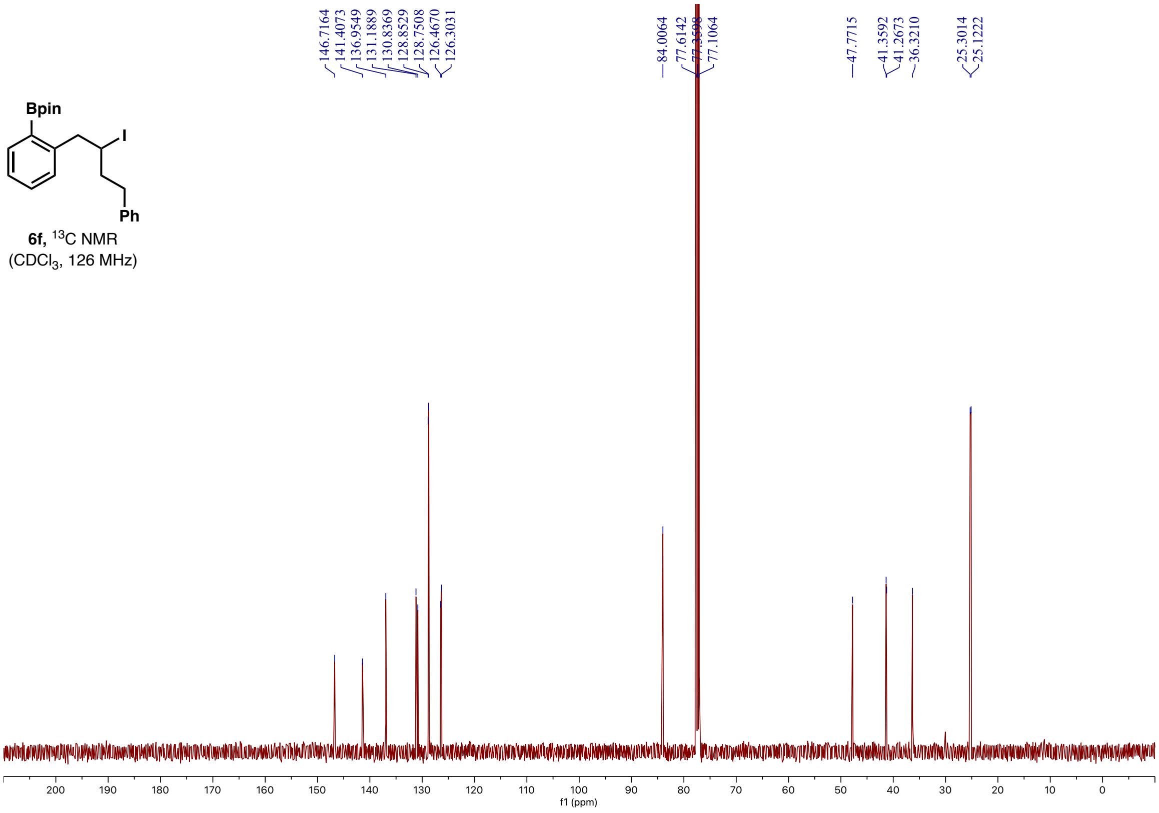
146.7164 141.4073 136.9549 131.1889 130.8369 128.8529 128.7508 126.4670 126.3031
84.0064 77.6142 77.3598 77.1064
47.7715 41.3592 41.2673 36.3210
25.3014 25.1222
Bpin
I
Ph
6f, 13C NMR
(CDCl3, 126 MHz)
f1 (ppm)

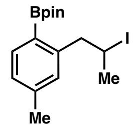

6g, ^{1}H NMR
($CDCl_3$, 500 MHz)

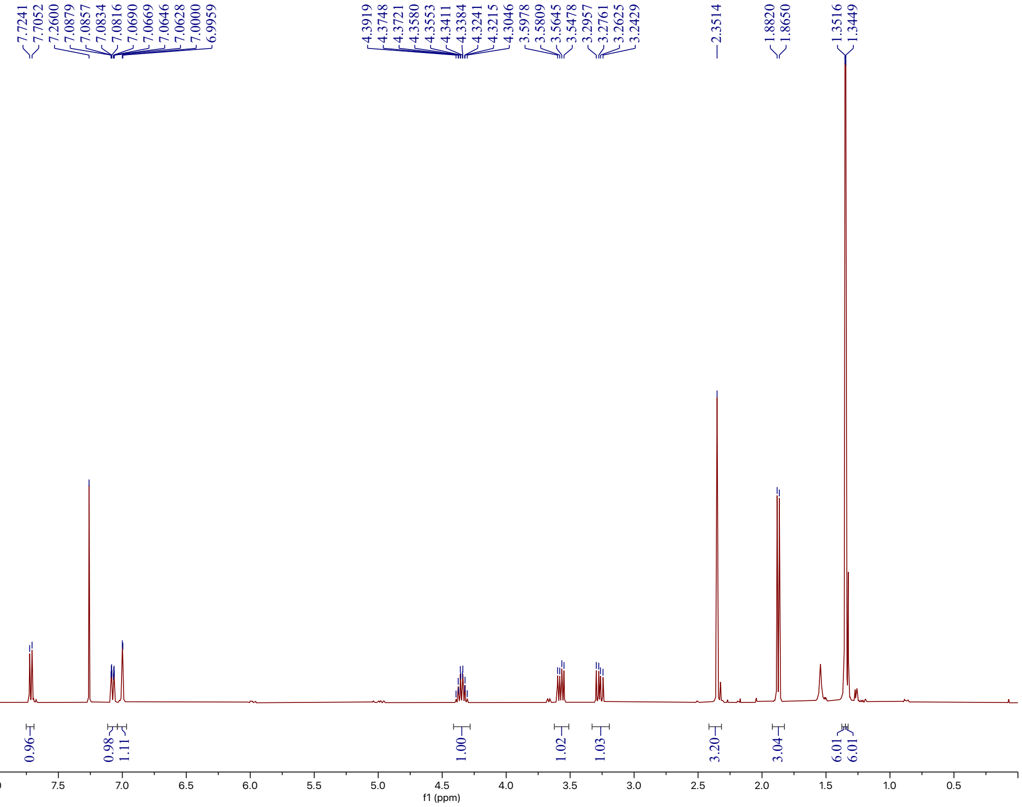

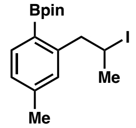

6g, ^{13}C NMR
($CDCl_3$, 126 MHz)

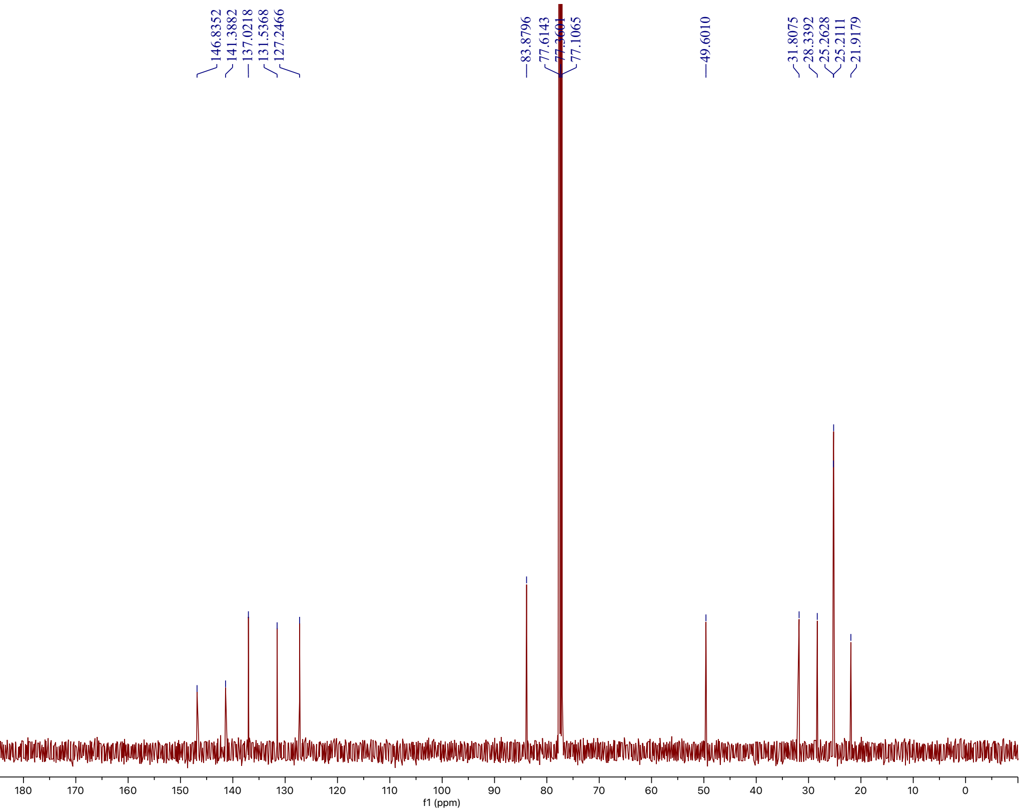

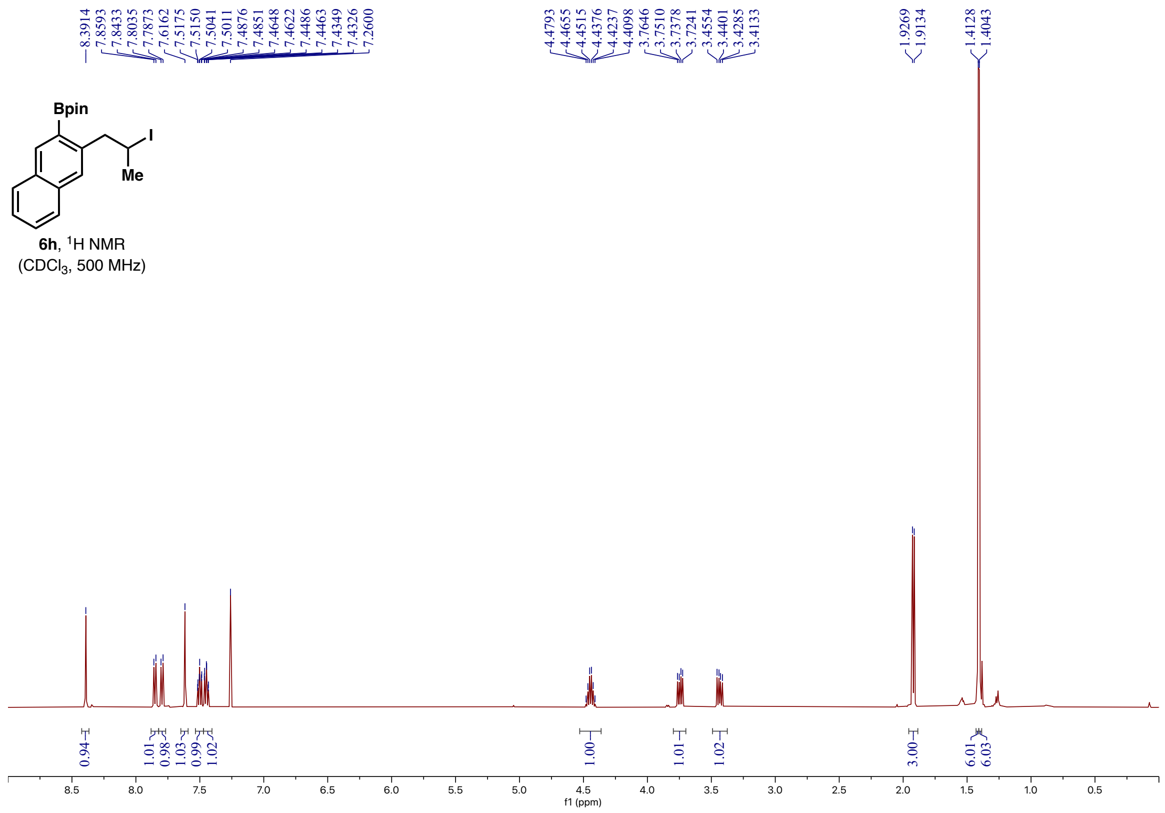

6h, ^1H NMR
($CDCl_3$, 500 MHz)

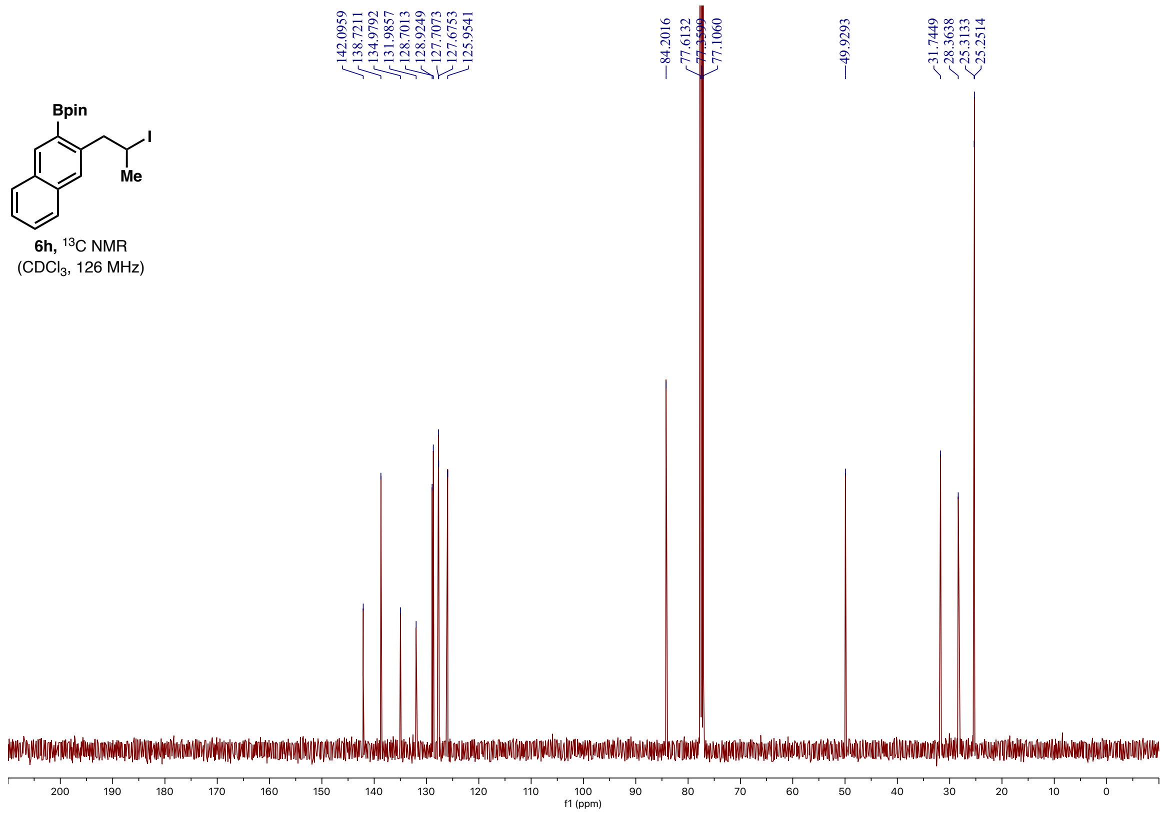

6h, ^{13}C NMR
($CDCl_3$, 126 MHz)

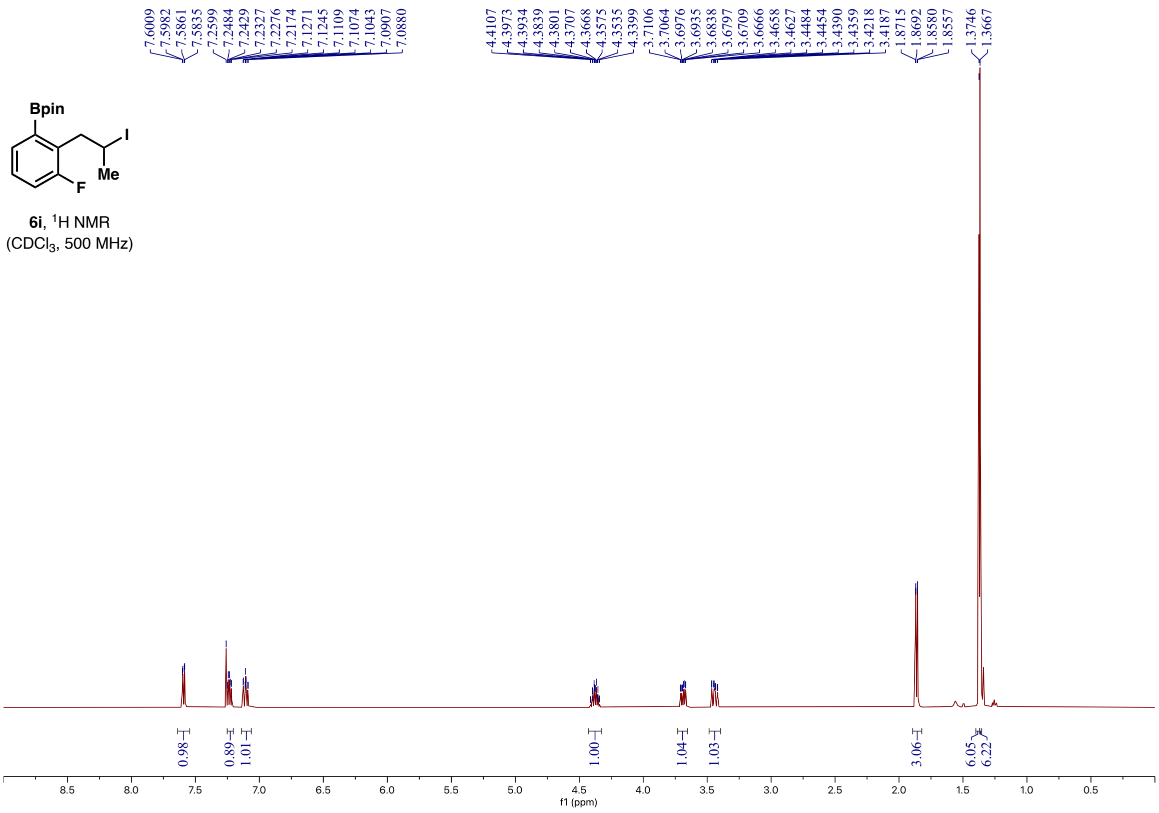

6i, ¹H NMR
(CDCl$_3$, 500 MHz)

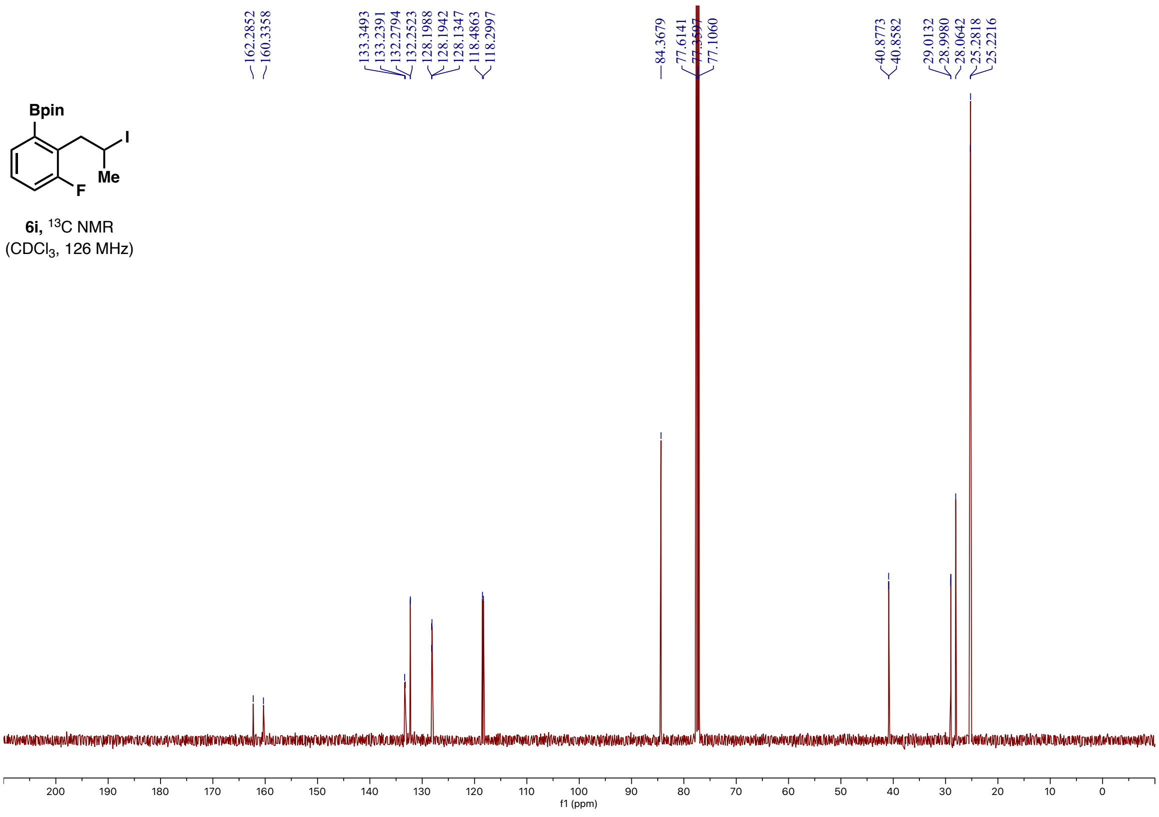

6i, ¹³C NMR
(CDCl$_3$, 126 MHz)

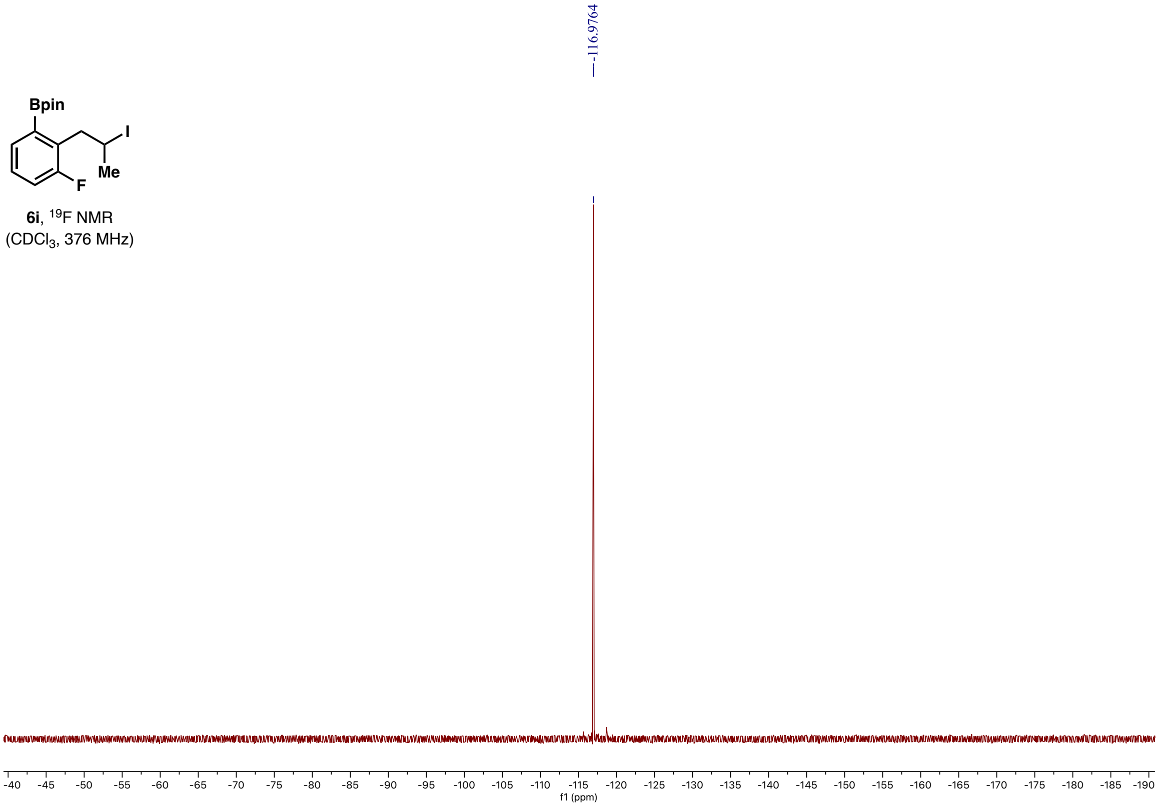

6i, ^{19}F NMR
($CDCl_3$, 376 MHz)

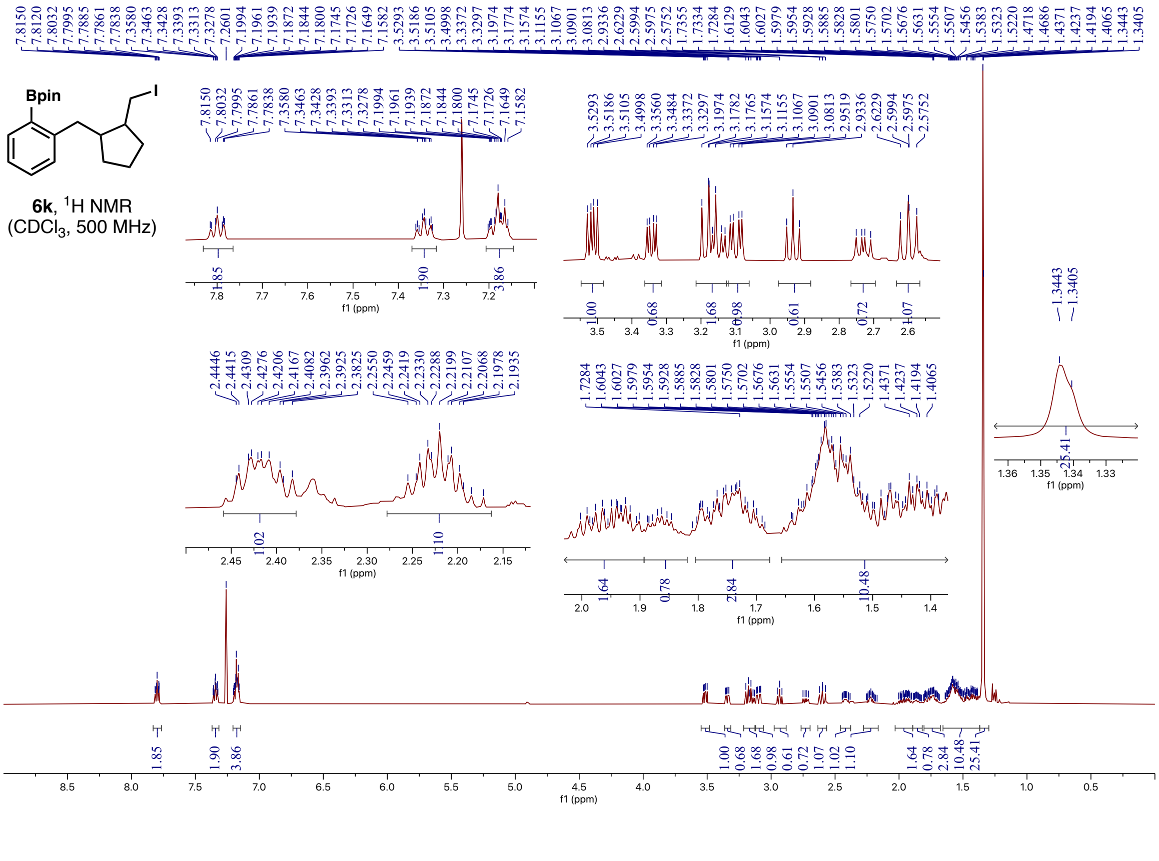

6k, ^{1}H NMR
($CDCl_3$, 500 MHz)

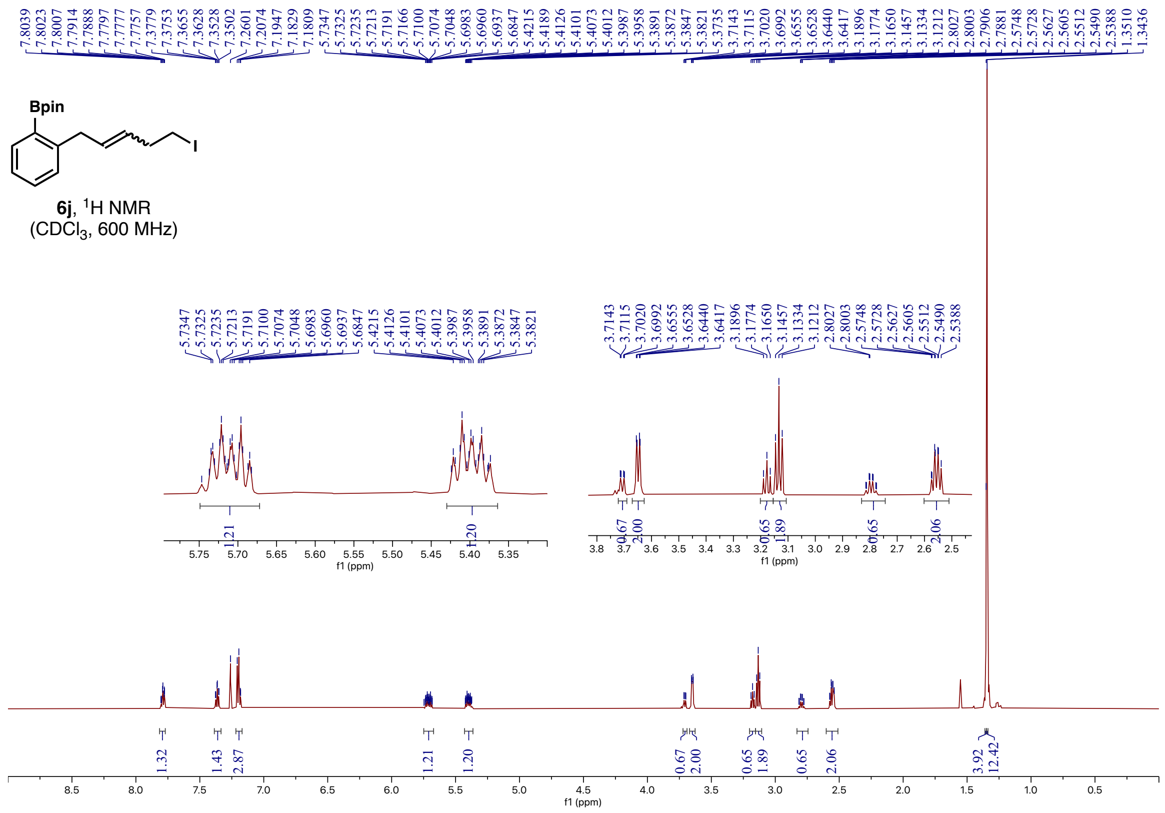

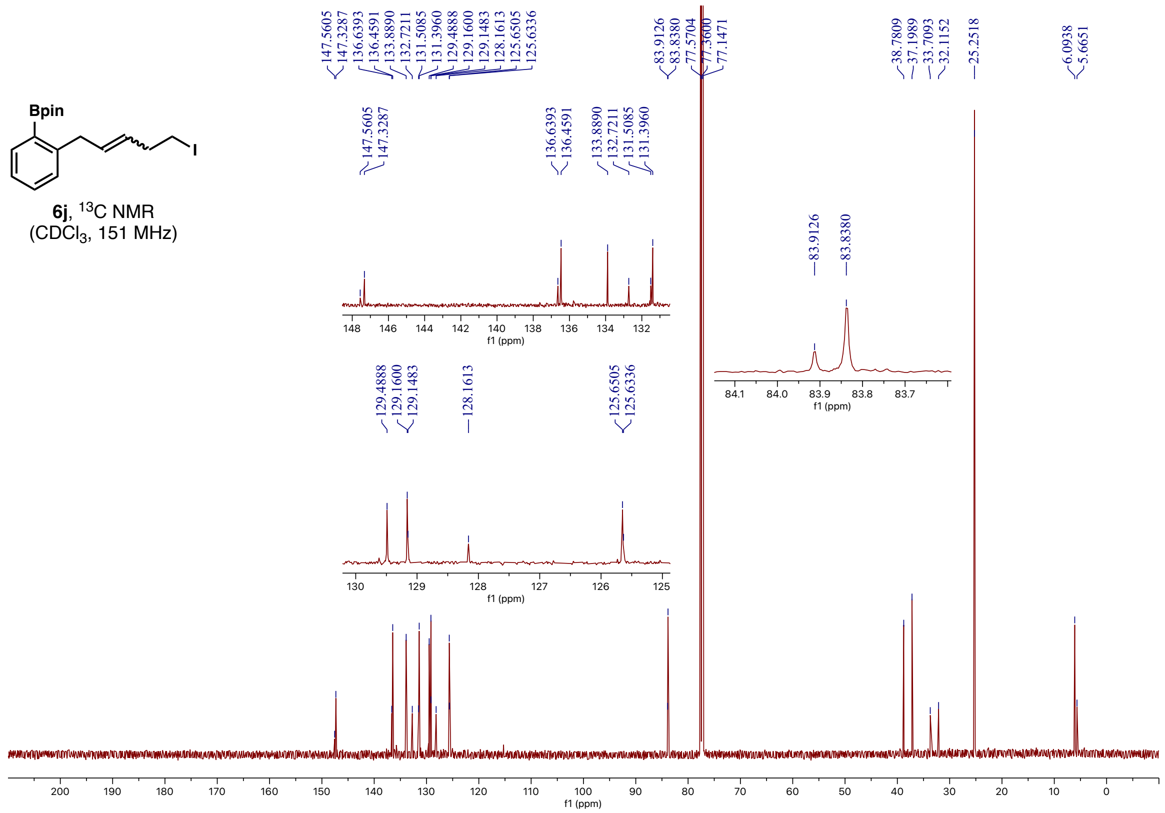

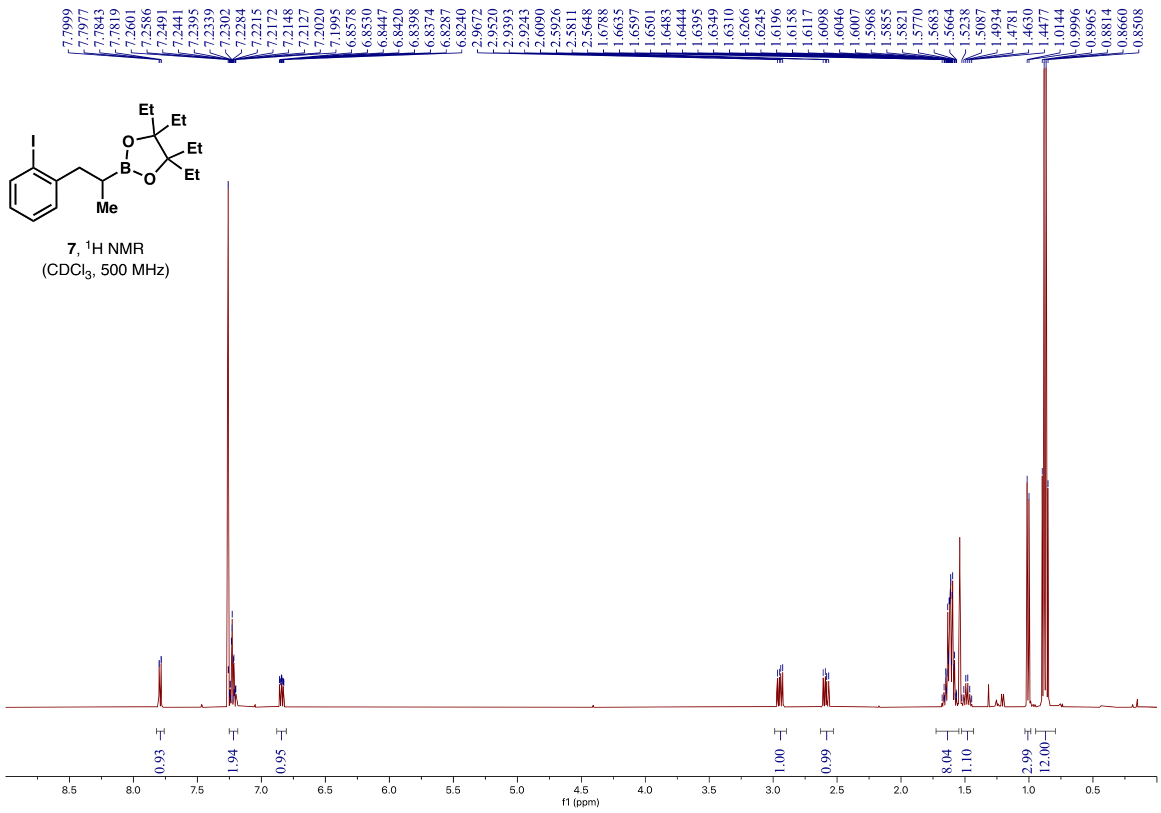

7, ^{1}H NMR
($CDCl_3$, 500 MHz)

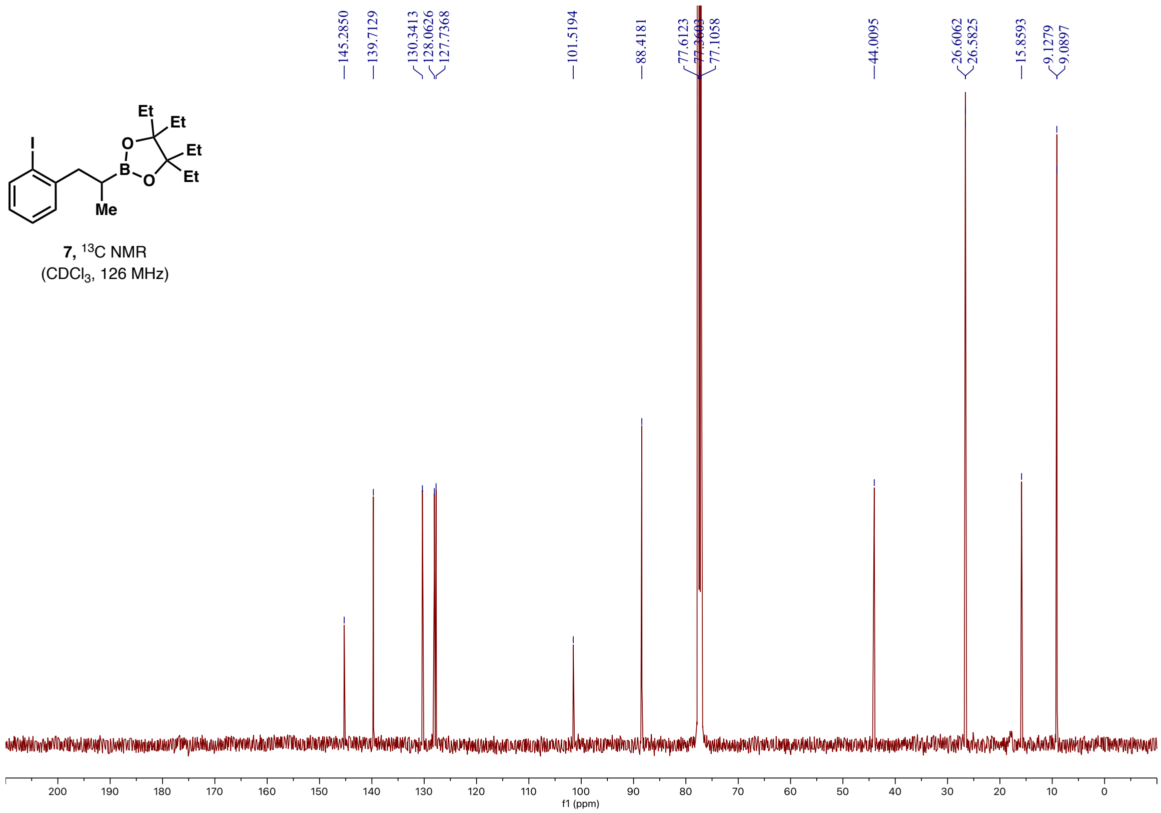

7, ^{13}C NMR
($CDCl_3$, 126 MHz)

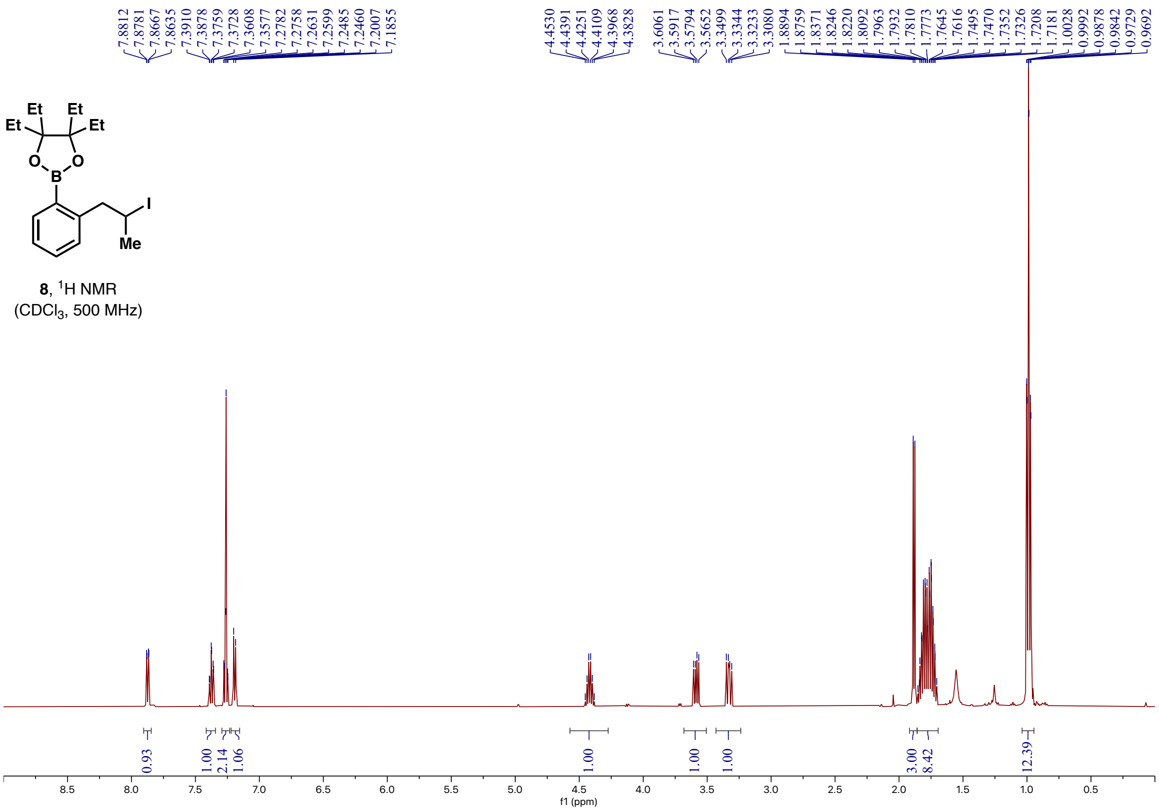
7.8812
7.8781
7.8667
7.8635
7.3910
7.3878
7.3759
7.3728
7.3608
7.3577
7.2782
7.2758
7.2631
7.2599
7.2485
7.2460
7.2007
7.1855
4.4530
4.4391
4.4251
4.4109
4.3968
4.3828
3.6061
3.5917
3.5794
3.5652
3.3499
3.3344
3.3233
3.3080
1.8894
1.8759
1.8371
1.8246
1.8220
1.8092
1.7963
1.7932
1.7810
1.7773
1.7645
1.7616
1.7495
1.7470
1.7352
1.7326
1.7208
1.7181
1.0028
0.9992
0.9878
0.9842
0.9729
0.9692
Et
Et
Et
Et
O
B
O
I
Me
8, ^{1}H NMR
($CDCl_3$, 500 MHz)
0.93
1.00
2.14
1.06
1.00
1.00
1.00
3.00
8.42
12.39
f1 (ppm)

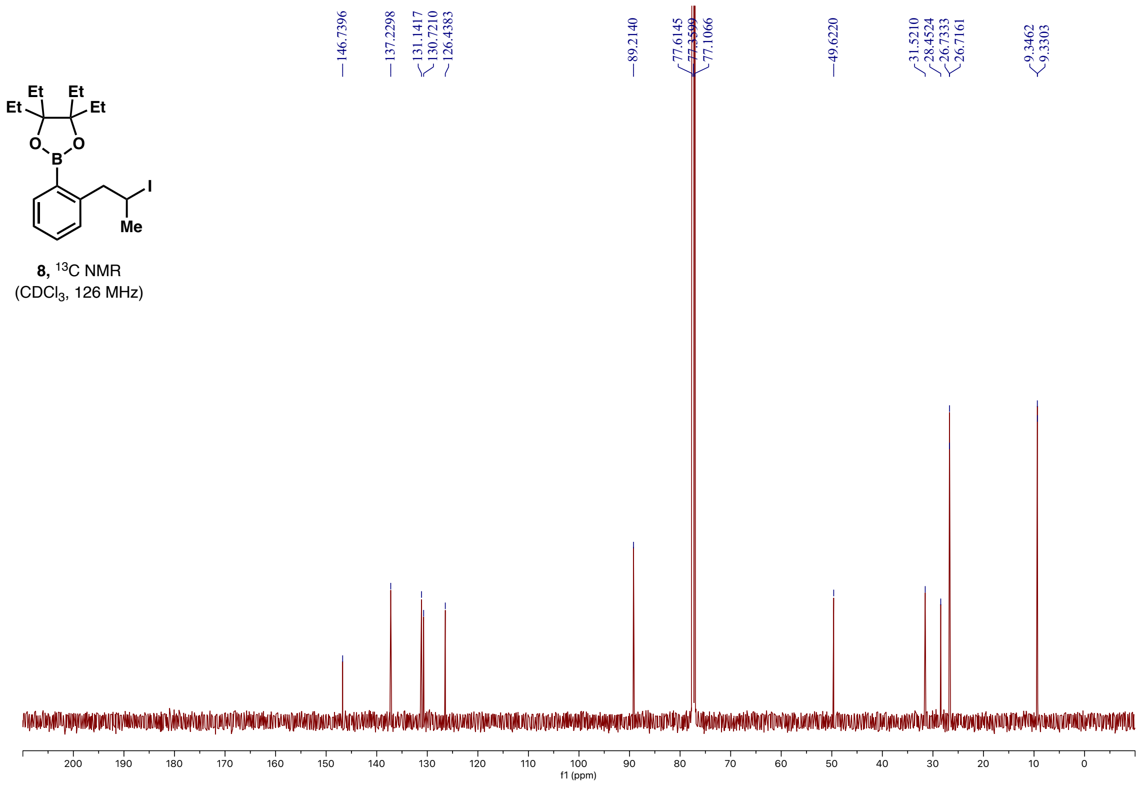
146.7396
137.2298
131.1417
130.7210
126.4383
89.2140
77.6145
77.3599
77.1066
49.6220
31.5210
28.4524
26.7333
26.7161
9.3462
9.3303
Et
Et
Et
Et
O
B
O
I
Me
8, ^{13}C NMR
($CDCl_3$, 126 MHz)
f1 (ppm)

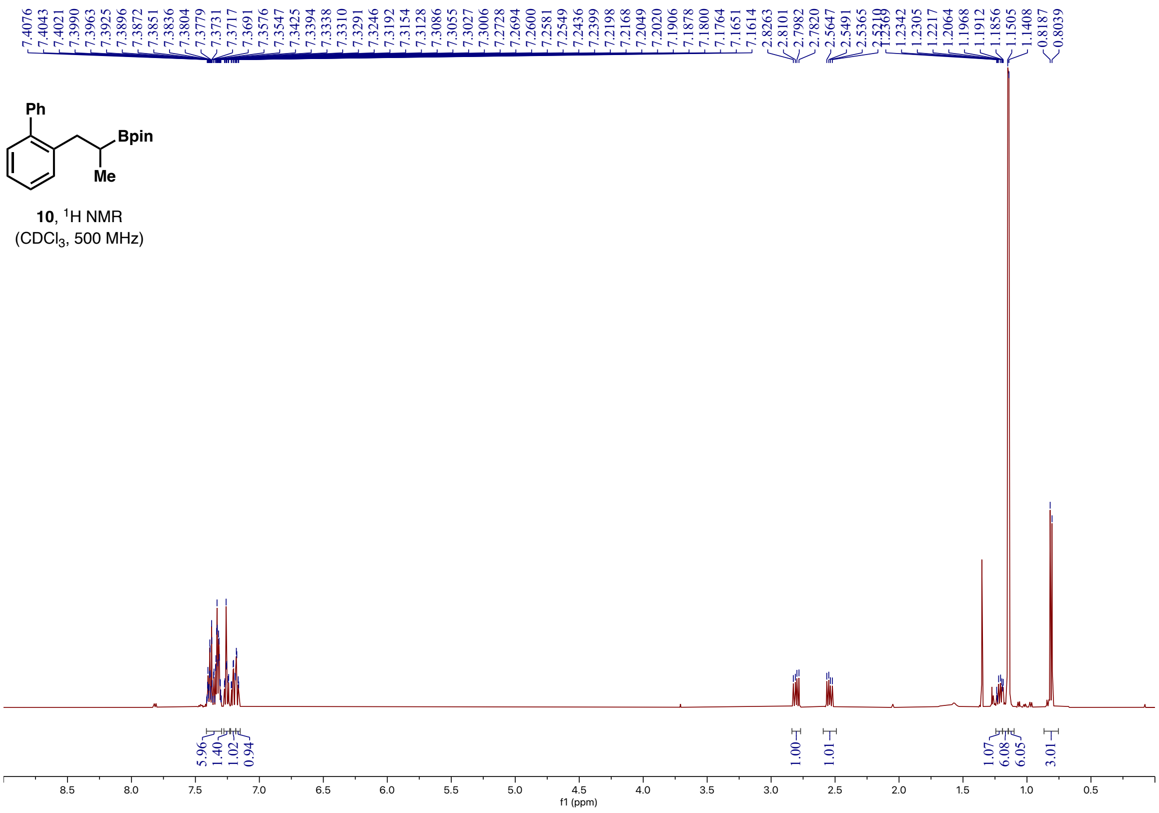
7.4076
7.4043
7.4021
7.3990
7.3963
7.3925
7.3896
7.3872
7.3851
7.3836
7.3804
7.3779
7.3731
7.3717
7.3691
7.3576
7.3547
7.3425
7.3394
7.3338
7.3310
7.3291
7.3246
7.3192
7.3154
7.3128
7.3086
7.3055
7.3027
7.3006
7.2728
7.2694
7.2600
7.2581
7.2549
7.2436
7.2399
7.2198
7.2168
7.2049
7.2020
7.1906
7.1878
7.1800
7.1764
7.1651
7.1614
2.8263
2.8101
2.7982
2.7820
2.5647
2.5491
2.5365
1.2342
1.2305
1.2217
1.2064
1.1968
1.1912
1.1856
1.1505
1.1408
0.8187
0.8039
Ph
Bpin
Me
10, ^{1}H NMR
(CDCl$_3$, 500 MHz)
5.96
1.40
1.02
0.94
1.00
1.01
1.07
6.08
6.05
3.01
f1 (ppm)

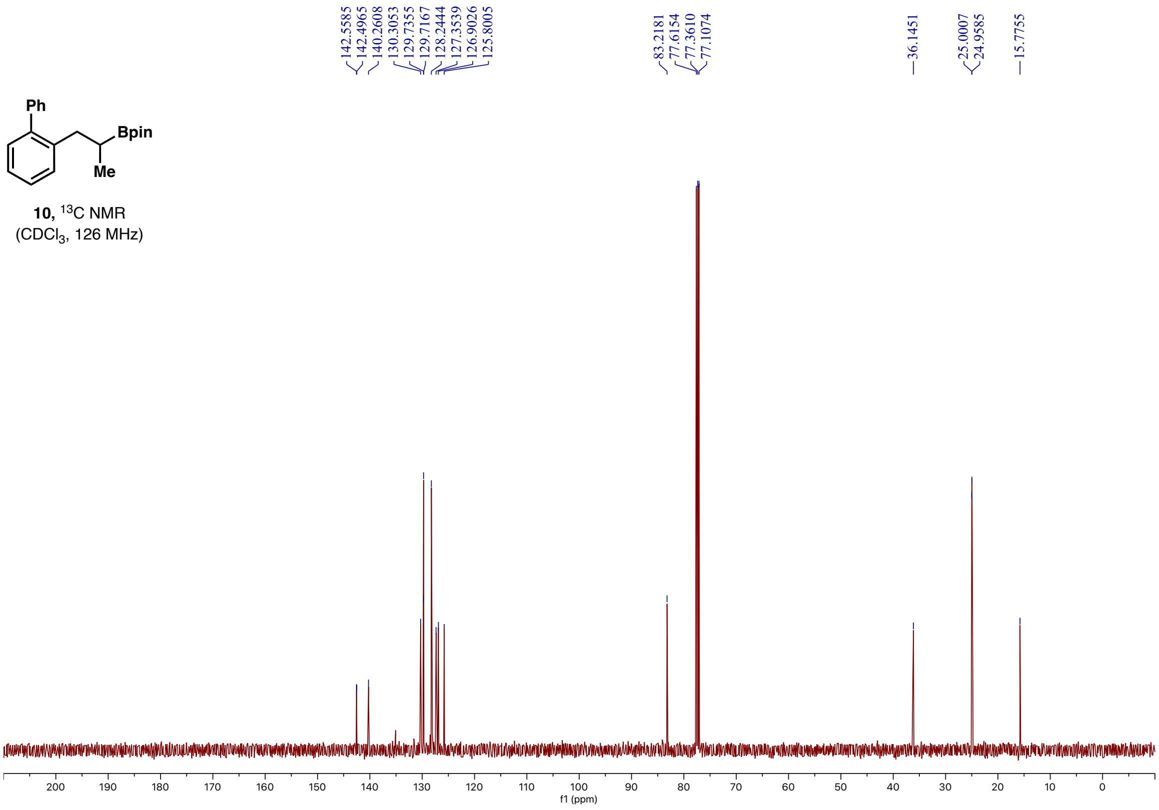
142.5585
142.4965
140.2608
130.3053
129.7355
129.7167
128.2444
127.3539
126.9026
125.8005
83.2181
77.6154
77.3610
77.1074
36.1451
25.0007
24.9585
15.7755
Ph
Bpin
Me
10, ^{13}C NMR
(CDCl$_3$, 126 MHz)
f1 (ppm)

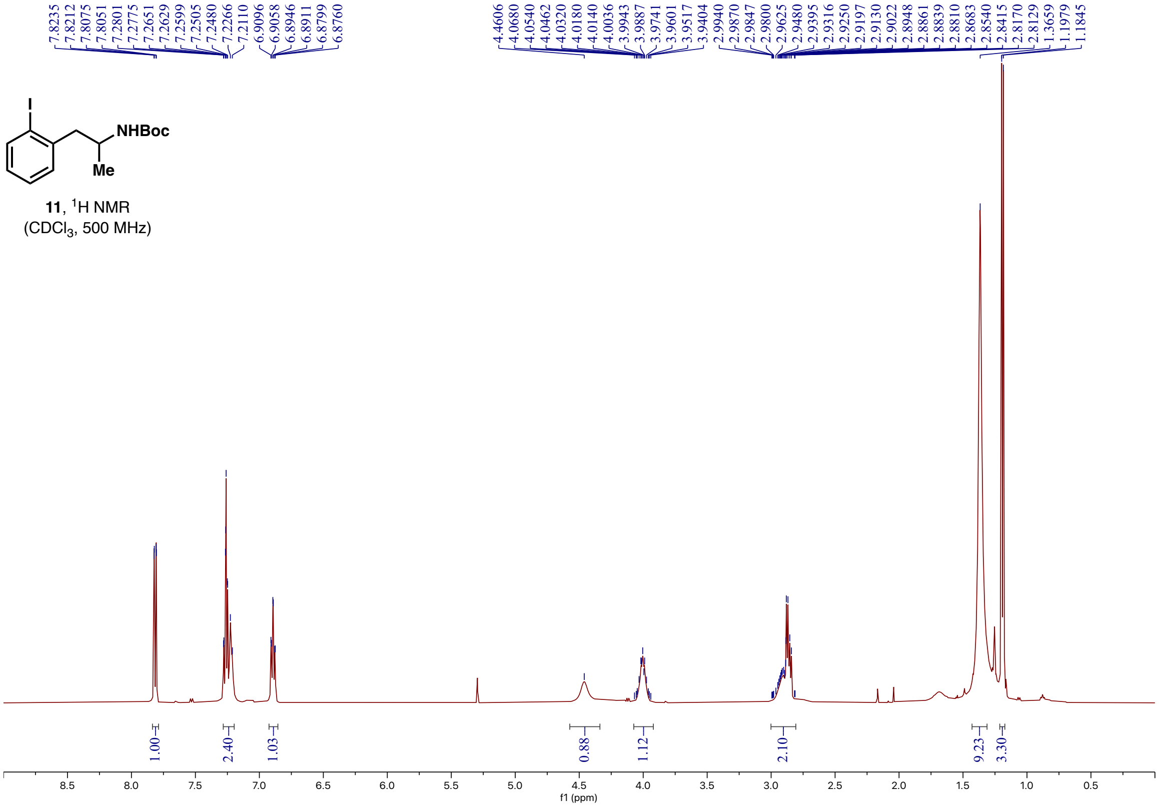

11, ^{1}H NMR
($CDCl_3$, 500 MHz)

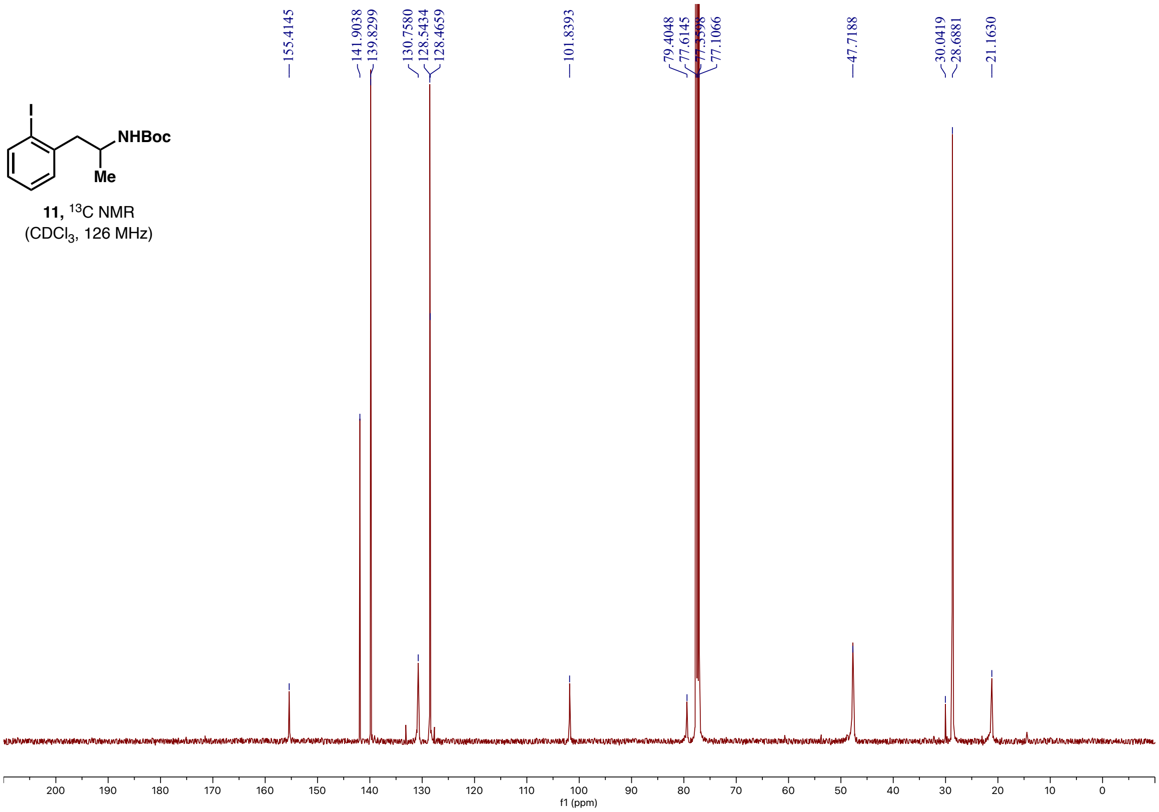

11, ^{13}C NMR
($CDCl_3$, 126 MHz)

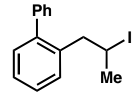

12, ^{1}H NMR
($CDCl_3$, 600 MHz)

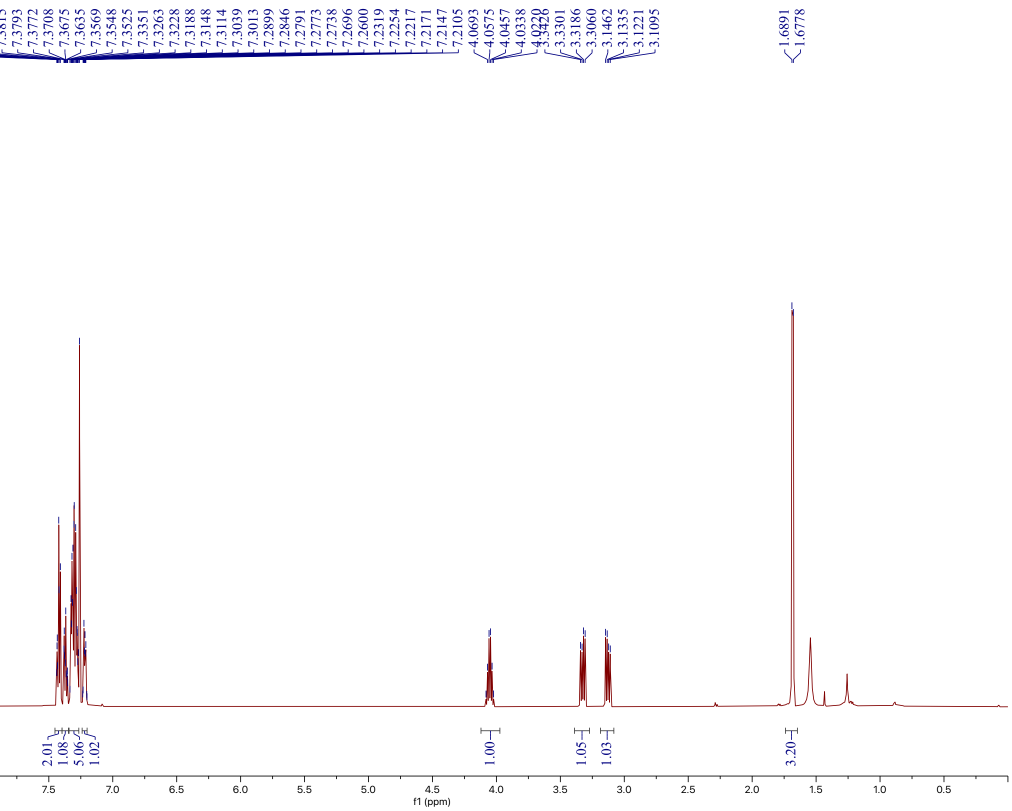

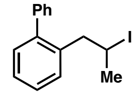

12, ^{13}C NMR
($CDCl_3$, 151 MHz)

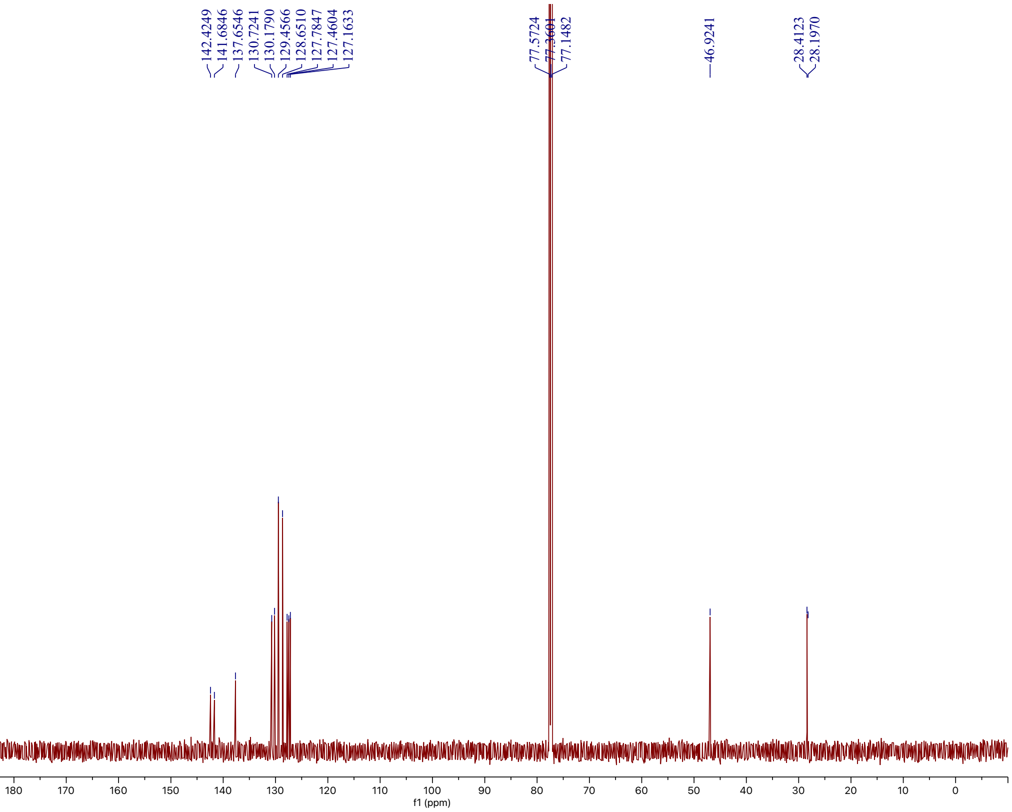

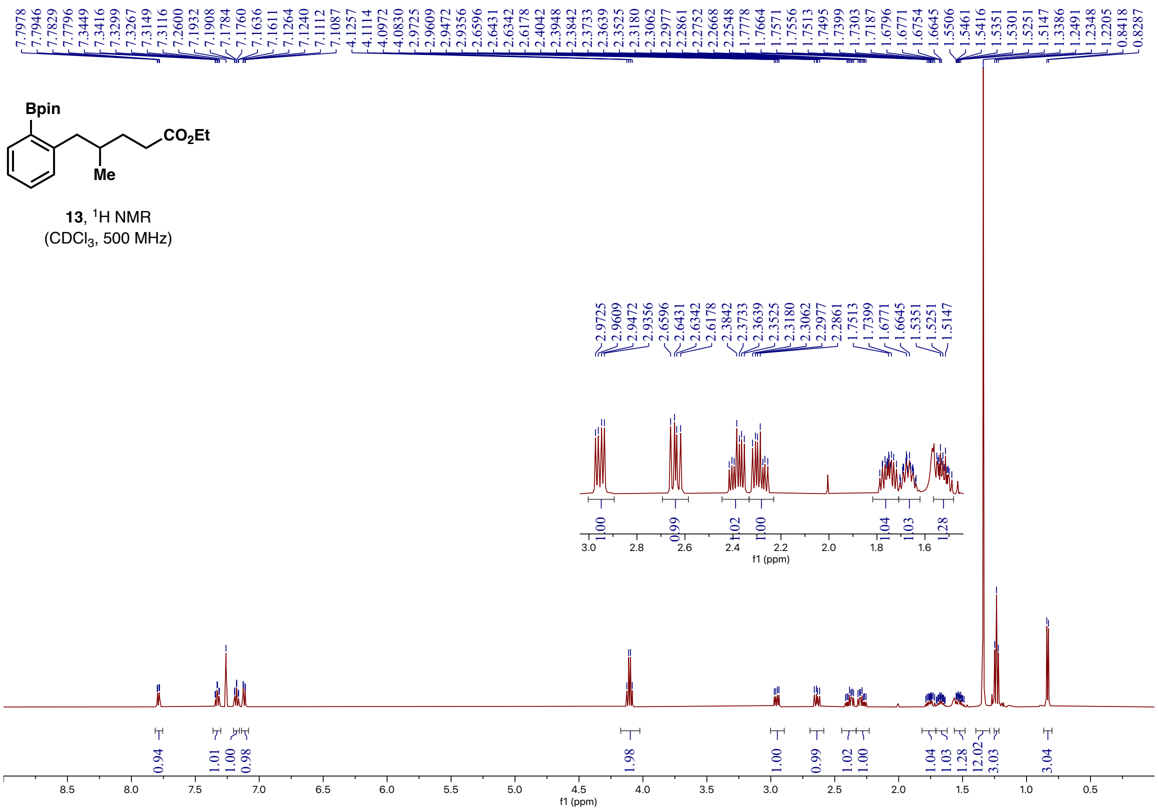
Bpin
CO_2Et
Me
13, ^1H NMR
($CDCl_3$, 500 MHz)
f1 (ppm)

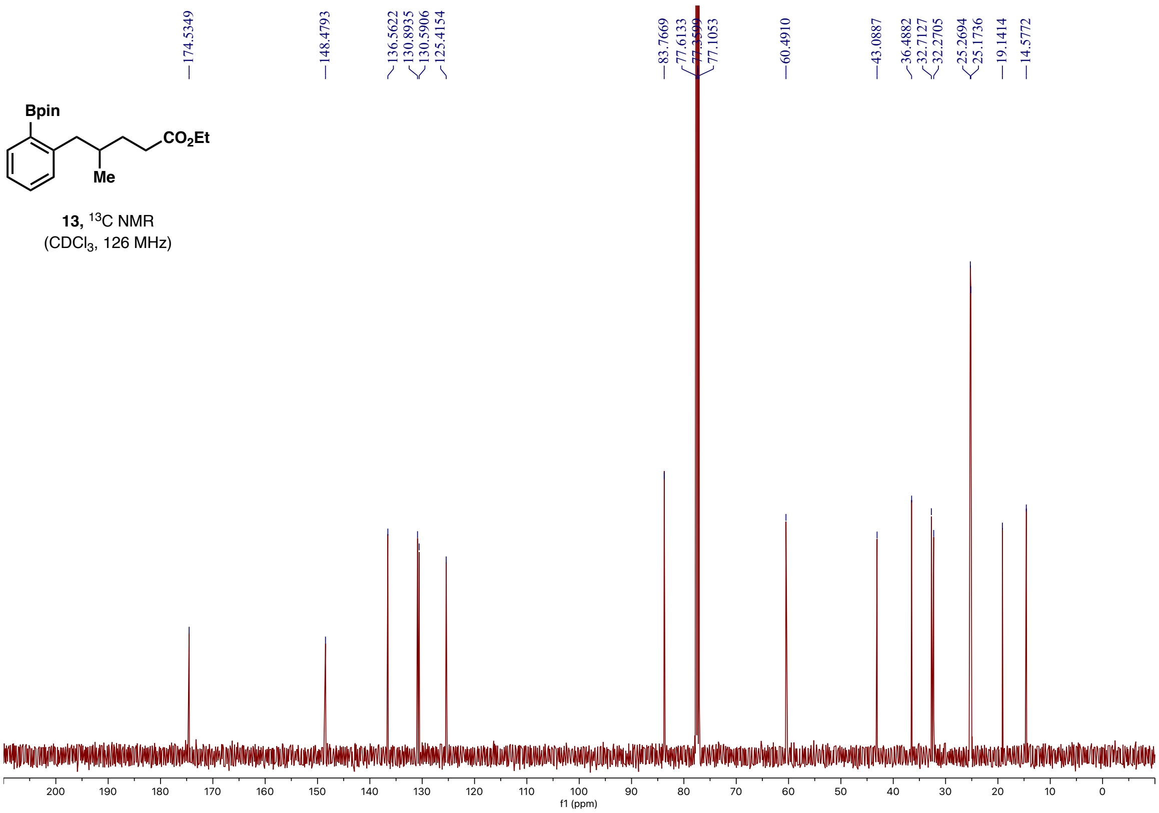
Bpin
CO_2Et
Me
13, ^{13}C NMR
($CDCl_3$, 126 MHz)
f1 (ppm)
